# From Fundamental Photophysics to Photocatalysis: Energy Gap Law Analysis of Anion Radical Excited States

**DOI:** 10.1021/acscentsci.6c00092

**Published:** 2026-06-15

**Authors:** Naresh Duvva, Tanjila Islam, Silvano R. Valandro, Habtom B. Gobeze, Abul Mansur Muhammed Fahim, Xiaodan Wang, Trang Le, Aimée L. Tomlinson, Kirk S. Schanze

**Affiliations:** † Department of Chemistry, The University of Texas at San Antonio, One UTSA Circle, San Antonio, Texas 78249, United States; ‡ Department of Chemistry and Biochemistry, 32193 University of North Georgia, Dahlonega, Georgia 30597, United States

## Abstract

Excited-state ion radicals have recently attracted attention as powerful reductants or oxidants for electron-transfer photocatalysis. A key challenge in their application as photocatalysts is the short lifetime of their excited states, which directly impacts the efficiency of bimolecular photoinduced electron transfer. Here, we present a comprehensive photophysical study aimed at elucidating the factors that govern the lifetime of the doublet excited state in a series of 10 anion radicals derived from structurally related π-conjugated donor–acceptor–donor (DAD) molecules. In these systems, the acceptor is a 2,1,3-benzothiadiazole (BTD) unit flanked by arylene or oligo­(arylene) groups. The anion radicals, generated by chemical reduction in deoxygenated DMF, are stable and were characterized using UV–visible–near-IR absorption/emission spectroscopy and femtosecond transient absorption spectroscopy. Remarkably, the doublet excited state lifetimes vary by nearly 200-fold across the series, ranging from 2.9 to 565 ps. Nonradiative decay rates increase as the excited-state energy decreases, in a manner that is quantitatively consistent with the energy gap law for nonradiative decay. Faster decay is observed for anion radicals with highly delocalized π-systems, reflecting their lower excited-state energies. Finally, we demonstrate the catalytic utility of DAD anion radicals with long-lived doublet excited states in a consecutive photoinduced electron transfer (ConPET) process, using the photodebromination of 4-bromoacetophenone as a model reaction.

## Graphical abstract

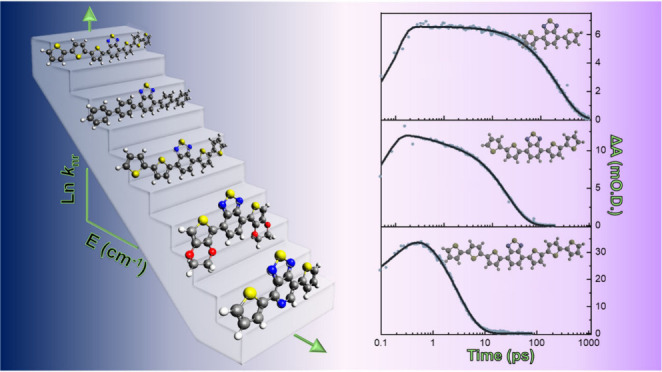

## Introduction

Electron transfer (ET) photocatalysis^1^ has been pivotal to chemical science for decades due to its widespread application in artificial photosynthesis,^2,3^ dye-sensitized solar cells,^4^ and synthetic chemistry.^5–8^ Previous research has explored a variety of photosensitizers, including transition metal and organometallic complexes,^9–11^ organic chromophores,^8,12^ conjugated polymers^13^ and semiconductor nanomaterials.^14^ Ion-radicals have recently emerged as a distinctive class of photosensitizer with intriguing photophysical properties.^15,16^ Unlike neutral chromophores, ion-radicals exhibit unique electronic structures that give rise to broad, intense absorption bands in the visible and near-infrared regions.^17^ The doublet excited states of ion-radicals display a range of lifetimes and propensity to engage in single electron transfer (ET) processes, making them attractive for applications in photocatalysis.^18–25^ Recent advances in synthetic methods, spectroscopic characterization, and computational modeling enable deeper insights into the structure–property relationships governing ion-radical photophysics.

The application of an ion-radical as a sensitizer for ET photocatalysis is facilitated when its doublet excited state has a lifetime that is sufficiently long to engage in diffusion controlled bimolecular encounters with electron donors or acceptors. As described by Rehm–Weller theory, moderately exothermic bimolecular photoinduced ET reactions occur at the diffusion-controlled rate (*k*_diff_) in solution.^26^ The Stern–Volmer equation relates quenching efficiency to quencher concentration and the excited state lifetime.^27^ Applying this relationship shows that at practical substrate concentrations (>50 mM), quenching efficiencies above 20% require sensitizer lifetimes longer than 250 ps (see Figure 1). While shorter lifetime excited states can still engage in bimolecular photoinduced ET, the efficiency may be low, giving rise to overall inefficient photocatalysis.^28–30^

**Figure 1:**
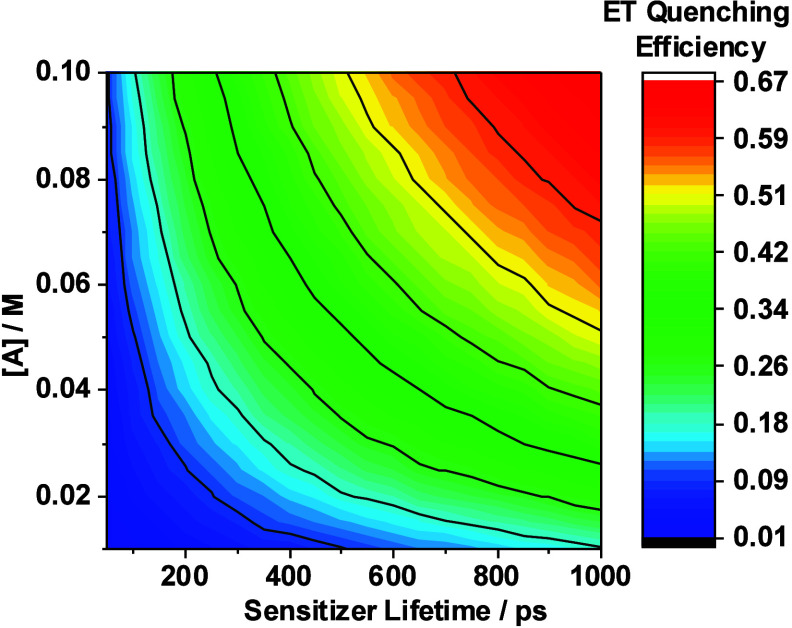
Surface map plot of the variation of the electron transfer excited state quenching efficiency as a function of sensitizer lifetime (*x*-axis) and acceptor concentration, [A] (*y*-axis). The efficiency map is computed by using the Stern–Volmer relationship assuming diffusion controlled bimolecular quenching (*k*_diff_ = 2 × 10^10^ M^–1^s^–1^).

In recent work we explored bimolecular ET quenching of the doublet excited states of anion radicals formed by chemical reduction of neutral chromophores.^20,25,31^ This work has relied on femtosecond transient absorption (TA) spectroscopy to monitor the lifetimes of anion radical doublet excited states in the presence and absence of electron acceptors. One goal of that work has been to understand the structure–property relationships that control the lifetimes of the doublet excited states of a variety of anion radical chromophores. In a separate research we have explored the photophysics of π-conjugated oligo­(arylene)­s that feature a donor–acceptor–donor (DAD) electronic structure.^32–35^ These oligomers feature visible light absorption due to charge transfer (CT) interactions between the electron rich and electron poor arylene units.^36^ In an intersection between these lines of research, we fortuitously discovered that anion radicals derived from DAD oligomers display remarkably long doublet excited state lifetimes, some approaching 600 ps. In addition, we also observed that the anion radical doublet excited state lifetimes vary substantially with DAD oligomer structure, in some cases decreasing by more than 100-fold by an increase in the conjugation length.

Stimulated by these preliminary observations, we carried out a systematic study that seeks to understand the factors that govern the lifetime of the doublet excited state in a series of 10 anion radicals derived by chemical reduction of the corresponding neutral DAD type π-conjugated oligo­(arylene)­s. This study fully characterized the electrochemical and photophysical properties of the neutrals and corresponding anion radicals. All anion radicals absorb broadly in the visible, with some extending into the near-IR region, and they exhibit weak fluorescence with a small Stokes shift. Femtosecond TA spectroscopy was applied to determine the excited state spectra and decay dynamics of the series of 10 anion radicals. Across the series the doublet excited state lifetimes range from 565 to 3 ps; by using the excited state energies computed from the absorption and/or fluorescence, we demonstrate that the lifetimes are governed by the energy gap law for nonradiative decay.^37,38^ This is the first work to demonstrate an energy gap law correlation for ion-radical excited states, and it provides insight into the pathway for developing ion-radical based sensitizers with sufficiently long lifetimes to serve as visible and near-IR absorbing photocatalysts. We have also utilized one of the DAD-based anion radicals that exhibits a long doublet excited state lifetime as a photosensitizer for a model photocatalytic reduction reaction, demonstrating a mechanism-based approach to consecutive photoinduced ET (ConPET) photocatalysis.^24^

## Results and Discussion

### Structures and Synthesis

The π-conjugated donor–acceptor–donor (DAD) chromophores used in this study are shown in Chart 1. Most compounds feature a 2,1,3-benzothiadiazole (BTD) moiety that is linked on the 4,7-positions with various arylene units including oligo­(thiophene), phenylene, biphenylene, and fluorenylene. The BTD unit is electron poor, whereas the arylenes are relatively electron rich, and consequently the neutral chromophores exhibit charge-transfer absorption bands in the near-UV or visible region. The chromophores have been widely investigated in the context of donor–acceptor π-conjugated polymers that are of interest in organic/polymer solar cells, photodetectors and transistor applications.^39^

**Chart 1:**
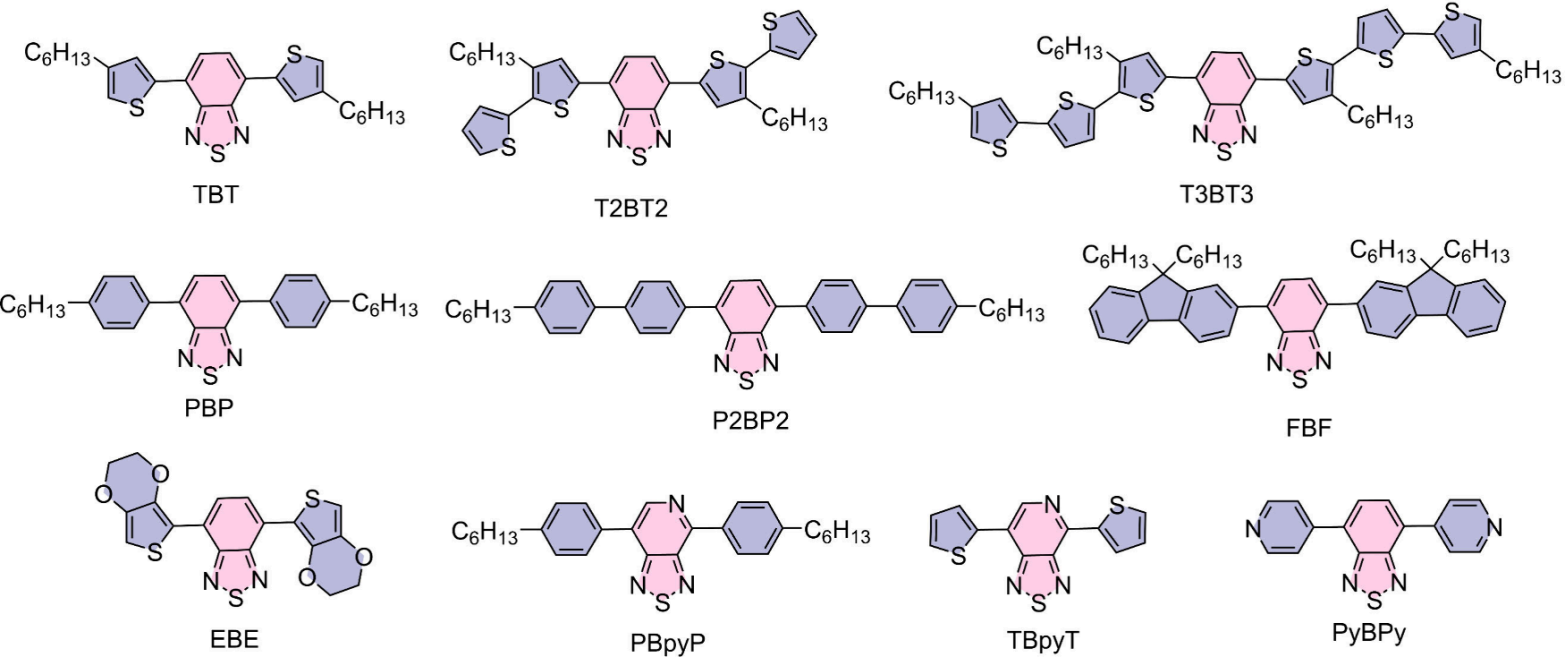


All compounds shown in Chart 1 were prepared by Pd-catalyzed coupling reactions as outlined in Schemes S1–S3. The detailed synthetic procedures are outlined in the Supporting Information. The intermediates and final compounds were fully characterized using ^1^H NMR, ^13^C NMR and high-resolution mass spectrometry.

### Absorption and Emission Spectroscopy

Absorption spectra of the neutral DAD chromophores generally feature two bands, one in the near-UV and a second, weaker band in the visible region. Figure 2 shows the spectra of the compounds TBT and T2BT2, which are characteristic of the series. (The spectra for all compounds shown in Chart 1 are provided in Figure S40 in the Supporting Information.) Both TBT and T2BT2 feature the near-UV and visible absorption bands (see Figure S40 for UV region), and notably both bands are red-shifted in T2BT2 due to the increase in the number of thiophenes in the donor units. Electrochemistry was conducted on the entire series of DAD chromophores, and the results are summarized in the Supporting Information (Figure S43 and Table S1). Each DAD exhibits a reversible reduction between −1.0 and −1.5 V along with a reversible or quasi-reversible oxidation between 0.9 and 1.6 V (potentials vs SCE reference). The oxidation potentials vary with the structure of the donor segment. For example, across the series TBT, T2BT2, T3BT3 the oxidations vary in the sequence 1.21, 0.95, 0.89, consistent with the increased donor strength as the thiophene segments increase in length.

The primary focus of this investigation is the photophysical properties of the anion radicals derived from the DAD compounds (DAD^–•^). These anions were accessed via chemical reduction of the neutrals with sodium acenaphthylenide (E_red_ = −2.62 V vs SCE)^40^ in DMF solution. The DAD^–•^ feature a series of absorption bands that are red-shifted significantly from the absorption bands of the corresponding neutrals. As an example, Figure 2 illustrates absorption spectra for TBT and T2BT2 in their neutral and anion radical forms; the full series of spectra are provided in Figures S41 and S42. Here it is seen that the anion radicals display a strong band that is red-shifted slightly from the lowest absorption of the neutral, and then there are a series of lower intensity absorptions that extend into the red and near-IR regions. In most cases, the long-wavelength features are well resolved and potentially arise from a single electronic transition that is split by vibronic coupling (see below). It is of interest that fluorescence was observed for most of the anion radicals (see Figures 2, S42 and Table S2). In the cases where fluorescence was observed, the 0–0 band appears with a small Stokes shift from the lowest absorption band, e.g. for TBT^–•^ the lowest absorption is at 826 nm and the fluorescence 0–0 band is at 867 nm. In several cases the fluorescence features a vibronic progression, with Δν ∼ 1050 cm^–1^ consistent with coupling to midfrequency modes that are associated with the benzothiadiazole moiety. The fluorescence quantum yields of the DAD^–•^ ranged from 0.00046–0.0024 (Table S3). The quantum yields are low because the DAD^–•^ excited state lifetimes are short and the radiative rate constants are relatively low (*k*_r_ < 10^7^ s^–1^, Table S3).

**Figure 2:**
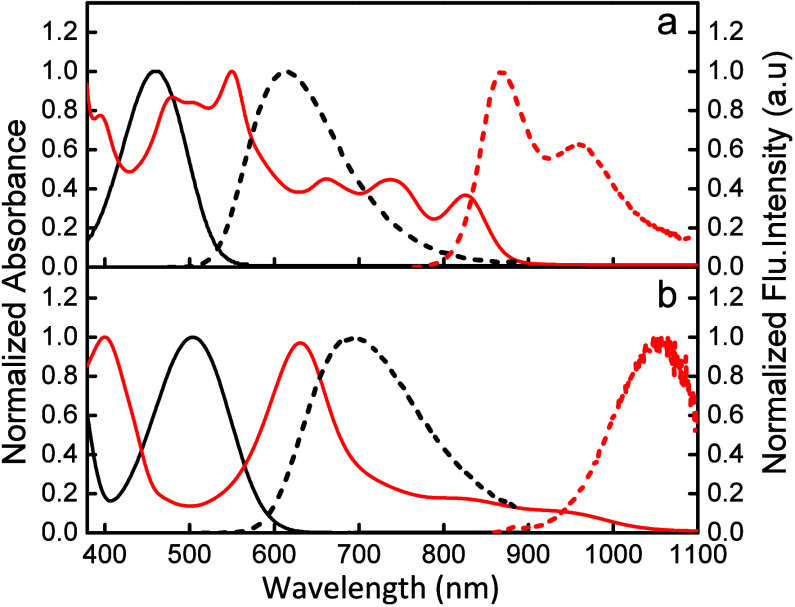
Normalized absorption (solid lines) and fluorescence (dashed lines) spectra of neutral DAD chromophores and radical anions (DAD^–•^) in DMF solution. (a) TBT (black, λ_ex_ = 450 nm for emission), TBT^–•^ (red, λ_ex_ = 742 nm for emission). (b) T2BT2 (black, λ_ex_ = 500 nm for emission), T2BT2^–•^ (red, λ_ex_ = 830 nm for emission).

To provide more insight regarding the nature of transitions involved in the absorption spectra of the anion radicals, density functional theory and time-dependent density functional theory (DFT and TDDFT, respectively) calculations were conducted on the anion radicals. The basis set/functional pairings used in the calculations were selected through benchmarking guided by comparison with experimental spectra (see DFT-SI section). All calculations used a continuum solvent model with static and optical dielectric constants of 36 and 1.75, respectively. To start, we segmented the 10 anion radicals into two sets where the structures were selected based on shared structural characteristics (Series 1 - thiophenyl set: TBT, T2BT2, T3BT3, TBpyT and EBE; Series 2 – phenylene set: PBP, P2BP2, PByP, PyBPy and FBF). The motivation for this segmentation was that no single basis set/functional pairing was successful in the benchmarking for all systems. Using the functionals and basis sets provided in the Supporting Information (DFT-SI, p. 2), it was found that HSEh1PBE/SV and B3LYP/6–31Gd gave the closest match in the benchmarking between the calculated and experimental absorption transitions for Series 1 and 2, respectively.

Figure 3 shows overlays of the calculated and experimental UV–visible absorption spectra and density plots for the frontier molecular orbitals involved in the three lowest energy transitions for the PBP, P2BP2 and FBF anion radicals. This series was selected for display in Figure 3 because they were all carried out with the same basis set/functional pairing (B3LYP/6–31Gd), and they are representative for a series in which the extent of π-conjugation is varied. The corresponding figures for the other seven anion radicals are in the DFT-SI section. While the energy of the lowest lying absorption bands were not well predicted by TD-DFT, the second lowest energy experimental absorption band was well correlated with the first TD-DFT excited state (ES1) with an average absolute difference of less than 4%. In all cases, the oscillator strength for the third transition (ES3) was much larger than that of ES1 which matches the pattern for the experimental spectra.

**Figure 3:**
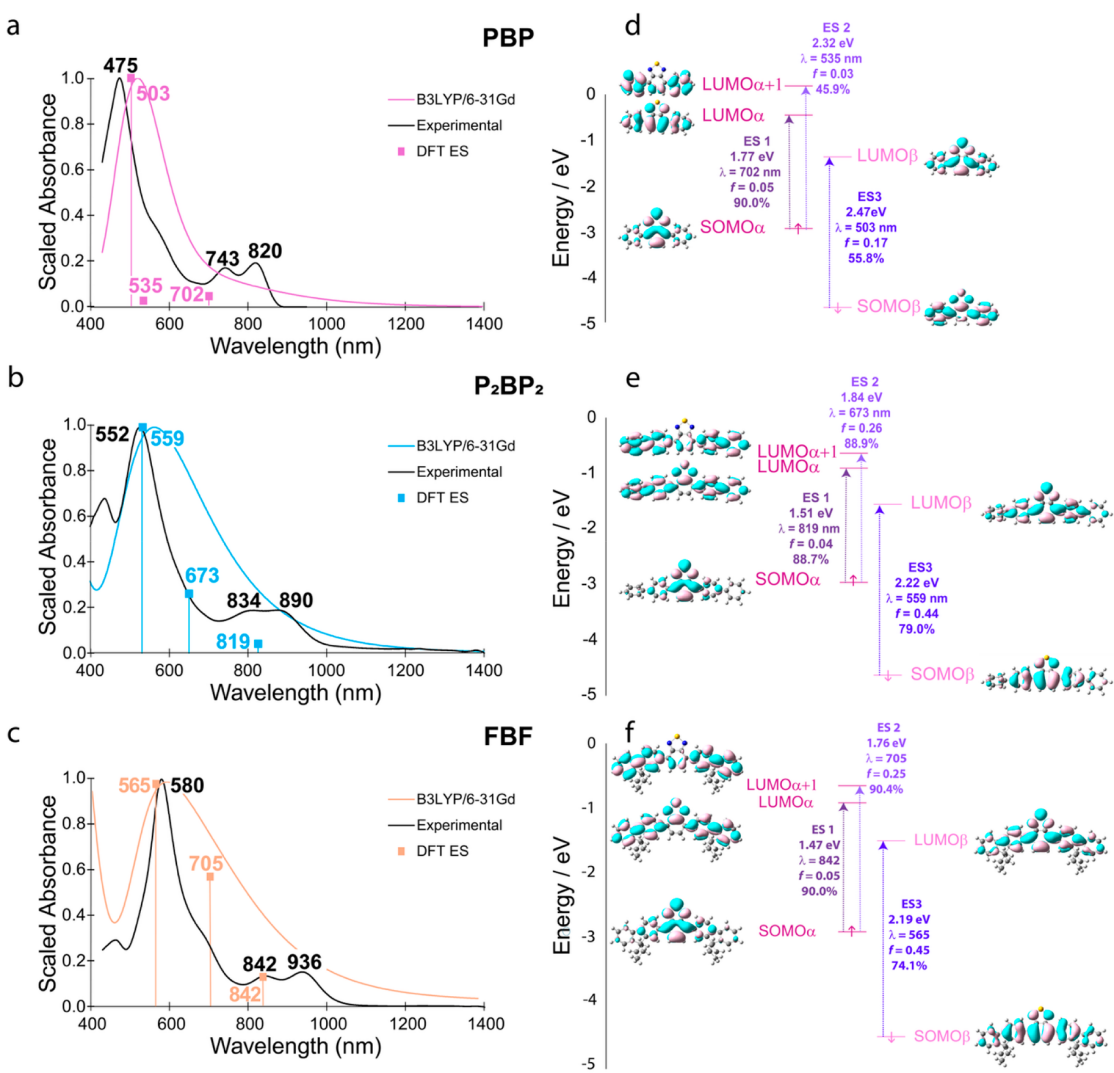
(a–c) Overlay of the experimental and calculated UV–visible absorption spectra of PBP^–•^, P_2_BP_2_^–•^ and FBF^–•^. The wavelengths for the calculated spectra are shown, and the lengths of the lines represent the relative oscillator strengths of the calculated transitions. The calculated spectra show the three lowest energy transitions. (d–f) Diagram showing the corresponding transitions for the first three lowest energy absorptions. Density plots of the frontier molecular orbitals that dominate each transition are provided to indicate how the change in electron density that accompanies the transition. For each transition, the energy, wavelength, oscillator strength and % contribution of the orbitals shown are given.

For most of the structures, the lowest lying excited state (ES1) was primarily generated from the SOMO_α_ to LUMO_α_ transition, with the exceptions being TBT, T2BT2, TBpyT, and EBE which were generated from the SOMO_β_ to LUMO_β_. For PBP, ES1 was 90% due to the SOMO_α_ to LUMO_α_ transition, and the electron density shifts from being primarily located on the BTD acceptor moiety to evenly distributed across all three arylene rings, suggesting that it is a local type of excitation (LE). By contrast, the ES2 transition exhibits a larger shift of electron density away from the BTD moiety to the arylene rings, consistent with a charge-transfer (CT) type of excitation. As was the case with the ES1 transition, ES3 shows a local excitation with only minor changes in the electron density distribution. Notably, the transition labeled ES3, which has the largest oscillator strength and maps closely to the strongest experimental absorption band, arises from the SOMO_β_ to LUMO_β_ transition. This transition appears to correspond to the HOMO–LUMO transition for the neutral DAD chromophore.

Importantly, the TD-DFT calculations show that in most cases the lowest transition (ES1), which presumably corresponds to the lowest doublet excited state of the anion radical, arises from the SOMO_α_ to LUMO_α_ transition. In a one-electron orbital view, this transition corresponds to an excitation from the LUMO to LUMO+1 in the neutral oligomer. Consequently, the energy of the lowest excited state (ES1) in the anion radical decreases as the energy spacing between the lowest virtual (unoccupied) orbitals in the neutral oligomer narrows. Thus, as the conjugation length (π-delocalization of the oligomer increases, the density of low-energy virtual orbitals rises, reducing their spacing and lowering the energy of the lowest transition. From this we conclude that molecules with a more localized π-electron distribution may be expected to have higher energy doublet excited states in their reduced form.

### Femtosecond Transient Absorption: Doublet Excited State Absorption and Lifetimes

The visible and near-IR excited state absorption spectra of the series of DAD anion radicals (DAD^–•^) were measured by pump–probe spectroscopy using 100 fs pulsed excitation. In every case, excitation produces an excited state that displays strong positive and negative difference absorption (ΔA) throughout the visible and near-IR spectral regions, and decay times vary from 565 to 3 ps across the series. Figure 4 illustrates the full set of time-resolved difference absorption spectra for TBT, T2BT2 and T3BT3, along with the experimental kinetics and single exponential decay fits for selected wavelengths. The data for all the other DAD^–•^ species are provided in the Supporting Information (Figures S44–S46). The fs-TA data shown in Figure 4 reveal several important characteristics. First, the visible region spectra exhibit positive and negative absorption features; the negative bands mainly correspond with the bleach of the ground state DAD^–•^ absorption bands. Weak negative absorption in the near-IR at ∼ 900 nm for TBT (Figure 4a) corresponds to stimulated emission. Taken together, these observations support assignment of the transients to the lowest doublet excited state of the anion radicals, e.g. ^2^DAD^–•^*. Analysis of the transient absorption dynamics at multiple wavelengths (and with global analysis) showed that all the ^2^DAD^–•^* display single exponential decay kinetics which correspond to the doublet excited state lifetime (τ). As seen in Figure 4, for the series consisting of TBT, T2BT2 and T3BT3, τ decreases by almost a factor of 100 from 245 ps for TBT to 2.9 ps for T3BT3. Kinetic analysis of the data for the other members of the series reveals similar trends and the decay lifetimes obtained from analysis of the time-resolved spectra data for all the ^2^DAD^–•^* are listed in Table 1.

**Figure 4:**
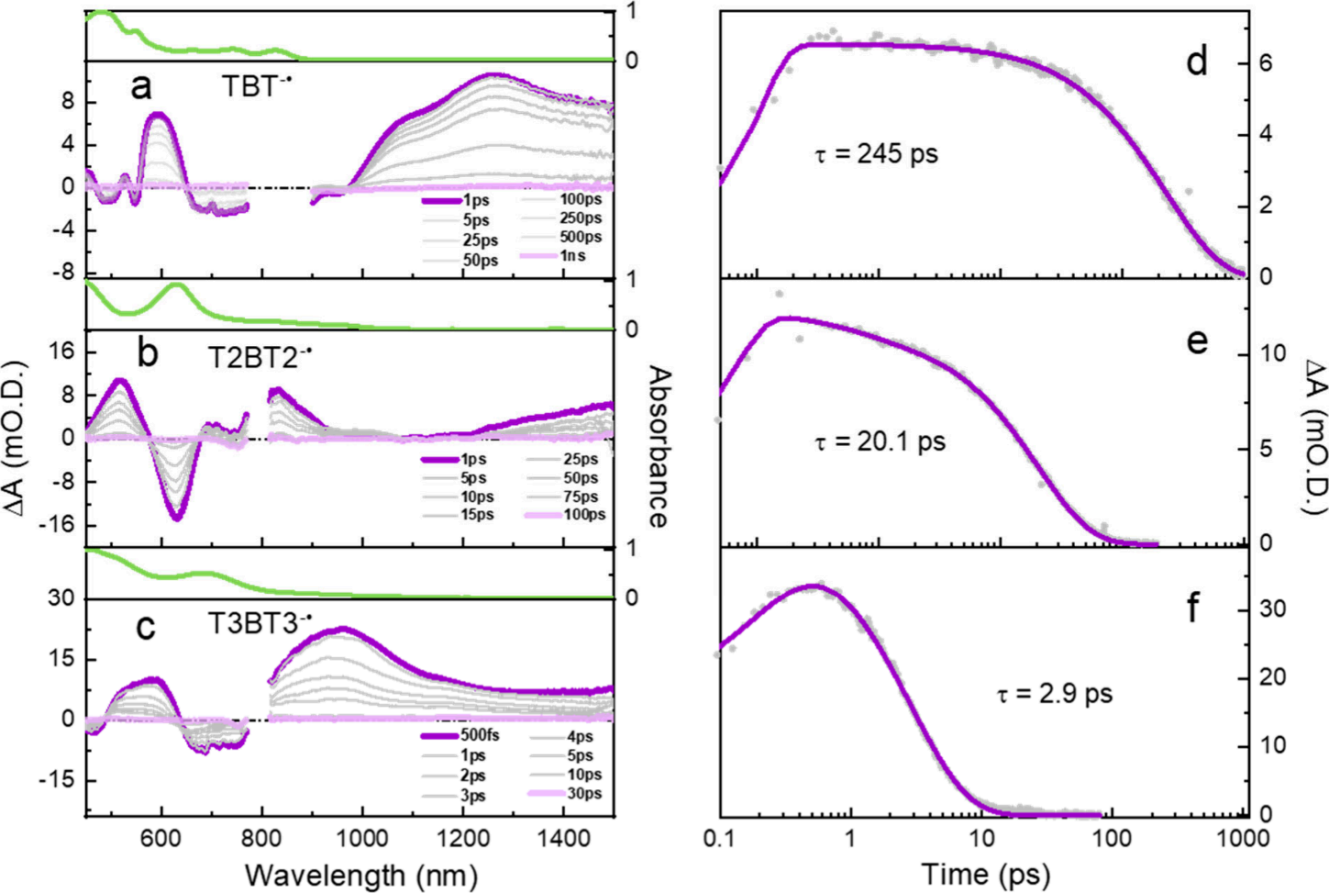
Femtosecond transient absorption spectra (left) and decay kinetics (right) of DAD^–•^ in DMF solution. Delay times for spectra are shown in the legends and lifetimes obtained from single exponential fits at 591 nm (TBT^–•^), 517 nm (T2BT2^–•^), and 977 nm (T3BT3^–•^). Ground state absorption spectra of the DAD^–•^ are shown in the plots above each of the sets of transient absorption spectra. (a,d) TBT^–•^, λ_ex_ = 825 nm. (b,e) T2BT2^–•^, λ_ex_ = 750 nm. (c,f) T3BT3^–•^, λ_ex_ = 750 nm.

**Table 1:** Electrochemical and Photophysical Parameters

DAD	E_00_/cm^–1^ ^a^	E_1/2red_/V^b^	E(^–*^/^0^)/V^c^	τ/ps^d^	*k*_nr_/10^9^ s^–1^	log *k*_nr_ ^e^
PBpyP	13,280	–1.17	–2.79	349	2.88	9.46
TBpyT	12,900	–1.03	–2.63	565	1.78	9.25
PBP	12,225	–1.50	–2.98	118	8.51	9.93
TBT	12,105	–1.32	–2.81	245	4.07	9.61
EBE	12,035	–1.44	–2.89	171	5.89	9.77
P2BP2	11,160	–1.41	–2.72	6	166	11.22
PyBPy	11,075	–1.22	–2.61	46	21.9	10.34
FBF	10,660	–1.45	–2.73	10	100	11.0
T2BT2	10,560	–1.24	–2.56	20	50.1	10.70
T3BT3	10,010	–1.17	–2.43	3	331	11.52

a Calculated from the maximum of the longest wavelength absorption band in DMF.

b Reduction potential from cyclic voltammetry in DMF solution relative to SCE. Potentials converted from Fc/Fc^+^ scale by the expression E = E­(Fc/Fc^+^) + 0.45 V, ref 40.

c Oxidation potential of anion radical excited state, E­(^−*/0^) = E_red_(DAD) – E*, relative to SCE.

d Lifetime of anion radical excited state from fs transient absorption.

e *k*_nr_ = 1/τ, *k*_nr_ is the nonradiative decay rate of the anion radical excited state.

The striking feature that emerges from comparison of the decay data for the series of DAD^–•^ excited states is that they display a wide range of lifetimes. Analysis of these data naturally raises the question of what underlies the large variation in the lifetimes. The fluorescence quantum yields (ϕ_fl_) for six of the DAD^–•^ (Tables S2 and S3) were measured, and in most cases, ϕ_fl_ < 0.001. Thus, we conclude that the excited state decay occurs almost exclusively by nonradiative pathways, and therefore the nonradiative decay rate, *k*_nr_, can be calculated by the equation *k*_nr_ = 1/τ, where τ is the decay lifetime determined by fs-TA. Table 1 lists the nonradiative decay rates (and log *k*_nr_ values) for the series. Note that the DAD^–•^ entries in the table are listed in order of decreasing energy (E_00_) for the lowest energy absorption feature, and it is seen that the *k*_nr_ values generally increase with decreasing E_00_ (top to bottom in Table 1). This is a strong indication that the nonradiative decay rates are correlated with E_00_ according to the energy gap law.

### Energy Gap Law Analysis

The energy gap law for nonradiative decay was formulated by Englman and Jortner, and Siebrand demonstrated its application to nonradiative decay of the triplet excited states of a series of aromatic hydrocarbons.^37,41^ Four decades ago, Meyer and co-workers applied the energy gap law to analyze the nonradiative decay of metal-to-ligand charge transfer (MLCT) excited states in a range of transition metal complexes.^38,42^ More recently, Caram and co-workers extended the energy gap law correlation to analysis of decay rates of a series of cyanine dyes that fluoresce in the visible and near-infrared regions.^43^ The energy gap law is derived from a quantum analysis of the electronic and vibrational coupling of the excited and ground state potential surfaces, and the effect derives from an increase in the vibronic coupling between the states as the energy gap between them decreases.^37,38,41^

In all cases that have been previously reported, there have been qualitative linear correlations between the excited state energy (E_00_) and the logarithm of the nonradiative decay rate (ln *k*_nr_).^37,38,43–45^ To date, although there have been various reports of the lifetimes of doublet excited states of odd electron organic species (radicals, anion radicals and cation radicals),^18,22,24,46–56^ there has never been a study that provided a correlation of the lifetimes with the doublet excited state energy.

Following the treatment outlined by Siebrand,^37^ and later by Caspar and Meyer,^38,42^ the quantitative mathematical formulation of the relationship between *k*_nr_ and emission energy (E_0_) is given by eq S1a in the Supporting Information. Under the assumption that all terms but the second in eq S1a do not vary with E_00_, the expression can be simplified to eqs 1a and 1b,

1a $$\ln\;k_{nr}=a-\left(\frac{\gamma_{o}}{\hbar \omega_{m}}\right)E_{00}$$

1b $$\gamma_{o}=\ln\left(\frac{E_{00}}{\hbar \omega_{m}S_{m}}\right)-1$$

where ℏω_m_ is the average frequency of the vibrational modes coupled to the doublet excited state decay, S_m_ is the Huang–Rhys factor (the electron-vibrational coupling constant), and a is a constant that depends on the ground-excited state electronic coupling, in addition to other parameters. Equations 1a and 1b are the mathematical expression of the energy gap law, and from eq 1a it predicts a linear dependence of ln *k*_nr_ on excited state energy, with slope −γ_o_/ℏω_m_. Figure 5a illustrates a plot of ln *k*_nr_ as a function of E_00_ for decay of the doublet excited states for the series of ten DAD^–•^. As can be seen, the data display a reasonable linear correlation (Pearson’s r = −0.913) over an energy range from 10,000 – 13,200 cm^–1^ with a slope of −1.6 × 10^–3^ cm.

**Figure 5:**
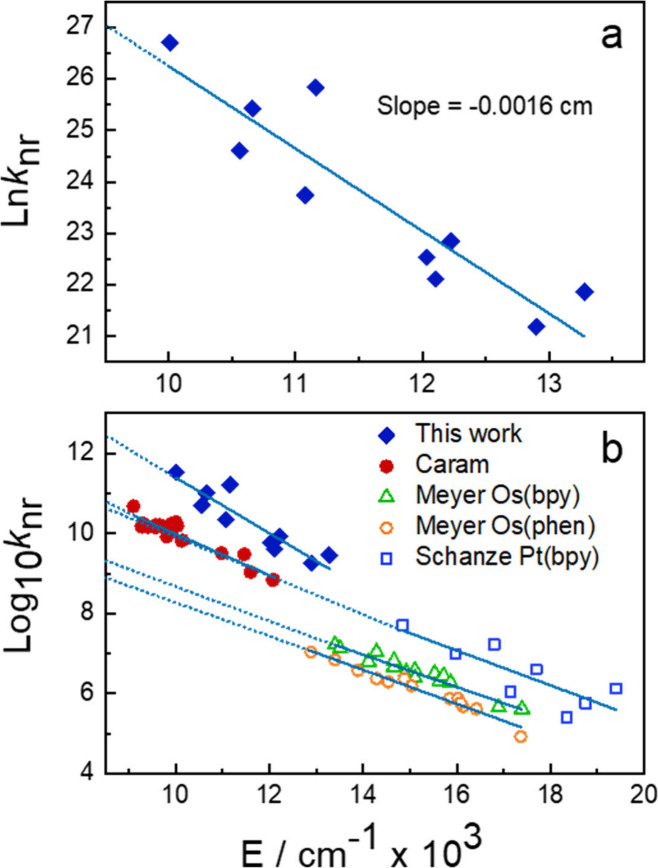
Energy gap law plots for excited state decay. (a) Energy gap law plot for nonradiative decay rates of ^2^DAD^–•^*. Slope is −0.0016 cm. (b) Comparison of energy gap law plots for ^2^DAD^–•^* compared to excited state decay from previous studies. Data for series of Os­(bpy) and Os­(phen) complexes from ref 38, for Pt­(bpy) complexes from ref 44, and for cyanine dyes from ref 43.

The energy gap law also provides the framework to relate the energy gap law correlation as the slope γ_o_/ℏω_m_ to the fluorescence spectra for the species.^44,57^ By fitting the shape of the emission spectra (via eq S3), it is possible to estimate values for E_00_, ℏω_m_ and S_m_, which then allows estimation of γ_o_/ℏω_m_ via eq 1b. Fits of the fluorescence spectra for three of the DAD^–•^ which display well-resolved emission bands with vibronic structure are shown in Figures S47–S49 in the Supporting Information, and analysis of the spectral fitting parameters gives an average of γ_o_/ℏω_m_ = 1.3 × 10^–3^ cm (Table S4), in good agreement with the slope of the energy gap law correlation in Figure 5a. Interestingly, the spectral fits suggest that the dominant high frequency vibronic mode is in the range 1000–1150 cm^–1^, which is less than typical values seen for the coupled vibrational modes for excited states of aromatic hydrocarbons and MLCT excited states, which are in the range 1300 cm^–1^. We attribute the lower frequency to the localization of electron density on the heterocyclic acceptor unit in the anion radicals, which leads to dominant C–N and N–S vibrational modes which are at relatively lower frequency.^58^

It is also of interest to compare the energy gap law correlation observed for the ^2^DAD^–•^* studied herein and correlations reported in previous studies of excited state decay. Figure 5b compares the correlations reported in studies of three series of metal complexes based on Os­(II) and Pt­(II) polypyridine complexes,^38,42,44^ along with results from Caram and co-workers on near-infrared fluorescent cyanine dyes.^43^ The average slope of the metal complex data is 9.7 × 10^–4^ cm, which is ∼ 40% less than the slope of the correlation for nonradiative decay of the DAD^–•^ excited states. This difference is likely due to the lower frequency modes that dominate the decay of the DAD^–•^ excited states. Also noteworthy is the fact that the correlation for the DAD^–•^ excited states lies above the one for the cyanine dyes which have similar excited state energies. This difference indicates that there is stronger excited state-ground state electronic coupling in the doublet excited states compared to singlet excited state decay in the cyanine dyes.

Taken together, the results show that the lifetimes of the anion radical doublet excited states are correlated to the excited state energy via the energy gap law. Given that the lowest energy transition in these species correlates with the SOMO-LUMO transition, which is derived from virtual orbitals in the corresponding neutrals that are relatively closely spaced in energy, it is inevitable that these species will have relatively low excited state energies and consequently relatively short lifetimes. As noted above, anion-radical chromophores with less π-conjugation are expected to have higher energy doublet excited states, and consequently relatively longer lifetimes.

### DAD Anion Serves As a Competent ConPET Photocatalyst

Since the photophysical studies outlined above demonstrate that some DAD anion radicals have sufficiently long lifetimes to undergo efficient bimolecular photoinduced ET with acceptors, we set out to demonstrate that a DAD chromophore can function as a competent ConPET catalyst. The photophysical studies reveal that *TBpyT^–•^ has a lifetime of 565 ps and an excited state oxidation potential of −2.63 V (vs SCE, Table 1). Accordingly, the relatively long lifetime of *TBpyT^–•^ should enable efficient bimolecular electron-transfer quenching in the presence of moderate concentrations (>20 mM, Figure 1) of electron acceptors with reduction potentials more positive than −2.5 V. Thus, a demonstration reaction was carried out using TBpyT as the photocatalyst and 4-bromoacetophenone (**1**) as a substrate. Cyclic voltammetry of **1** in DMF solution reveals an irreversible cathodic wave at −1.86 V (Figure S53a), consistent with the literature.^59^ Previous studies have shown that **1** undergoes reductive debromination to acetophenone (**2**) in the presence of a photocatalyst and a sacrificial reducing agent.^60^

First, we demonstrated that *TBpyT^–•^ is quenched effectively by **1**. As shown in Figure 6, addition of **1** at moderate concentrations to solutions of TBpyT^–•^ reduces both the excited state lifetime and the fluorescence intensity. Stern–Volmer (SV) plots were constructed with the lifetime and intensity quenching, and they are linear, with the slopes affording SV quenching constants, K_SV_ ∼ 10.4 and 15.0 M^–1^, respectively (Figure 6c). Using the relation *k*_q_ = K_SV_/τ_0_, and the unquenched lifetime τ_0_ of *TBpyT^–•^, we determined a bimolecular quenching rate constant of 1.9 × 10^10^ M^–1^s^–1^ from the dynamic quenching SV analysis. This rate constant is consistent with moderately exothermic (ΔG ∼ −0.8 eV) bimolecular ET reaction between *TBpyT^–•^ and **1**.

**Figure 6:**
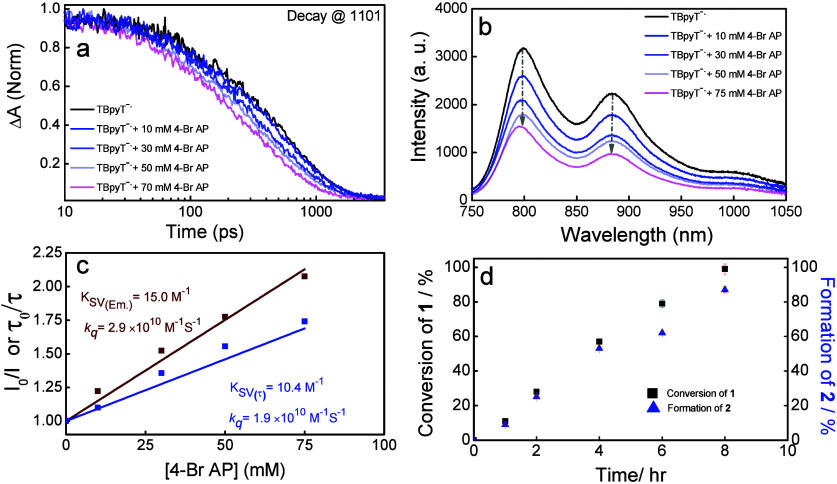
(a) Transient absorption lifetime quenching of TBpyT^–•^ (0.1 mM) by 4-bromoacetophenone (**1**) monitored at 1101 nm in deoxygenated DMF; excitation wavelength 550 nm. (b) Steady-state fluorescence intensity quenching of TBpyT^–•^ (0.1 mM) with **1** in deoxygenated DMF solution; excitation wavelength 725 nm. (c) Stern–Volmer plots for the transient absorption lifetime quenching (blue squares) and steady-state emission quenching (maroon squares) of TBpyT^–•^ by **1**. (d) Progress of reaction for conversion of **1** to **2**. Conditions: **1** (1 mM), TBpyT (0.35 mM), triethylamine (0.2 M), LED sources at 470 nm (250 mW) and 525 nm (110 mW). Analysis by HPLC and product identity confirmed by GC-MS.

Next, a degassed solution of **1** (1 mM), TBpyT (0.35 mM) and triethylamine (0.2 M) was subjected to photolysis with a pair of LED sources (470 and 525 nm). The reaction was monitored over a course of 8 h by HPLC which gives well resolved peaks for **1**, **2** and naphthalene. Here is it seen that the photocatalyzed reduction of **1** occurs to cleanly afford debromination product **2** and the reaction is complete in ∼ 8 h (Figure 6d). The identity of the product **2** as acetophenone was confirmed by GC-MS analysis of the reaction mixture at 4 h (Figure S52). This experiment demonstrates that TBpyT can serve as a competent photocatalyst for the reductive dehalogenation of 4-bromoacetophenone.^31^

Scheme 1 provides a summary of the proposed mechanism of the photocatalyzed reductive debromination reaction. The singlet excited state of TBpyT is reduced by triethylamine to afford the anion, TBpyT^–•^. The anion is then promoted to its excited state, *TBpyT^–•^ which is quenched by bimolecular ET with the substrate, which then goes on to product formation. TbpyT is regenerated and available to re-enter the photocatalytic cycle. It is important to note that the ground state TBpyT^–•^ has insufficient potential to accomplish the direct reduction of **1**; the reaction must involve a ConPET mechanism with *TBpyT^–•^ to generate sufficient reducing power.

**Scheme 1:**
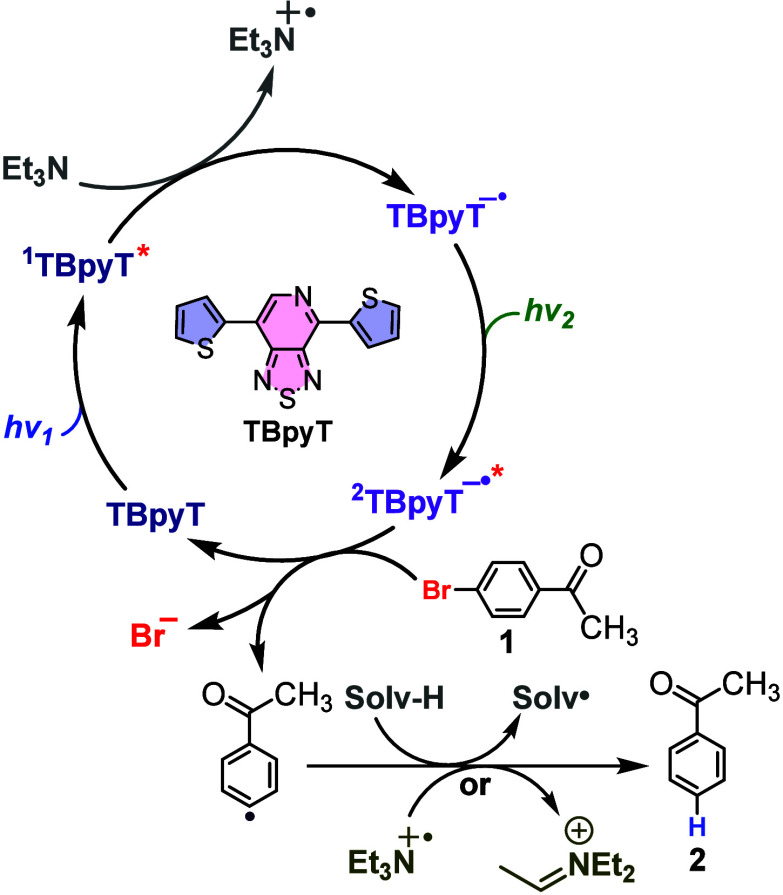
ConPET Reaction Mechanism

We also examined whether a DAD anion radical with a shorter lifetime could serve as a photocatalyst. For this study we selected PBP^–•^ which has a lifetime of 118 ps, which is 4.8-fold shorter than TBpyT^–•^. PBP has a more negative reduction potential than TBpyT (Table 1), and because of this PBP^–•^ undergoes a slow thermal reaction with 4-bromoacetophenone. Thus, we compared the SV quenching and photocatalytic reaction of TBpyT and PBP with 4-bromoanisole, which features a more negative reduction potential at E_p_ ∼ −2.77 V (Figure S53b), consistent with the literature.^61^ The results of this study are summarized in the Supporting Information (see Figures S54–S57). Key findings are that the excited state quenching of both TBpyT^–•^and PBP^–•^ by 4-bromoanisole is less efficient due to its more negative reduction potential. Additionally, the photocatalytic debromination reaction of 4-bromoanisole proceeded to only 20% at 18 h of photolysis with TBpyT, and under the same conditions no reaction was observed with PBP. Given that *PBP^–•^ is a stronger reductant compared to *TBpyT^–•^ (Table 1), we conclude that its lack of photocatalytic reactivity is associated with its shorter doublet excited state lifetime which limits the efficiency of bimolecular photoinduced ET.

## Summary and Conclusions

This work presents a comprehensive investigation of the photophysical properties of anion radicals derived from a series of donor–acceptor–donor (DAD) π-conjugated oligomers and establishes a quantitative correlation between their doublet excited state lifetimes and excited state energies via the energy gap law. Ten DAD-based chromophores were synthesized and characterized using electrochemical methods, steady-state absorption and emission spectroscopy, and femtosecond transient absorption spectroscopy. Upon chemical reduction, the DAD anion radicals exhibit broad absorption bands extending into the near-IR region and weak fluorescence with small Stokes shifts. TD-DFT calculations reveal that the lowest excited state (ES1) of the anion radicals originates primarily from the SOMOα → LUMOα transition, which corresponds to the LUMO → LUMO+1 excitation in the neutral oligomer. This orbital mapping explains the observed trend that increasing conjugation length reduces the energy spacing between virtual orbitals, thereby lowering the excited state energy.

Femtosecond transient absorption measurements show that the doublet excited state lifetimes vary dramatically across the series, ranging from 565 ps for TBpyT to 3 ps for T3BT3. Analysis of the decay kinetics and fluorescence quantum yields indicates that nonradiative decay dominates, and the calculated nonradiative rate constants (*k*_nr_) exhibit a strong correlation with the excited state energy (E_00_), consistent with the energy gap law. This represents the first demonstration of an energy gap law correlation for ion-radical excited states. Comparison with previous studies on metal-to-ligand charge transfer (MLCT) excited states and cyanine dye singlet excited states reveals that the slope of the correlation for DAD anion radicals is steeper, likely due to lower-frequency vibrational modes associated with the benzothiadiazole heterocyclic acceptor units.

The practical significance of these findings was demonstrated by employing TBpyT^–•^, an anion radical with a relatively long-lived excited state (τ ≈ 565 ps), as a photosensitizer in a consecutive photoinduced electron transfer photocatalytic reaction. In the presence of a sacrificial reducing agent, TBpyT^–•^ catalyzed the reductive debromination of 4-bromoacetophenone under visible light irradiation, validating the potential of DAD-based anion radicals as competent photocatalysts. This study provides a mechanistic framework for designing ion-radical chromophores with tailored excited state lifetimes by controlling conjugation length and electronic structure. Future work will focus on expanding the structural diversity of DAD systems, optimizing their excited state properties, and exploring their application in a broader range of photoredox transformations.

## Experimental Section

### Synthesis and Characterization

Details of the synthesis and spectroscopic characterization (NMR, mass spectrometry) of all compounds are provided in the Supporting Information section.

### General Methods

Cyclic voltammetry measurements were carried out in dichloromethane (DCM) at scan rate 100 mV-s^–1^ using a CHI-660E electrochemical workstation and the measurements were conducted at room temperature. UV–visible absorption spectra were recorded using the Shimadzu UV 2600 spectrophotometer and V-770 UV–visible/NIR spectrophotometer. Steady-state visible and near-IR photoluminescence measurements were performed on an Edinburg FLS1000 and Horiba Scientific Duetta spectrophotometer. The fluorescence lifetime data of neutral compounds were obtained on a Pico Quant FT300 time correlated single photon counting (TCSPC) instrument. Excitation was generated by the second harmonic output of a mode-locked Ti:sapphire oscillator (Coherent Chameleon) that included a pulse-picker to reduce the repetition rate.

### Generation of Radical Anions

Dry solvents (THF and DMF) were obtained from an MBraun MB-SPS-800 solvent purification system. After a 30 min nitrogen purge THF and DMF solvents were transferring into an MBraun inert atmosphere glovebox, where the reduction reaction was carried out under the inert atmosphere. A mixture of sodium metal (23 mg, 1 equiv) and acenaphthylene (152 mg, 1 equiv) was prepared in a 15 mL vial, followed by addition of 5 mL of dry THF. The reaction mixture was stirred in dark inside the glovebox (covered with aluminum foil) overnight to generate a 0.1 M solution of acenaphthylene radical anion. The resulting reaction mixture solution was filtered through a syringe, and the filtered solution was diluted to 10 mM by mixing 100 μL of the 0.1 M acenaphthylene radical anion solution in THF with 900 μL of DMF. To prepare the sample for fs-TA measurement, 200 μL of 1 mM DAD neutral chromophore in DMF, 100 μL of acenaphthylene radical anion solution, and 200 μL of DMF were mixed in a 2 mm path length quartz cuvette. The cuvette was protected from the light by wrapping it with aluminum foil and sealed with a rubber septum and parafilm before removing it from the glovebox. The samples were carefully handled free from oxygen and light throughout the measurements.

### Femtosecond Transient Absorption

Femtosecond transient absorption spectroscopy was carried out using a system consisting of a Coherent Astrella Ti:sapphire (100 fs,1 kHz repetition, 5 W, 800 nm) source coupled with an Opera Solo optical parametric amplifier (OPA) and an Ultrafast Systems Helios transient absorption spectrometer. The 80% of the power in the fundamental output the Astrella was directed through the OPA, which provided wavelength tunable excitation over the range of 550–825 nm. The excitation beam was then directed into a Helios Fire (Ultrafast) automated femtosecond transient absorption spectrometer where it was passed through a chopper, depolarizer and neutral density filter to bring the power to 100 μW (100 nJ-pulse^–1^) prior to incidence on the stirred sample contained in a 2 mm path cell (O.D. ≈ 0.4–0.5). The residual from the Astrella (∼1 W) was passed through a digitally controlled delay stage with a maximum range of 8 ns, and then the beam was focused into a sapphire crystal to generate broadband probe light ranging from 420–700 nm. The near-IR probe was generated using a proprietary crystal (Ultrafast Systems) producing a broadband probe spanning from 820–1600 nm. The excitation (pump) and broadband probe beams overlap at the sample position with their respective electronic polarizations at the magic angle. The output signal with and without pumping (at several time delays) was detected using a fiber-coupled alignment-free spectrometer with a 1024-pixel CMOS sensor. Chirp, time-zero, and solvent response corrections as well as spectral selection and single-wavelength decay fits were carried out using the software supplied by Ultrafast Systems. OriginLab Corporation (version 9.55) data analysis software was used to plot the spectra.

### Photocatalysis Study

In an aluminum foil-covered, screw capped vial, 250 μL of a 10 mM solution of compound 4-bromoacetophenone, 500 μL of a solution of 1.0 M triethylamine (Et_3_N), and 875 μL of a 1.0 mM solution of TBpyT (all the stock solutions were prepared in DMF) and 875 μL of DMF were mixed inside a N_2_ atmosphere glovebox. Then, 1.2 mL of this solution was transferred to an NMR tube, which was sealed with parafilm and Teflon tape and covered with aluminum foil, before being removed from the glovebox. The NMR tube was then irradiated simultaneously with a 470 nm blue (fwhm = 25 nm, 250 mW) and a 525 nm green LED (fwhm = 25 nm, 110 mW) for various time intervals (LEDs sourced from ThorLabs).

After a specific time interval of light exposure, 1080 μL of the reaction mixture was spiked with 75.6 μL of a 0.1 M naphthalene (Std.) and 44.4 μL of DMF to produce a total volume of 1.20 mL of sample for HPLC analysis. A 1.00 mL aliquot of this sample was filtered through a 0.45 μm PTFE syringe filter and collected in an amber glass HPLC vial, which was then submitted for HPLC injection. The peak areas from the HPLC analysis were used as described in the Supporting Information to determine the concentrations of the reactant and products in the solutions.

HPLC analyses were conducted using a Shimadzu Prominence LC-20AT system fitted with a reversed-phase C18 column (Restek; 250 × 4.6 mm, 5 μm particle size), with the column temperature held at 25 °C. The mobile phase comprised methanol and water and was delivered under gradient elution conditions at a flow rate of 1.0 mL/min. Detection was carried out by UV absorbance at either 242 or 272 nm, depending on the analyte. The typical injection volume was 20 μL. Selected samples from photocatalysis were also analyzed by gas chromatography–mass spectrometry using a Shimadzu instrument (GC model 2030 and GCMS-QP2050). The column temperature and flow rate was 30 °C and 0.68 mL-min^–1^.

## Supplementary Material





## Notes

Complete synthesis details and compound characterization by ^1^H, ^13^C NMR and high-resolution mass spectrometery, electrochemical data, absorption and fluorescence data, fluorescence quantum yields, emission spectral fitting, and photocatalysis results and analysis (PDF)
Complete description of the methods used for DFT and TDDFT calculations, along with the results for the complete set of 10 anion radicals (PDF)
