# Supplementary material for: From Fundamental Photophysics to Photocatalysis: Energy Gap Law Analysis of Anion Radical Excited States
Source: ACS Cent Sci. 2026 Jun 15;12(6):856–66. doi: 10.1021/acscentsci.6c00092 (PMC13306596; doi:10.1021/acscentsci.6c00092)
Supplement: Supplementary file 1 [file oc6c00092_si_001.pdf]

Supporting Information

From Fundamental Photophysics to Photocatalysis: Energy Gap Law Analysis of Anion Radical Excited States

Naresh Duvva†, Tanjila Islam†, Silvano R. Valandro†, Habtom B. Gobeze†, Abul Mansur Muhammed Fahim†, Xiaodan Wang†, Trang Le†, Aimée L. Tomlinson‡* and Kirk S. Schanze†*

†Department of Chemistry, the University of Texas at San Antonio, One UTSA Circle, San Antonio, Texas 78249, United States.

‡Department of Chemistry and Biochemistry, University of North Georgia, Dahlonega, Georgia, 30597, United States.

*Corresponding author E-mails: kirk.schanze@utsa.edu, aimee.tomlinson@ung.edu

Table of Contents

**Topic**	**Pages**
Synthesis of Thiophene Based DAD Compounds	S-3
Compound Characterization	S-7
Synthesis of Phenyl, Biphenyl and Fluorene based DAD Compounds	S-15
Compound Characterization	S-18
Synthesis of Thiadiazol-Pyridine Based DAD Compounds	S-27
Compound Characterization	S-28
Absorption and Fluorescence of Neutrals and Anion Radicals	S-32
Electrochemistry Results	S-35
Femtosecond Transient Absorption Results	S-37
Photophysical Parameters for Neutrals and Anion Radicals	S40
Energy Gap Law and Fitting of Fluorescence Data	S-42
Photocatalysis Experiments: TBpyT with 4-Bromoacetophenone	S46
Electrochemistry of 4-Bromoacetophenone and 4-Bromoanisole	S50
Quenching and Photocatalysis: TBpyT and PBP with 4-bromoanisole	S51
References	S-55

Experimental

General Methods, Materials, and Characterization. All reactions were carried out under dry nitrogen atmosphere using dry solvents which were obtained from MBraun MB-SPS-800 solvent purification system. Solvents for flash column were obtained from commercial sources from fisher scientific and used without any further purification. Compounds 4,7-dibromo-2,1,3-benzothiadiazole, 4,7-dibromo-pyridal[2,1,3]thiadiazole, 2-(4-hexylthiophen-2-yl)-4,4,5,5-tetramethyl-1,3,2-dioxaborolane, 2-(tributylstannyl) thiophene, N-bromosuccinimide (NBS), 2-(tributylstannyl)thiophene, (4-hexylphenyl)boronic acid, 1-bromo-4-iodobenzene and 4-pyridinylboronic acid were purchased from Sigma-Aldrich, TCI America and Fisher Scientific and used as received. All other reagents were obtained from Sigma-Aldrich, Fisher Scientific, Acros and TCI America. Detailed synthetic procedures and compound characterization data are summarized below (in Scheme S1, S2 and S3), and all the synthesized compounds were characterized by ^{1}H-NMR and ^{13}C {^{1}H} NMR and HRMS (ESI) mass spectrometry. All chemical shifts are quoted as ppm relative to tetramethylsilane.

General Suzuki Coupling Procedure for Synthesis of DAD Compounds. The reactions were performed using 150 – 200 mg of the starting compound under standard conditions. In a 100 mL Schlenk flask, 1 equivalent of 4,7-Dibromo-2,1,3-benzothiadiazole (or) 4,7-dibromo-pyridal[2,1,3]thiadiazole, 2.4 equivalents of aryl boronic acid/boronic acid pinacol ester derivative and 4 equivalents of sodium carbonate were added to 20 mL of dioxane, 8 mL of toluene and 5 mL of H_2O. Four cycles of freeze-pump-thaw were performed to degas the solution before adding 12 mol% of $Pd(PPh_3)_4$ under N_2 protection and the solution was refluxed for 18 hours. After 18 hours, the reaction was quenched by adding aq. NaCl. Then the mixture was rinsed with water and brine

and extracted three times with DCM. The organic layer was dried over Na_2SO_4 and purified by flash column chromatography with hexanes and ethyl acetate.

General Stille Coupling Procedure for Synthesis of DAD Compounds. The reactions were performed using 150 - 200 mg of the starting compound under standard conditions. In a 100 mL Schlenk flask, 1 equivalent of dibromo derivative of 4,7-bis(5-bromo-4-hexylthiophen-2-yl)benzo[c][1,2,5]thiadiazole (or) 4,7-dibromo-pyridal[2,1,3]thiadiazole and 2.3 equivalent of 2-(tributylstannyl) thiophene were added to DMF (50 mL) under argon and degassed for 45 min then 12 mol% of $Pd(PPh_3)_4$ was added under argon and again degassed for 30 min. The reaction mixture was stirred at 100°C for 16 hours, then reaction mixture was cooled to room temperature and organic layer was separated. The organic layer was washed with water, sodium bicarbonate solution and dried over anhydrous Na_2SO_4. The crude product obtained after evaporation of solvent was separated using flash column chromatography with gradient of hexane and ethyl acetate. (*Important note*: After the column purification, the final compound was washed with aq. 2 M NaOH to remove tin impurities).

General Procedure for Bromination of Thiophene Oligomers. The reactions were performed using 150 - 200 mg of the starting compound under standard conditions. In a 100 mL two neck round bottom flask, 1 equivalent of TBT or T2BT2 thiophene oligomer was dissolved in 50 mL of N, N-dimethylformamide (DMF) under argon and cooled in an ice bath. Then NBS (2.3 eq.) was added to the reaction mixture at 0 °C and stirred for 1 hour. The reaction mixture was further stirred at room temperature overnight. Then the solvent evaporated under rotary evaporator and additional DMF solution was quenched by ice water. The precipitate was collected by filtration and washed with methanol. In general, the solid thus obtained was pure. If necessary, it was further

purified by recrystallization in DCM/methanol or flash column chromatography on silica using gradient of hexanes and ethyl acetate.

Scheme S1. Synthesis of Thiophene Based DAD Compounds

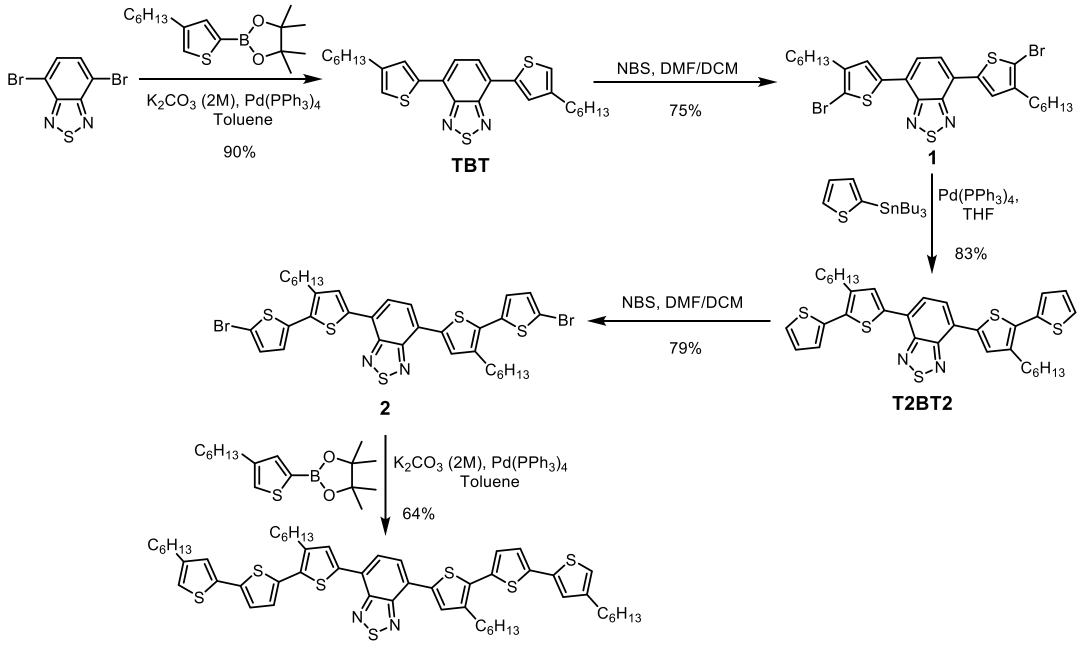

Synthesis of 4,7-bis(4-hexylthiophen-2-yl)benzo[c][1,2,5]thiadiazole (TBT). Following the general procedure for Suzuki coupling, TBT was synthesized by reacting 4,7-dibromobenzo[c][1,2,5]thiadiazole (200 mg, 0.68 mmol) with 2-(4-hexylthiophen-2-yl)-4,4,5,5-tetramethyl-1,3,2-dioxaborolane (480 mg, 1.63 mmol). The product was obtained as a red crystalline solid (287 mg, 0.612 mmol, 90% yield). ^{1}H NMR (500 MHz, $CDCl_3$) δ 8.00 (s, 2H), 7.85 (s, 2H), 7.06 (s, 2H), 2.72 (t, J = 7.7 Hz, 4H), 1.76 – 1.69 (m, 4H), 1.45 – 1.34 (m, 12H), 0.93 (t, J = 6.4 Hz, 6H). ^{13}C {^{1}H} NMR (126 MHz, $CDCl_3$) δ 152.65, 144.38, 139.01, 129.00, 126.04, 125.55, 121.53, 31.72, 30.67, 30.49, 29.06, 22.64, 14.12. HRMS (ESI) calculated for $C_{26}H_{32}N_2S_3$ $[M+H]^+$: m/z 469.1798. Found: m/z 469.1800.

Synthesis of 4,7-bis(5-bromo-4-hexylthiophen-2-yl)benzo[c][1,2,5]thiadiazole (1). The compound TBT (150 mg, 0.32 mmol) was brominated with NBS (131 mg, 0.74 mmol) according

to general procedure for bromination of thiophene oligomers. The product (compound 1) was obtained as a red crystalline solid (150 mg, 0.24 mmol, 75% yield). ^{1}H NMR (500 MHz, $CDCl_3$) δ 7.80 (s, 2H), 7.78 (s, 2H), 2.66 (t, J = 7.7 Hz, 4H), 1.73 – 1.66 (m, 4H), 1.44 – 1.34 (m, 12H), 0.93 (t, J = 6.4 Hz, 6H). ^{13}C {^{1}H} NMR (126 MHz, $CDCl_3$) δ 152.27, 143.10, 138.51, 128.10, 125.36, 124.91, 111.62, 31.64, 29.75, 29.69, 28.96, 22.62, 14.11. HRMS (ESI) calculated for $C_{26}H_{30}Br_2N_2S_3$ $[M+H]^+$: m/z 626.9917. Found: m/z 626.9974.

Synthesis of 4,7-bis(3-hexyl-[2,2'-bithiophen]-5-yl)benzo[c][1,2,5]thiadiazole (T2BT2). Following the general procedure Stille coupling, T2BT2 was synthesized by reacting compound **1** (200 mg, 0.32 mmol) with 2-(tributylstannyl)thiophene (274 mg, 0.73 mmol). The product was obtained as a purple solid (167 mg, 0.26 mmol, 83% yield). ^{1}H NMR (500 MHz, $CDCl_3$) δ 8.02 (s, 2H), 7.86 (s, 2H), 7.38 (d, J = 5.1 Hz, 1H), 7.26 (d, J = 3.4 Hz, 2H), 7.15 – 7.11 (dd, J = 4.2 Hz, 2H), 2.91 – 2.84 (t, J = 6.4 Hz, 4H), 1.81 – 1.73 (t, J = 7.1 Hz, 4H), 1.46 (dd, J = 13.7, 6.6 Hz, 4H), 1.38 – 1.34 (m, 8H), 0.92 (t, J = 6.7 Hz, 6H). ^{13}C {^{1}H} NMR (126 MHz, $CDCl_3$) δ 152.59, 140.56, 136.94, 136.04, 132.38, 130.59, 127.51, 126.09, 125.60, 125.51, 125.27, 31.69, 30.65, 29.50, 29.30, 22.66, 14.11. HRMS (ESI) calculated for $C_{34}H_{36}N_2S_5$ $[M]^+$: m/z 632.1482. Found: m/z 632.1470.

Synthesis of 4,7-bis(5'-bromo-3-hexyl-[2,2'-bithiophen]-5-yl)benzo[c][1,2,5]thiadiazole (2). The compound T2BT2 (200 mg, 0.32 mmol) was brominated with NBS (131 mg, 0.74 mmol) according to the general procedure for bromination of thiophene oligomers. The product (compound **2**) was obtained as a black solid (197.4 mg, 0.25 mmol, 79% yield). ^{1}H NMR (500 MHz, $CDCl_3$) δ 7.99 (s, 2H), 7.85 (s, 2H), 7.08 (d, J = 3.8 Hz, 2H), 7.00 (d, J = 3.8 Hz, 2H), 2.85 – 2.79 (t, J = 7.4 Hz, 4H), 1.78 – 1.71 (m, 4H), 1.48 – 1.43 (m, 4H), 1.36 (dt, J = 7.1, 3.7 Hz, 8H), 0.93 (t, J = 6.9 Hz, 6H). ^{13}C {^{1}H} NMR (126 MHz, $CDCl_3$) δ 152.51, 141.03, 137.53, 137.36,

131.44, 130.49, 130.34, 126.27, 125.47, 125.33, 112.20, 31.67, 30.57, 29.46, 29.32, 22.64, 14.11. HRMS (ESI) calculated for $C_{34}H_{34}Br_2N_2S_5$ $[M]^+$: m/z 789.9672. Found: m/z 789.9662.

Synthesis of 4,7-bis(3,4''-dihexyl-[2,2':5',2''-terthiophen]-5-yl)benzo[c][1,2,5]thiadiazole (T3BT3). Following the general procedure for Suzuki coupling, T3BT3 was synthesized by reacting to compound **2** (150 mg, 0.19 mmol) with 2-(4-hexylthiophen-2-yl)-4,4,5,5-tetramethyl-1,3,2-dioxaborolane (134.2 mg, 0.46 mmol). The product was obtained as a purple solid (117 mg, 0.12 mmol, 64% yield). ^{1}H NMR (500 MHz, $CDCl_3$) δ 7.91 (s, 2H), 7.79 (s, 2H), 7.15 (s, 4H), 7.07 (s, 2H), 6.85 (s, 2H), 2.93 – 2.85 (t, J = 7.5 Hz, 4H), 2.62 (t, J = 7.7 Hz, 4H), 1.83 – 1.73 (dt, J = 14.8, 7.2 Hz, 4H), 1.67 (dt, J = 15.2, 7.5 Hz, 4H), 1.43 – 1.30 (m, 24H), 0.93 (t, J = 6.8 Hz, 12H). ^{13}C {^{1}H} NMR (126 MHz, $CDCl_3$) δ 158.12, 144.23, 140.26, 137.70, 137.21, 136.70, 134.82, 132.79, 130.41, 126.89, 126.44, 125.39, 125.09, 123.77, 119.22, 53.44, 31.71, 31.69, 30.56, 30.54, 30.39, 29.71, 29.62, 29.32, 29.01, 22.67, 22.63, 14.15, 14.12. HRMS (ESI) calculated for $C_{54}H_{64}N_2S_7$ $[M]^+$: *m/z* 964.3109. Found: *m/z* 964.3099.

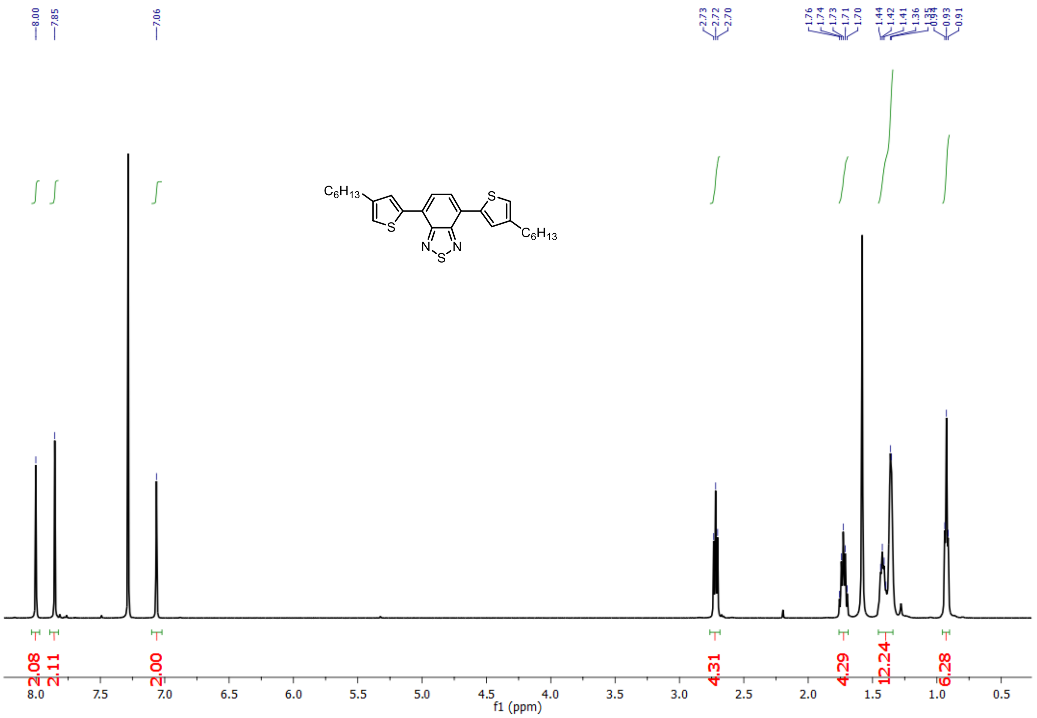

Figure S1. ^{1}H NMR spectrum of **TBT** (500 MHz, $CDCl_3$).

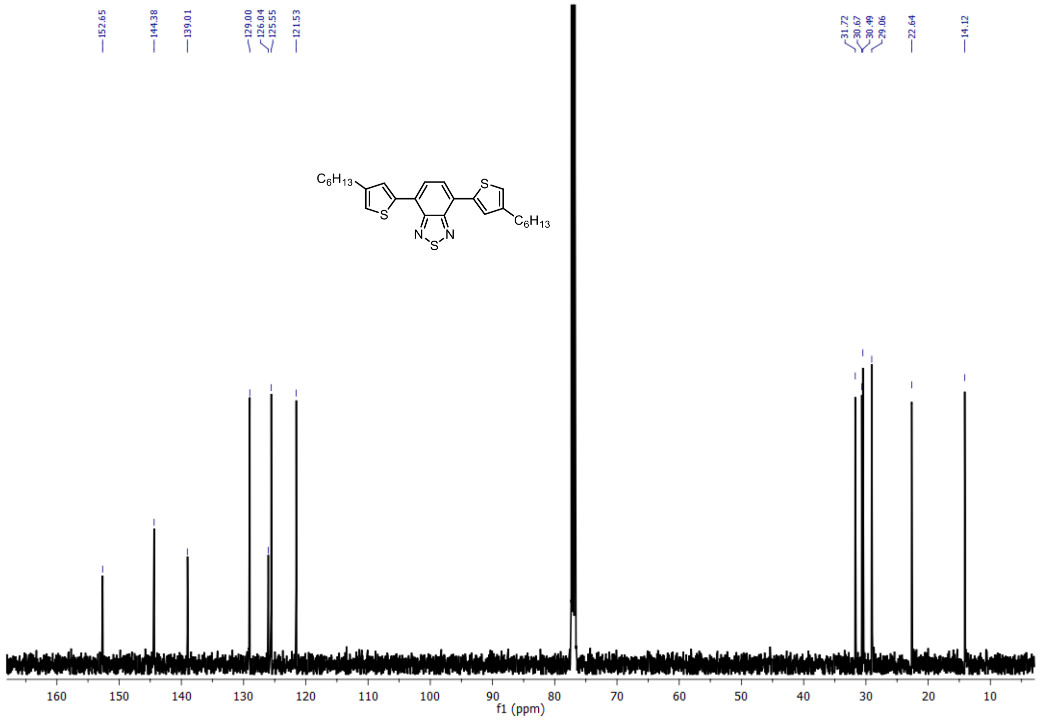

Figure S2. $^{13}C\ \{^{1}H\}$ NMR spectrum of **TBT** (126 MHz, $CDCl_3$).

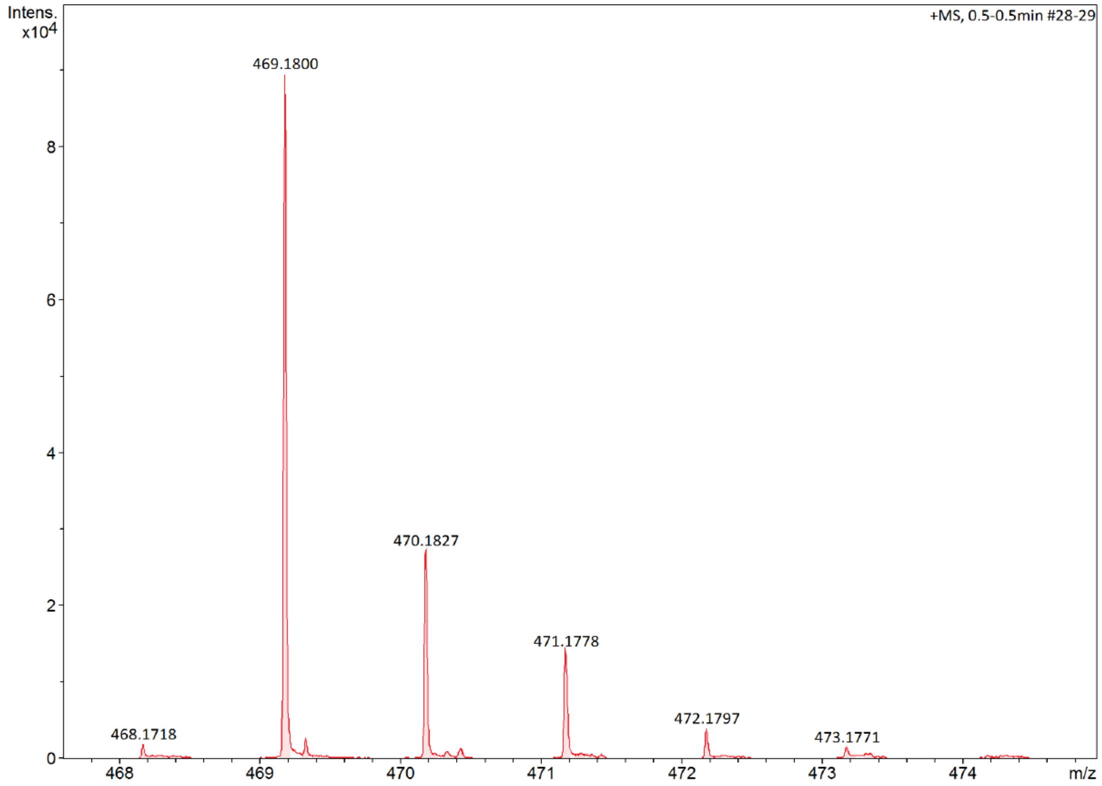

Figure S3. HRMS (m/z) spectrum of **TBT**.

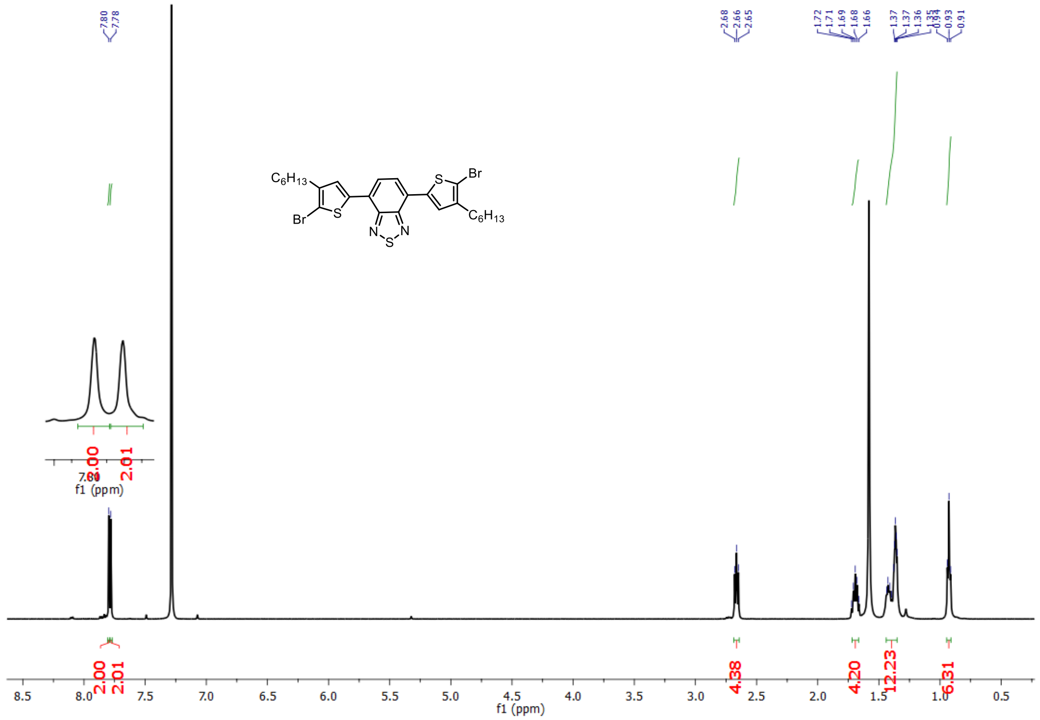

Figure S4. ^{1}H NMR spectrum of compound **1** (500 MHz, $CDCl_3$).

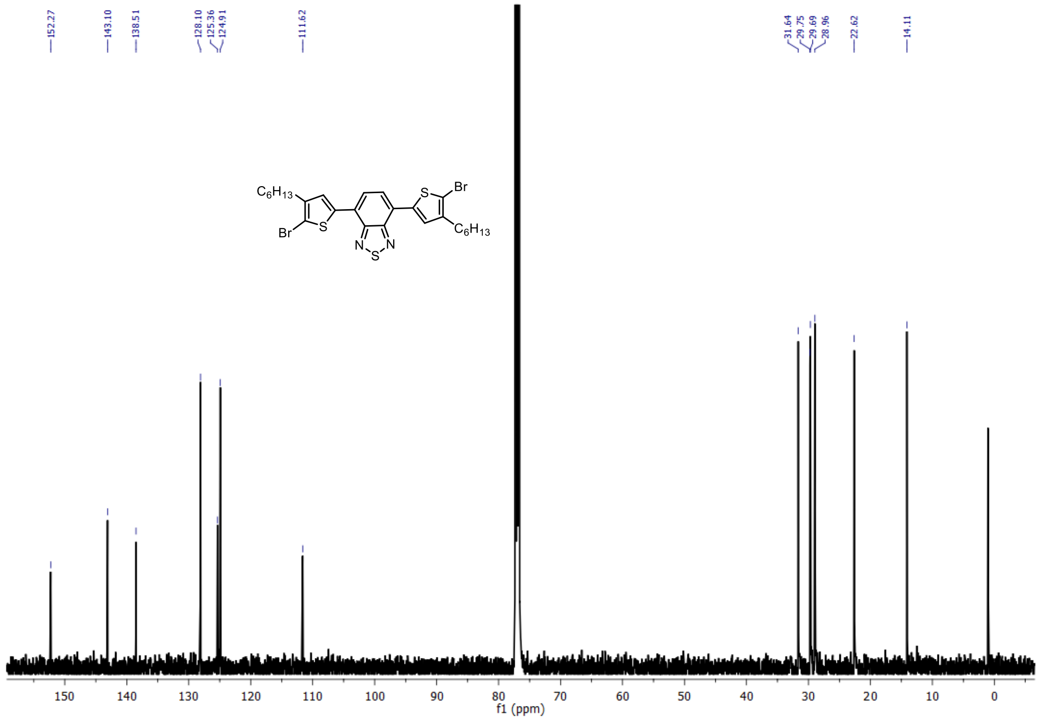

Figure S5. ^{13}C {^{1}H} NMR spectrum of compound **1** (126 MHz, $CDCl_3$).

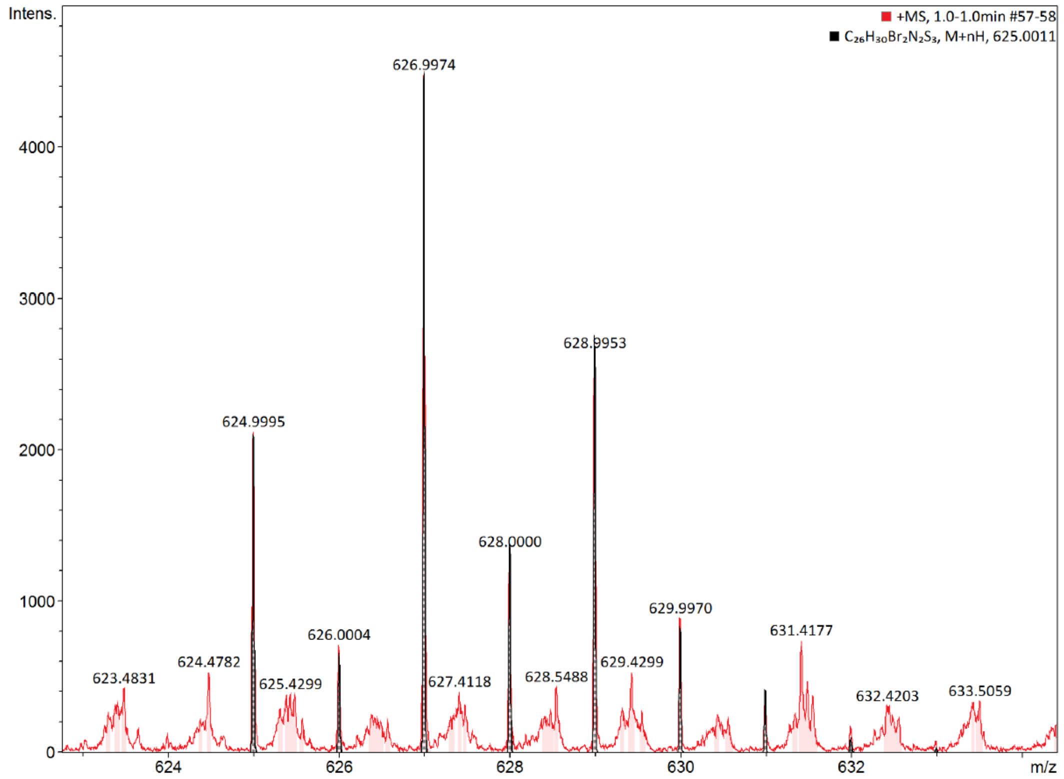

Figure S6. HRMS (m/z) spectrum of compound **1**.

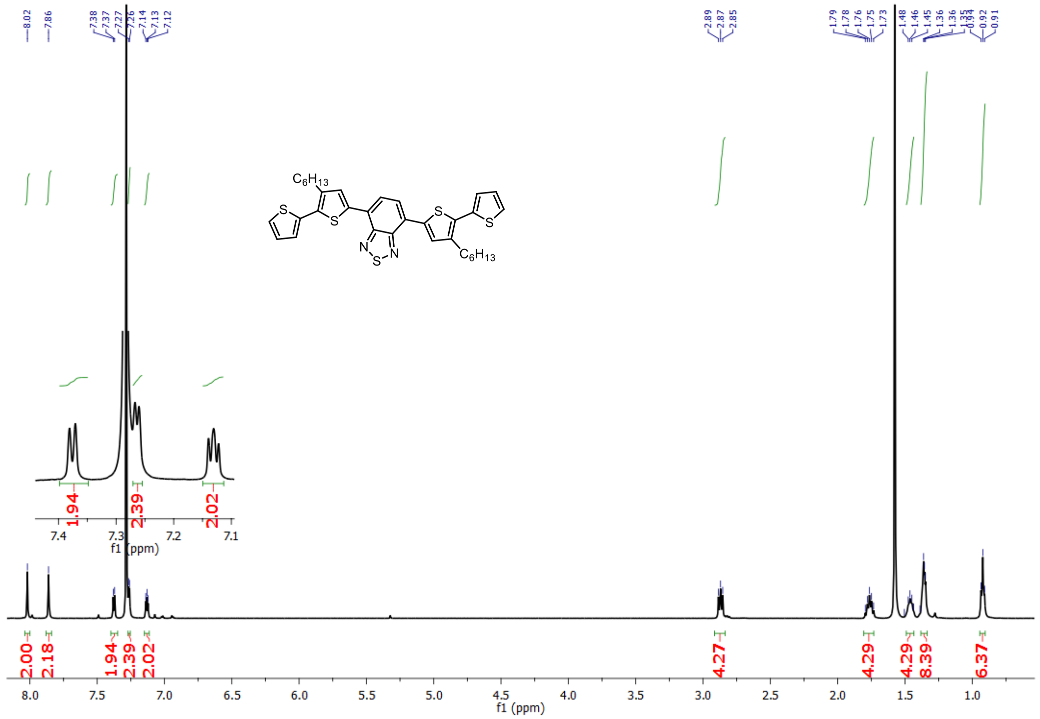

Figure S7. ^{1}H NMR spectrum of **T2BT2** (500 MHz, $CDCl_3$).

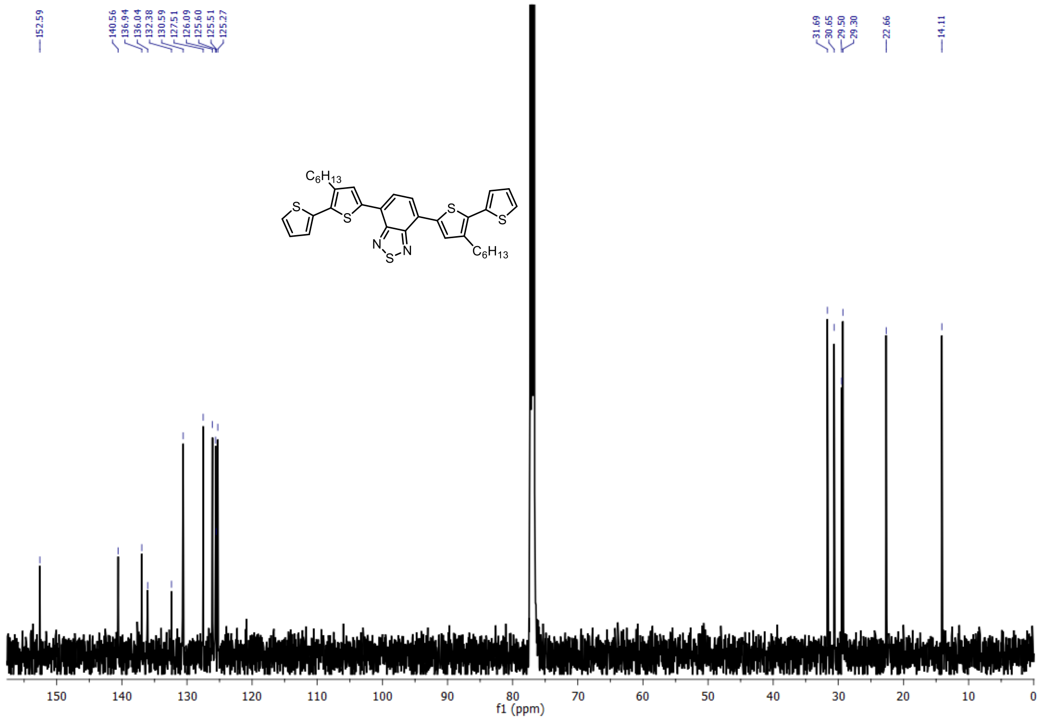

Figure S8. ^{13}C {^{1}H} NMR spectrum of **T2BT2** (126 MHz, $CDCl_3$).

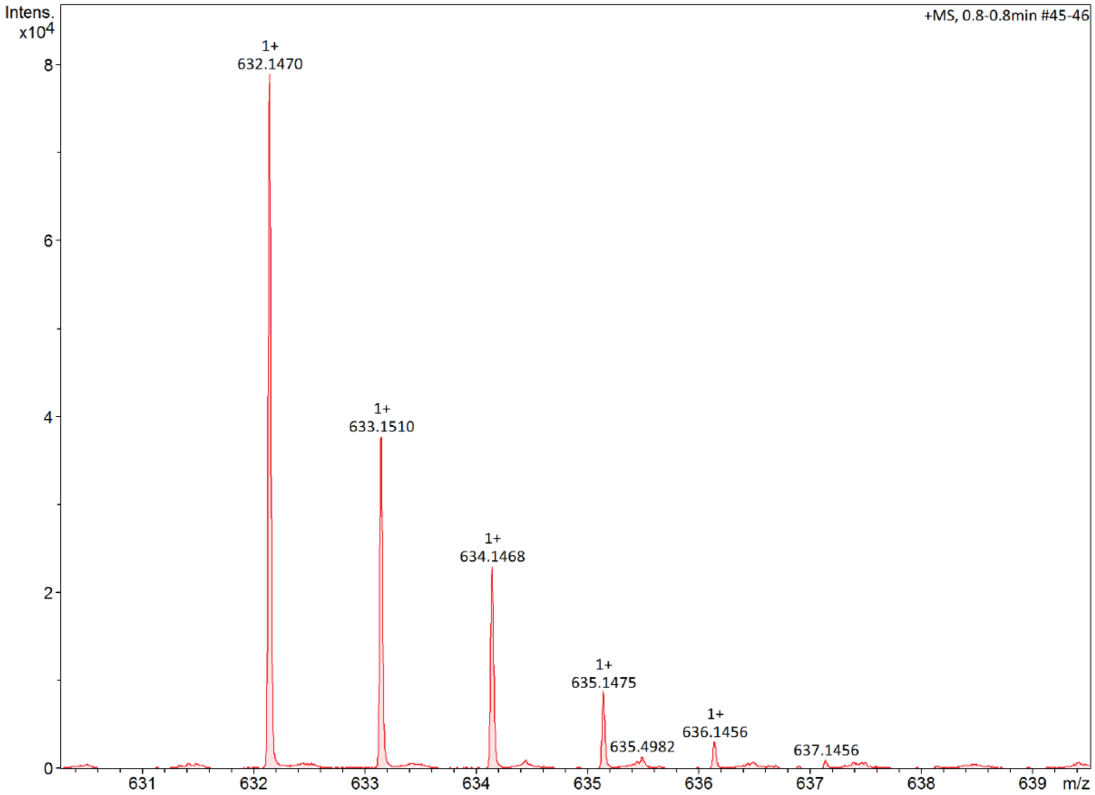

Figure S9. HRMS (m/z) spectrum of **T2BT2**.

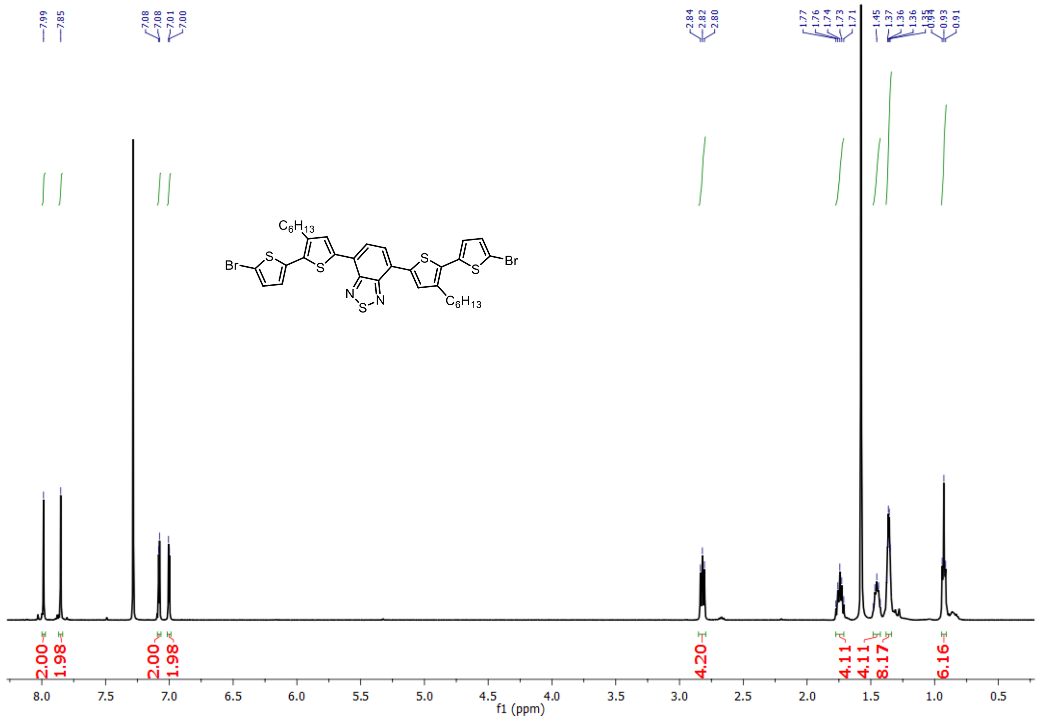

Figure S10. ^{1}H NMR spectrum of compound **2** (500 MHz, $CDCl_3$).

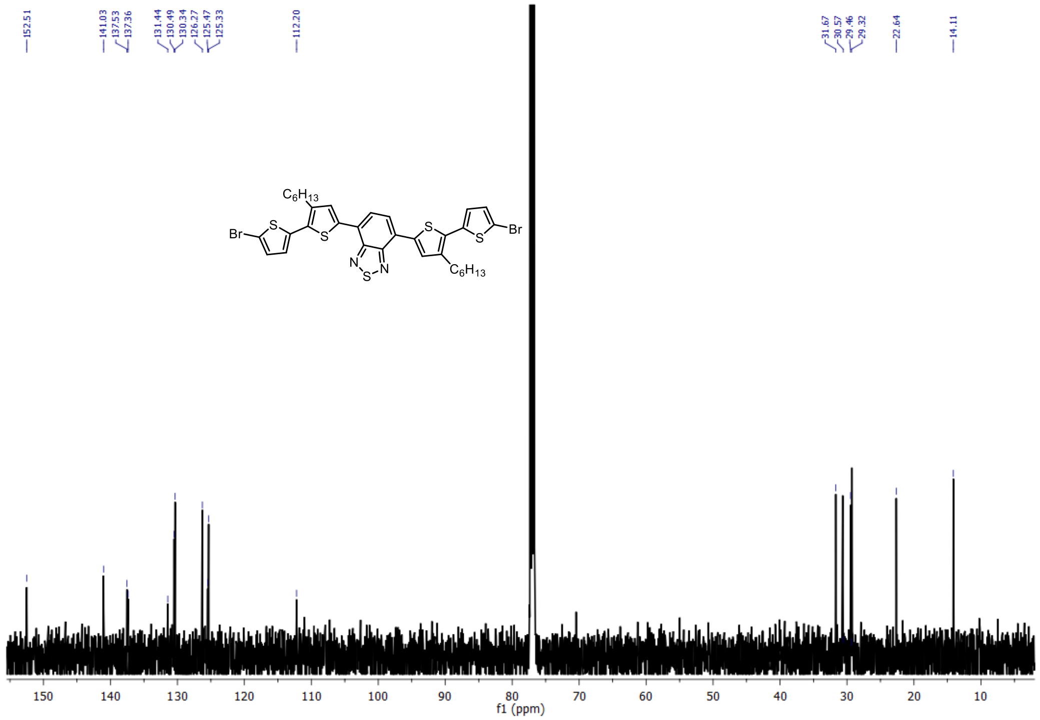

Figure S11. ^{13}C {^{1}H} NMR spectrum of compound **2** (126 MHz, $CDCl_3$).

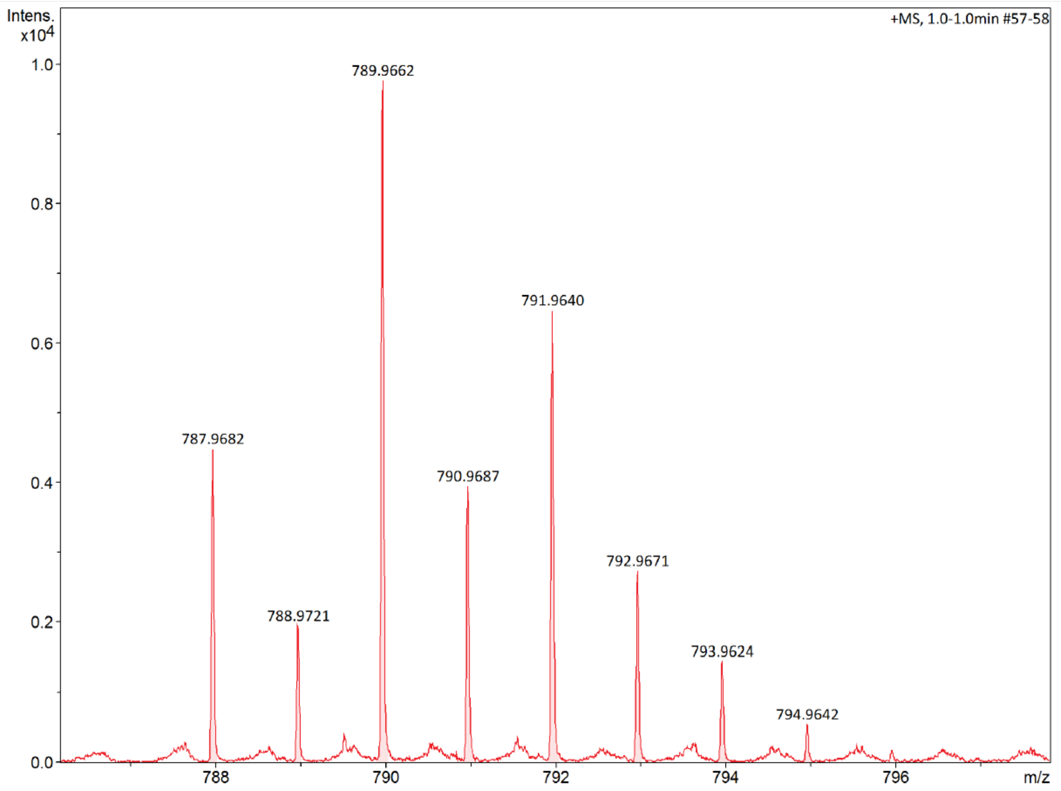

Figure S12. HRMS (m/z) spectrum of compound **(2)**.

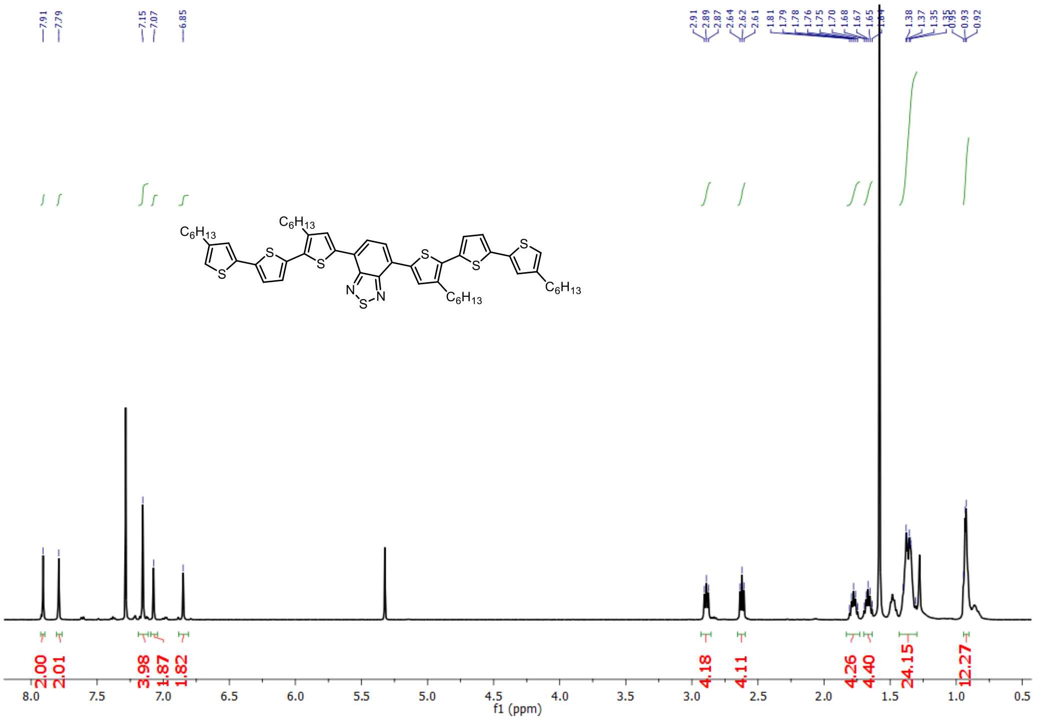

Figure S13. ^{1}H NMR spectrum of **T3BT3** (500 MHz, $CDCl_3$).

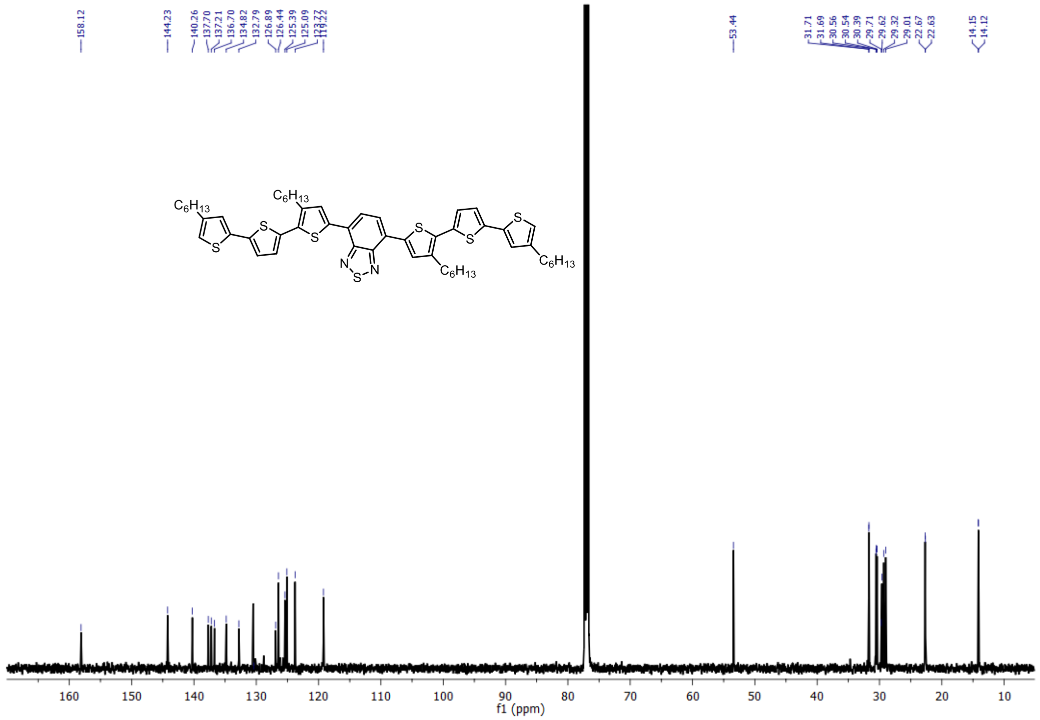

Figure S14. ^{13}C {^{1}H} NMR spectrum of **T3BT3** (126 MHz, $CDCl_3$).

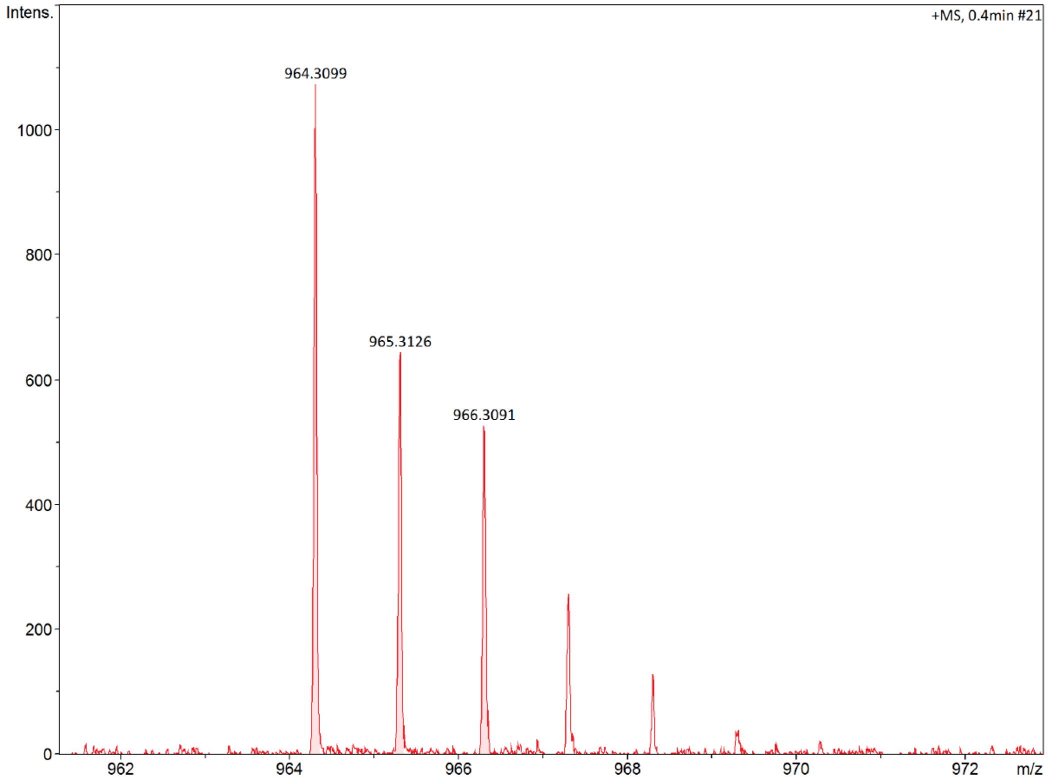

Figure S15. HRMS (m/z) spectrum of **T3BT3**.

Scheme S2. Synthesis of Phenyl, Biphenyl and Fluorene based DAD Compounds

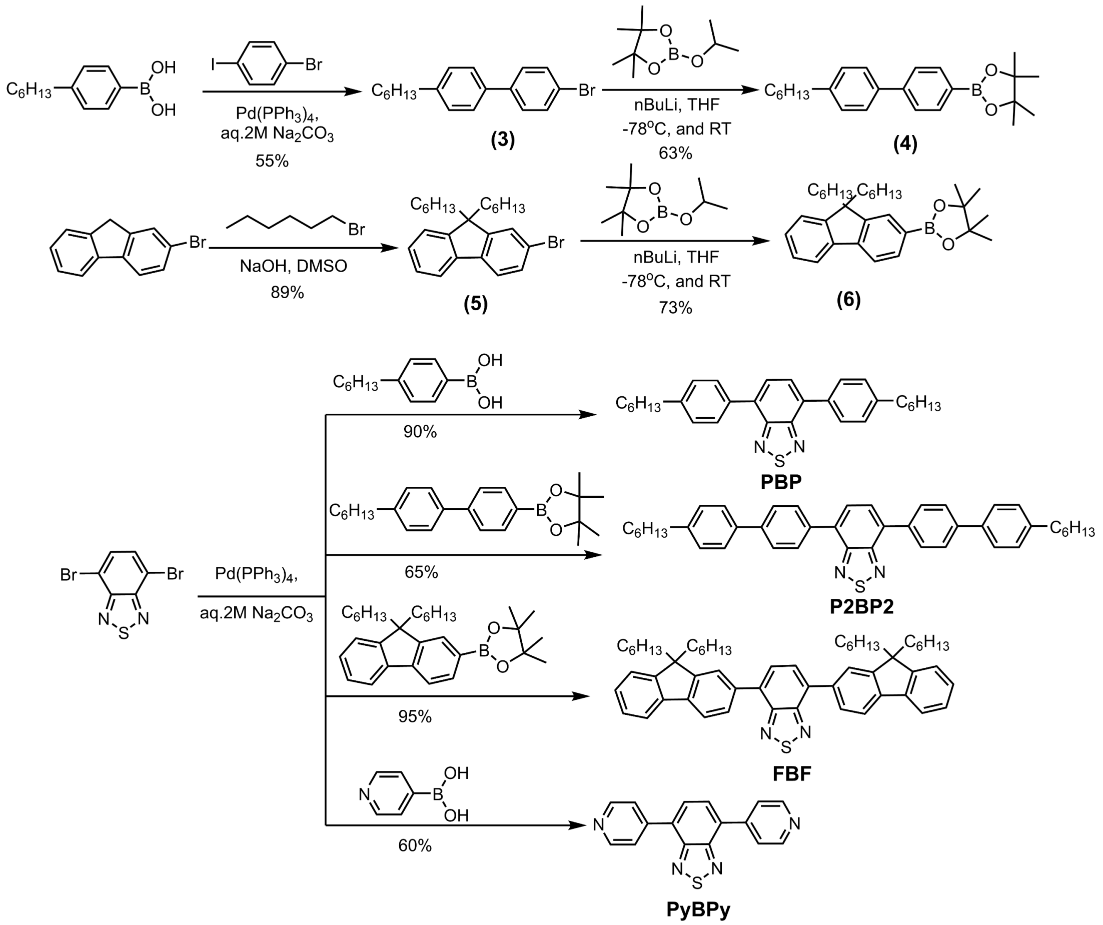

Compounds (**5**)[4], (**6**)[4], were synthesized according to literature procedure.

Synthesis of 4-bromo-4'-hexyl-1,1'-biphenyl (3). Following the general procedure for Suzuki coupling, compound **3** was synthesized by reacting to 4-hexylphenylboronic acid (180 mg, 0.87 mmol) with 1-Bromo-4-iodobenzene (246 mg, 0.87 mmol). The product was obtained as a white solid (151 mg, 0.48 mmol, 55% yield). ^{1}H NMR (500 MHz, $CDCl_3$) δ 7.57 (d, J = 8.3 Hz, 2H), 7.48 (dd, J = 10.5, 8.4 Hz, 4H), 7.28 (d, J = 7.6 Hz, 2H), 2.66 (t, J = 7.7 Hz, 2H), 1.71 – 1.61 (m, 2H), 1.36 (dd, J = 16.4, 5.1 Hz, 6H), 0.92 (t, J = 7.9 Hz, 3H). ^{13}C {^{1}H} NMR (126 MHz, $CDCl_3$) δ 142.60, 140.09, 137.28, 131.78, 128.96, 128.56, 126.76, 121.15, 35.62, 31.74, 31.45, 29.03, 22.62, 14.11. Calc. for $C_{18}H_{21}Br$ $[M+H^+]^+$ (m/z): 317.0827, Found $[M+H^+]^+$: 317.0899.

Synthesis of 2-(4'-hexyl-[1,1'-biphenyl]-4-yl)-4,4,5,5-tetramethyl-1,3,2-dioxaborolane (4). In a 100 mL Schlenk-flask, the solution 150 mg (0.35 mmol) of compound **3** dissolved in 25 mL of dry THF was cooled to -78 °C under N_2 atmosphere and slowly added nBuLi 0.45 mL (3.4 mmol) under nitrogen atmosphere and the resultant mixture was stirred at -78 °C for 30 min. After 120 mg (0.18 mmol) of 2-Isopropoxy-4,4,5,5-tetramethyl-1,3,2-dioxaborolane was added slowly drop wise. The resulting mixture was allowed to room temperature slowly and stirred for 12 hours at room temperature. The reaction mixture was washed by aq. NH_4Cl and extracted by DCM (2x60 mL). The organic layer was washed with brine, and dried by $NaSO_4$, filtrated and then the solvent was removed under reduced pressure, and the residue was chromatographed by flash column with 2:8 dichloromethane/hexane. The pure product compound **4** was isolated as a white solid (150 mg, 0.35 mmol, 73% yield) and the compound **4** was immediately stored in refrigerator. ^{1}H NMR (500 MHz, $CDCl_3$) δ 7.89 (d, *J* = 7.8 Hz, 2H), 7.59 (dd, *J* = 31.7, 7.8 Hz, 4H), 7.27 (d, *J* = 7.4 Hz, 2H), 2.67 (t, *J* = 7.6 Hz, 2H), 1.72 – 1.63 (m, 2H), 1.38 (s, 12H), 1.35 (m, 6H), 0.91 (t, *J* = 7.2 Hz,, 3H); ^{13}C {^{1}H} NMR (126 MHz, $CDCl_3$) δ 143.84, 142.50, 138.28, 135.21, 134.73, 131.24, 128.83, 127.70, 127.04, 126.26, 83.78, 35.64, 31.75, 31.47, 29.05, 24.88, 22.63, 14.12. Calc. for $C_{24}H_{33}BO_2$ $[M+H^{+}]^{+}$ (m/z): 365.2574, Found $[M+H^{+}]^{+}$: 365.2656.

Synthesis of 4,7-bis(4-hexylphenyl)benzo[c][1,2,5]thiadiazole (PBP). Following the general procedure for Suzuki coupling PBP was synthesized by reacting to 4,7-dibromo-2,1,3-benzothiadiazole (200 mg, 0.68 mmol) with 4-hexylphenylboronic acid (336 mg, 1.63 mmol). The product was obtained as a bright fluorescent yellow solid (151 mg, 0.48 mmol, 73% yield). ^{1}H NMR (500 MHz, $CDCl_3$) δ 7.91 (d, *J* = 7.7 Hz, 4H), 7.79 (s, 2H), 7.39 (d, *J* = 7.8 Hz, 4H), 2.72 (t, *J* = 7.7 Hz, 4H), 1.78 – 1.65 (m, 4H), 1.39 (dd, *J* = 31.0, 4.5 Hz, 12H), 0.94 (d, *J* = 5.9 Hz, 6H). ^{13}C {^{1}H} NMR (126 MHz, $CDCl_3$) δ 154.20, 143.33, 134.82, 133.07, 129.07, 128.72, 127.85,

35.83, 31.79, 31.43, 29.11, 22.65, 14.15. Calc. for $C_{30}H_{36}N_2S_2$ $[M+H^+]^+$ (m/z): 457.2672, Found $[M+H^+]^+$: 457.2672.

Synthesis of 4,7-bis(4'-hexyl-[1,1'-biphenyl]-4-yl)benzo[c][1,2,5]thiadiazole (P2BP2). Following the general procedure for Suzuki coupling, P2BP2 was synthesized by reacting to 4,7-dibromo-2,1,3-benzothiadiazole (200 mg, 0.68 mmol) with compound **4** (594 mg, 1.63 mmol). The product was obtained as a bright fluorescent pale-yellow solid (268 mg, 0.44 mmol, 65% yield). ^{1}H NMR (500 MHz, $CDCl_3$) δ 8.09 (d, *J* = 8.0 Hz, 4H), 7.89 (s, 2H), 7.81 (d, *J* = 8.0 Hz, 4H), 7.64 (d, *J* = 7.7 Hz, 4H), 7.32 (d, *J* = 7.7 Hz, 4H), 2.70 (t, *J* = 7.5 Hz, 4H), 1.69 (m, 4H), 1.46 – 1.31 (m, 12H), 0.93 (t, *J* = 7.1 Hz, 6H). ^{13}C {^{1}H} NMR (126 MHz, $CDCl_3$) δ 154.18, 142.47, 141.20, 137.96, 136.03, 132.94, 129.58, 128.94, 127.99, 127.21, 126.98, 35.68, 31.77, 31.49, 29.09, 22.64, 14.13. Calc. for $C_{42}H_{44}N_2S$ $[M+H^+]^+$ (m/z): 609.3298, Found $[M+H^+]^+$: 609.3299.

Synthesis of 4,7-bis(9,9-dihexyl-9H-fluoren-2-yl)benzo[c][1,2,5]thiadiazole (FBF). Following the general procedure for Suzuki coupling, FBF was synthesized by reacting to 4,7-dibromo-2,1,3-benzothiadiazole (200 mg, 0.68 mmol) with compound **6** (751 mg, 1.63 mmol). The product was obtained as a bright fluorescent yellow solid (517 mg, 0.65 mmol, 95% yield). ^{1}H NMR (500 MHz, $CDCl_3$) δ 8.05 (d, *J* = 7.8 Hz, 2H), 7.98 (s, 2H), 7.94 – 7.86 (m, 4H), 7.80 (d, *J* = 7.1 Hz, 2H), 7.39 (m, 6H), 2.15 – 1.99 (m, 8H), 1.20 – 1.05 (m, 24H), 0.80 (t, *J* = 6.9 Hz, 20H). ^{13}C {^{1}H} NMR (126 MHz, $CDCl_3$) δ 154.37, 151.32, 151.10, 141.32, 140.66, 136.18, 133.61, 128.12, 127.90, 127.26, 126.84, 123.91, 122.96, 119.95, 119.70, 55.21, 40.32, 31.50, 29.76, 23.86, 22.60, 14.03. Calc. for $C_{56}H_{68}N_2S$ $[M+H^+]^+$ (m/z): 801.5176, Found $[M+H^+]^+$: 801.5152.

Synthesis of 4,7-di(pyridin-4-yl)benzo[c][1,2,5]thiadiazole (PyBPy). Following the general procedure for Suzuki coupling, PyBPy was synthesized by reacting to 4,7-dibromo-2,1,3-benzothiadiazole (200 mg, 0.68 mmol) with 4-pyridinylboronic acid (200 mg, 1.63 mmol). The

product was obtained as a pale yellow solid (118 mg, 0.41 mmol, 65% yield). ^{1}H NMR (500 MHz, $CDCl_3$) δ 8.83 (d, *J* = 5.8 Hz, 4H), 7.96 (d, *J* = 5.4 Hz, 2\4H), 7.95 (s, 2H). ^{13}C {^{1}H} NMR (126 MHz, $CDCl_3$) δ 153.50, 150.30, 144.20, 131.99, 128.52, 123.60. Calc. for $C_{16}H_{10}N_4S$ $[M+H^+]^+$ (m/z): 291.0626, Found $[M+H^+]^+$: 291.0698.

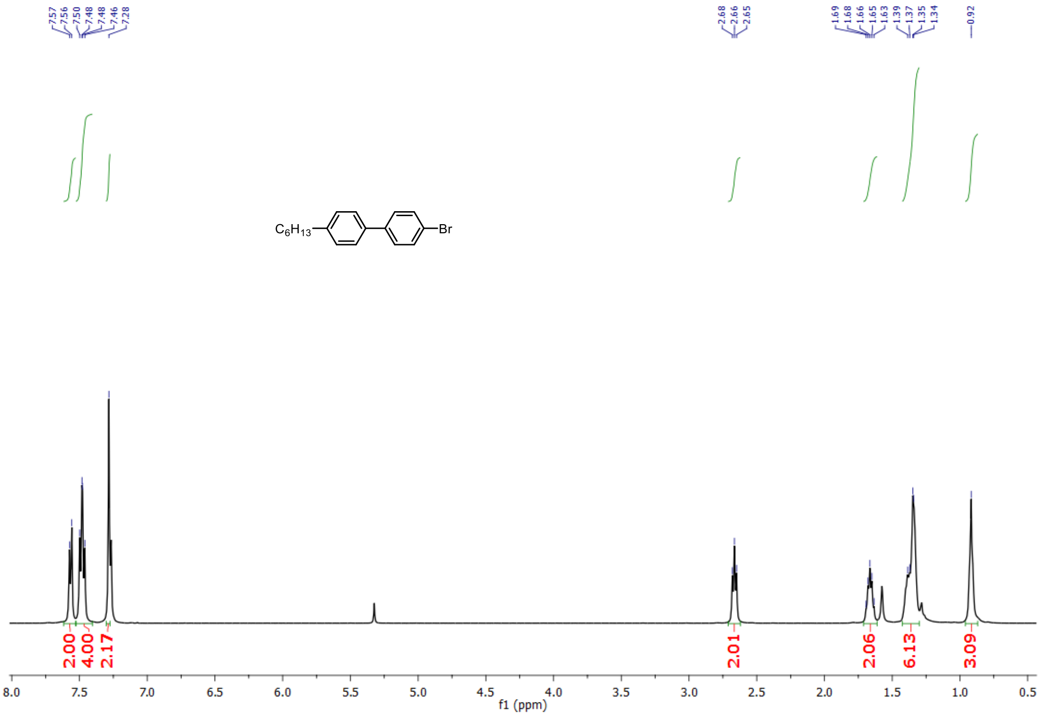

Figure S16. ^{1}H NMR (500 MHz, $CDCl_3$) spectrum of compound **3**.

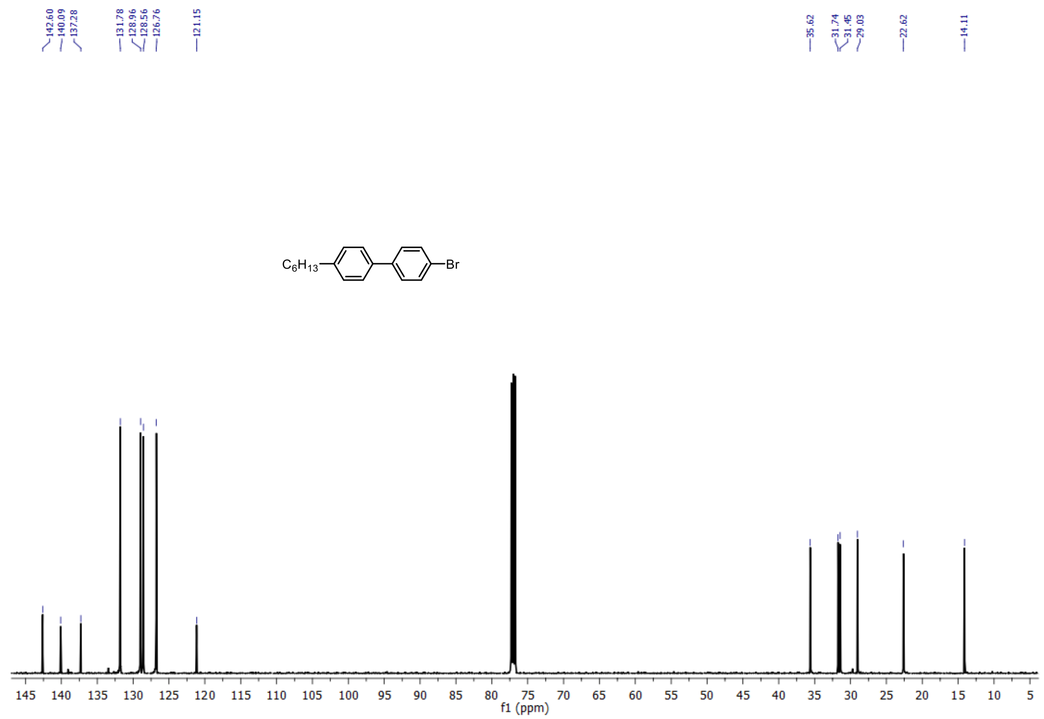

Figure S17. ^{13}C {^{1}H} NMR spectrum of compound **3** (126 MHz, $CDCl_3$).

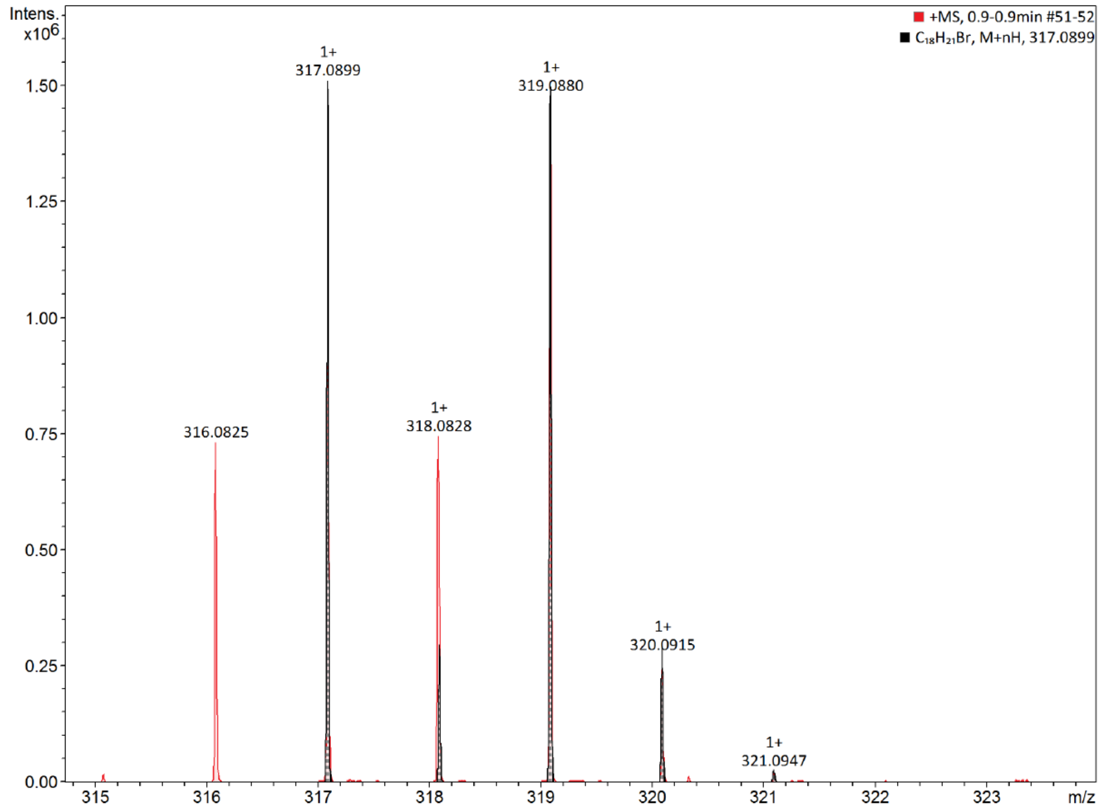

Figure S18. HRMS (m/z) spectrum of compound **3**.

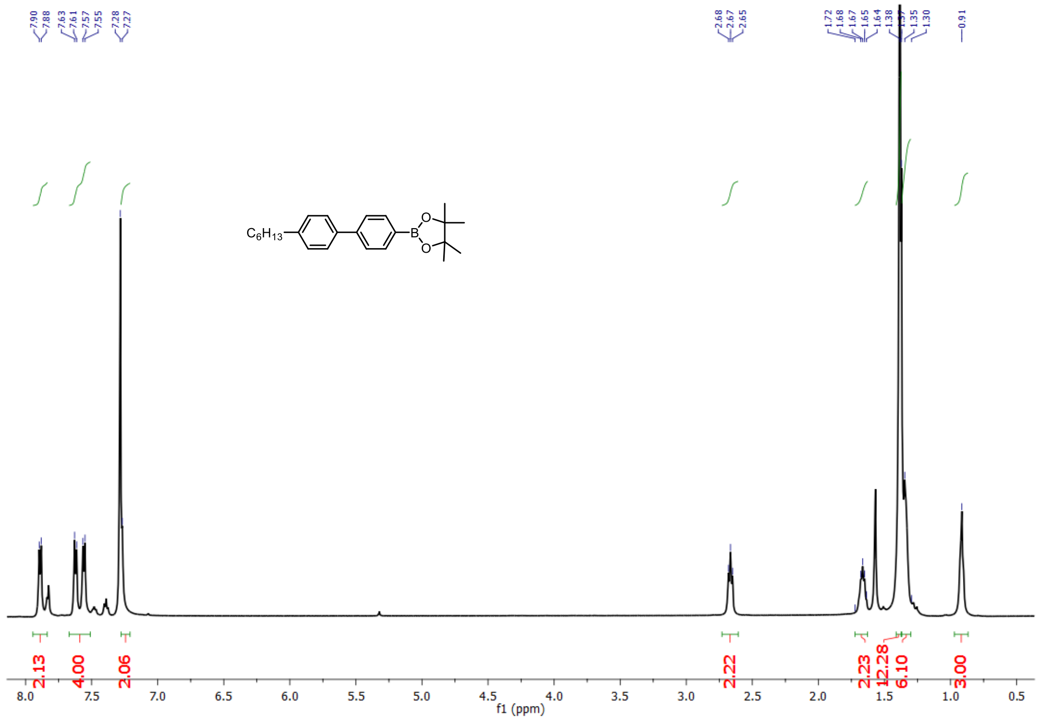

Figure S19. ^{1}H NMR spectrum of compound **4** (500 MHz, $CDCl_3$).

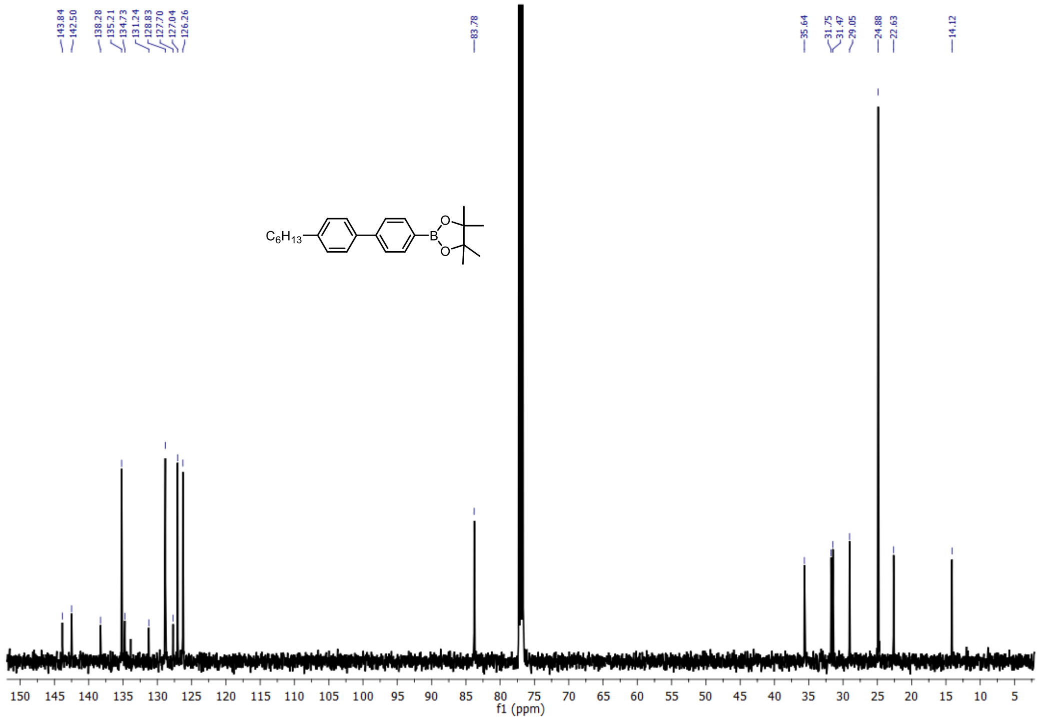

Figure S20. $^{13}C\ \{^{1}H\}$ NMR spectrum of compound **4** (126 MHz, $CDCl_3$).

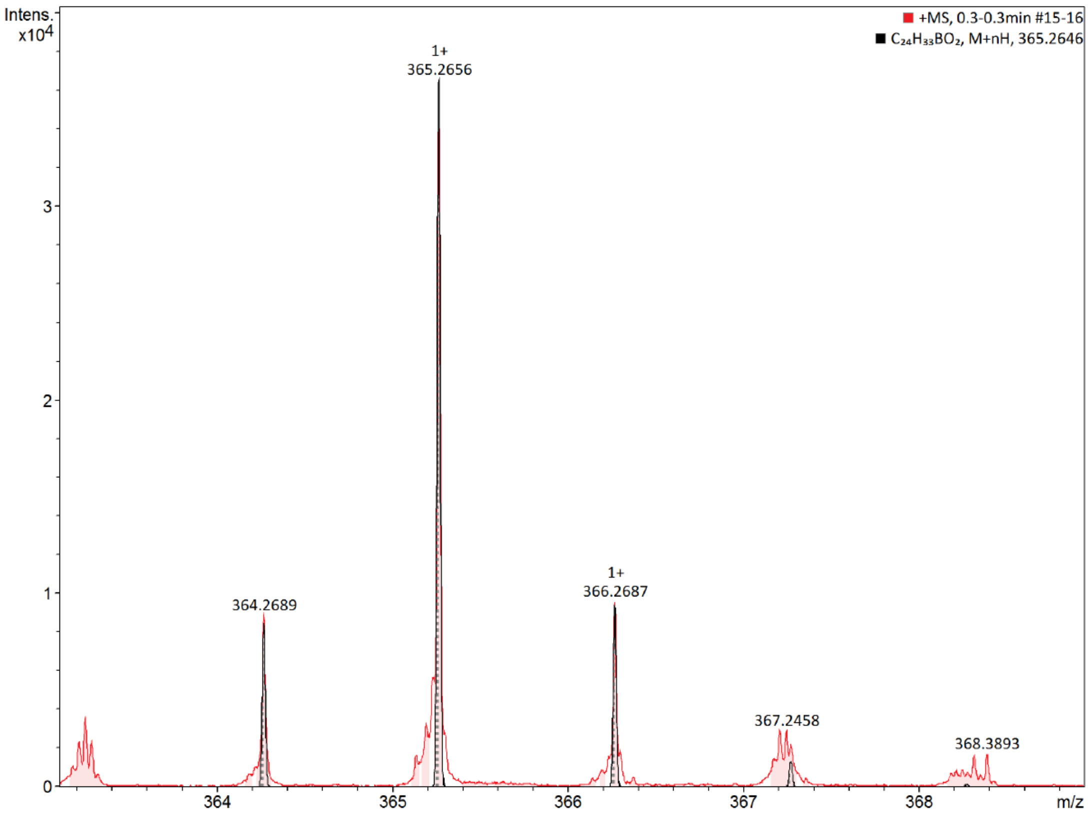

Figure S21. HRMS (m/z) spectrum of compound **4**.

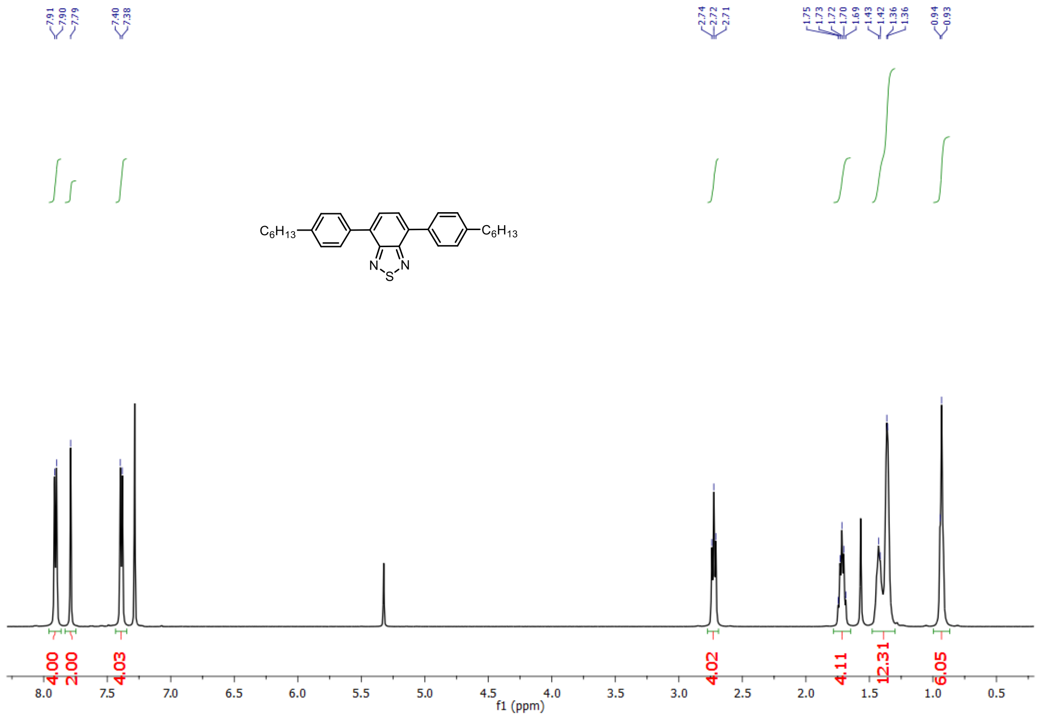

Figure S22. ^{1}H NMR spectrum of **PBP** (500 MHz, $CDCl_3$).

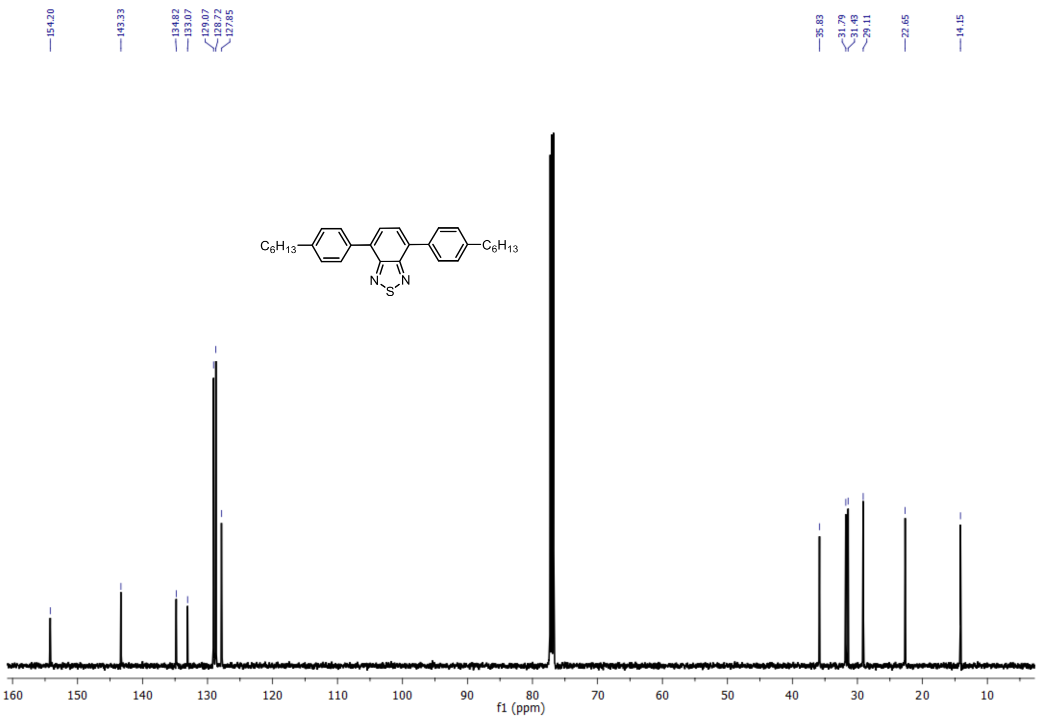

Figure S23. ^{13}C {^{1}H} NMR spectrum of **PBP** (126 MHz, $CDCl_3$).

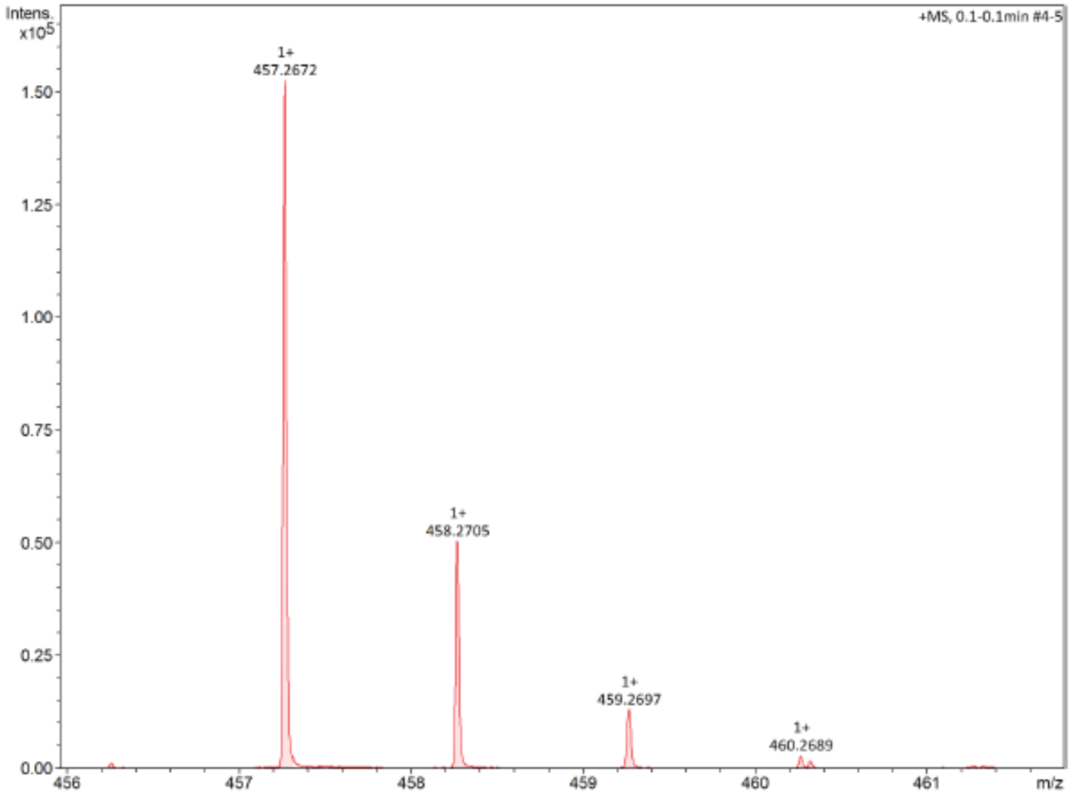

Figure S24. HRMS (m/z) spectrum of compound **PBP**.

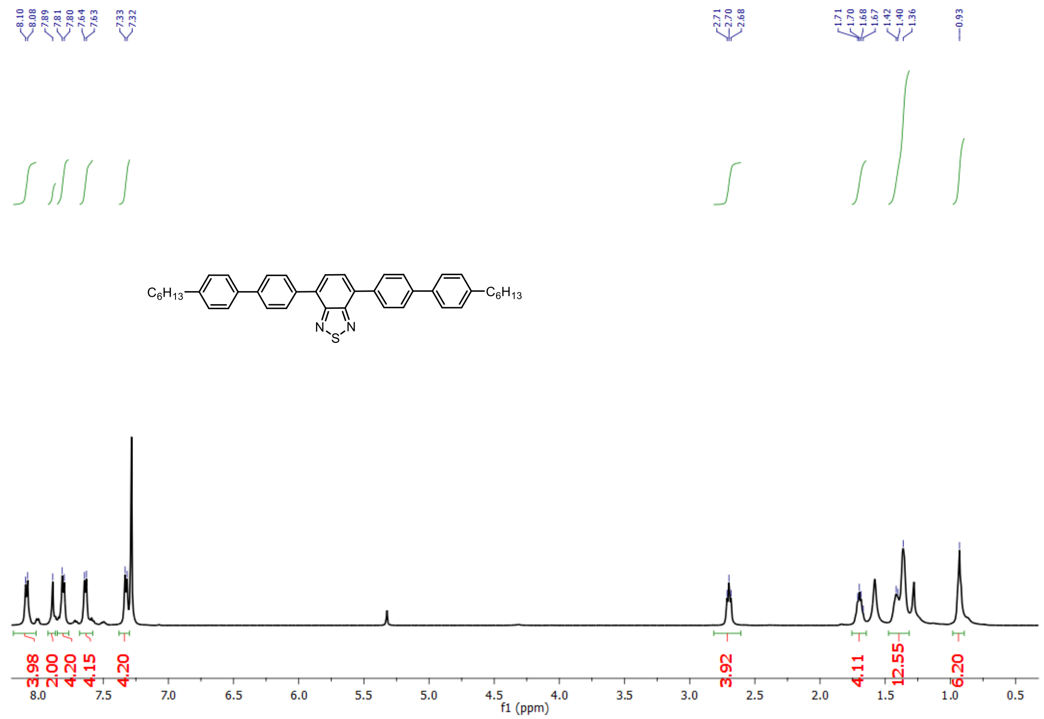

Figure S25. ^{1}H NMR spectrum of **P2BP2** (500 MHz, $CDCl_3$).

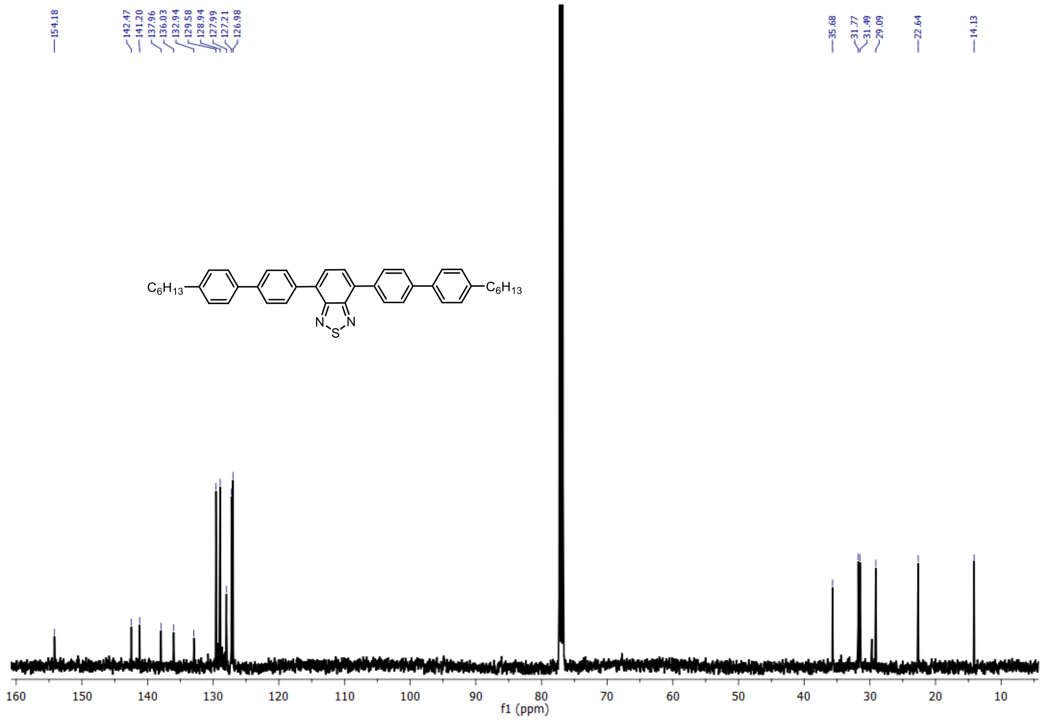

Figure S26. ^{13}C {^{1}H} NMR spectrum of **P2BP2** (126 MHz, $CDCl_3$).

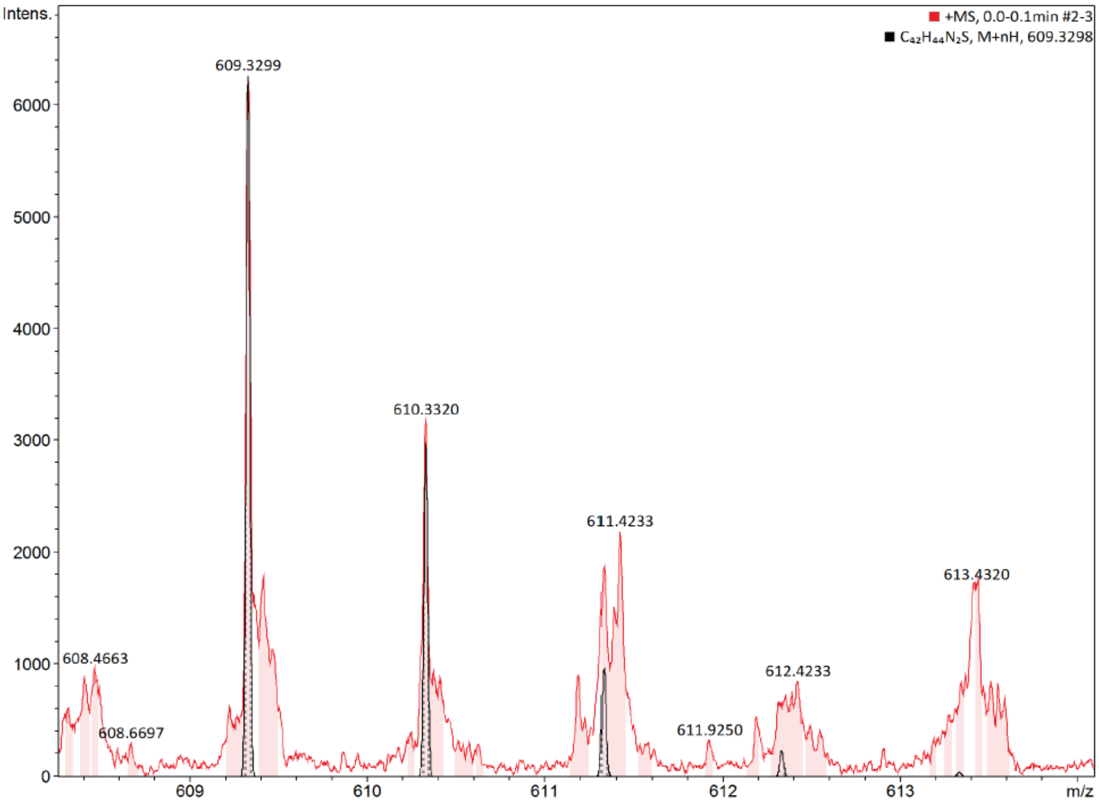

Figure S27. HRMS (m/z) spectrum of compound **P2BP2**.

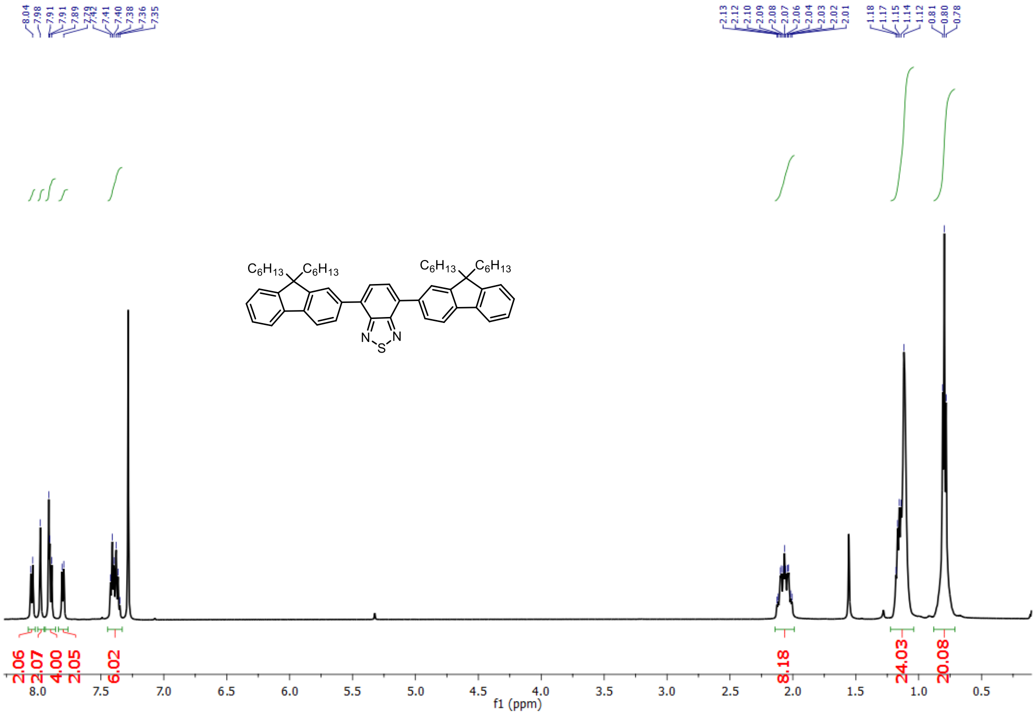

Figure S28. ^{1}H NMR spectrum of **FBF** (500 MHz, $CDCl_3$).

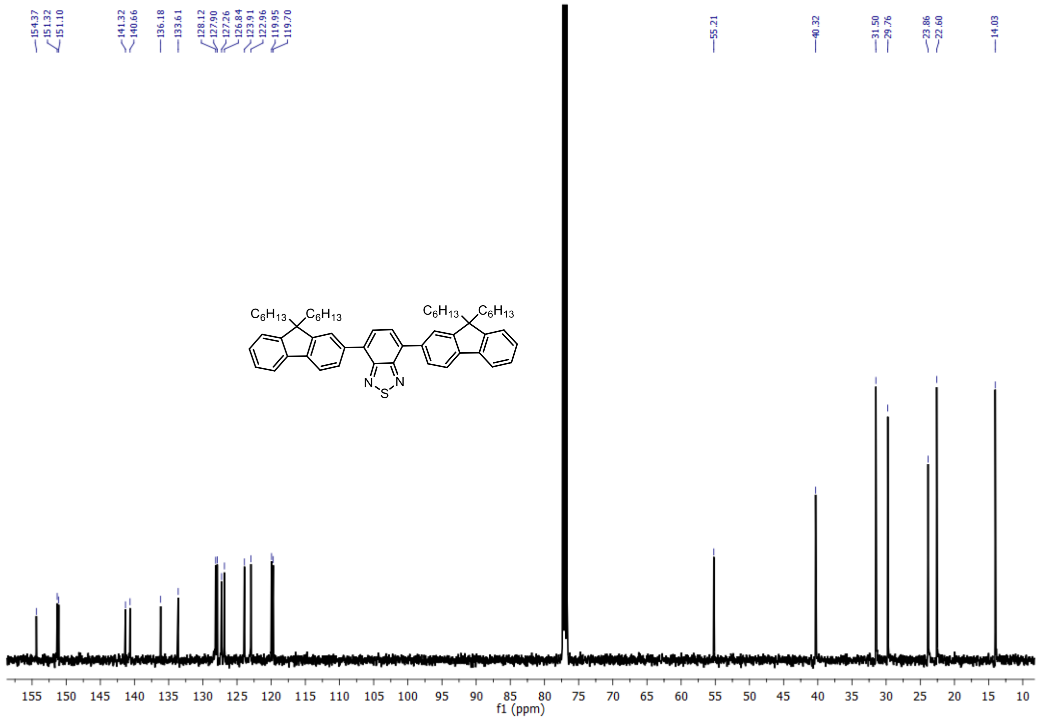

Figure S29. ^{13}C {^1H} NMR spectrum of **FBF** (126 MHz, $CDCl_3$).

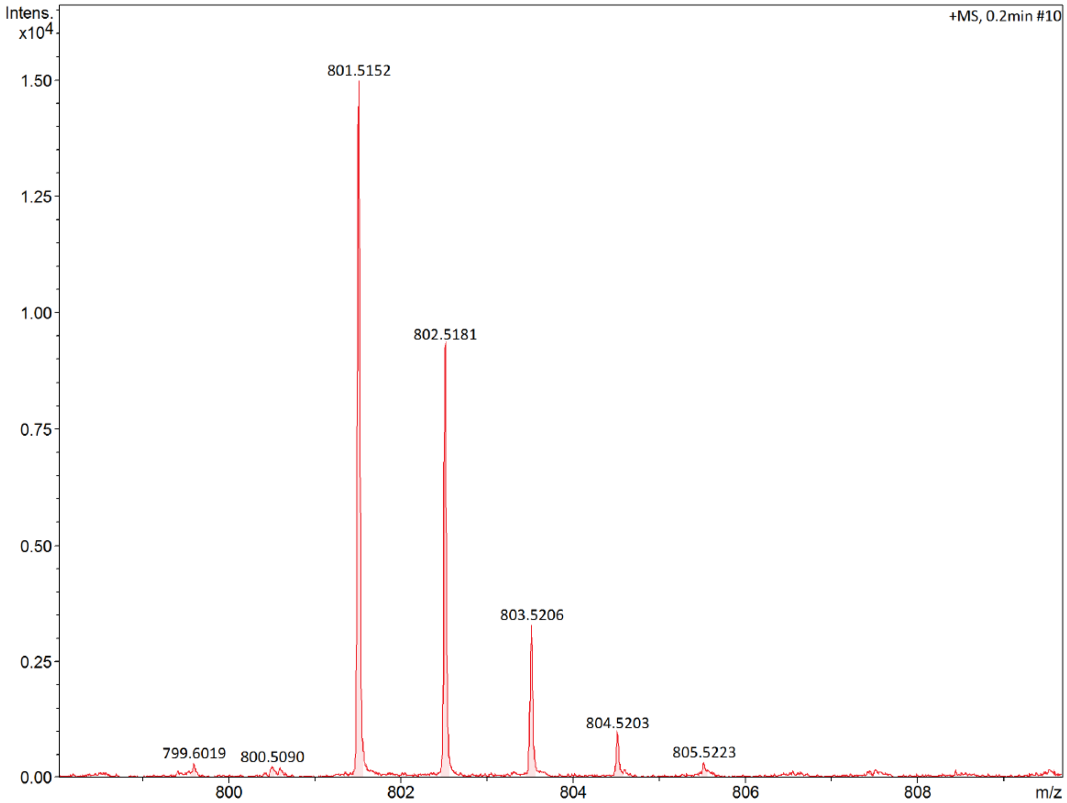

Figure S30. HRMS (m/z) spectrum of compound **FBF**.

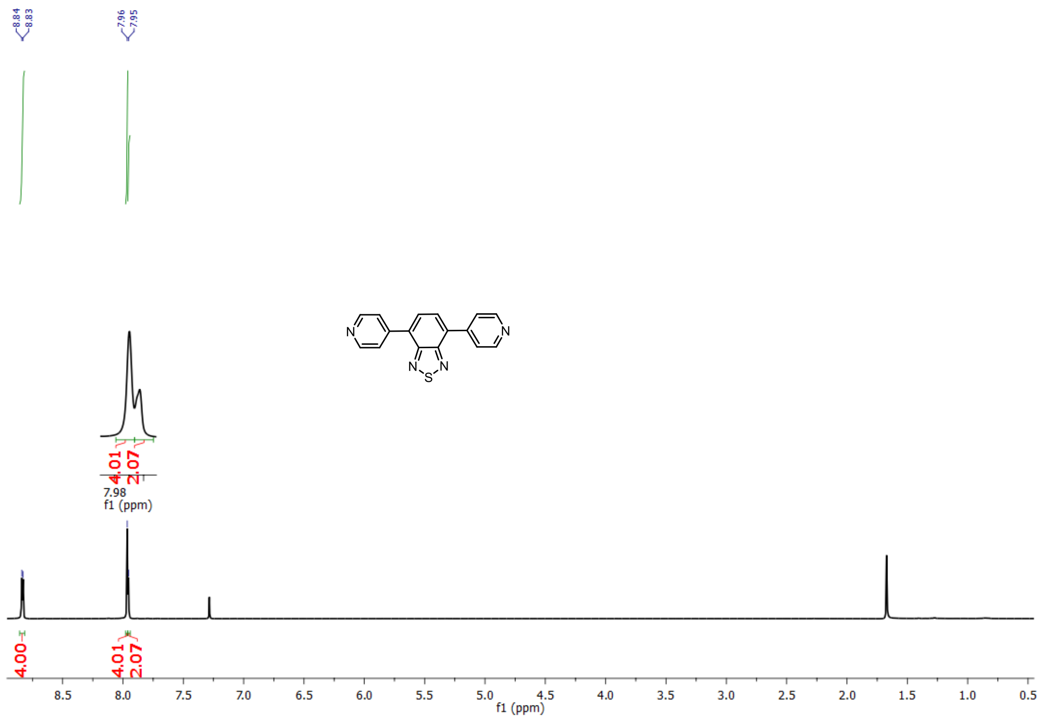

Figure S31. ^{1}H NMR spectrum of **PyBPy** (500 MHz, $CDCl_3$).

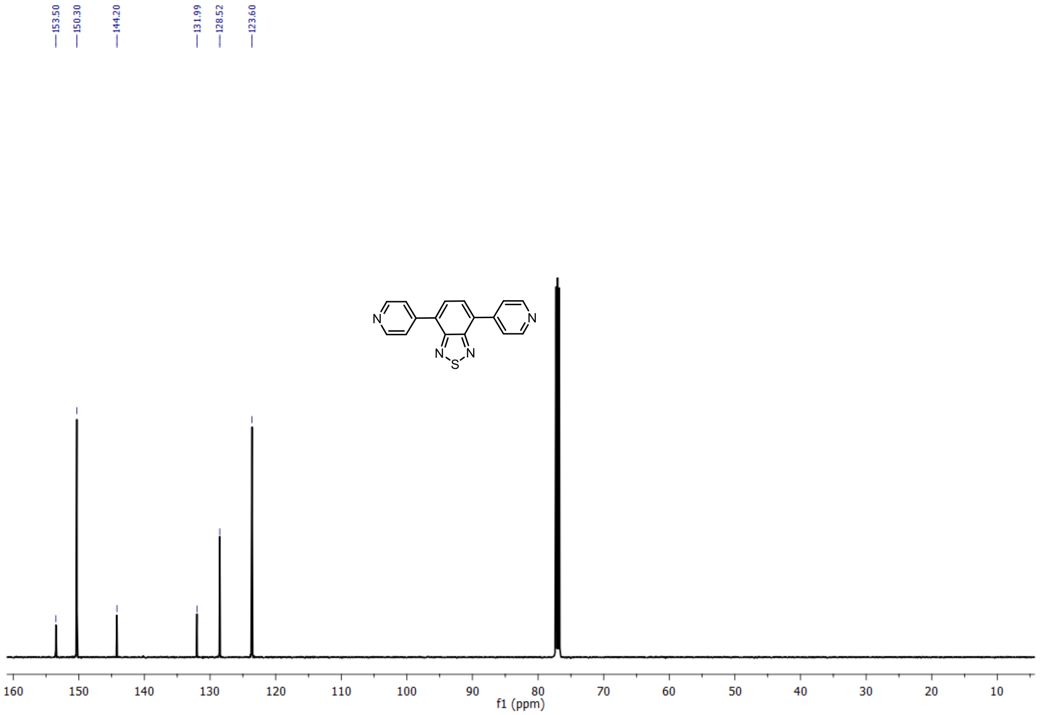

Figure S32. ^{13}C {^{1}H} NMR spectrum of **PyBPy** (126 MHz, $CDCl_3$).

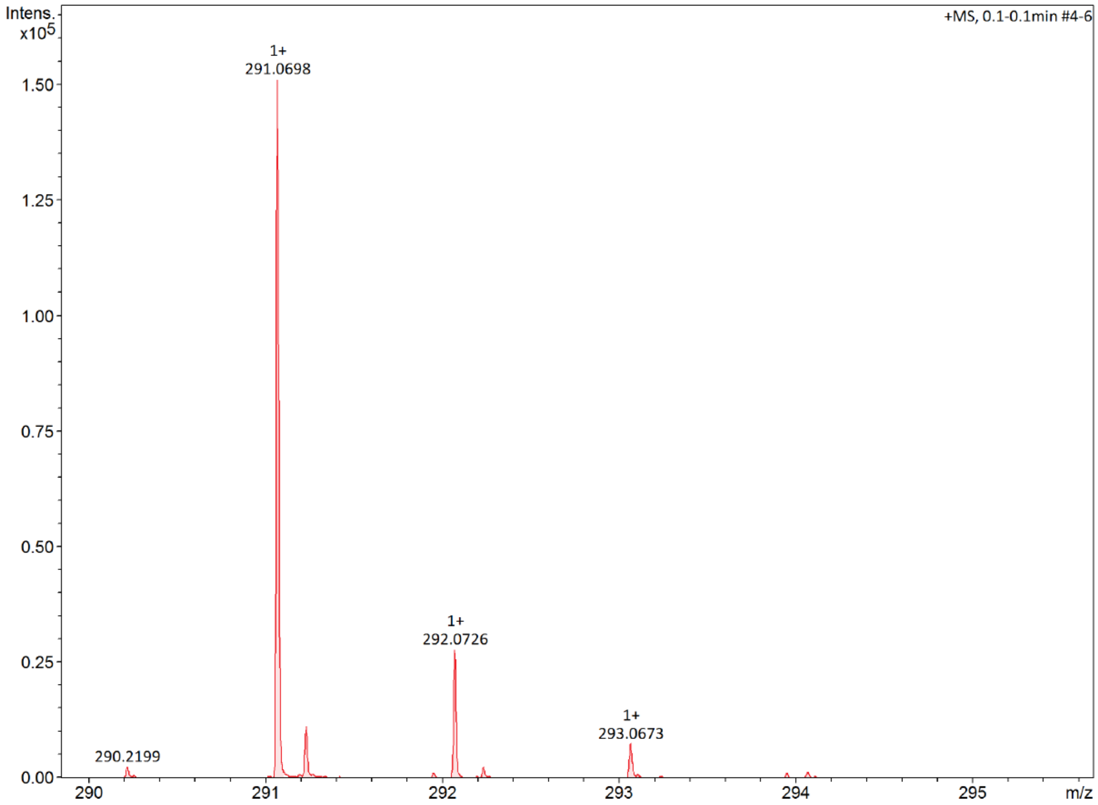

Figure S33. HRMS (m/z) spectrum of **PyBPy**.

Scheme S3. Synthesis of Thiadiazol-Pyridine Based DAD Compounds

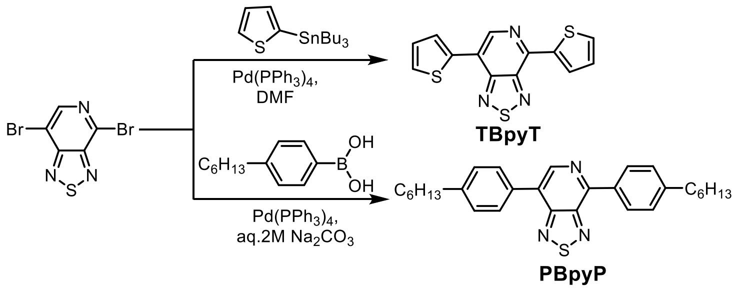

Synthesis of 4,7-di(thiophen-2-yl)-[1,2,5]thiadiazolo[3,4-c]pyridine (TBpyT). Following the general procedure for Stille coupling, TBpyT was synthesized by reacting to 4,7-dibromo-pyridal[2,1,3]thiadiazole (180 mg, 0.61 mmol) with 2-(tributylstannyl)thiophene (524 mg, 1.4 mmol). The product was obtained as an orange red solid (128 mg, 0.43 mmol, 70% yield). ^{1}H NMR (500 MHz, $CDCl_3$) δ 8.88 (s, 1H), 8.73 (d, *J* = 3.7 Hz, 1H), 8.14 (d, *J* = 3.6 Hz, 1H), 7.63 (d, *J* = 5.0 Hz, 1H), 7.52 (d, *J* = 5.0 Hz, 1H), 7.30 (d, *J* = 3.9 Hz, 1H), 7.26 (dd, *J* = 4.9, 3.9 Hz,

1H). ^{13}C {^1H} NMR (126 MHz, $CDCl_3$) δ 154.97, 146.52, 140.83, 136.55, 131.90, 130.55, 128.90, 128.15, 127.88, 127.26, 123.36, 120.52. Calc. for $C_{13}H_7N_3S$ $[M+H^+]^+$ (m/z): 301.9802, Found $[M+H^+]^+$: 301.9874.

Synthesis of 4,7-bis(4-hexylphenyl)-[1,2,5]thiadiazolo[3,4-c]pyridine (PBpyP). Following the general procedure for Suzuki coupling, PBpyP was synthesized by reacting to 4,7-dibromo-pyridal[2,1,3]thiadiazole (180 mg, 0.61 mmol) with 4-hexylphenylboronic acid (306 mg, 1.46 mmol). The product was obtained as a fluorescent yellow solid (210 mg, 0.46 mmol, 75% yield). ^1H NMR (300 MHz, $CDCl_3$) δ 8.83 (s, 1H), 8.55 (d, *J* = 8.2 Hz, 2H), 7.95 (d, *J* = 8.2 Hz, 2H), 7.42 (dd, *J* = 8.2, 4.3 Hz, 4H), 2.83 – 2.65 (m, 4H), 1.81 – 1.63 (m, 4H), 1.47 – 1.23 (m, 12H), 0.93 (td, *J* = 6.9, 2.2 Hz, 6H). ^{13}C {^1H} NMR (126 MHz, $CDCl_3$) δ 156.84, 152.29, 149.80, 145.76, 143.93, 142.53, 134.41, 131.95, 129.77, 129.02, 128.99, 128.78, 126.75, 35.96, 31.76, 31.41, 29.08, 22.64, 14.13. Calc. for $C_{29}H_{35}N_3S$ $[M+H^+]^+$ (m/z): 458.2618, Found $[M+H^+]^+$: 458.2612.

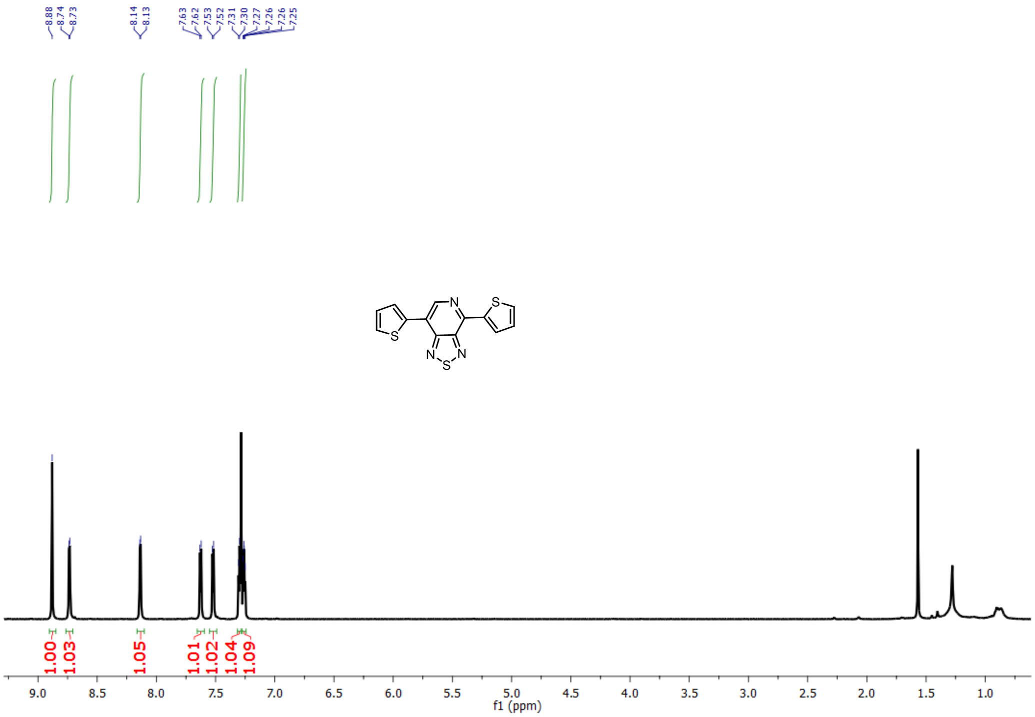

Figure S34. ^1H NMR spectrum of **TBpyT** (500 MHz, $CDCl_3$).

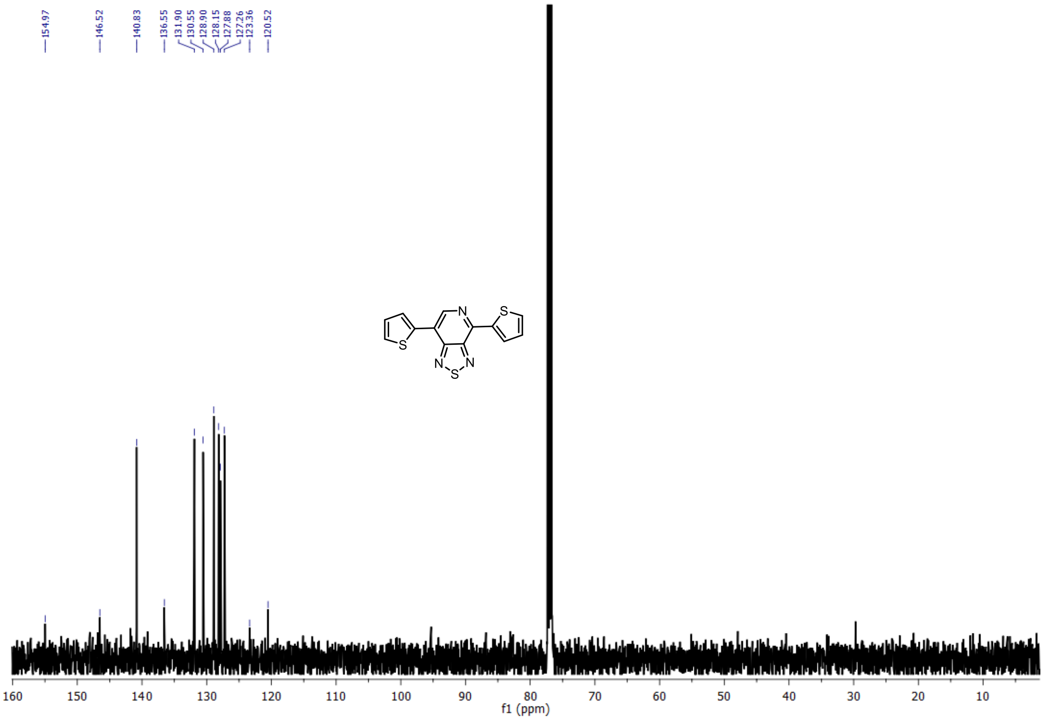

Figure S35. $^{13}C\ \{^{1}H\}$ NMR spectrum of **TBpyT** (126 MHz, $CDCl_3$).

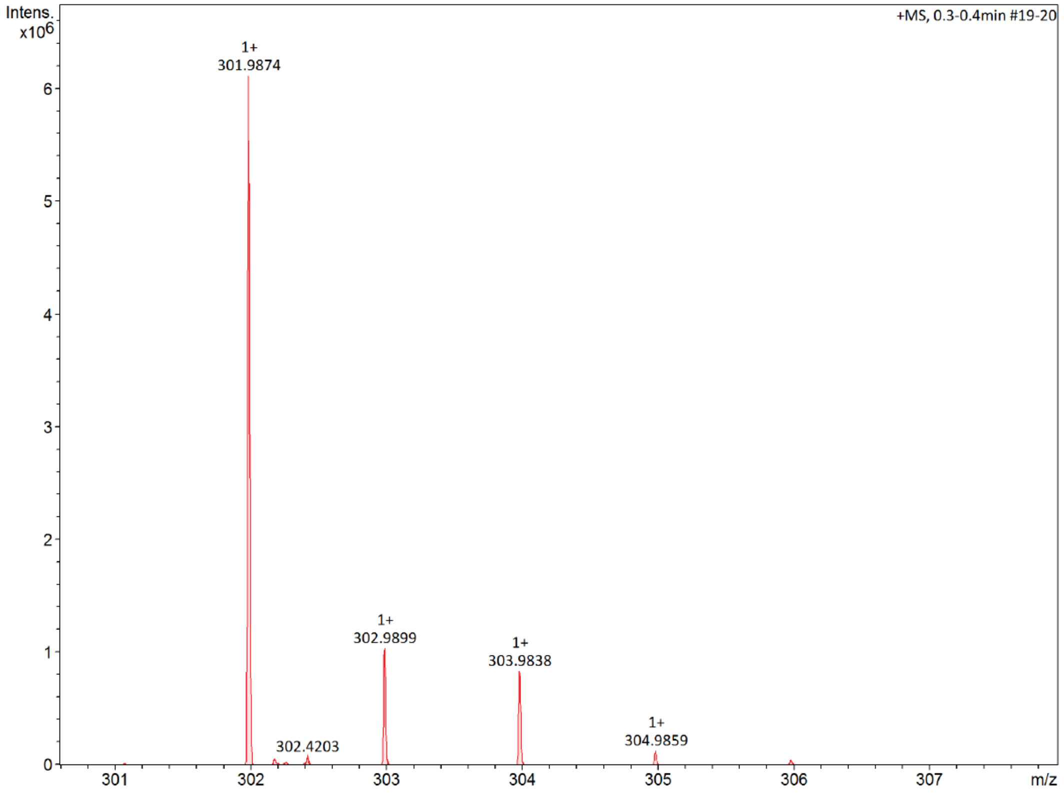

Figure S36. HRMS (m/z) spectrum of compound **TBpyT**.

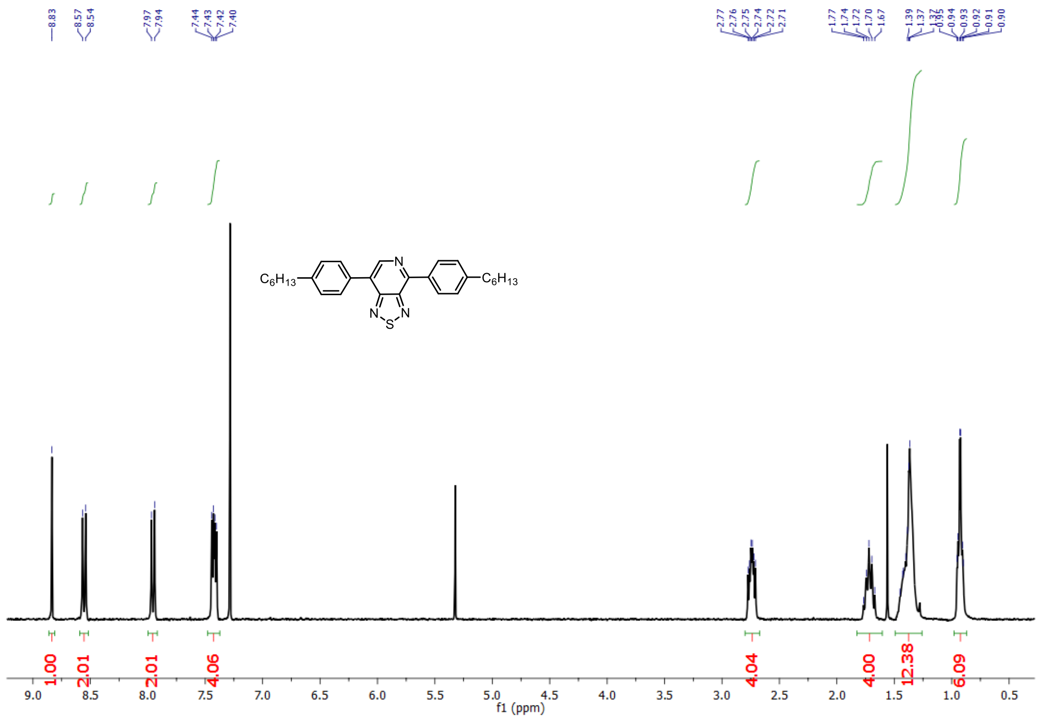

Figure S37. ^{1}H NMR spectrum of **PBpyP** (500 MHz, $CDCl_3$).

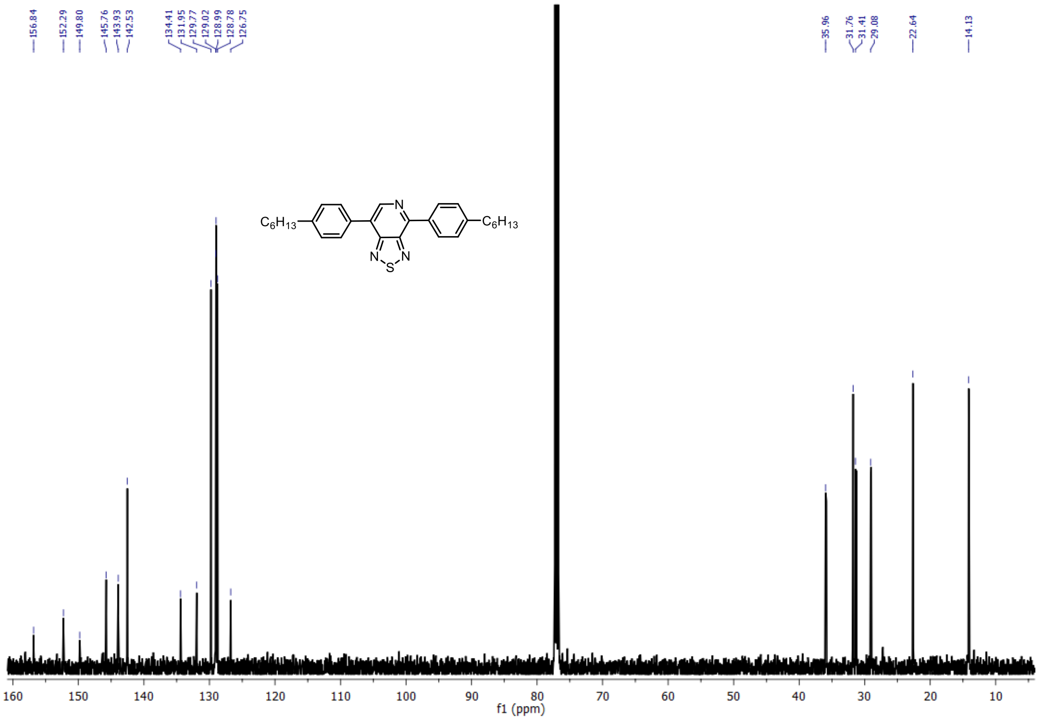

Figure S38. ^{13}C {^{1}H} NMR spectrum of **PBpyP** (126 MHz, $CDCl_3$).

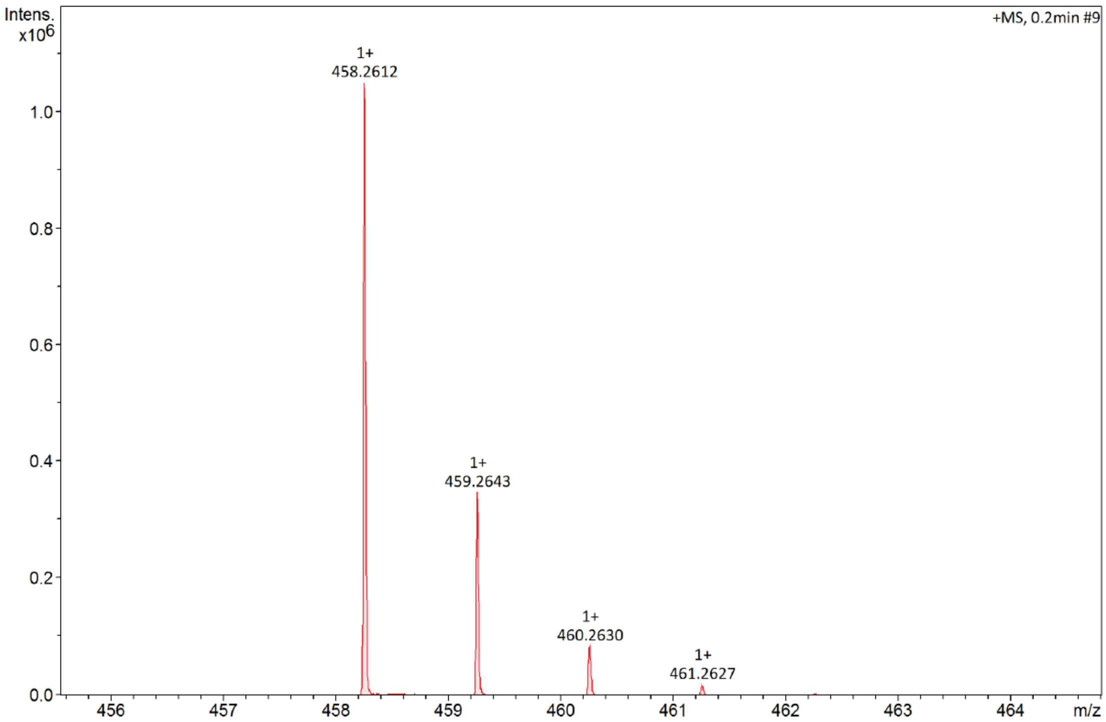

Figure S39. HRMS (m/z) spectrum of compound **PBpyP**.

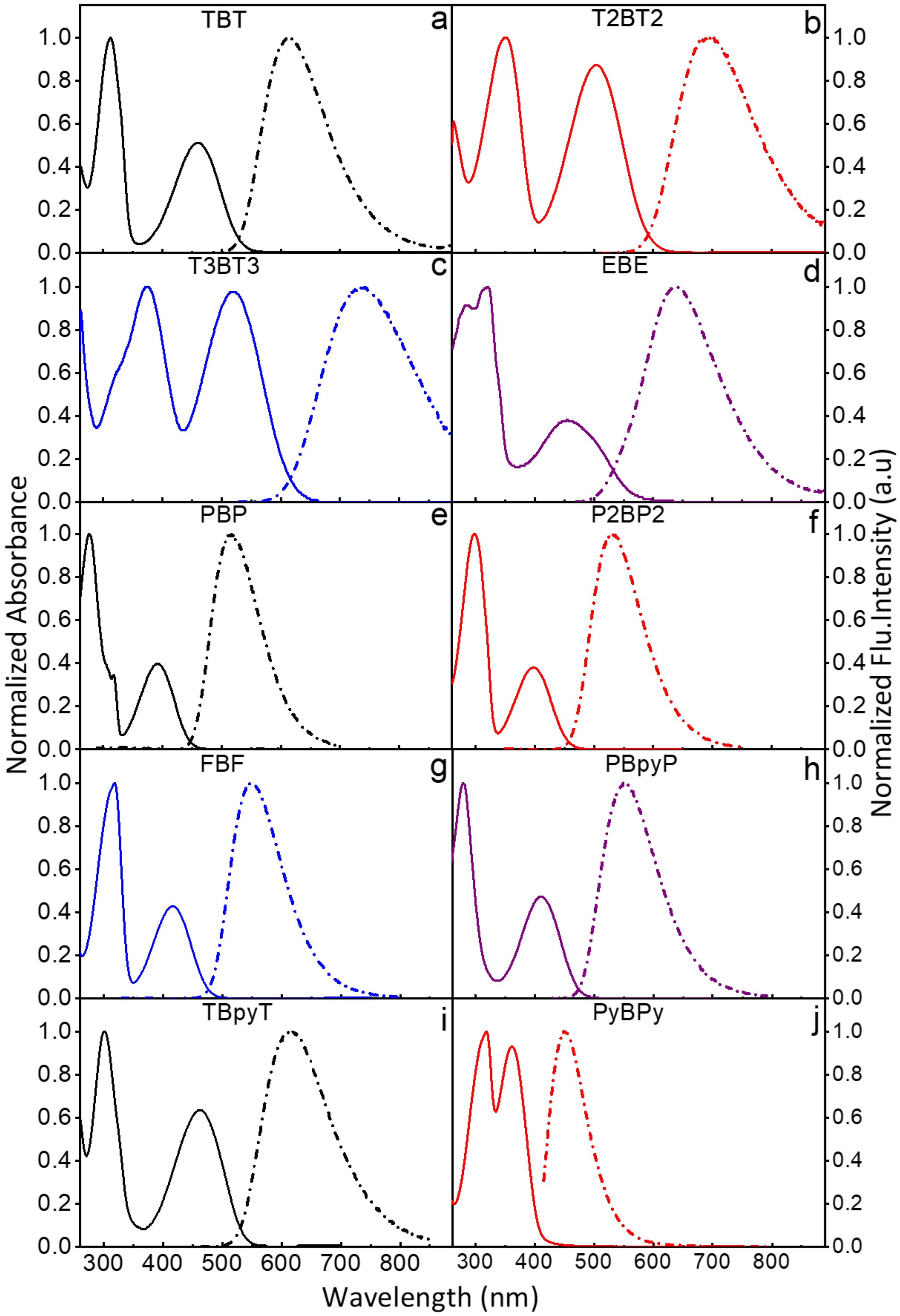

Figure S40. Absorption (solid lines) and fluorescence (dotted lines) spectra of DAD chromophores in DMF solution. (a) TBT (black, λ_{ex} = 450 nm); (b) T2BT2 (red, λ_{ex} = 500 nm); (c) T3BT3 (blue, λ_{ex} = 500 nm); (d) EBE (purple, λ_{ex} = 485 nm); (e) PBP (black, λ_{ex} = 395 nm); (F) P2BP2 (red, λ_{ex} = 395 nm f); (g) FBF (blue, λ_{ex} = 415 nm); (h) PBpyP (purple, λ_{ex} = 410 nm); (i) TBpyT (black, λ_{ex} = 460 nm); (j) PyBPy (red, λ_{ex} = 360 nm).

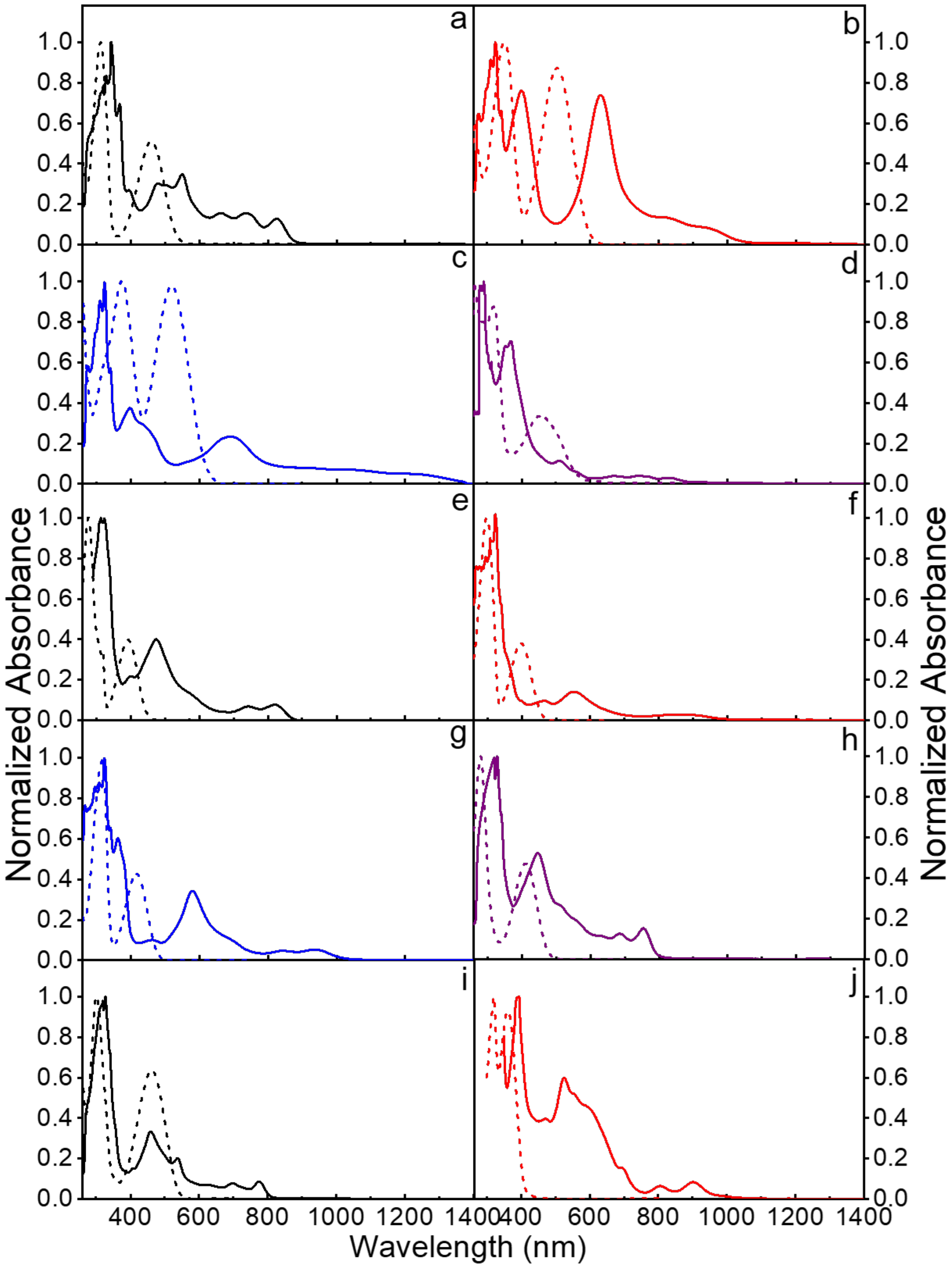

Figure S41. Normalized absorption spectra of DAD chromophores (dotted lines) and corresponding radical anions (solid lines) in DMF solution. (a) TBT; (b) T2BT2; (c) T3BT3; (d) EBE; (e) PBP; (F) P2BP2; (g) FBF; (h) PBpyP; (i) TBpyT; (j) PyBPy.

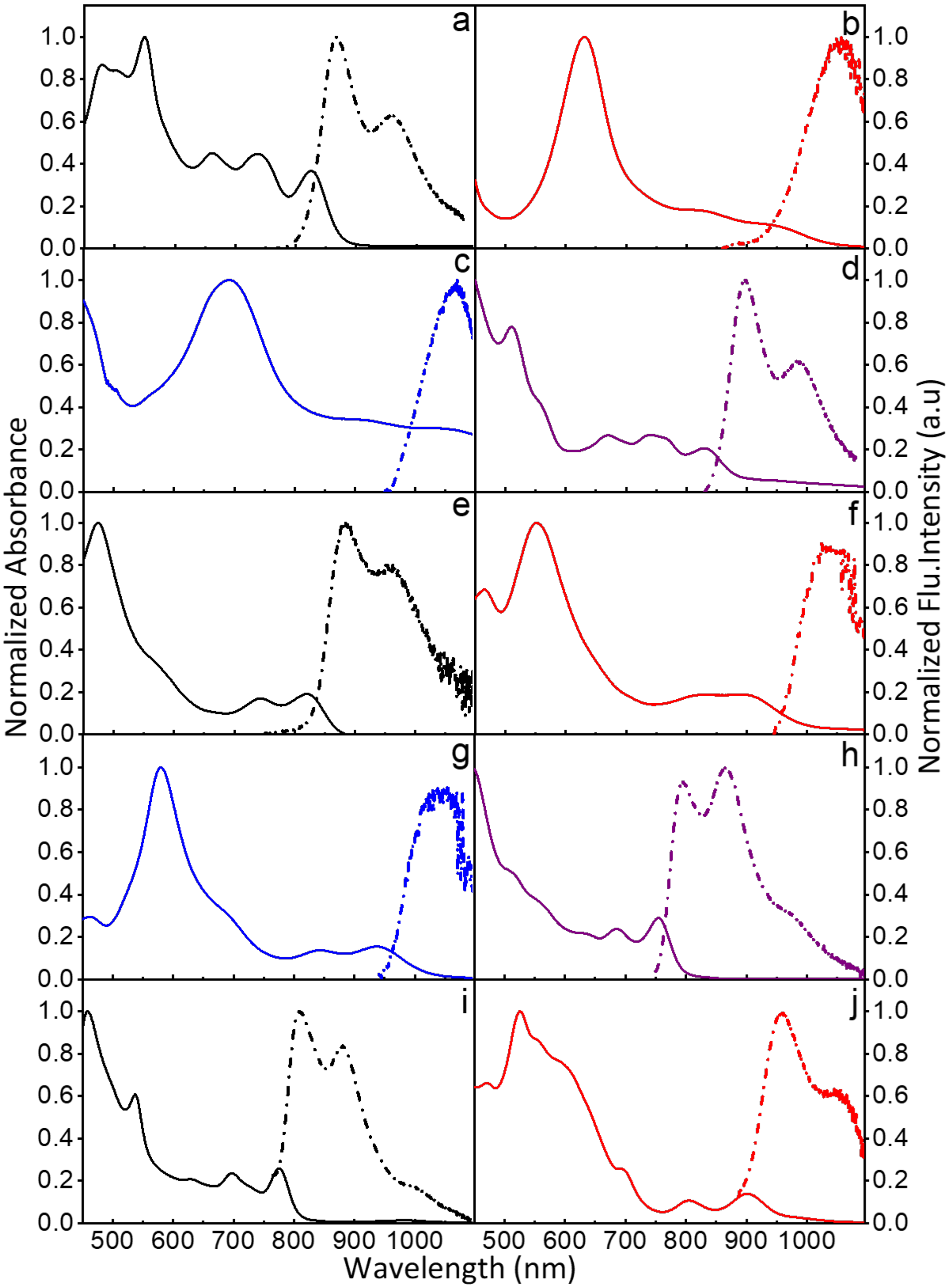

Figure S42. Absorption (black lines) and fluorescence (red lines) spectra of radical anions in DMF solution. (a) TBT (black, λ_{ex} = 742 nm); (b) T2BT2 (red, λ_{ex} = 830 nm); (c) T3BT3 (blue, λ_{ex} = 915 nm); (d) EBE (purple, λ_{ex} = 830 nm); (e) PBP (black, λ_{ex} = 815 nm); (f) P2BP2 (red, λ_{ex} = 820 nm); (g) FBF (blue, λ_{ex} = 840 nm); (h) PBpyP (purple, λ_{ex} = 750 nm); (i) TBpyT (black, λ_{ex} = 750 nm); (j) PyBPy (red, λ_{ex} = 875 nm).

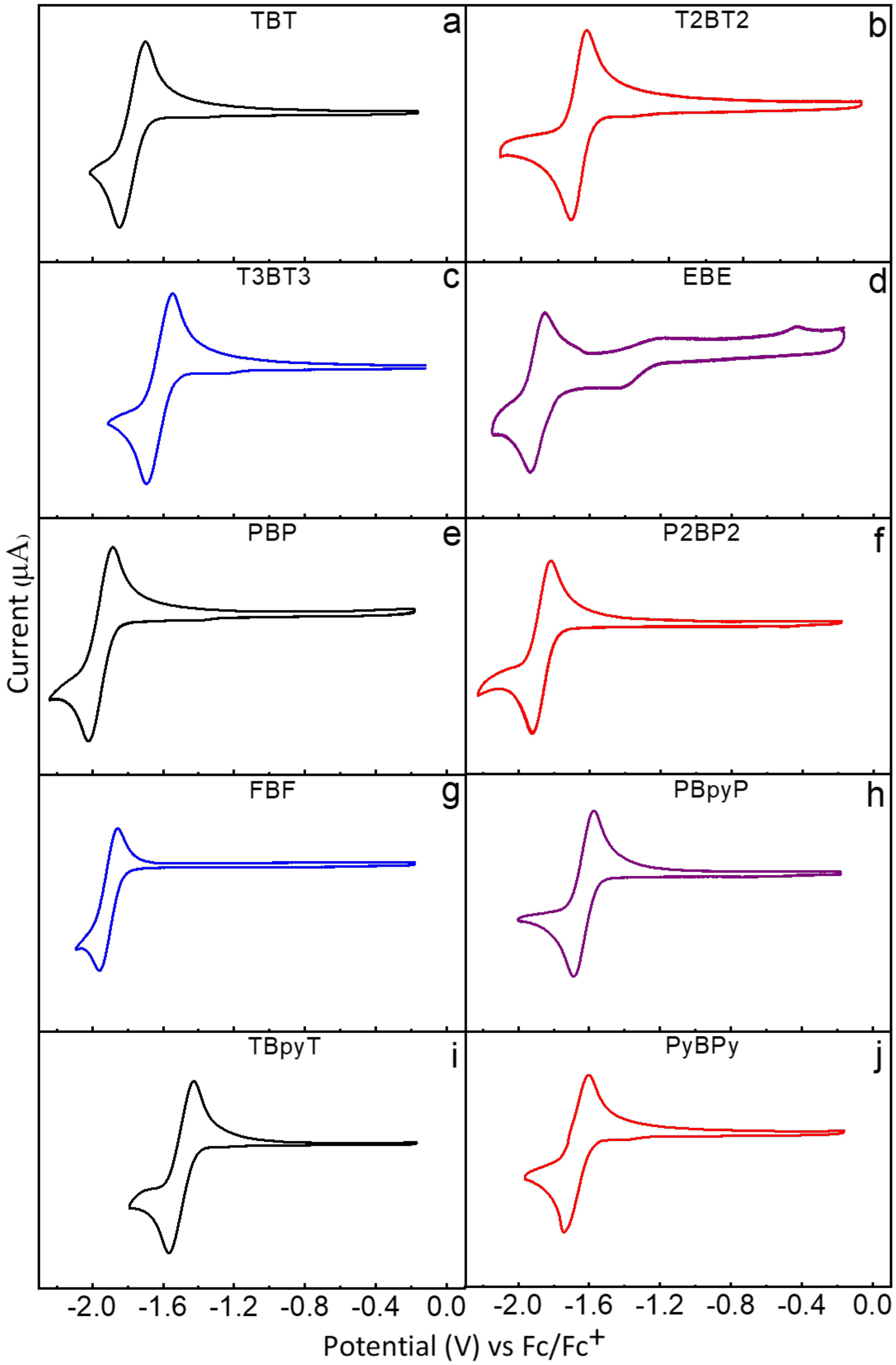

Figure S43: Cyclic voltammograms of the DAD compounds measured in DMF (0.1M NBu4PF6 as supporting electrolyte). Glassy carbon (surface area is 7.07 mm^2) was used as a working electrode, platinum wire as an auxiliary electrode and Ag/Ag+ as a reference electrode. Fc/Fc+ redox couple was used as an internal reference. Half wave potential for ferrocene/ferrocenium redox couple vs Ag/Ag+ which was ~0.163 V. (a) TBT (black); (b) T2BT2 (red); (c) T3BT3 (blue); (d) EBE (purple); (e) PBP (black); (f) P2BP2 (red); (g) FBF (blue); (h) PBpyP (perple); (i) TBpyT (black) and (j) PyBPy (red).

Table S1. Cyclic Voltammetry Data and HOMO and LUMO Energies

DAD	$E_{1/2}^{ox}$ / V vs SCE	$E_{1/2}^{red}$ / V vs SCE	$E_{1/2}^{ox}$ / V vs Fc/Fc^{+}	$E_{1/2}^{red}$ / V vs Fc/Fc^{+}	HOMO / eV	LUMO / eV	ΔE_g / eV	ΔE_{opt} / eV
**TBT**	1.21	-1.32	0.76	-1.77	-5.56	-3.03	2.53	2.37
**T2BT2**	0.95	-1.24	0.50	-1.69	-5.30	-3.11	2.19	2.10
**T3BT3**	0.89	-1.17	0.44	-1.62	-5.24	-3.18	2.06	1.98
**EBE**	-	-1.44	-	-1.89	-5.21	-2.91	2.30	-
**PBP**	1.56	-1.50	1.11	-1.95	-5.91	-2.85	3.06	2.77
**P2BP2**	1.55	-1.41	1.10	-1.86	-5.90	-2.94	2.96	2.71
**FBF**	1.36	-1.45	0.91,	-1.90	-5.71	-2.90	2.81	2.60
**PBpyP**	1.65	-1.17	1.20	-1.62	-6.0	-3.18	2.82	2.62
**TBpyT**	1.42	-1.03	0.97	-1.48	-5.77	-3.32	2.45	2.34
**PyBPy**	-	-1.22	-	-1.67	-6.18	-3.13	3.05	-

Scan rate = 100 mV/sec. Working electrode - Glassy carbon. Auxiliary electrode- Pt-wire. Reference electrode - Ag/Ag+. Internal standard- Fc/Fc+. Solvent:DMF, ΔE_{opt} were estimated from the intersection of the normalized absorption and emission spectra. All potentials referenced to Fc/Fc^{+}. HOMO = $-(4.8+E_{ox})$, LUMO = $-(4.8+E_{red})$, $\Delta E_g = E_{ox} - E_{red}$. ref. [6].

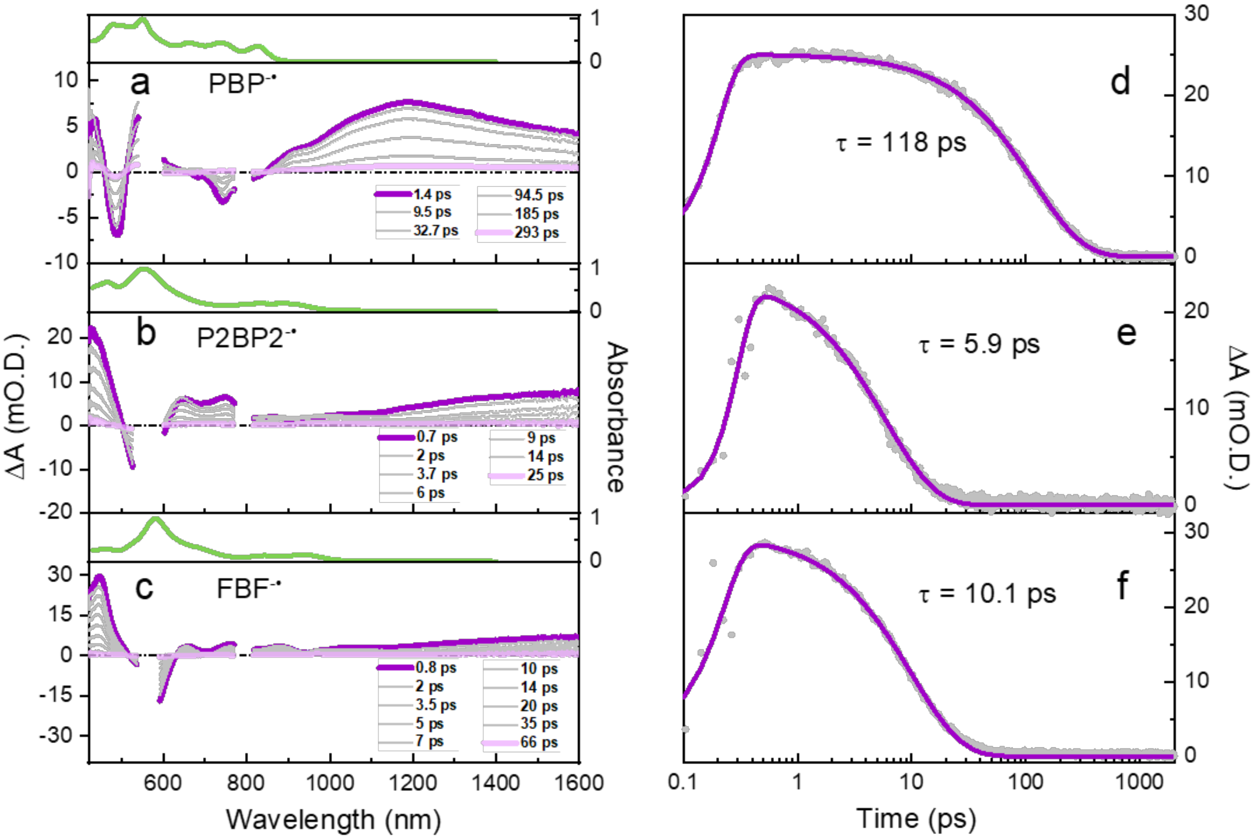

Figure S44. Femtosecond transient absorption spectra (left) and decay kinetics (right) of $DAD^{-\bullet}$ in DMF solution. Delay times for spectra are shown in the legends and lifetimes obtained from single exponential fits at 1191 nm ($PBP^{-\bullet}$), 428 nm ($P2BP2^{-\bullet}$), and 436 nm ($FBF^{-\bullet}$) . Ground state absorption spectra of the $DAD^{-\bullet}$ are shown in the plots above each of the sets of transient absorption spectra. (a,d) $PBP^{-\bullet}$, λ_{ex} = 570 nm. (b,e) $P2BP2^{-\bullet}$, λ_{ex} = 570 nm. (c,f) $FBF^{-\bullet}$, λ_{ex} = 570 nm.

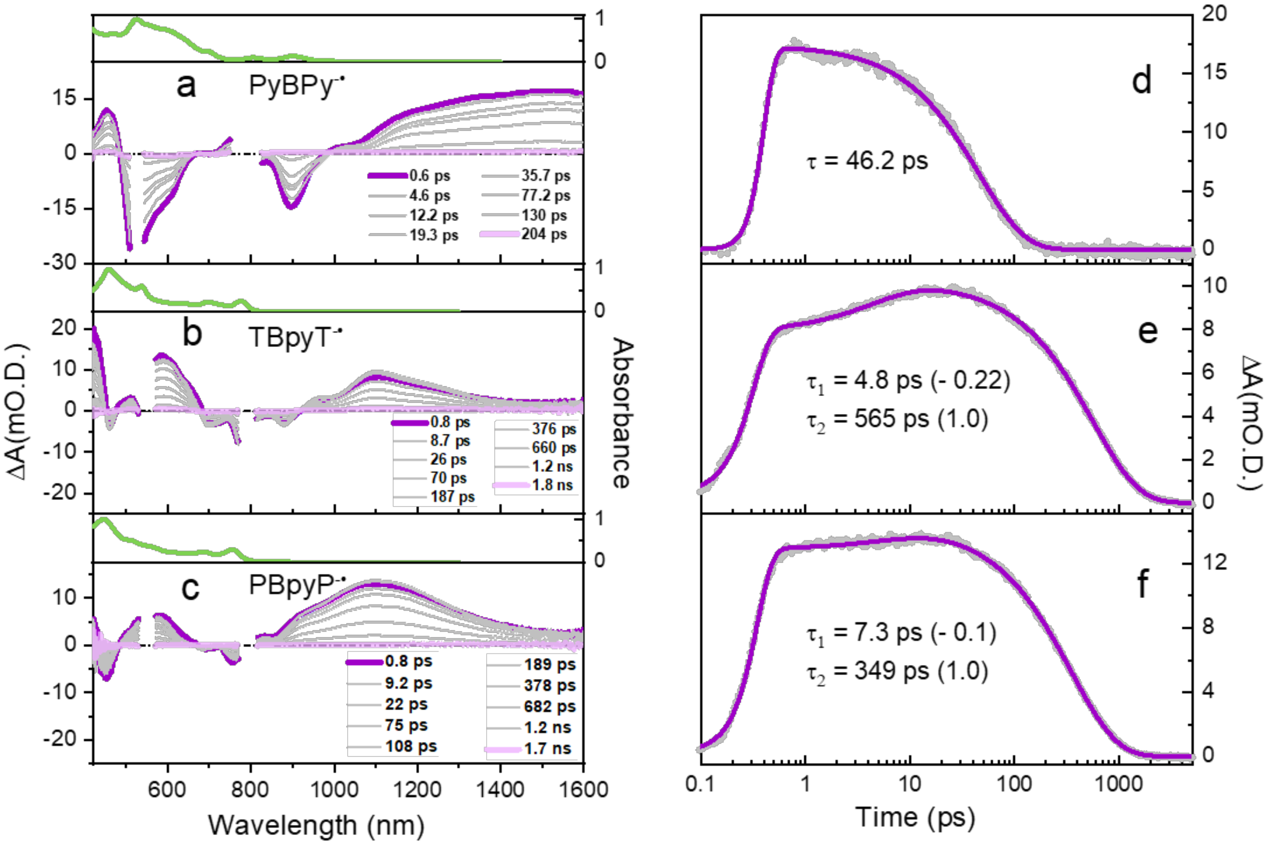

Figure S45. Femtosecond transient absorption spectra (left) and decay kinetics (right) of $DAD^{-•}$ in DMF solution. Delay times for spectra are shown in the legends and lifetimes obtained from single exponential fits at 1531 nm ($PyBPy^{-•}$), 1101 nm ($TBpyT^{-•}$), and 1091 nm ($PBpyP^{-•}$). Ground state absorption spectra of the $DAD^{-•}$ are shown in the plots above each of the sets of transient absorption spectra. (a,d) $PyBPy^{-•}$, λ_{ex} = 525 nm. (b,e) $TBpyT^{-•}$, λ_{ex} = 550 nm. (c,f) $PBpyP^{-•}$, λ_{ex} = 550 nm.

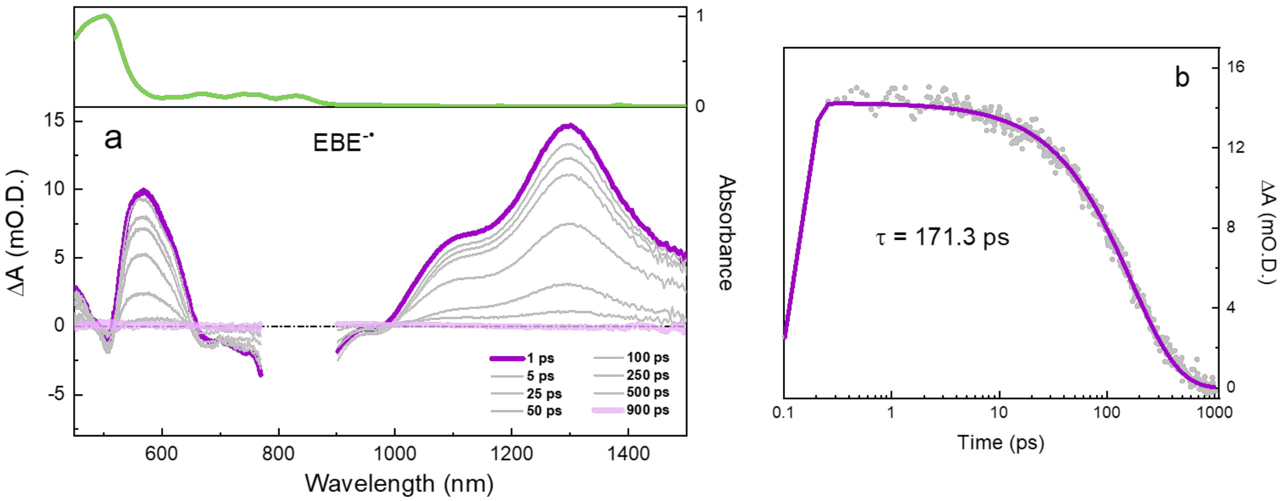

Figure S46. Femtosecond transient absorption spectra (left) and decay kinetics (right) of $EBE^{-\bullet}$ in DMF solution, λ_{ex} = 825 nm. Delay times for spectra are shown in the legends and lifetimes obtained from single exponential fits at 1305 nm. Ground state absorption spectra of $EBE^{-\bullet}$ is shown in the plots above each of the sets of transient absorption spectra.

Table S2: Photophysical Data in DMF Solution

Compounds	Neutrals (DAD)			Radical Anions ($DAD^{-\bullet}$)			
	$\lambda_{max, abs}$[a] (nm)	$\lambda_{max, em}$[b] (nm)	τ[c] (ns)	$\lambda_{max, abs}$[d] (nm)	$\lambda_{00, max}$[e] (nm)	ϕ[f]	τ[g] (ps)
**TBT**	310, 454	581	12.9	742, 826	867	0.0014	245
**T2BT2**	346, 503	654	6.4	832, 947	1053	-	-
**T3BT3**	376, 522	693	2.7	916, 1055	1066	-	-
**EBE**	324, 487	638	11.6	738, 831	895	0.00117	183
**PBP**	276, 393	517	7.2	740, 818	883	0.00096	129
**P2BP2**	298, 398	530	5.4	822, 896	1036	-	-
**FBF**	319, 415	550	6.5	842, 938	1047	-	-
**PBpyP**	280, 410	551	10.8	685, 753	795	0.0018	448
**TBpyT**	300, 460	615	11.2	685, 775	809	0.0024	642
**PyBPy**	320, 361	450	2.6	804, 903	957	0.00046	-

[a] Absorption maximum. [b] Fluorescence maximum. [c] Fluorescence lifetimes, excitation at 450 nm (TBT), 500 nm (T2BT2), 500 nm (T3BT3), 485 nm (EBE), 395 nm (PBP), 395 nm (P3BP2), 415 nm (FBF), 410 nm (PBpyP), 460 nm (TBpyT) and 360 nm (PyBPy). [d] Absorption maximum of radical anions. [e] Fluorescence maximum of radical anions. Excitation wavelength 742 nm ($TBT^{-\bullet}$), 830 nm ($T2BT2^{-\bullet}$), 915 nm ($T3BT3^{-\bullet}$), 830 nm ($EBE^{-\bullet}$), 815 nm ($PBP^{-\bullet}$), 820 nm ($P2BP2^{-\bullet}$), 840 nm ($FBF^{-\bullet}$), 750 nm ($PBpyP^{-\bullet}$) and 750 nm ($TBpyT^{-\bullet}$) and 875 nm ($PyBPy^{-\bullet}$). [f] Fluorescence quantum yield of radical anion measured in DMF (λ_{ex} = 710 nm) against IR-125 dye in ethanol as standard (ϕ = 0.132), ref. 5. [g] Fluorescence lifetimes of radical anions measured using TCSPC excited at 500 nm. Emission wavelengths: 880 nm ($TBT^{-\bullet}$), 870 nm ($PBP^{-\bullet}$), 870 nm ($EBE^{-\bullet}$), 750 nm ($PBpyP^{-\bullet}$) and 750 nm ($TBpyT^{-\bullet}$).

Table S3. Anion Radical Photophysical Parameters

$DAD^{-\bullet}$	ϕ [a]	τ / ps [b]	k_r / 10^6 s^{-1} [c]	k_{nr} / 10^9 s^{-1} [d]
**PyBPy**	0.00046	46	10.0	21.7
**PBP**	0.00096	118	8.1	8.5
**EBE**	0.00117	171	6.8	5.8
**TBT**	0.0014	245	5.7	4.1
**PBpyP**	0.0018	349	5.2	2.8
**TBpyT**	0.0024	565	4.3	1.8

[a] Fluorescence quantum yields measured in DMF, λ_{ex} = 710 nm relative to IR-125 dye in ethanol (λ_{ex} = 710 nm) as standard (ϕ = 0.132, ref 5). [b] Lifetime of excited state by transient absorption. [c] Radiative decay rate constant, $k_r = \phi / \tau$. [d] Non-radiative decay rate constant, $k_{nr} = (1 - \phi) / \tau$.

Energy Gap Law and Fitting of Fluorescence Data

Equation S1a is used to analyze the energy gap law for non-radiative decay.[7,8] In this expression S_m is the electron-vibration coupling constant, $\hbar\omega_m$ is the average of medium-frequency acceptor modes coupled to the excited/ground state transition, E_{00} is the energy of the relaxed excited state, $\Delta\nu_{0,1/2}$ is the half-width of the individual vibronic bands and β_0 is the vibronically induced electronic coupling. Under the assumption that all but the second term on the right-hand side of eq S1a are approximately constant within a narrow range of E_{00}, it can be simplified to give eq S2. A linear fit of the natural logarithm of the k_{nr} vs E_{00} gives a slope of -0.0016 cm (Figure 5a), which according to eq S2 is $-\gamma_0/\hbar\omega_m$.

The room-temperature emission spectra for three anion radicals ($TBT^{-\bullet}$, $PBP^{-\bullet}$, and $PyBPy^{-\bullet}$) were fitted using single-mode Franck-Condon expression (eq S3).[7,8] To see if the slope calculated using parameters obtained from the fits of the fluorescence spectra according to single-mode Franck-Condon expression (eq S3) match the slope from the linear fit of the energy gap law data, we performed spectral fitting for the three anion radicals that exhibit well-defined fluorescence spectra ($TBT^{-\bullet}$, $PBP^{-\bullet}$, and $PyBPy^{-\bullet}$). The fits are shown in Figs. S47 – S49 and the parameters obtained from the fits are summarized in Table S4. Using the parameters obtained from these fits ($\hbar\omega_m$ and S_m, Table S4), γ_0 was calculated for these radicals using eq 1c and γ_0 is in turn used to calculate $\gamma_0/\hbar\omega_m$. As can be seen from Table S4 (last column), the calculated slopes are in reasonable agreement with the slope obtained from the linear fit of the experimental ln k_{nr} vs E_{00} data.

$$ln\, k_{nr} = \ln\beta_o - \frac{\gamma_o E_{00}}{\hbar\omega_m} - S_m + (\gamma_o + 1)^2 \frac{(\Delta\bar{\nu}_{0,1/2}/\hbar\omega_m)^2}{16\, ln\, 2} - 0.5\, ln\left(\frac{\hbar\omega_m E_{00}}{1000\ \text{cm}^{-1}}\right) \quad \text{(S1a)}$$

$$\beta_o = |C_k|^2 \omega_k \left(\frac{\sqrt{\pi/2}}{1000\ \text{cm}^{-1}}\right) \quad \text{(S1b)}$$

$$\gamma_o = \ln\left(\frac{E_{00}}{\hbar\omega_m S_m}\right) - 1 \quad \text{(S1c)}$$

$$ln\, k_{nr} = a - \left(\frac{\gamma_o}{\hbar\omega_m}\right) E_{00} \quad \text{(S2)}$$

$$I(\bar{\nu}) = \sum_{\nu_m=0}^{5} \left\{ \left(\frac{E_{00} - \nu_m\hbar\omega_m}{E_{00}}\right)^3 \frac{(S_m)^{\nu_m}}{\nu_m!} exp\left[-4\, ln\, 2\left(\frac{\bar{\nu} - E_{00} + \nu_m\hbar\omega_m}{\Delta\bar{\nu}_{0,1/2}}\right)^2\right]\right\} \quad \text{(S3)}$$

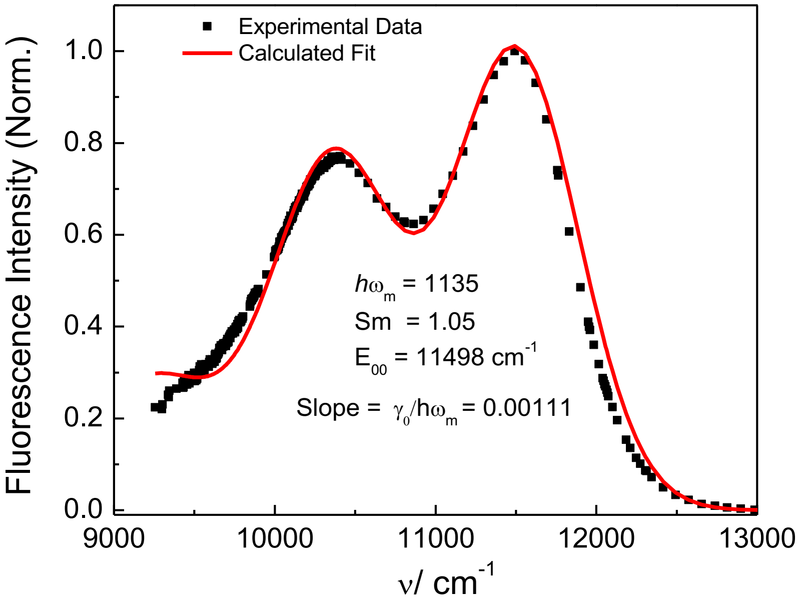

Figure S47. Fit of the fluorescence emission of $TBT^{-\bullet}$ in DMF by using eq. S1.

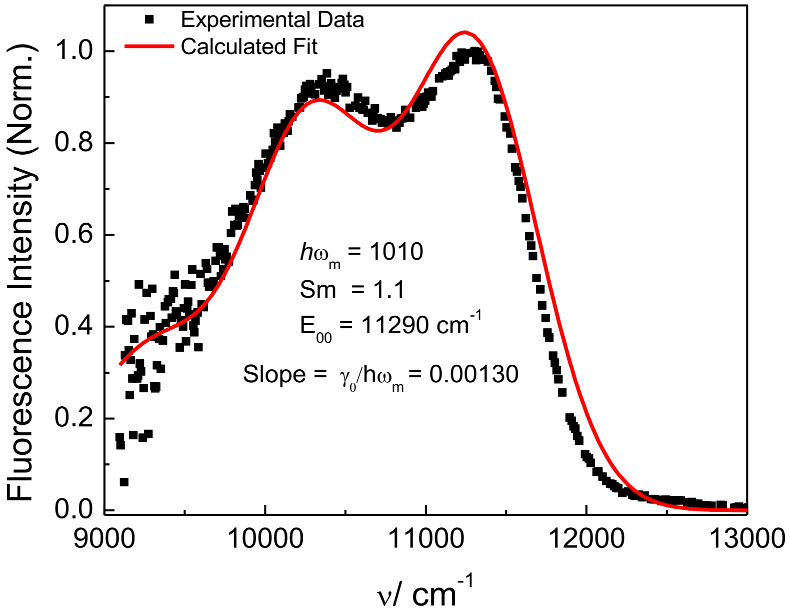

Figure S48. Fit of the fluorescence emission of $PBP^{-\bullet}$ in DMF by using eq. S1.

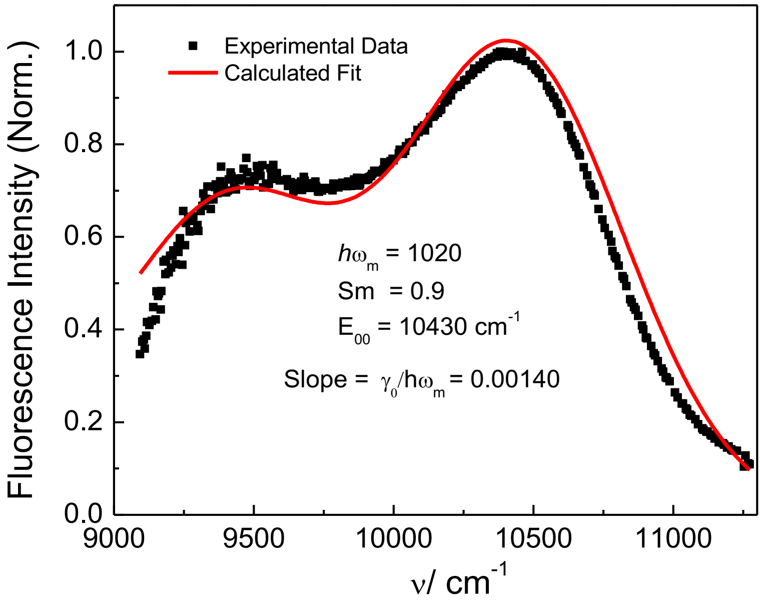

Figure S49. Fit of the fluorescence emission of $PyBPy^{-\bullet}$ in DMF by using eq. S1.

Table S4. Fluorescence Spectral Fit Parameters

Anion Radical	E_{00} / cm^{-1}	$\hbar\omega_m$ / cm^{-1}	S_m	$\gamma_0 = \ln(E_{00}/\hbar\omega_m S_m) - 1$	$\gamma_0/\hbar\omega_m$ / cm
**TBT**	11498	1135	1.05	1.27	0.0011
**PBP**	11290	1010	1.1	1.32	0.0013
**PyBPy**	10430	1020	0.9	1.43	0.0014

Photocatalysis Experiments

Preparation of Calibration Standards. A series of calibration standards was prepared by mixing known concentrations of **1** and **2**, with a fixed concentration of naphthalene (7.0 mM) in DMF. Naphthalene was used as an internal standard (**Std.**). The ratio of concentrations of 1: 2: Std. in calibration solutions includes 0.25:0.25:7, 0.5:0.5:7, 0.75:0.75:7, and 1:1:7 (all concentrations were at mM levels). All solutions were filtered through a 0.45 µm PTFE syringe filter before HPLC injection.

Construction of Calibration Plots. For each calibration solution, the peak areas corresponding to **1**, **2**, and **Std.** were recorded. Response factors of **1** and **2** relative to the **Std.** in each calibration solution were calculated as the ratios of their corresponding areas to the Std. area. (7.00 mM). The following equations were used:

$$R_1 = \frac{A_1}{A_{Std.}} \quad \text{and} \quad R_2 = \frac{A_2}{A_{Std.}}$$

where A_1, A_2, and $A_{Std.}$ are the peak areas of the starting material, product, and internal standard, respectively, and R_1 and R_2 are the response factors of **1** and **2** in each calibration solution vs. the Std.

Then, calibration plots of R_1 vs. C_1 and R_2 vs. C_2 were constructed (here C_1 and C_2 are the concentrations). Linear regression was performed with the intercept set to zero. The slope of each plot corresponds to the relative response factor (RRF) for **1** and **2** relative to Std. (of known conc.) respectively.

Analysis of Reaction Samples. From the sample HPLC chromatograms, the peak areas were used to calculate the R_1 and R_2 in the sample using the following equations.

$$R_{1\text{ in sample}} = \frac{A_{1\text{ in sample}}}{A_{Std.in\ sample}} \quad \text{and} \quad R_{2\text{ in sample}} = \frac{A_{2\text{ in sample}}}{A_{Std.in\ sample}}$$

Using these values, the concentration of **1** and **2** in the sample (after a specific time interval) was determined

$$[\mathbf{1}] = \frac{R_{1\text{ in sample}}}{RRF_1} \quad \text{and} \quad [\mathbf{2}] = \frac{R_{2\text{ in sample}}}{RRF_2}$$

After that, the conversion of **1** and formation of **2** were calculated based on the initial concentration of **1** in the reaction mixture, denoted as $[\mathbf{1}]_0$, using the following expressions:

$$\%\text{ conversion} = \frac{[\mathbf{1}]_0 - [\mathbf{1}]}{[\mathbf{1}]_0} \times 100, \quad \text{and } \%\text{ Yield} = \frac{[\mathbf{2}]}{[\mathbf{1}]_0} \times 100$$

Analysis of Photoreduction of 4-Bromoacetophenone with TBpyT Photocatalyst

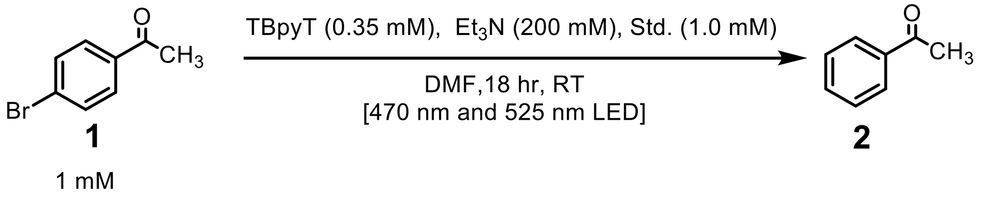

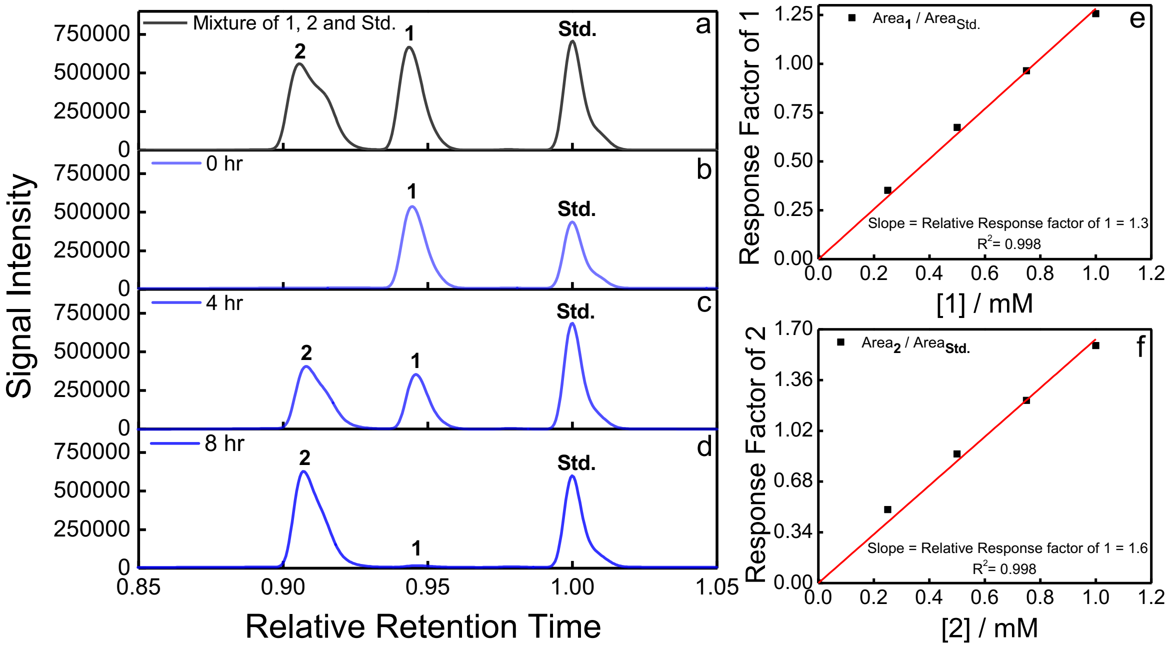

Figure S50. HPLC Analysis of photoreduction of **1** by **TBpyT**$^{-\bullet}$. Plots in panels a - d show the HPLC chromatograms. HPLC analysis was carried out with UV detection at 242 nm. The peaks are labeled to identify the components 4-bromoacetophenone (**1**), acetophenone (**2**) and internal standard naphthalene (**Std.**). The area ratio corresponds to the integrated HPLC peak of the component relative to the naphthalene internal standard. (a) Standard solution mixture of **1** (1 mM), **2** (1 mM) and **Std**. (7 mM). (b) Reaction mixture at t = 0 hr. (c) Analysis of reaction mixture at t = 4 hr. (d) Analysis of reaction mixture at t = 8 hr. (e) Calibration curve for determining the concentration of **1** in the reaction mass at different time intervals. (f) Calibration curve for determining the concentration of **2** in the reaction mass at different time intervals.

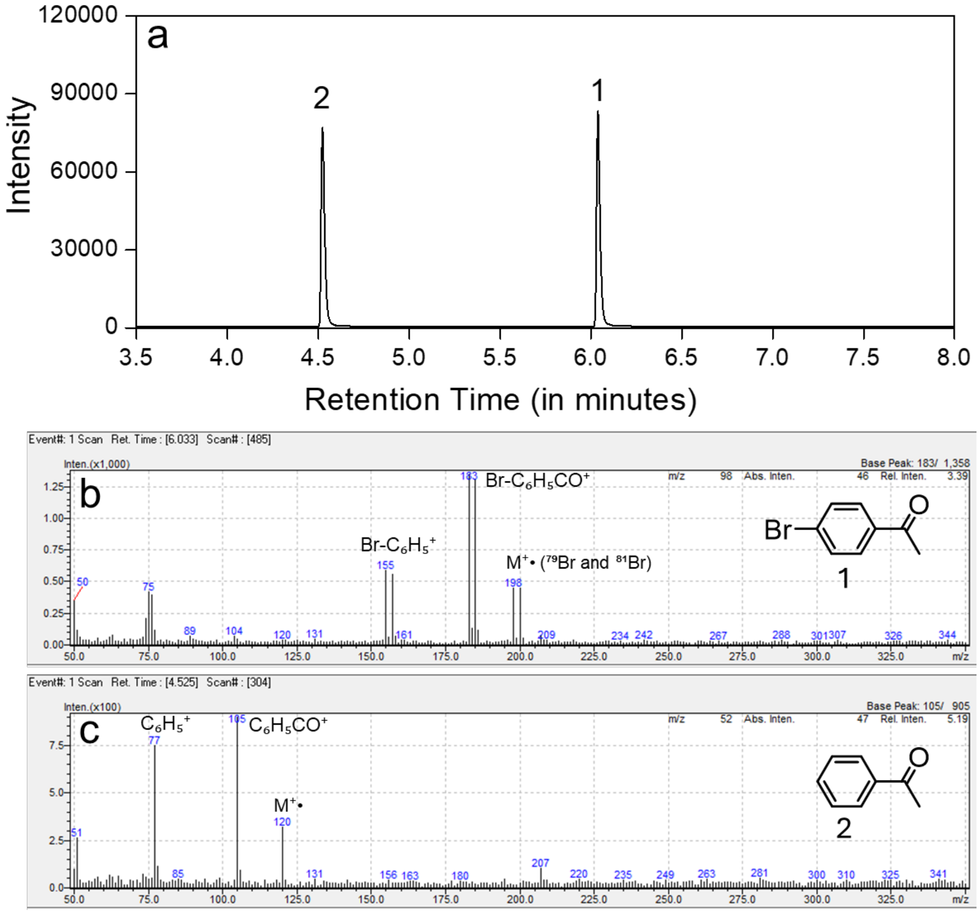

Figure S51. Gas chromatography-mass spectroscopy analysis of standard solution of **1** and **2** at equimolar concentration in acetone solution. (a) Gas chromatogram for mixture of 4-bromoacetophenone (**1**) and acetophenone (**2**). (b) Mass spectrum at retention time corresponding to **1**; parent peak and fragmentation pattern consistent with 4-bromoacetophenone. (c) Mass spectrum at retention time corresponding to **2**; parent peak and fragmentation pattern consistent with compound acetophenone.

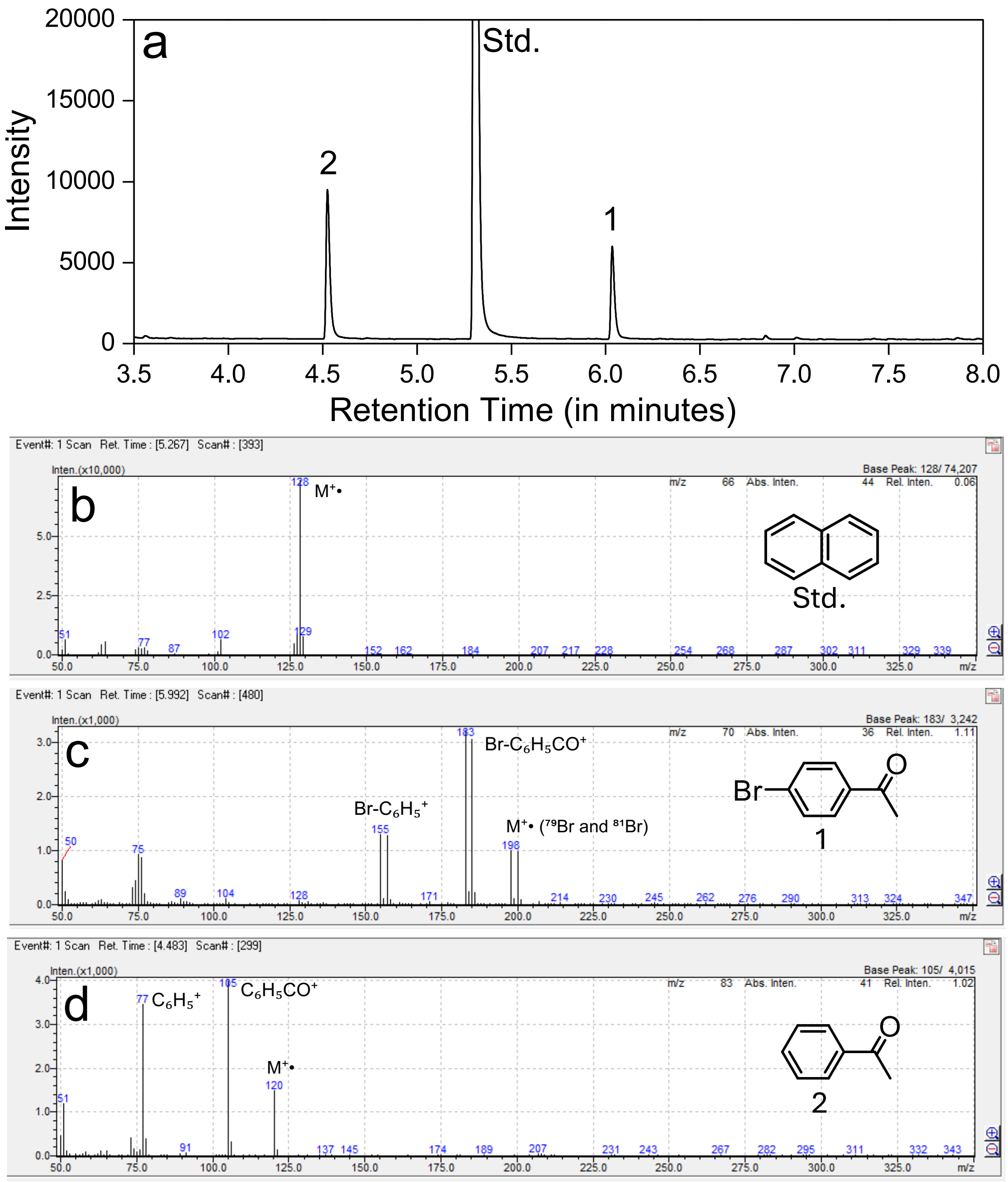

Figure S52. Gas chromatographic analysis of photocatalysis reaction mixture (see reaction scheme in Fig. S50 for components). (a) Gas chromatogram for reaction mixture at 4 hr irradiation time. (b) Mass spectrum at retention time corresponding to **Std**; parent peak and fragmentation pattern consistent with naphthalene. (c) Mass spectrum at retention time corresponding to **1**; parent peak and fragmentation pattern consistent with compound acetophenone. (d) Mass spectrum at retention time corresponding to **2**; parent peak and fragmentation pattern consistent with compound acetophenone.

Electrochemistry of 4-Bromoacetophenone and 4-Bromoanisole

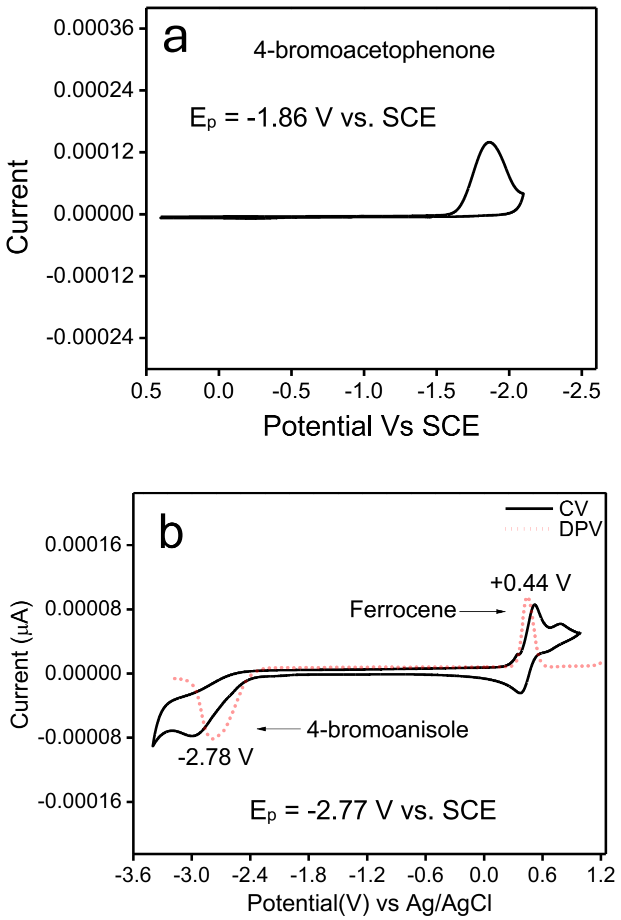

Figure S53. Cyclic voltammograms of (a) 4-bromoacetophenone and (b) 4-bromoanisole measured in DMF solution. Red plot in (b) shows differential pulse voltammagram (DPV). Conditions for (a): 0.1 M of NBu_4PF_6 electrolyte, glassy carbon (surface area 7.07 mm^2) working electrode, Pt-wire auxiliary electrode, and Ag/$AgNO_3$ acetonitrile quasi-reference electrode. Ferrocene (Fc/Fc^+) was used as internal standard (added after the CV that is shown), and the potential scale was converted to SCE by using E(Fc/Fc^+) = +0.45 V vs. SCE. Conditions for (b): 0.1 M of NBu_4PF_6 electrolyte, glassy carbon (surface area 7.07 mm^2) working electrode, Pt-wire auxiliary electrode, and Ag/AgCl in acetonitrile quasi-reference electrode. Ferrocene (Fc/Fc^+) was used as internal standard (CV wave shown). The potential axis is shown relative to the Ag/AgCl quasi-reference electrode, and the potential of the 4-bromoanisole is converted to SCE by using E(Fc/Fc^+) = +0.45 V vs. SCE.

Quenching Studies of TbpyT$^{-•}$ by 4-Bromoanisole

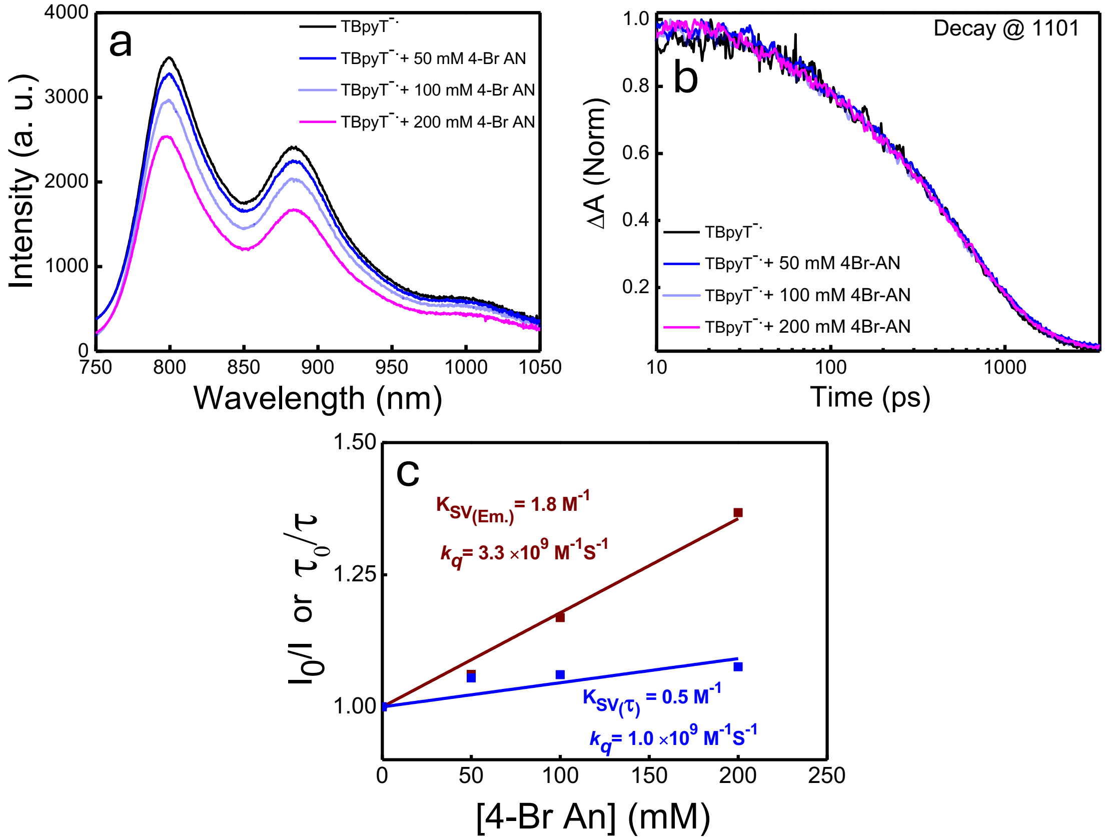

d

Compound	τ_1 (ps)	τ_2 (ps)
TBpyT$^{-•}$	3.0	540
TBpyT$^{-•}$ + 50 mM 4Br-AN	2.5	512
TBpyT$^{-•}$ + 100 mM 4Br-AN	2.5	509
TBpyT$^{-•}$ + 200 mM 4Br-AN	2.7	502

Figure S54. Quenching of TBpyT$^{-•}$ (0.1 mM) with 4-bromoanisole (4-Br AN, 0 - 200 mM) in deoxygenated DMF solution. (a) Steady-state fluorescence intensity quenching; samples were excited at 725 nm. (b) Excited state lifetime quenching measured by transient absorption; samples were excited at 550 nm, and the exited state absorption decay was monitored at 1101 nm. (c) Stern-Volmer plots for the lifetime quenching (blue squares) and steady-state emission quenching (maroon squares) of TBpyT$^{-•}$ by 4-Br AN. (d) Table of time constants for transient absorption decay fits at 1101nm in the quenching of TBpyT$^{-•}$ by 4-bromoanisole (4Br-AN), λ_{ex} = 550 nm.

Analysis of Photoreduction of 4-Bromoanisole with TBpyT Photocatalyst

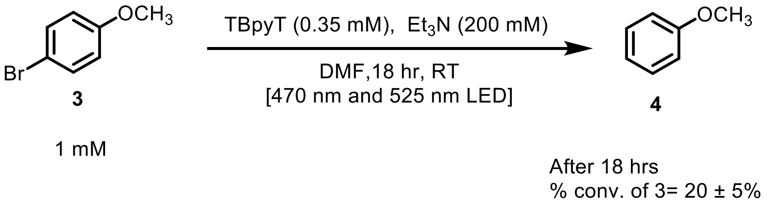

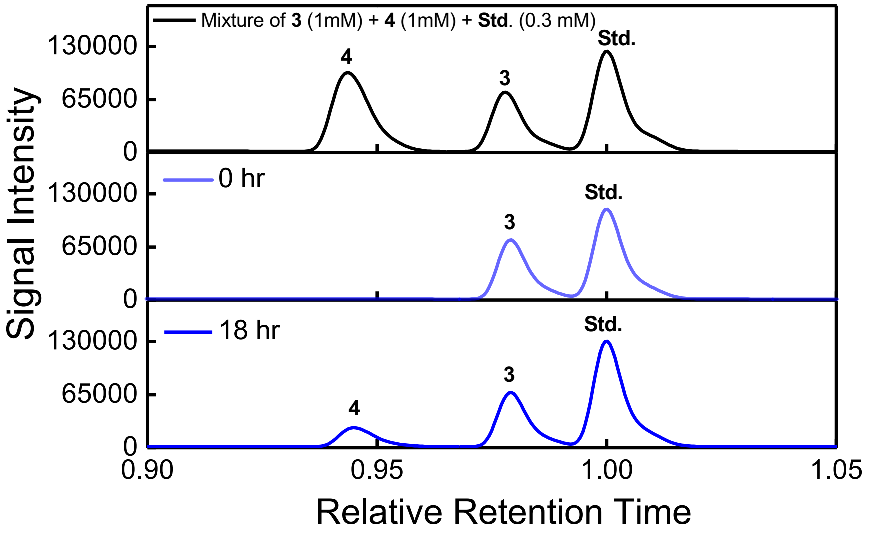

Figure S55. HPLC Analysis of photoreduction of **3** by **TBpyT**$^{-\bullet}$, the plots show the HPLC traces as a function of relative retention time. HPLC analysis was carried out with UV detection at 272 nm. The peaks are labeled to identify the components **3** and **4** are 4-bromoanisole and anisole, respectively, naphthalene was used as an internal standard (**Std**.). Reaction conditions:1.0 mM of **3**, 0.35 mM of TBpyT, 200.0 mM Et_3N and 1.0 mM of naphthalene (**Std**.) in a 1.0 mL mixture of DMF was irradiated simultaneously by blue (470 nm) and green (525 nm) LEDs for 18 hours.

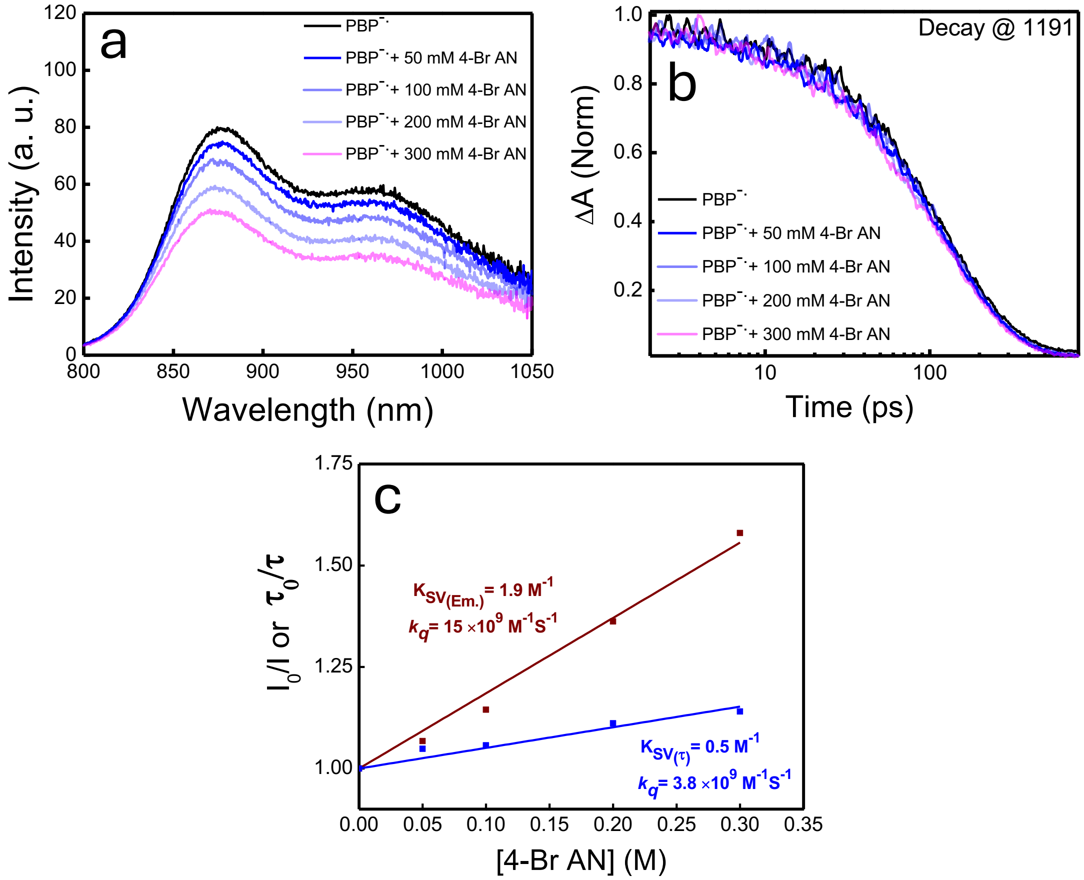

d

Compound	τ_1 (ps)
$PBP^{-\bullet}$	130
$PBP^{-\bullet}$ + 50 mM 4Br-AN	124
$PBP^{-\bullet}$ + 100 mM 4Br-AN	123
$PBP^{-\bullet}$ + 200 mM 4Br-AN	117
$PBP^{-\bullet}$ + 300 mM 4Br-AN	114

Figure S56. (a) Steady-state fluorescence intensity quenching and (b) exited state quenching of $PBP^{-\bullet}$ (0.1 mM) with 4-bromoanisole (4-Br AN, 0 - 300 mM) in deoxygenated DMF solution. Samples for lifetime studies were excited at 550 nm, and the exited state decay was monitored at 1101 nm. Samples for steady-state quenching were excited at 725 nm. (c) Stern-Volmer plots for the lifetime quenching (blue squares) and steady-state emission quenching (maroon squares) of $PBP^{-\bullet}$ by 4-Br AN. (d) Table of time constants for a decay fit at 1101nm in the quenching of $PBP^{-\bullet}$ by 4-Bromoanisole (4Br-AN), λ_{ex} = 550 nm.

Photocatalysis Reaction of 4-bromoanisole with PBP

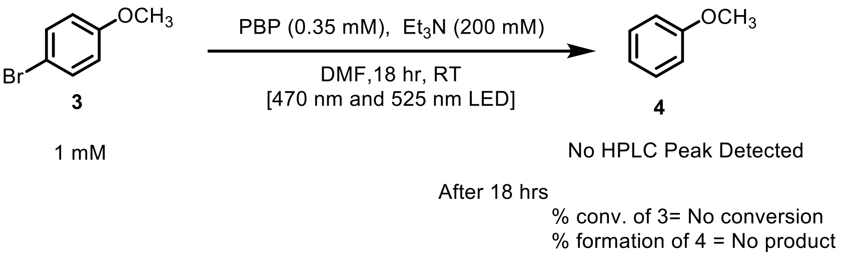

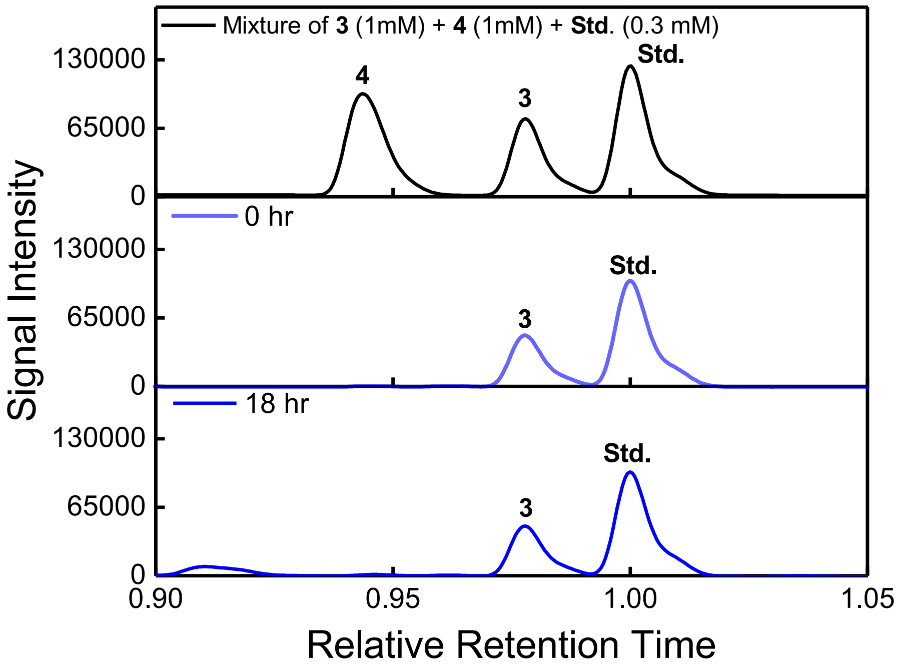

Figure S57. HPLC Analysis of photoreduction of **3** by **PBP**$^{-\bullet}$, the plots show the HPLC traces as a function of relative retention time. HPLC analysis was carried out with UV detection at 272 nm. The peaks are labeled to identify the components **3** and **4** are 4-bromoanisole and anisole, respectively, naphthalene was used as an internal standard (Std.). Reaction onditions:1.0 mM of **3**, 0.35 mM of PBP, 200.0 mM Et_3N and 1.0 mM of naphthalene (Std.) in a 1.0 ml mixture of DMF was irradiated simultaneously by a blue LED (470 nm) and a green LED (525 nm) for 18 hours.

References

1. https://www.coherent.com/lasers/laser/astrella-ultrafast-tisapphire-amplifier .

2. https://www.coherent.com/lasers/laser/opera-solo-ultrafast-optical-parametric-amplifier .

3. https://ultrafastsystems.com/products/helios-fire .

4. Hossain, M. M.; Ivanov, M. V.; Wang, D.; Reid, S. A.; Rathore, R. Spreading Electron Density Thin: Increasing the Chromophore Size in Polyaromatic Wires Decreases Interchromophoric Electronic Coupling. *J. Phys. Chem. C* **2018**, *122*, 17668– 17675, 10.1021/acs.jpcc.8b05299.

5. Rurack, K.; Spieles, M. Fluorescence Quantum Yields of a Series of Red and Near-Infrared Dyes Emitting at 600−1000 nm. *Anal. Chem.* **2011**, *83*, 1232−1242, 10.1021/ac101329h.

6. Cardona, C. M.; Li, W.; Kaifer, A. E.; Stockdale, D.; Bazan, G. C. Electrochemical Considerations for Determining Absolute Frontier Orbital Energy Levels of Conjugated Polymers for Solar Cell Applications. *Adv. Mater.* **2011**, *23*, 2367– 2371, 10.1002/adma.201004554.

7. Kober, E. M.; Caspar, J. V.; Lumpkin, R. S.; Meyer, T. J., Application of the Energy Gap Law to Excited-State Decay of Osmium(II)-Polypyridine Complexes: Calculation of Relative Nonradiative Decay Rates from Emission Spectral Profiles. *J. Phys. Chem.* **1986,** *90*, 3722-3734, 10.1021/j100407a046.

8. Whittle, C. E.; Weinstein, J. A.; George, M. W.; Schanze, K. S., Photophysics of Diimine Platinum(II) Bis-Acetylide Complexes. *Inorg. Chem.* **2001,** *40*, 4053-4062, 10.1021/ic0102182.
