# Supplementary material for: From Fundamental Photophysics to Photocatalysis: Energy Gap Law Analysis of Anion Radical Excited States
Source: ACS Cent Sci. 2026 Jun 15;12(6):856–66. doi: 10.1021/acscentsci.6c00092 (PMC13306596; doi:10.1021/acscentsci.6c00092)
Supplement: Supplementary file 2 [file oc6c00092_si_002.pdf]

DFT Supporting Information

From Fundamental Photophysics to Photocatalysis: Energy Gap Law Analysis of Anion Radical Excited States

Naresh Duvva[†], Tanjila Islam[†], Silvano R. Valandro[†], Habtom B. Gobeze[†], Abul Mansur Muhammed Fahim[†], Xiaodan Wang[†], Trang Le[†], Aimée L. Tomlinson[‡*] and Kirk S. Schanze[†*]

[†]Department of Chemistry, the University of Texas at San Antonio, One UTSA Circle, San Antonio, Texas 78249, United States.

[‡]Department of Chemistry and Biochemistry, University of North Georgia, Dahlonega, Georgia, 30597, United States.

*Corresponding author E-mails: kirk.schanze@utsa.edu, aimee.tomlinson@ung.edu

Contents

Computational Details ... 2
Benchmarking Functionals & Basis Sets ... 2
Solvent criteria ... 2
Density Functional Theory (DFT) ... 2
Series 1 ... 2
Series 2 ... 2
Time Dependent DFT ... 3
Series 1 ... 3
Series 2 ... 3
Series 1 ... 3
Benchmarking Plots ... 3
UV-Vis Experimental vs DFT Overlays + FMO Density Plots ... 4
TBT ... 4
T_2BT_2 ... 5
T_3BT_3 ... 6
TBpyT ... 7
EBE ... 8
Cartesian Coordinates ... 9
TBT ... 9
T_2BT_2 ... 9
T_3BT_3 ... 10
TBpyT ... 11
EBE ... 12
Energy Levels ... 13
TBT ... 13
T_2BT_2 ... 15
T_3BT_3 ... 18
TBpyT ... 22
EBE ... 24
Series 2 ... 28
Benchmarking Plots ... 28

UV-Vis Experimental vs DFT Overlays + FMO Density Plots 29
PBP........ 34
P_2BP_2........ 34
PBpyP........ 36
PyBPy........ 36
FBF........ 37
Cartesian Coordinates........ 34
Energy Levels........ 39
PBP........ 39
P_2BP_2........ 42
PBpyP........ 47
PyBPy........ 50
FBF........ 53
References........ 60

Computational Details

Benchmarking Functionals & Basis Sets

To benchmark each set, the following functionals were used: OHSEh1PBE[1], OHSeh2PBE[2], X3LYP[3], B98[4], B971[5], B972[6], B1LYP[7], B3LYP,[8] B3PW91,[9] HSEh1PBE,[10] mPW3PBE,[11] mPW1PBE,[12] mPW1LYP,[13] along with 6-31Gd,[14] and SV[15] basis sets. The upper and lower peak maxima for the experimental versus the computationally derived data for Series 1 (TBT, T_2BT_2, T_3BT_3, TBpyT, EBE) and Series 2(PBP, P_2BP_2, PByP, PyBPy) were plotted. The R^2 value from the fitted line was utilized to determine the best pairing. The plots for the best few ($R^2 > 0.96$ (Series 1), $R^2 > 0.953$ (Series 2)) are shown below where the plot highlighted in orange was the best pairing and then utilized for all results and images.

Solvent criteria

eps=36.71 epsinf=1.75

Density Functional Theory (DFT)

Series 1

uhseh1pbe scrf=(cpcm,solvent=generic,read) geom=connectivity sv

Series 2

ub3lyp/6-31G(d) scrf=(cpcm,solvent=generic,read) geom=connectivity

Time Dependent DFT

Series 1

td=(nstates=15) uhseh1pbe scrf=(cpcm,solvent=generic,read) geom=connectivity sv

Series 2

td=(nstates=15)ub3lyp/6-31G(d) scrf=(cpcm,solvent=generic,read) geom=connectivity

Series 1

Benchmarking Plots

Table DFT-SI.1: Benchmarking for Series 1 (TBT, T_2BT_2, T_3BT_3, $TB_{py}T$, EBE). For each structure the computational wavelength maxima were compared to those generated from experiment and plotted to identify the best basis-set/functional pairing that would work for all 5 systems. The top 8 (identified by their R^2 values) are shown below. For this series the HSEh1PBE/SV pairing was found to possess the strongest correlation with an R^2 value of 0.9745.

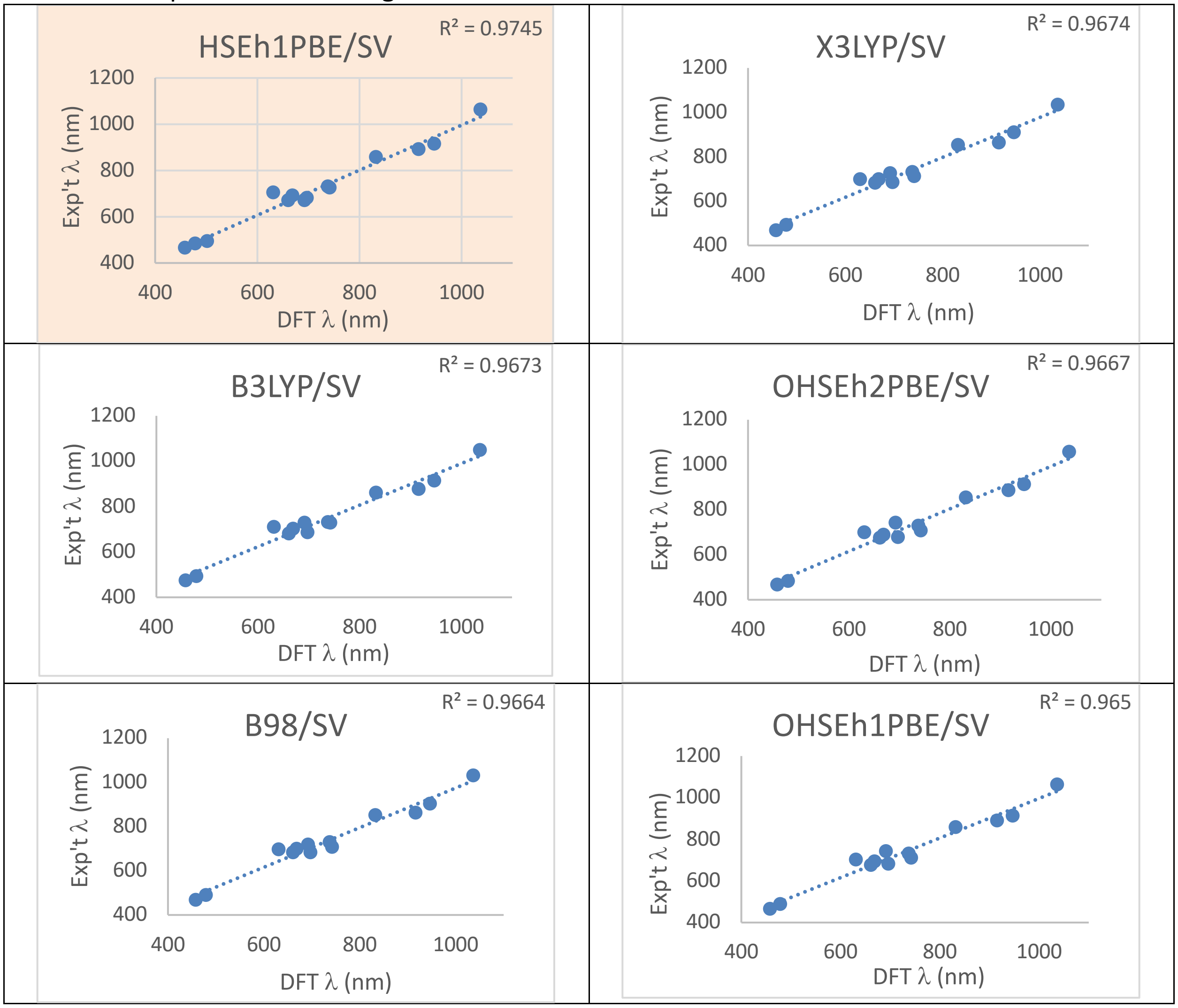

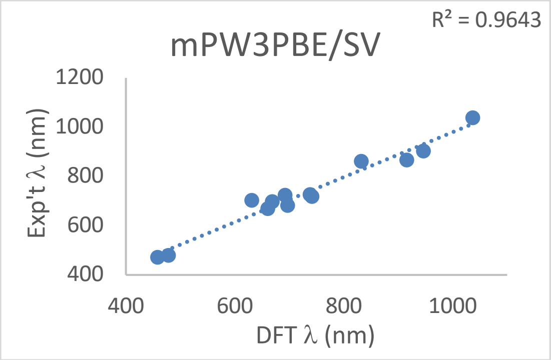

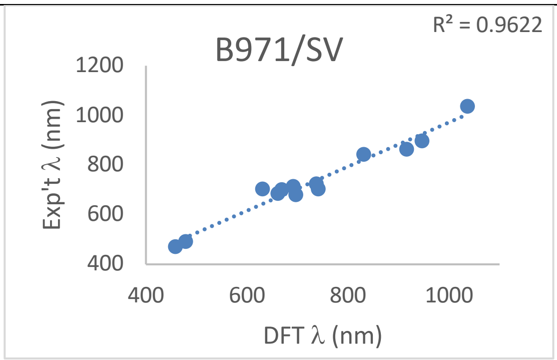

UV-Vis Experimental vs DFT Overlays + FMO Density Plots

TBT

a

b

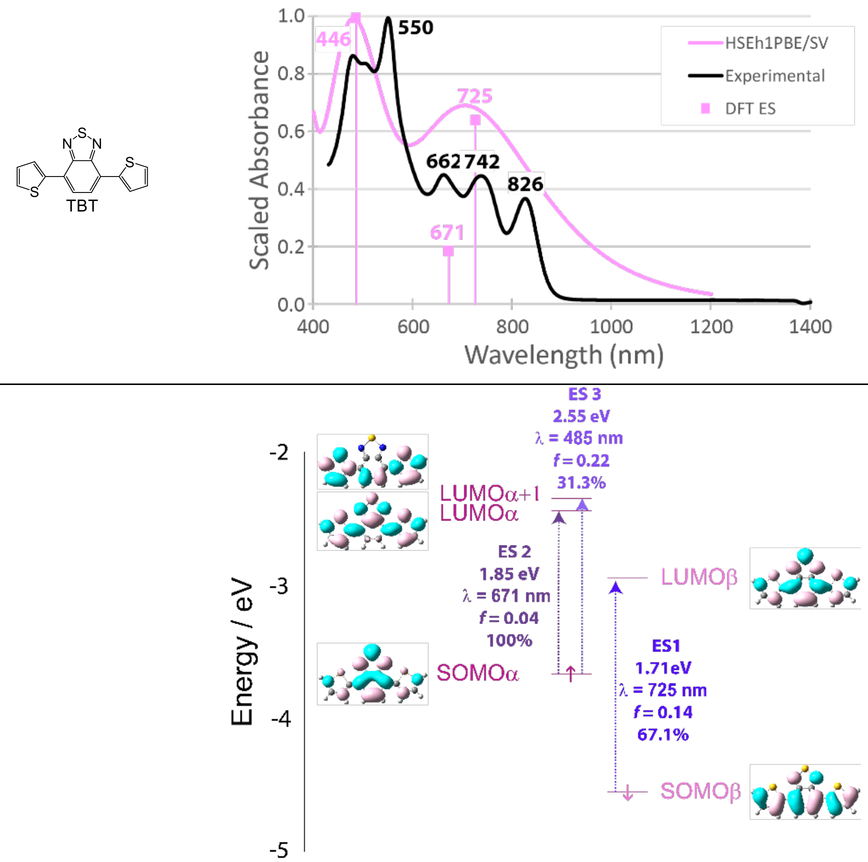

Figure DFT-SI.1: a. An overlay of the experimental (black) and benchmarked computational (pink) UV-visible absorption spectra is shown. The height of the lines below are indicative of oscillator strengths and the numbers correspond to the peak maxima (black/experimental) or excited state value in nm. **b.** Diagram with corresponding energy levels and frontier molecular orbitals is given. The excited state energy (eV), oscillator strength (*f*), and percentage of contribution of the indicated transition is provided for the lowest 3 lying transitions for TBT.

T_2BT_2

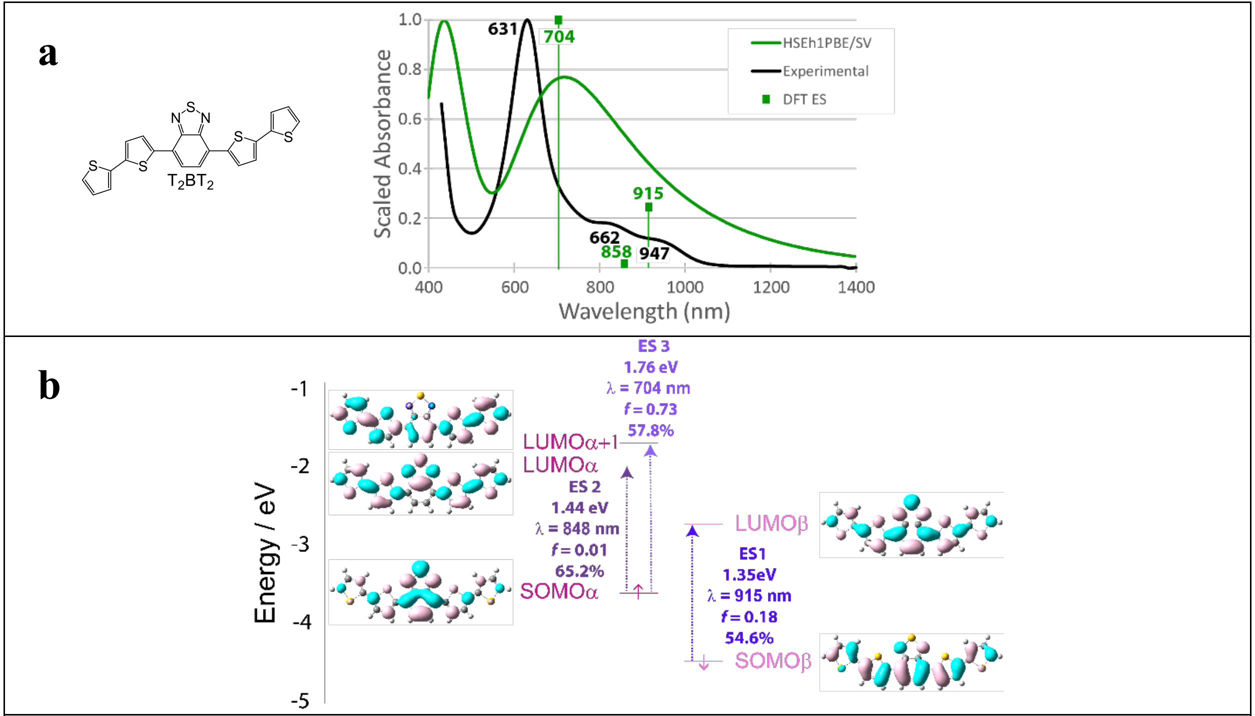

Figure DFT-SI.2: a. An overlay of the experimental (black) and benchmarked computational (green) UV-visible absorption spectra is shown. The height of the lines below are indicative of oscillator strengths and the numbers correspond to the peak maxima (black/experimental) or excited state value in nm. **b.** Diagram with corresponding energy levels and frontier molecular orbitals is given. The excited state energy (eV), oscillator strength (*f*), and percentage of contribution of the indicated transition is provided for the lowest 3 lying transitions for T_2BT_2.

T_3BT_3

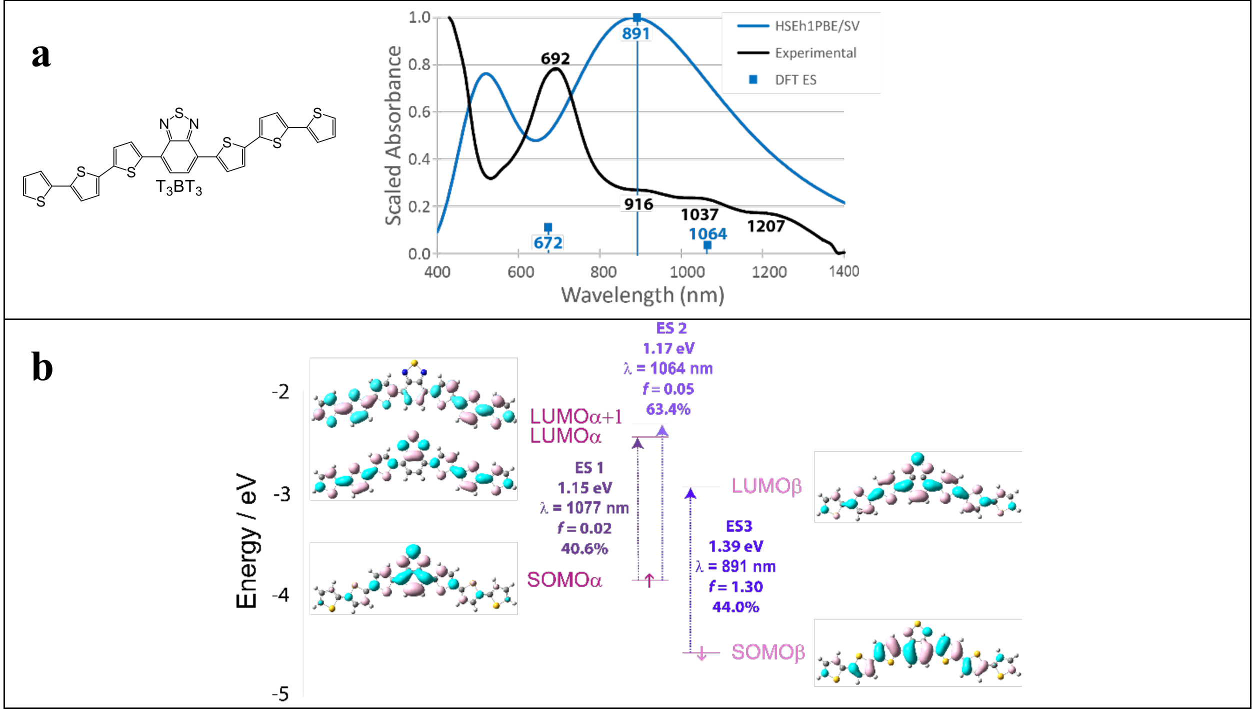

Figure DFT-SI.3: a. An overlay of the experimental (black) and benchmarked computational (blue) UV-visible absorption spectra is shown. The height of the lines below are indicative of oscillator strengths and the numbers correspond to the peak maxima (black/experimental) or excited state value in nm. **b.** Diagram with corresponding energy levels and frontier molecular orbitals is given. The excited state energy (eV), oscillator strength (*f*), and percentage of contribution of the indicated transition is provided for the lowest 3 lying transitions for T_3BT_3.

TBpyT

a

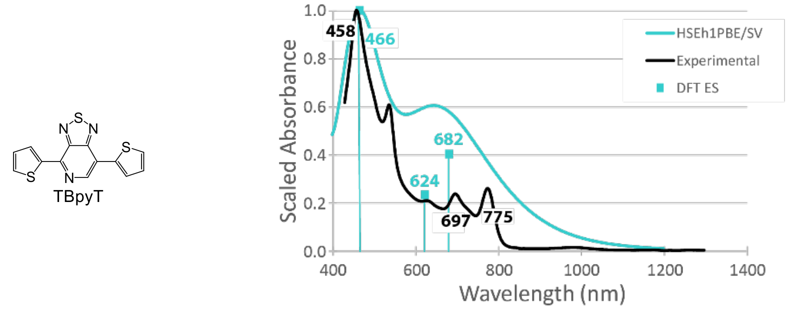

b

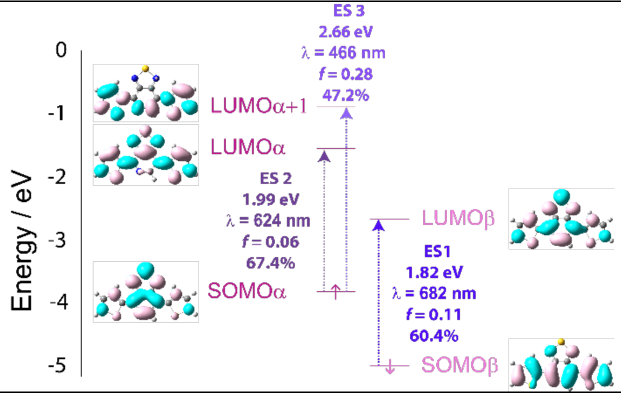

Figure DFT-SI.4: a. An overlay of the experimental (black) and benchmarked computational (cyan) UV-visible absorption spectra is shown. The height of the lines below are indicative of oscillator strengths and the numbers correspond to the peak maxima (black/experimental) or excited state value in nm. **b.** Diagram with corresponding energy levels and frontier molecular orbitals is given. The excited state energy (eV), oscillator strength (*f*), and percentage of contribution of the indicated transition is provided for the lowest 3 lying transitions for TBpyT.

EBE

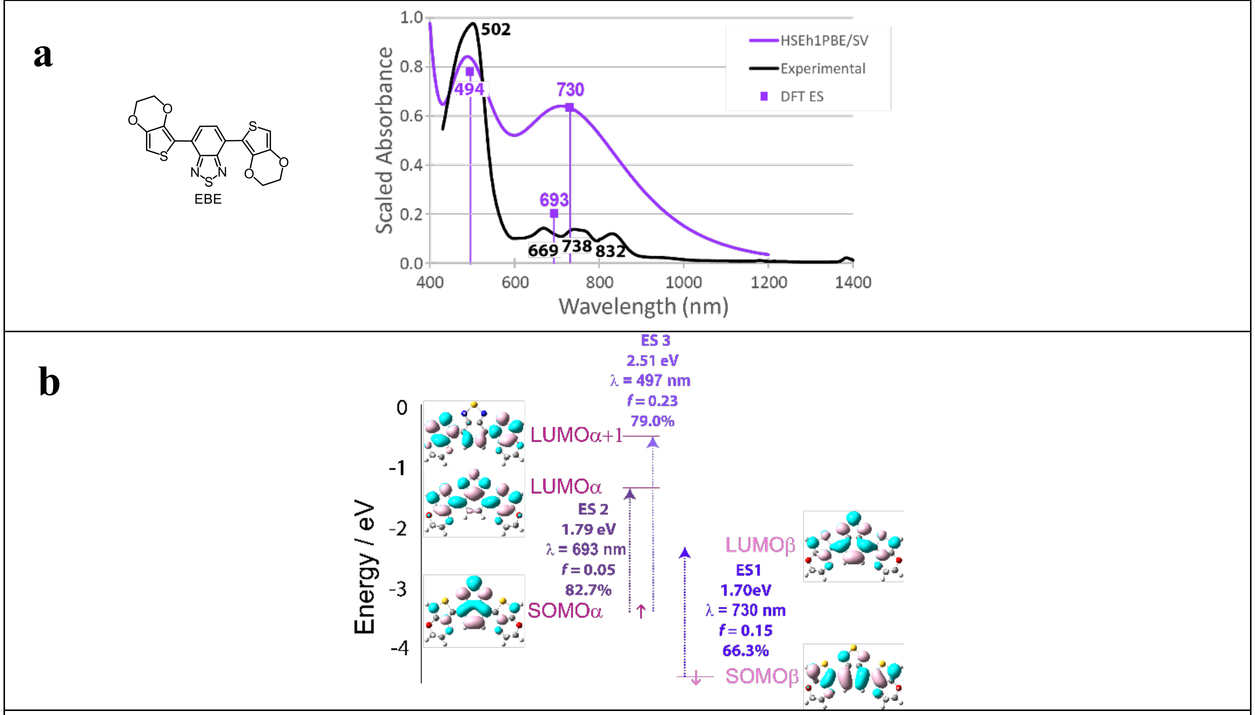

Figure DFT-SI.5: a. An overlay of the experimental (black) and benchmarked computational (violet) UV-visible absorption spectra is shown. The height of the lines below are indicative of oscillator strengths and the numbers correspond to the peak maxima (black/experimental) or excited state value in nm. **b.** Diagram with corresponding energy levels and frontier molecular orbitals is given. The excited state energy (eV), oscillator strength (*f*), and percentage of contribution of the indicated transition is provided for the lowest 3 lying transitions for EBE.

Cartesian Coordinates

TBT

C	0.	-0.73441	0.52844
C	0.	-1.4666	-0.70254
C	0.	-0.69705	-1.89291
C	0.	0.69705	-1.89291
C	0.	1.4666	-0.70254
H	0.	-1.21041	-2.85708
H	0.	1.21041	-2.85708
S	0.	0.	2.9501
N	0.	-1.31828	1.74121
N	0.	1.31828	1.74121
C	0.	2.91316	-0.75631
C	0.	3.72503	-1.8834
S	0.	3.95214	0.73347
C	0.	5.12893	-1.61346
H	0.	3.32152	-2.89759
C	0.	5.43043	-0.27516
H	0.	5.88863	-2.39901
H	0.	6.41016	0.20089
C	0.	-2.91316	-0.75631
C	0.	-3.72503	-1.8834
S	0.	-3.95214	0.73347
C	0.	-5.12893	-1.61346
H	0.	-3.32152	-2.89759
C	0.	-5.43043	-0.27516
H	0.	-5.88863	-2.39901
H	0.	-6.41016	0.20089

T_2BT_2

C	0.73423	0.30806	-0.00002
C	-0.73423	0.30806	0.00003
C	-1.46886	-0.92362	0.00004
C	-0.69582	-2.11542	0.00002
C	0.69583	-2.11542	-0.00002
C	1.46886	-0.92362	-0.00003
H	-1.20898	-3.07956	0.
H	1.20898	-3.07956	0.00001
N	-1.31238	1.51878	-0.00003
N	1.31238	1.51878	0.00004
S	0.	2.72648	0.
C	-2.90664	-0.96891	0.0001
C	-3.72923	-2.09386	0.00031
C	-5.11921	-1.82075	0.00024

C	-5.44067	-0.47372	0.00008
C	2.90664	-0.96891	-0.00009
C	3.72923	-2.09386	-0.0003
C	5.11921	-1.82075	-0.00024
C	5.44067	-0.47372	-0.00008
S	-3.9319	0.52928	-0.00012
S	3.9319	0.52928	0.00013
H	-5.87958	-2.60655	0.00037
H	-3.33249	-3.11037	0.00051
H	3.33249	-3.11037	-0.00051
H	5.87958	-2.60655	-0.00037
C	-6.72497	0.15552	0.00003
C	-7.05166	1.50056	0.
S	-8.23447	-0.84628	-0.00005
C	-8.45947	1.76581	-0.00007
H	-6.29143	2.28527	0.00001
C	-9.23523	0.63588	-0.00009
H	-8.88002	2.77372	-0.0001
H	-10.32045	0.54884	-0.00015
C	6.72497	0.15552	-0.00003
C	7.05166	1.50056	0.
S	8.23447	-0.84628	0.00003
C	8.45947	1.76581	0.00006
H	6.29143	2.28527	0.
C	9.23523	0.63588	0.00008
H	8.88002	2.77372	0.00009
H	10.32045	0.54884	0.00013

T_3BT_3

C	0.	2.27997	0.73791
C	0.	2.27997	-0.73791
C	0.	1.03788	-1.47113
C	0.	-0.14934	-0.69539
C	0.	-0.14934	0.69539
C	0.	1.03788	1.47113
H	0.	-1.11425	-1.21148
H	0.	-1.11425	1.21148
S	0.	4.69655	0.
N	0.	3.49374	1.30781
N	0.	3.49374	-1.30781
C	0.	1.00367	2.90526
C	0.	2.04589	3.82899
S	0.	-0.58807	3.78828
C	0.	1.63765	5.18579

H	0.	3.08139	3.48922
C	0.	0.26481	5.38501
H	0.	2.34484	6.01975
C	0.	1.00367	-2.90526
C	0.	2.04589	-3.82899
S	0.	-0.58807	-3.78828
C	0.	1.63765	-5.18579
H	0.	3.08139	-3.48922
C	0.	0.26481	-5.38501
H	0.	2.34484	-6.01975
C	0.	-0.48269	-6.59775
C	0.	-1.8558	-6.79439
S	0.	0.37082	-8.19413
C	0.	-2.25662	-8.15742
H	0.	-2.56404	-5.96283
C	0.	-1.21425	-9.06639
H	0.	-3.30358	-8.47033
C	0.	-1.24391	-10.49767
C	0.	-0.19694	-11.4009
S	0.	-2.82429	-11.37597
C	0.	-0.60603	-12.77343
H	0.	0.84917	-11.08546
C	0.	-1.96623	-12.94213
H	0.	0.09615	-13.60949
H	0.	-2.54201	-13.86614
C	0.	-0.48269	6.59775
C	0.	-1.8558	6.79439
S	0.	0.37082	8.19413
C	0.	-2.25662	8.15742
H	0.	-2.56404	5.96283
C	0.	-1.21425	9.06639
H	0.	-3.30358	8.47033
C	0.	-1.24391	10.49767
C	0.	-0.19694	11.4009
S	0.	-2.82429	11.37597
C	0.	-0.60603	12.77343
H	0.	0.84917	11.08546
C	0.	-1.96623	12.94213
H	0.	0.09615	13.60949
H	0.	-2.54201	13.86614

TBpyT

C	-0.73292	1.483	0.00226
C	0.73292	1.483	-0.00224

C	1.46784	0.25176	-0.00368
C	0.69796	-0.9398	-0.00281
C	-0.69796	-0.9398	0.00281
C	-1.46784	0.25176	0.00368
H	1.22701	-1.8928	-0.00625
H	-1.22701	-1.8928	0.00623
S	0.	3.90779	0.00003
N	1.31834	2.69646	-0.00492
N	-1.31834	2.69646	0.00496
C	2.9122	0.2226	-0.0054
C	3.76858	-0.87031	0.01964
S	3.91812	1.74298	-0.05096
C	5.16852	-0.5522	-0.00871
O	3.34293	-2.19593	0.06235
C	5.43413	0.78715	-0.04527
O	6.14623	-1.53704	0.01408
C	4.37563	-3.14315	0.46844
C	5.65436	-2.87492	-0.29455
H	4.54432	-3.054	1.55729
H	3.97303	-4.14067	0.24375
H	6.45371	-3.56788	0.00342
H	5.4849	-2.96148	-1.38341
C	-2.9122	0.2226	0.00539
C	-3.76858	-0.87031	-0.01965
S	-3.91812	1.74298	0.05091
C	-5.16852	-0.5522	0.00868
O	-3.34293	-2.19593	-0.06232
C	-5.43413	0.78715	0.04521
O	-6.14623	-1.53704	-0.0141
C	-4.37563	-3.14316	-0.46841
C	-5.65436	-2.87491	0.29456
H	-4.5443	-3.05403	-1.55726
H	-3.97302	-4.14067	-0.24369
H	-6.45371	-3.56788	-0.00341
H	-5.48492	-2.96145	1.38343
H	6.40742	1.27053	-0.07005
H	-6.40742	1.27053	0.06998

EBE

C	-0.73292	1.483	0.00226
C	0.73292	1.483	-0.00224
C	1.46784	0.25176	-0.00368
C	0.69796	-0.9398	-0.00281
C	-0.69796	-0.9398	0.00281

C	-1.46784	0.25176	0.00368
H	1.22701	-1.8928	-0.00625
H	-1.22701	-1.8928	0.00623
S	0.	3.90779	0.00003
N	1.31834	2.69646	-0.00492
N	-1.31834	2.69646	0.00496
C	2.9122	0.2226	-0.0054
C	3.76858	-0.87031	0.01964
S	3.91812	1.74298	-0.05096
C	5.16852	-0.5522	-0.00871
O	3.34293	-2.19593	0.06235
C	5.43413	0.78715	-0.04527
O	6.14623	-1.53704	0.01408
C	4.37563	-3.14315	0.46844
C	5.65436	-2.87492	-0.29455
H	4.54432	-3.054	1.55729
H	3.97303	-4.14067	0.24375
H	6.45371	-3.56788	0.00342
H	5.4849	-2.96148	-1.38341
C	-2.9122	0.2226	0.00539
C	-3.76858	-0.87031	-0.01965
S	-3.91812	1.74298	0.05091
C	-5.16852	-0.5522	0.00868
O	-3.34293	-2.19593	-0.06232
C	-5.43413	0.78715	0.04521
O	-6.14623	-1.53704	-0.0141
C	-4.37563	-3.14316	-0.46841
C	-5.65436	-2.87491	0.29456
H	-4.5443	-3.05403	-1.55726
H	-3.97302	-4.14067	-0.24369
H	-6.45371	-3.56788	-0.00341
H	-5.48492	-2.96145	1.38343
H	6.40742	1.27053	-0.07005
H	-6.40742	1.27053	0.06998

Energy Levels

TBT

Alpha occ. eigenvalues -- -89.03764 -89.02038 -89.02037 -14.38687 -14.38681
Alpha occ. eigenvalues -- -10.26010 -10.26002 -10.25829 -10.25806 -10.23895
Alpha occ. eigenvalues -- -10.23895 -10.23143 -10.23135 -10.22270 -10.22270
Alpha occ. eigenvalues -- -10.21784 -10.21783 -10.21544 -10.21494 -8.03315
Alpha occ. eigenvalues -- -8.01635 -8.01635 -5.97452 -5.96936 -5.96372
Alpha occ. eigenvalues -- -5.95651 -5.95651 -5.95227 -5.95227 -5.94770
Alpha occ. eigenvalues -- -5.94770 -0.97695 -0.88613 -0.88260 -0.86388

```
Alpha  occ. eigenvalues --   -0.84116  -0.77474  -0.74305  -0.73151  -0.72977
Alpha  occ. eigenvalues --   -0.72715  -0.68715  -0.64210  -0.60803  -0.59040
Alpha  occ. eigenvalues --   -0.55622  -0.55499  -0.53164  -0.52154  -0.50558
Alpha  occ. eigenvalues --   -0.49452  -0.47297  -0.46313  -0.44533  -0.42784
Alpha  occ. eigenvalues --   -0.41374  -0.39585  -0.39215  -0.38807  -0.38402
Alpha  occ. eigenvalues --   -0.38381  -0.37615  -0.36853  -0.35441  -0.34878
Alpha  occ. eigenvalues --   -0.33681  -0.33087  -0.32931  -0.32123  -0.28276
Alpha  occ. eigenvalues --   -0.26976  -0.25564  -0.25432  -0.24678  -0.24647
Alpha  occ. eigenvalues --   -0.23645  -0.18397  -0.12837
Alpha virt. eigenvalues --  -0.05047  -0.02092  -0.00339   0.01630   0.02807
Alpha virt. eigenvalues --   0.02846   0.05413   0.06279   0.06563   0.08570
Alpha virt. eigenvalues --   0.08591   0.09499   0.09565   0.09600   0.11227
Alpha virt. eigenvalues --   0.12304   0.14860   0.14879   0.17752   0.18880
Alpha virt. eigenvalues --   0.21094   0.22952   0.24830   0.25061   0.27803
Alpha virt. eigenvalues --   0.28707   0.29207   0.30550   0.30897   0.31420
Alpha virt. eigenvalues --   0.34398   0.34693   0.35542   0.36494   0.38313
Alpha virt. eigenvalues --   0.39332   0.40799   0.41586   0.42713   0.43068
Alpha virt. eigenvalues --   0.44348   0.44422   0.44589   0.45716   0.46114
Alpha virt. eigenvalues --   0.47968   0.48846   0.49050   0.49421   0.49971
Alpha virt. eigenvalues --   0.51668   0.52634   0.53439   0.53868   0.55568
Alpha virt. eigenvalues --   0.55894   0.56096   0.56397   0.57443   0.57496
Alpha virt. eigenvalues --   0.58596   0.59455   0.60672   0.62272   0.63213
Alpha virt. eigenvalues --   0.65819   0.66151   0.66865   0.67782   0.67959
Alpha virt. eigenvalues --   0.69327   0.69363   0.69620   0.70095   0.71271
Alpha virt. eigenvalues --   0.71729   0.72637   0.73168   0.73289   0.73864
Alpha virt. eigenvalues --   0.74547   0.75002   0.75510   0.75625   0.77324
Alpha virt. eigenvalues --   0.78338   0.79580   0.79794   0.80043   0.81062
Alpha virt. eigenvalues --   0.81076   0.83230   0.83352   0.85224   0.85471
Alpha virt. eigenvalues --   0.87056   0.87057   0.87328   0.88980   0.91926
Alpha virt. eigenvalues --   0.97889   0.98378   1.07275   1.08885   1.10770
Alpha virt. eigenvalues --   1.11561   1.15831   1.16405   1.18154   1.20298
Alpha virt. eigenvalues --   1.23213   1.26851   1.28394   1.29495   1.31547
Alpha virt. eigenvalues --   1.34960   1.36814   1.38207   1.43500   1.44324
Alpha virt. eigenvalues --   1.55450
 Beta  occ. eigenvalues -- -89.03668 -89.02034 -89.02033 -14.38095 -14.38090
 Beta  occ. eigenvalues -- -10.26091 -10.26083 -10.25939 -10.25916 -10.23782
 Beta  occ. eigenvalues -- -10.23782 -10.22920 -10.22912 -10.22325 -10.22325
 Beta  occ. eigenvalues -- -10.21659 -10.21658 -10.21464 -10.21415  -8.03218
 Beta  occ. eigenvalues --  -8.01631  -8.01631  -5.97409  -5.96897  -5.96018
 Beta  occ. eigenvalues --  -5.95650  -5.95650  -5.95226  -5.95226  -5.94755
 Beta  occ. eigenvalues --  -5.94755  -0.97036  -0.88377  -0.88140  -0.85594
 Beta  occ. eigenvalues --  -0.83741  -0.77063  -0.74037  -0.73058  -0.72920
 Beta  occ. eigenvalues --  -0.72504  -0.68460  -0.63622  -0.60494  -0.58810
 Beta  occ. eigenvalues --  -0.55537  -0.55410  -0.53038  -0.52055  -0.50507
 Beta  occ. eigenvalues --  -0.49296  -0.47123  -0.45999  -0.44444  -0.41808
 Beta  occ. eigenvalues --  -0.41308  -0.39477  -0.39125  -0.38761  -0.38268
```

Beta occ. eigenvalues -- -0.38071 -0.37061 -0.36694 -0.35011 -0.34779
Beta occ. eigenvalues -- -0.33043 -0.32860 -0.32376 -0.30648 -0.27778
Beta occ. eigenvalues -- -0.25263 -0.24778 -0.24770 -0.24137 -0.24082
Beta occ. eigenvalues -- -0.23183 -0.16849
Beta virt. eigenvalues -- -0.08841 -0.03054 -0.01765 0.00176 0.02523
Beta virt. eigenvalues -- 0.03086 0.03540 0.05432 0.06643 0.06725
Beta virt. eigenvalues -- 0.08624 0.08754 0.09592 0.09652 0.10369
Beta virt. eigenvalues -- 0.11329 0.12360 0.14919 0.14924 0.17818
Beta virt. eigenvalues -- 0.19747 0.21299 0.23010 0.24831 0.25253
Beta virt. eigenvalues -- 0.27955 0.28890 0.29292 0.30714 0.31029
Beta virt. eigenvalues -- 0.31515 0.34595 0.34725 0.35687 0.36677
Beta virt. eigenvalues -- 0.38461 0.39623 0.40962 0.41637 0.42879
Beta virt. eigenvalues -- 0.43131 0.44404 0.44546 0.44667 0.46051
Beta virt. eigenvalues -- 0.46237 0.48189 0.48987 0.49164 0.49633
Beta virt. eigenvalues -- 0.50169 0.51985 0.52663 0.53566 0.54091
Beta virt. eigenvalues -- 0.55679 0.56200 0.56347 0.56759 0.57552
Beta virt. eigenvalues -- 0.57598 0.58746 0.59486 0.60847 0.62390
Beta virt. eigenvalues -- 0.63471 0.65905 0.66462 0.67755 0.67904
Beta virt. eigenvalues -- 0.68073 0.69450 0.69486 0.69763 0.70210
Beta virt. eigenvalues -- 0.71427 0.71779 0.72792 0.73324 0.73440
Beta virt. eigenvalues -- 0.74428 0.74645 0.75557 0.75641 0.76015
Beta virt. eigenvalues -- 0.77412 0.78401 0.79903 0.80086 0.80117
Beta virt. eigenvalues -- 0.81156 0.81168 0.83380 0.83409 0.85408
Beta virt. eigenvalues -- 0.85671 0.87312 0.88888 0.88920 0.89168
Beta virt. eigenvalues -- 0.92017 0.98115 0.98547 1.07409 1.08976
Beta virt. eigenvalues -- 1.10842 1.11731 1.15934 1.16565 1.18203
Beta virt. eigenvalues -- 1.20449 1.23554 1.26948 1.28590 1.29560
Beta virt. eigenvalues -- 1.31663 1.35223 1.37047 1.38255 1.43761
Beta virt. eigenvalues -- 1.44644 1.55513

T_2BT_2

Alpha occ. eigenvalues -- -89.04709 -89.03215 -89.03215 -89.02542 -89.02541
Alpha occ. eigenvalues -- -14.39661 -14.39655 -10.27457 -10.27457 -10.26616
Alpha occ. eigenvalues -- -10.26611 -10.26605 -10.26585 -10.26190 -10.26190
Alpha occ. eigenvalues -- -10.25526 -10.25526 -10.23716 -10.23708 -10.23379
Alpha occ. eigenvalues -- -10.23379 -10.23138 -10.23138 -10.22978 -10.22977
Alpha occ. eigenvalues -- -10.22406 -10.22405 -10.22086 -10.22034 -8.04260
Alpha occ. eigenvalues -- -8.02955 -8.02955 -8.02071 -8.02071 -5.98396
Alpha occ. eigenvalues -- -5.97875 -5.97304 -5.96979 -5.96979 -5.96564
Alpha occ. eigenvalues -- -5.96564 -5.96113 -5.96113 -5.96056 -5.96056
Alpha occ. eigenvalues -- -5.95690 -5.95690 -5.95171 -5.95171 -0.98792
Alpha occ. eigenvalues -- -0.90349 -0.90273 -0.88791 -0.88293 -0.87136
Alpha occ. eigenvalues -- -0.84797 -0.79241 -0.77244 -0.75254 -0.75227
Alpha occ. eigenvalues -- -0.74699 -0.74607 -0.73291 -0.73155 -0.71052
Alpha occ. eigenvalues -- -0.68539 -0.65147 -0.62446 -0.60744 -0.59451
Alpha occ. eigenvalues -- -0.58761 -0.57012 -0.55970 -0.53877 -0.53344

Alpha occ. eigenvalues -- -0.52729 -0.52674 -0.51993 -0.50345 -0.49543
Alpha occ. eigenvalues -- -0.47198 -0.46923 -0.46915 -0.43958 -0.43510
Alpha occ. eigenvalues -- -0.42074 -0.41791 -0.41613 -0.40711 -0.40407
Alpha occ. eigenvalues -- -0.40379 -0.40158 -0.39957 -0.39321 -0.38855
Alpha occ. eigenvalues -- -0.37891 -0.37767 -0.37349 -0.37069 -0.36133
Alpha occ. eigenvalues -- -0.35624 -0.34453 -0.34281 -0.33905 -0.33266
Alpha occ. eigenvalues -- -0.33197 -0.33065 -0.28909 -0.28837 -0.27897
Alpha occ. eigenvalues -- -0.26250 -0.26110 -0.26109 -0.25990 -0.25169
Alpha occ. eigenvalues -- -0.25135 -0.24197 -0.20987 -0.17676 -0.13325
Alpha virt. eigenvalues -- -0.07371 -0.06280 -0.02440 -0.01017 -0.00465
Alpha virt. eigenvalues -- 0.01305 0.01728 0.02189 0.02322 0.02995
Alpha virt. eigenvalues -- 0.03736 0.05112 0.05684 0.06318 0.06941
Alpha virt. eigenvalues -- 0.07648 0.07949 0.08350 0.08756 0.08923
Alpha virt. eigenvalues -- 0.08971 0.09941 0.10809 0.11328 0.12471
Alpha virt. eigenvalues -- 0.13147 0.14869 0.15164 0.17078 0.17860
Alpha virt. eigenvalues -- 0.18357 0.18744 0.20715 0.22803 0.23436
Alpha virt. eigenvalues -- 0.24018 0.25558 0.26228 0.27189 0.27244
Alpha virt. eigenvalues -- 0.27559 0.28415 0.28662 0.30058 0.30948
Alpha virt. eigenvalues -- 0.31615 0.32364 0.32607 0.33837 0.33967
Alpha virt. eigenvalues -- 0.35526 0.36144 0.37044 0.38004 0.38778
Alpha virt. eigenvalues -- 0.38930 0.39044 0.39883 0.41113 0.41280
Alpha virt. eigenvalues -- 0.41804 0.42595 0.42661 0.43773 0.44210
Alpha virt. eigenvalues -- 0.44758 0.45276 0.45351 0.45778 0.46362
Alpha virt. eigenvalues -- 0.46542 0.46638 0.46893 0.47134 0.47542
Alpha virt. eigenvalues -- 0.48045 0.49168 0.49661 0.50453 0.50950
Alpha virt. eigenvalues -- 0.51639 0.52019 0.52260 0.52959 0.53998
Alpha virt. eigenvalues -- 0.54288 0.55045 0.55447 0.55693 0.55899
Alpha virt. eigenvalues -- 0.56333 0.56803 0.58041 0.58296 0.58382
Alpha virt. eigenvalues -- 0.59863 0.60819 0.61018 0.61748 0.63507
Alpha virt. eigenvalues -- 0.63717 0.63975 0.65473 0.65602 0.67280
Alpha virt. eigenvalues -- 0.67545 0.67589 0.67985 0.67998 0.68463
Alpha virt. eigenvalues -- 0.69449 0.70103 0.70365 0.70417 0.70485
Alpha virt. eigenvalues -- 0.70711 0.71203 0.71214 0.71621 0.71815
Alpha virt. eigenvalues -- 0.72404 0.73260 0.73272 0.73718 0.74430
Alpha virt. eigenvalues -- 0.74870 0.74970 0.75081 0.75568 0.75787
Alpha virt. eigenvalues -- 0.76232 0.76294 0.76362 0.76623 0.77644
Alpha virt. eigenvalues -- 0.77870 0.79721 0.79756 0.79898 0.80814
Alpha virt. eigenvalues -- 0.81359 0.82415 0.82650 0.83690 0.85034
Alpha virt. eigenvalues -- 0.85679 0.86458 0.87257 0.87304 0.88277
Alpha virt. eigenvalues -- 0.90137 0.91392 0.91858 0.94717 0.96453
Alpha virt. eigenvalues -- 0.99471 1.01716 1.03073 1.08967 1.09738
Alpha virt. eigenvalues -- 1.09893 1.10818 1.12107 1.14233 1.14931
Alpha virt. eigenvalues -- 1.15136 1.17753 1.19635 1.20903 1.23126
Alpha virt. eigenvalues -- 1.23310 1.24261 1.24852 1.26175 1.26965
Alpha virt. eigenvalues -- 1.28774 1.29426 1.34495 1.35880 1.38812
Alpha virt. eigenvalues -- 1.39777 1.42493 1.47205 1.47230 1.55088

Beta occ. eigenvalues -- -89.04622 -89.03210 -89.03210 -89.02538 -89.02537
Beta occ. eigenvalues -- -14.39157 -14.39152 -10.27515 -10.27515 -10.26715
Beta occ. eigenvalues -- -10.26708 -10.26698 -10.26691 -10.26047 -10.26047
Beta occ. eigenvalues -- -10.25455 -10.25455 -10.23497 -10.23489 -10.23414
Beta occ. eigenvalues -- -10.23414 -10.23059 -10.23059 -10.23055 -10.23055
Beta occ. eigenvalues -- -10.22271 -10.22270 -10.22005 -10.21955 -8.04171
Beta occ. eigenvalues --   -8.02950 -8.02950 -8.02067 -8.02067 -5.98356
Beta occ. eigenvalues --   -5.97838 -5.96983 -5.96977 -5.96977 -5.96562
Beta occ. eigenvalues --   -5.96562 -5.96112 -5.96112 -5.96039 -5.96039
Beta occ. eigenvalues --   -5.95689 -5.95689 -5.95153 -5.95153 -0.98228
Beta occ. eigenvalues --   -0.90258 -0.90201 -0.88504 -0.88197 -0.86572
Beta occ. eigenvalues --   -0.84450 -0.78952 -0.77141 -0.75099 -0.75053
Beta occ. eigenvalues --   -0.74606 -0.74418 -0.73234 -0.73073 -0.70937
Beta occ. eigenvalues --   -0.68350 -0.64657 -0.62166 -0.60614 -0.59360
Beta occ. eigenvalues --   -0.58641 -0.56929 -0.55918 -0.53780 -0.53268
Beta occ. eigenvalues --   -0.52690 -0.52626 -0.51962 -0.50215 -0.49458
Beta occ. eigenvalues --   -0.46959 -0.46827 -0.46770 -0.43898 -0.42666
Beta occ. eigenvalues --   -0.42005 -0.41736 -0.41574 -0.40489 -0.40312
Beta occ. eigenvalues --   -0.40137 -0.40126 -0.39921 -0.39296 -0.38750
Beta occ. eigenvalues --   -0.37838 -0.37499 -0.36893 -0.36848 -0.35841
Beta occ. eigenvalues --   -0.35556 -0.34428 -0.33865 -0.33192 -0.33165
Beta occ. eigenvalues --   -0.33145 -0.31954 -0.28423 -0.27565 -0.27547
Beta occ. eigenvalues --   -0.25993 -0.25956 -0.25561 -0.25253 -0.24987
Beta occ. eigenvalues --   -0.24698 -0.23096 -0.20646 -0.16518
Beta virt. eigenvalues --   -0.10134 -0.06106 -0.05829 -0.01510 -0.00574
Beta virt. eigenvalues --   0.00006   0.01490   0.02193   0.02345   0.02356
Beta virt. eigenvalues --   0.03421   0.03780   0.05406   0.05768   0.06370
Beta virt. eigenvalues --   0.07214   0.07980   0.08118   0.08374   0.08854
Beta virt. eigenvalues --   0.08999   0.09173   0.10466   0.10867   0.11403
Beta virt. eigenvalues --   0.12513   0.13164   0.14895   0.15185   0.17121
Beta virt. eigenvalues --   0.17898   0.18812   0.19179   0.20880   0.22859
Beta virt. eigenvalues --   0.23461   0.24169   0.25668   0.26265   0.27257
Beta virt. eigenvalues --   0.27287   0.27647   0.28505   0.28856   0.30219
Beta virt. eigenvalues --   0.30995   0.31724   0.32450   0.32673   0.33879
Beta virt. eigenvalues --   0.34150   0.35612   0.36242   0.37127   0.38121
Beta virt. eigenvalues --   0.38914   0.39044   0.39151   0.40055   0.41181
Beta virt. eigenvalues --   0.41300   0.41907   0.42656   0.42735   0.43877
Beta virt. eigenvalues --   0.44287   0.44806   0.45483   0.45589   0.45830
Beta virt. eigenvalues --   0.46409   0.46609   0.46735   0.47037   0.47263
Beta virt. eigenvalues --   0.47615   0.48214   0.49300   0.49780   0.50642
Beta virt. eigenvalues --   0.51028   0.51717   0.52057   0.52432   0.53098
Beta virt. eigenvalues --   0.54218   0.54454   0.55115   0.55523   0.55985
Beta virt. eigenvalues --   0.56009   0.56580   0.56891   0.58114   0.58346
Beta virt. eigenvalues --   0.58424   0.59974   0.60917   0.61207   0.61911
Beta virt. eigenvalues --   0.63593   0.64128   0.64274   0.65555   0.65760
Beta virt. eigenvalues --   0.67378   0.67701   0.67764   0.68076   0.68541

Beta virt. eigenvalues -- 0.68624 0.69578 0.70153 0.70366 0.70439
Beta virt. eigenvalues -- 0.70501 0.70851 0.71284 0.71287 0.71710
Beta virt. eigenvalues -- 0.71867 0.72544 0.73335 0.73397 0.74090
Beta virt. eigenvalues -- 0.74885 0.74915 0.75018 0.75174 0.75849
Beta virt. eigenvalues -- 0.76169 0.76265 0.76340 0.76442 0.76764
Beta virt. eigenvalues -- 0.77750 0.77942 0.79829 0.79947 0.80095
Beta virt. eigenvalues -- 0.80879 0.81478 0.82447 0.82705 0.83852
Beta virt. eigenvalues -- 0.85344 0.85779 0.86664 0.88369 0.88547
Beta virt. eigenvalues -- 0.88808 0.90231 0.91427 0.91889 0.94794
Beta virt. eigenvalues -- 0.96525 0.99580 1.01845 1.03210 1.09017
Beta virt. eigenvalues -- 1.09810 1.09942 1.10910 1.12165 1.14297
Beta virt. eigenvalues -- 1.15017 1.15224 1.17817 1.19758 1.21044
Beta virt. eigenvalues -- 1.23139 1.23400 1.24337 1.24947 1.26276
Beta virt. eigenvalues -- 1.27035 1.28910 1.29546 1.34526 1.36056
Beta virt. eigenvalues -- 1.38962 1.39990 1.42568 1.47419 1.47430
Beta virt. eigenvalues -- 1.55145

T_3BT_3

Alpha occ. eigenvalues -- -89.05043 -89.03808 -89.03808 -89.03694 -89.03694
Alpha occ. eigenvalues -- -89.02729 -89.02728 -14.40050 -14.40045 -10.28071
Alpha occ. eigenvalues -- -10.28071 -10.28026 -10.28026 -10.27928 -10.27928
Alpha occ. eigenvalues -- -10.27170 -10.27159 -10.27159 -10.27149 -10.26726
Alpha occ. eigenvalues -- -10.26719 -10.26065 -10.26065 -10.24028 -10.24028
Alpha occ. eigenvalues -- -10.24007 -10.23999 -10.23840 -10.23840 -10.23792
Alpha occ. eigenvalues -- -10.23792 -10.23741 -10.23741 -10.23361 -10.23361
Alpha occ. eigenvalues -- -10.22349 -10.22297 -10.22086 -10.22084 -8.04626
Alpha occ. eigenvalues -- -8.03556 -8.03556 -8.03373 -8.03373 -8.02376
Alpha occ. eigenvalues -- -8.02375 -5.98757 -5.98233 -5.97668 -5.97580
Alpha occ. eigenvalues -- -5.97580 -5.97424 -5.97424 -5.97160 -5.97160
Alpha occ. eigenvalues -- -5.97007 -5.97007 -5.96653 -5.96653 -5.96439
Alpha occ. eigenvalues -- -5.96439 -5.96429 -5.96428 -5.96016 -5.96015
Alpha occ. eigenvalues -- -5.95454 -5.95454 -0.99237 -0.91495 -0.91490
Alpha occ. eigenvalues -- -0.90013 -0.89947 -0.88823 -0.88482 -0.87586
Alpha occ. eigenvalues -- -0.85098 -0.80248 -0.79337 -0.77699 -0.76252
Alpha occ. eigenvalues -- -0.75869 -0.75439 -0.75101 -0.74588 -0.74473
Alpha occ. eigenvalues -- -0.74168 -0.73545 -0.72133 -0.70513 -0.68892
Alpha occ. eigenvalues -- -0.65615 -0.63188 -0.62725 -0.61415 -0.60094
Alpha occ. eigenvalues -- -0.58909 -0.58582 -0.56447 -0.55989 -0.54851
Alpha occ. eigenvalues -- -0.54173 -0.53720 -0.53407 -0.53289 -0.53092
Alpha occ. eigenvalues -- -0.52596 -0.52188 -0.49932 -0.49286 -0.48093
Alpha occ. eigenvalues -- -0.47737 -0.45824 -0.45662 -0.44665 -0.43686
Alpha occ. eigenvalues -- -0.42803 -0.42460 -0.42404 -0.42014 -0.41935
Alpha occ. eigenvalues -- -0.41919 -0.41832 -0.41120 -0.40917 -0.40512
Alpha occ. eigenvalues -- -0.40159 -0.39949 -0.39674 -0.39268 -0.38756
Alpha occ. eigenvalues -- -0.37947 -0.37462 -0.37400 -0.37302 -0.37266
Alpha occ. eigenvalues -- -0.36789 -0.36765 -0.35568 -0.35158 -0.34613

Alpha occ. eigenvalues -- -0.34096 -0.33865 -0.33622 -0.33255 -0.32734
Alpha occ. eigenvalues -- -0.30085 -0.29954 -0.29274 -0.27069 -0.26893
Alpha occ. eigenvalues -- -0.26843 -0.26627 -0.26429 -0.26185 -0.26169
Alpha occ. eigenvalues -- -0.25595 -0.25462 -0.24922 -0.22258 -0.19956
Alpha occ. eigenvalues -- -0.17650 -0.13452
Alpha virt. eigenvalues -- -0.08879 -0.08523 -0.05081 -0.03754 -0.00870
Alpha virt. eigenvalues -- -0.00797 0.00479 0.00484 0.00625 0.00689
Alpha virt. eigenvalues -- 0.00938 0.01786 0.02255 0.02477 0.02983
Alpha virt. eigenvalues -- 0.04353 0.04683 0.04767 0.05161 0.06667
Alpha virt. eigenvalues -- 0.07390 0.07498 0.07746 0.07982 0.08139
Alpha virt. eigenvalues -- 0.08534 0.09024 0.09069 0.09069 0.09597
Alpha virt. eigenvalues -- 0.10729 0.10913 0.11330 0.12869 0.13005
Alpha virt. eigenvalues -- 0.14333 0.14344 0.16177 0.16190 0.16670
Alpha virt. eigenvalues -- 0.17097 0.17600 0.17739 0.19893 0.20268
Alpha virt. eigenvalues -- 0.21927 0.23264 0.23418 0.24873 0.25518
Alpha virt. eigenvalues -- 0.25612 0.25798 0.26193 0.26924 0.27184
Alpha virt. eigenvalues -- 0.27277 0.27671 0.27867 0.29111 0.29968
Alpha virt. eigenvalues -- 0.30772 0.30988 0.31140 0.31354 0.32343
Alpha virt. eigenvalues -- 0.32401 0.32822 0.34199 0.35603 0.36148
Alpha virt. eigenvalues -- 0.36474 0.36508 0.36675 0.37397 0.37675
Alpha virt. eigenvalues -- 0.38219 0.38315 0.39073 0.39839 0.40269
Alpha virt. eigenvalues -- 0.40441 0.41464 0.41767 0.42150 0.42759
Alpha virt. eigenvalues -- 0.42832 0.43474 0.43734 0.44006 0.44477
Alpha virt. eigenvalues -- 0.45077 0.45175 0.45485 0.45933 0.46221
Alpha virt. eigenvalues -- 0.46254 0.46353 0.46472 0.46949 0.46953
Alpha virt. eigenvalues -- 0.47021 0.47274 0.48181 0.48245 0.48345
Alpha virt. eigenvalues -- 0.48740 0.48977 0.49490 0.49853 0.50845
Alpha virt. eigenvalues -- 0.51061 0.51256 0.52123 0.52701 0.53254
Alpha virt. eigenvalues -- 0.53303 0.54274 0.54451 0.54600 0.54610
Alpha virt. eigenvalues -- 0.55067 0.55194 0.55224 0.55686 0.56633
Alpha virt. eigenvalues -- 0.57161 0.57941 0.57946 0.57965 0.60086
Alpha virt. eigenvalues -- 0.60669 0.60782 0.61149 0.61610 0.62083
Alpha virt. eigenvalues -- 0.63021 0.63047 0.64036 0.65250 0.65276
Alpha virt. eigenvalues -- 0.66280 0.66437 0.67248 0.67434 0.67599
Alpha virt. eigenvalues -- 0.67769 0.68013 0.68140 0.68954 0.68969
Alpha virt. eigenvalues -- 0.70074 0.70103 0.70163 0.70461 0.70482
Alpha virt. eigenvalues -- 0.70494 0.70894 0.70949 0.71167 0.71202
Alpha virt. eigenvalues -- 0.71876 0.71939 0.72688 0.72883 0.73027
Alpha virt. eigenvalues -- 0.73044 0.73388 0.73570 0.74011 0.74278
Alpha virt. eigenvalues -- 0.74318 0.74672 0.75143 0.75425 0.75609
Alpha virt. eigenvalues -- 0.75920 0.75981 0.76056 0.76353 0.76355
Alpha virt. eigenvalues -- 0.76806 0.76964 0.77191 0.77195 0.77345
Alpha virt. eigenvalues -- 0.78164 0.79004 0.79109 0.79931 0.79985
Alpha virt. eigenvalues -- 0.80815 0.80828 0.82053 0.82272 0.83649
Alpha virt. eigenvalues -- 0.85582 0.85587 0.86282 0.86555 0.88054
Alpha virt. eigenvalues -- 0.89051 0.90557 0.90851 0.91660 0.92549

Alpha virt. eigenvalues --   0.94450   0.96072   0.96559   0.97529   0.98236
Alpha virt. eigenvalues --   0.98710   1.00364   1.01889   1.02840   1.07202
Alpha virt. eigenvalues --   1.08159   1.09646   1.09760   1.12184   1.12250
Alpha virt. eigenvalues --   1.12959   1.13977   1.15765   1.16624   1.18078
Alpha virt. eigenvalues --   1.19452   1.20108   1.20757   1.21858   1.23159
Alpha virt. eigenvalues --   1.23622   1.23640   1.23745   1.24123   1.25320
Alpha virt. eigenvalues --   1.25642   1.27012   1.29767   1.29849   1.31917
Alpha virt. eigenvalues --   1.32163   1.34736   1.35554   1.39392   1.39599
Alpha virt. eigenvalues --   1.42246   1.47245   1.48042   1.54807
Beta occ. eigenvalues -- -89.04962 -89.03805 -89.03805 -89.03688 -89.03688
Beta occ. eigenvalues -- -89.02721 -89.02720 -14.39603 -14.39598 -10.28107
Beta occ. eigenvalues -- -10.28107 -10.28096 -10.28095 -10.27836 -10.27836
Beta occ. eigenvalues -- -10.27274 -10.27253 -10.27009 -10.27009 -10.26817
Beta occ. eigenvalues -- -10.26810 -10.26022 -10.26022 -10.24081 -10.24081
Beta occ. eigenvalues -- -10.23826 -10.23826 -10.23789 -10.23782 -10.23779
Beta occ. eigenvalues -- -10.23779 -10.23651 -10.23651 -10.23447 -10.23447
Beta occ. eigenvalues -- -10.22264 -10.22214 -10.21958 -10.21957 -8.04543
Beta occ. eigenvalues --   -8.03553 -8.03553 -8.03367 -8.03367 -8.02367
Beta occ. eigenvalues --   -8.02367 -5.98720 -5.98198 -5.97579 -5.97579
Beta occ. eigenvalues --   -5.97421 -5.97421 -5.97371 -5.97159 -5.97159
Beta occ. eigenvalues --   -5.97004 -5.97004 -5.96643 -5.96643 -5.96426
Beta occ. eigenvalues --   -5.96425 -5.96420 -5.96420 -5.96012 -5.96011
Beta occ. eigenvalues --   -5.95424 -5.95424 -0.98730 -0.91441 -0.91437
Beta occ. eigenvalues --   -0.89924 -0.89883 -0.88586 -0.88405 -0.87076
Beta occ. eigenvalues --   -0.84760 -0.80056 -0.79258 -0.77504 -0.76199
Beta occ. eigenvalues --   -0.75810 -0.75207 -0.75062 -0.74527 -0.74372
Beta occ. eigenvalues --   -0.74036 -0.73495 -0.72010 -0.70441 -0.68770
Beta occ. eigenvalues --   -0.65194 -0.62936 -0.62673 -0.61307 -0.60013
Beta occ. eigenvalues --   -0.58757 -0.58507 -0.56403 -0.55959 -0.54772
Beta occ. eigenvalues --   -0.54108 -0.53682 -0.53369 -0.53260 -0.53042
Beta occ. eigenvalues --   -0.52549 -0.52155 -0.49865 -0.49215 -0.47934
Beta occ. eigenvalues --   -0.47517 -0.45776 -0.45600 -0.44607 -0.42913
Beta occ. eigenvalues --   -0.42734 -0.42433 -0.42376 -0.41980 -0.41899
Beta occ. eigenvalues --   -0.41757 -0.41645 -0.41075 -0.40890 -0.40474
Beta occ. eigenvalues --   -0.40140 -0.39747 -0.39426 -0.39200 -0.38731
Beta occ. eigenvalues --   -0.37868 -0.37333 -0.37256 -0.37184 -0.36939
Beta occ. eigenvalues --   -0.36717 -0.36443 -0.35526 -0.35138 -0.34067
Beta occ. eigenvalues --   -0.33846 -0.33592 -0.33212 -0.32702 -0.32682
Beta occ. eigenvalues --   -0.29664 -0.29227 -0.28915 -0.26786 -0.26761
Beta occ. eigenvalues --   -0.26320 -0.26190 -0.26129 -0.25779 -0.25625
Beta occ. eigenvalues --   -0.25204 -0.24952 -0.24648 -0.21537 -0.19740
Beta occ. eigenvalues --   -0.16775
Beta virt. eigenvalues --   -0.10745 -0.08424 -0.07642 -0.04182 -0.03487
Beta virt. eigenvalues --   -0.00450 -0.00183   0.00503   0.00505   0.00740
Beta virt. eigenvalues --   0.00978   0.01098   0.02261   0.02299   0.02490
Beta virt. eigenvalues --   0.03304   0.04593   0.04787   0.04800   0.05588

Beta virt. eigenvalues --	0.07070	0.07419	0.07524	0.07945	0.07997
Beta virt. eigenvalues --	0.08168	0.08807	0.09066	0.09067	0.09240
Beta virt. eigenvalues --	0.10024	0.10744	0.10926	0.11388	0.12894
Beta virt. eigenvalues --	0.13051	0.14347	0.14365	0.16215	0.16258
Beta virt. eigenvalues --	0.16696	0.17158	0.17652	0.18503	0.19933
Beta virt. eigenvalues --	0.20307	0.21986	0.23323	0.23484	0.24943
Beta virt. eigenvalues --	0.25581	0.25667	0.25869	0.26248	0.26961
Beta virt. eigenvalues --	0.27224	0.27457	0.27696	0.27971	0.29242
Beta virt. eigenvalues --	0.30094	0.30784	0.31047	0.31261	0.31479
Beta virt. eigenvalues --	0.32377	0.32435	0.32894	0.34319	0.35724
Beta virt. eigenvalues --	0.36229	0.36562	0.36572	0.36728	0.37468
Beta virt. eigenvalues --	0.37769	0.38260	0.38410	0.39248	0.39909
Beta virt. eigenvalues --	0.40436	0.40504	0.41519	0.41790	0.42233
Beta virt. eigenvalues --	0.42775	0.42877	0.43511	0.43813	0.44094
Beta virt. eigenvalues --	0.44559	0.45168	0.45455	0.45508	0.45975
Beta virt. eigenvalues --	0.46316	0.46347	0.46378	0.46536	0.46988
Beta virt. eigenvalues --	0.47015	0.47139	0.47383	0.48312	0.48333
Beta virt. eigenvalues --	0.48405	0.48776	0.49085	0.49593	0.49999
Beta virt. eigenvalues --	0.50943	0.51088	0.51463	0.52196	0.52820
Beta virt. eigenvalues --	0.53404	0.53590	0.54318	0.54535	0.54675
Beta virt. eigenvalues --	0.54688	0.55102	0.55243	0.55600	0.55679
Beta virt. eigenvalues --	0.56701	0.57215	0.57971	0.57974	0.58011
Beta virt. eigenvalues --	0.60178	0.60859	0.60937	0.61305	0.61813
Beta virt. eigenvalues --	0.62116	0.63067	0.63161	0.64216	0.65291
Beta virt. eigenvalues --	0.65653	0.66384	0.66744	0.67290	0.67507
Beta virt. eigenvalues --	0.67730	0.67797	0.68032	0.68212	0.69036
Beta virt. eigenvalues --	0.69341	0.70087	0.70094	0.70273	0.70507
Beta virt. eigenvalues --	0.70564	0.70606	0.70953	0.71001	0.71226
Beta virt. eigenvalues --	0.71244	0.71911	0.71986	0.72721	0.72971
Beta virt. eigenvalues --	0.73074	0.73092	0.73435	0.73656	0.74055
Beta virt. eigenvalues --	0.74316	0.74742	0.74849	0.75215	0.75476
Beta virt. eigenvalues --	0.75641	0.75969	0.76047	0.76152	0.76444
Beta virt. eigenvalues --	0.76455	0.76865	0.77021	0.77236	0.77420
Beta virt. eigenvalues --	0.77679	0.78249	0.79289	0.79500	0.79965
Beta virt. eigenvalues --	0.80052	0.80892	0.80938	0.82086	0.82297
Beta virt. eigenvalues --	0.83706	0.85635	0.86352	0.87075	0.87665
Beta virt. eigenvalues --	0.88173	0.89169	0.90590	0.90966	0.91779
Beta virt. eigenvalues --	0.92684	0.94500	0.96128	0.96623	0.97573
Beta virt. eigenvalues --	0.98311	0.98791	1.00439	1.01954	1.02877
Beta virt. eigenvalues --	1.07284	1.08212	1.09675	1.09796	1.12225
Beta virt. eigenvalues --	1.12308	1.13004	1.14011	1.15805	1.16726
Beta virt. eigenvalues --	1.18160	1.19521	1.20249	1.20842	1.21911
Beta virt. eigenvalues --	1.23229	1.23647	1.23668	1.23814	1.24171
Beta virt. eigenvalues --	1.25409	1.25714	1.27061	1.29808	1.29917
Beta virt. eigenvalues --	1.32059	1.32304	1.34811	1.35674	1.39501
Beta virt. eigenvalues --	1.39643	1.42510	1.47298	1.48235	1.54879

TBpyT

Alpha occ. eigenvalues --	-89.03472	-89.02472	-89.02471	-19.23762	-19.23761
Alpha occ. eigenvalues --	-19.23522	-19.23522	-14.38377	-14.38372	-10.28834
Alpha occ. eigenvalues --	-10.28834	-10.28538	-10.28538	-10.28410	-10.28410
Alpha occ. eigenvalues --	-10.28358	-10.28358	-10.26230	-10.26222	-10.25485
Alpha occ. eigenvalues --	-10.25461	-10.23756	-10.23756	-10.22998	-10.22991
Alpha occ. eigenvalues --	-10.20527	-10.20478	-8.03013	-8.01981	-8.01980
Alpha occ. eigenvalues --	-5.97153	-5.96641	-5.96063	-5.96022	-5.96022
Alpha occ. eigenvalues --	-5.95616	-5.95615	-5.95051	-5.95051	-1.09201
Alpha occ. eigenvalues --	-1.09199	-1.05343	-1.05342	-0.97343	-0.89003
Alpha occ. eigenvalues --	-0.88746	-0.86327	-0.83824	-0.78311	-0.78306
Alpha occ. eigenvalues --	-0.77633	-0.74303	-0.73401	-0.71270	-0.70926
Alpha occ. eigenvalues --	-0.70457	-0.66309	-0.64307	-0.63073	-0.61237
Alpha occ. eigenvalues --	-0.59452	-0.57259	-0.57028	-0.54361	-0.54216
Alpha occ. eigenvalues --	-0.53965	-0.53065	-0.50106	-0.49740	-0.48755
Alpha occ. eigenvalues --	-0.47780	-0.46778	-0.45561	-0.45296	-0.44340
Alpha occ. eigenvalues --	-0.44266	-0.44048	-0.43393	-0.42904	-0.42384
Alpha occ. eigenvalues --	-0.41552	-0.40456	-0.39940	-0.39423	-0.39103
Alpha occ. eigenvalues --	-0.38505	-0.37308	-0.35377	-0.35255	-0.34475
Alpha occ. eigenvalues --	-0.34462	-0.34138	-0.33838	-0.33039	-0.32965
Alpha occ. eigenvalues --	-0.31374	-0.30649	-0.30103	-0.29901	-0.27934
Alpha occ. eigenvalues --	-0.26010	-0.25267	-0.25020	-0.22604	-0.21861
Alpha occ. eigenvalues --	-0.21839	-0.17661	-0.12369		
Alpha virt. eigenvalues --	-0.04850	-0.01732	-0.00223	0.02274	0.02362
Alpha virt. eigenvalues --	0.03528	0.03976	0.04655	0.05610	0.06372
Alpha virt. eigenvalues --	0.07417	0.07603	0.08914	0.09553	0.09591
Alpha virt. eigenvalues --	0.10087	0.10228	0.10658	0.11407	0.12051
Alpha virt. eigenvalues --	0.12157	0.12662	0.13689	0.13903	0.14424
Alpha virt. eigenvalues --	0.15600	0.16094	0.18987	0.19738	0.19932
Alpha virt. eigenvalues --	0.20311	0.21207	0.22164	0.22559	0.25918
Alpha virt. eigenvalues --	0.26809	0.26927	0.28752	0.29339	0.30396
Alpha virt. eigenvalues --	0.30780	0.31204	0.32223	0.32403	0.35633
Alpha virt. eigenvalues --	0.36183	0.36904	0.37031	0.38268	0.38688
Alpha virt. eigenvalues --	0.40005	0.40281	0.40986	0.43791	0.45178
Alpha virt. eigenvalues --	0.45212	0.45626	0.45960	0.46690	0.47107
Alpha virt. eigenvalues --	0.47199	0.48181	0.48488	0.48584	0.49745
Alpha virt. eigenvalues --	0.49754	0.50701	0.50987	0.51653	0.52275
Alpha virt. eigenvalues --	0.52407	0.52574	0.52945	0.54604	0.55671
Alpha virt. eigenvalues --	0.55677	0.56449	0.56865	0.56995	0.57464
Alpha virt. eigenvalues --	0.58258	0.58737	0.60131	0.60514	0.61201
Alpha virt. eigenvalues --	0.62176	0.63666	0.64404	0.65001	0.65478
Alpha virt. eigenvalues --	0.66368	0.66900	0.66978	0.66996	0.67122
Alpha virt. eigenvalues --	0.67395	0.68051	0.68428	0.68760	0.68809
Alpha virt. eigenvalues --	0.68959	0.69540	0.69704	0.70582	0.71084
Alpha virt. eigenvalues --	0.71738	0.72335	0.72586	0.73192	0.74049

Alpha virt. eigenvalues -- 0.74390 0.74974 0.75299 0.76762 0.76957
Alpha virt. eigenvalues -- 0.77205 0.79585 0.79870 0.80304 0.81125
Alpha virt. eigenvalues -- 0.81228 0.82045 0.82747 0.84187 0.84205
Alpha virt. eigenvalues -- 0.86644 0.87336 0.87486 0.88080 0.91046
Alpha virt. eigenvalues -- 0.91325 0.93987 0.97443 0.97939 1.01524
Alpha virt. eigenvalues -- 1.02816 1.04960 1.06459 1.07327 1.09786
Alpha virt. eigenvalues -- 1.09893 1.12531 1.12940 1.13587 1.15426
Alpha virt. eigenvalues -- 1.16272 1.17562 1.18628 1.20871 1.24127
Alpha virt. eigenvalues -- 1.25638 1.26060 1.28820 1.29122 1.33168
Alpha virt. eigenvalues -- 1.35176 1.35254 1.37505 1.38224 1.41189
Alpha virt. eigenvalues -- 1.44793 1.45215 1.53313 1.59643 1.61311
Alpha virt. eigenvalues -- 1.63855 1.72444 1.74051 1.76701 1.79206
Alpha virt. eigenvalues -- 1.80162
Beta occ. eigenvalues -- -89.03379 -89.02471 -89.02470 -19.23718 -19.23717
Beta occ. eigenvalues -- -19.23533 -19.23533 -14.37762 -14.37756 -10.28884
Beta occ. eigenvalues -- -10.28884 -10.28416 -10.28416 -10.28411 -10.28411
Beta occ. eigenvalues -- -10.28357 -10.28357 -10.26311 -10.26303 -10.25583
Beta occ. eigenvalues -- -10.25560 -10.23649 -10.23649 -10.22803 -10.22796
Beta occ. eigenvalues -- -10.20438 -10.20390 -8.02917 -8.01980 -8.01980
Beta occ. eigenvalues -- -5.97113 -5.96603 -5.96022 -5.96022 -5.95715
Beta occ. eigenvalues -- -5.95616 -5.95616 -5.95047 -5.95047 -1.09162
Beta occ. eigenvalues -- -1.09160 -1.05306 -1.05304 -0.96658 -0.88857
Beta occ. eigenvalues -- -0.88660 -0.85440 -0.83440 -0.78292 -0.78263
Beta occ. eigenvalues -- -0.77309 -0.74221 -0.73053 -0.71077 -0.70860
Beta occ. eigenvalues -- -0.70350 -0.66186 -0.64244 -0.62584 -0.60975
Beta occ. eigenvalues -- -0.59293 -0.57108 -0.56929 -0.54323 -0.54175
Beta occ. eigenvalues -- -0.53927 -0.53008 -0.50012 -0.49687 -0.48686
Beta occ. eigenvalues -- -0.47595 -0.46647 -0.45332 -0.45200 -0.44200
Beta occ. eigenvalues -- -0.44166 -0.43957 -0.43359 -0.42854 -0.41601
Beta occ. eigenvalues -- -0.41449 -0.40200 -0.39833 -0.39287 -0.38963
Beta occ. eigenvalues -- -0.38431 -0.37188 -0.35162 -0.34880 -0.34345
Beta occ. eigenvalues -- -0.33948 -0.33852 -0.33549 -0.32699 -0.32457
Beta occ. eigenvalues -- -0.30986 -0.30440 -0.29796 -0.29016 -0.27436
Beta occ. eigenvalues -- -0.24578 -0.24111 -0.23792 -0.22156 -0.21724
Beta occ. eigenvalues -- -0.21622 -0.16305
Beta virt. eigenvalues -- -0.08379 -0.02837 -0.01427 0.00291 0.02593
Beta virt. eigenvalues -- 0.03281 0.03875 0.04342 0.04719 0.05658
Beta virt. eigenvalues -- 0.06508 0.07566 0.07878 0.09154 0.09635
Beta virt. eigenvalues -- 0.09680 0.10101 0.10300 0.11002 0.11434
Beta virt. eigenvalues -- 0.12057 0.12213 0.12852 0.13722 0.13953
Beta virt. eigenvalues -- 0.14490 0.15628 0.16126 0.19461 0.19975
Beta virt. eigenvalues -- 0.20127 0.20326 0.21343 0.22264 0.22685
Beta virt. eigenvalues -- 0.25955 0.26939 0.27039 0.28963 0.29453
Beta virt. eigenvalues -- 0.30501 0.30907 0.31336 0.32432 0.32521
Beta virt. eigenvalues -- 0.35685 0.36237 0.36916 0.37131 0.38432
Beta virt. eigenvalues -- 0.38872 0.40033 0.40474 0.41075 0.44074

Beta virt. eigenvalues -- 0.45308 0.45422 0.45750 0.45997 0.46727
Beta virt. eigenvalues -- 0.47227 0.47311 0.48218 0.48509 0.48750
Beta virt. eigenvalues -- 0.49829 0.49896 0.50789 0.51045 0.51763
Beta virt. eigenvalues -- 0.52309 0.52688 0.52920 0.53052 0.54753
Beta virt. eigenvalues -- 0.55720 0.55723 0.56696 0.56953 0.57072
Beta virt. eigenvalues -- 0.57586 0.58517 0.58833 0.60183 0.60558
Beta virt. eigenvalues -- 0.61352 0.62266 0.63728 0.64448 0.65248
Beta virt. eigenvalues -- 0.65706 0.66777 0.66917 0.67007 0.67140
Beta virt. eigenvalues -- 0.67247 0.67441 0.68160 0.68497 0.68792
Beta virt. eigenvalues -- 0.68835 0.69028 0.69567 0.69802 0.70708
Beta virt. eigenvalues -- 0.71311 0.72047 0.72509 0.72744 0.73360
Beta virt. eigenvalues -- 0.74523 0.74675 0.75330 0.75614 0.76831
Beta virt. eigenvalues -- 0.77064 0.77391 0.79662 0.79917 0.80602
Beta virt. eigenvalues -- 0.81268 0.81331 0.82193 0.82884 0.84317
Beta virt. eigenvalues -- 0.84326 0.86844 0.88227 0.89117 0.89162
Beta virt. eigenvalues -- 0.91253 0.91408 0.94080 0.97545 0.98140
Beta virt. eigenvalues -- 1.01596 1.02877 1.05080 1.06584 1.07496
Beta virt. eigenvalues -- 1.09866 1.09915 1.12635 1.13026 1.13631
Beta virt. eigenvalues -- 1.15484 1.16425 1.17627 1.18750 1.20990
Beta virt. eigenvalues -- 1.24204 1.25804 1.26268 1.28895 1.29182
Beta virt. eigenvalues -- 1.33249 1.35224 1.35471 1.37625 1.38496
Beta virt. eigenvalues -- 1.41259 1.45023 1.45511 1.53349 1.59662
Beta virt. eigenvalues -- 1.61322 1.63899 1.72467 1.74071 1.76743
Beta virt. eigenvalues -- 1.79254 1.80213

EBE

Alpha occ. eigenvalues -- -89.03472 -89.02472 -89.02471 -19.23762 -19.23761
Alpha occ. eigenvalues -- -19.23522 -19.23522 -14.38377 -14.38372 -10.28834
Alpha occ. eigenvalues -- -10.28834 -10.28538 -10.28538 -10.28410 -10.28410
Alpha occ. eigenvalues -- -10.28358 -10.28358 -10.26230 -10.26222 -10.25485
Alpha occ. eigenvalues -- -10.25461 -10.23756 -10.23756 -10.22998 -10.22991
Alpha occ. eigenvalues -- -10.20527 -10.20478 -8.03013 -8.01981 -8.01980
Alpha occ. eigenvalues -- -5.97153 -5.96641 -5.96063 -5.96022 -5.96022
Alpha occ. eigenvalues -- -5.95616 -5.95615 -5.95051 -5.95051 -1.09201
Alpha occ. eigenvalues -- -1.09199 -1.05343 -1.05342 -0.97343 -0.89003
Alpha occ. eigenvalues -- -0.88746 -0.86327 -0.83824 -0.78311 -0.78306
Alpha occ. eigenvalues -- -0.77633 -0.74303 -0.73401 -0.71270 -0.70926
Alpha occ. eigenvalues -- -0.70457 -0.66309 -0.64307 -0.63073 -0.61237
Alpha occ. eigenvalues -- -0.59452 -0.57259 -0.57028 -0.54361 -0.54216
Alpha occ. eigenvalues -- -0.53965 -0.53065 -0.50106 -0.49740 -0.48755
Alpha occ. eigenvalues -- -0.47780 -0.46778 -0.45561 -0.45296 -0.44340
Alpha occ. eigenvalues -- -0.44266 -0.44048 -0.43393 -0.42904 -0.42384
Alpha occ. eigenvalues -- -0.41552 -0.40456 -0.39940 -0.39423 -0.39103
Alpha occ. eigenvalues -- -0.38505 -0.37308 -0.35377 -0.35255 -0.34475
Alpha occ. eigenvalues -- -0.34462 -0.34138 -0.33838 -0.33039 -0.32965
Alpha occ. eigenvalues -- -0.31374 -0.30649 -0.30103 -0.29901 -0.27934

Alpha occ. eigenvalues --   -0.26010 -0.25267 -0.25020 -0.22604 -0.21861
Alpha occ. eigenvalues --   -0.21839 -0.17661 -0.12369
Alpha virt. eigenvalues --   -0.04850 -0.01732 -0.00223   0.02274   0.02362
Alpha virt. eigenvalues --   0.03528   0.03976   0.04655   0.05610   0.06372
Alpha virt. eigenvalues --   0.07417   0.07603   0.08914   0.09553   0.09591
Alpha virt. eigenvalues --   0.10087   0.10228   0.10658   0.11407   0.12051
Alpha virt. eigenvalues --   0.12157   0.12662   0.13689   0.13903   0.14424
Alpha virt. eigenvalues --   0.15600   0.16094   0.18987   0.19738   0.19932
Alpha virt. eigenvalues --   0.20311   0.21207   0.22164   0.22559   0.25918
Alpha virt. eigenvalues --   0.26809   0.26927   0.28752   0.29339   0.30396
Alpha virt. eigenvalues --   0.30780   0.31204   0.32223   0.32403   0.35633
Alpha virt. eigenvalues --   0.36183   0.36904   0.37031   0.38268   0.38688
Alpha virt. eigenvalues --   0.40005   0.40281   0.40986   0.43791   0.45178
Alpha virt. eigenvalues --   0.45212   0.45626   0.45960   0.46690   0.47107
Alpha virt. eigenvalues --   0.47199   0.48181   0.48488   0.48584   0.49745
Alpha virt. eigenvalues --   0.49754   0.50701   0.50987   0.51653   0.52275
Alpha virt. eigenvalues --   0.52407   0.52574   0.52945   0.54604   0.55671
Alpha virt. eigenvalues --   0.55677   0.56449   0.56865   0.56995   0.57464
Alpha virt. eigenvalues --   0.58258   0.58737   0.60131   0.60514   0.61201
Alpha virt. eigenvalues --   0.62176   0.63666   0.64404   0.65001   0.65478
Alpha virt. eigenvalues --   0.66368   0.66900   0.66978   0.66996   0.67122
Alpha virt. eigenvalues --   0.67395   0.68051   0.68428   0.68760   0.68809
Alpha virt. eigenvalues --   0.68959   0.69540   0.69704   0.70582   0.71084
Alpha virt. eigenvalues --   0.71738   0.72335   0.72586   0.73192   0.74049
Alpha virt. eigenvalues --   0.74390   0.74974   0.75299   0.76762   0.76957
Alpha virt. eigenvalues --   0.77205   0.79585   0.79870   0.80304   0.81125
Alpha virt. eigenvalues --   0.81228   0.82045   0.82747   0.84187   0.84205
Alpha virt. eigenvalues --   0.86644   0.87336   0.87486   0.88080   0.91046
Alpha virt. eigenvalues --   0.91325   0.93987   0.97443   0.97939   1.01524
Alpha virt. eigenvalues --   1.02816   1.04960   1.06459   1.07327   1.09786
Alpha virt. eigenvalues --   1.09893   1.12531   1.12940   1.13587   1.15426
Alpha virt. eigenvalues --   1.16272   1.17562   1.18628   1.20871   1.24127
Alpha virt. eigenvalues --   1.25638   1.26060   1.28820   1.29122   1.33168
Alpha virt. eigenvalues --   1.35176   1.35254   1.37505   1.38224   1.41189
Alpha virt. eigenvalues --   1.44793   1.45215   1.53313   1.59643   1.61311
Alpha virt. eigenvalues --   1.63855   1.72444   1.74051   1.76701   1.79206
Alpha virt. eigenvalues --   1.80162
Beta occ. eigenvalues -- -89.03379 -89.02471 -89.02470 -19.23718 -19.23717
Beta occ. eigenvalues -- -19.23533 -19.23533 -14.37762 -14.37756 -10.28884
Beta occ. eigenvalues -- -10.28884 -10.28416 -10.28416 -10.28411 -10.28411
Beta occ. eigenvalues -- -10.28357 -10.28357 -10.26311 -10.26303 -10.25583
Beta occ. eigenvalues -- -10.25560 -10.23649 -10.23649 -10.22803 -10.22796
Beta occ. eigenvalues -- -10.20438 -10.20390 -8.02917 -8.01980 -8.01980
Beta occ. eigenvalues --   -5.97113 -5.96603 -5.96022 -5.96022 -5.95715
Beta occ. eigenvalues --   -5.95616 -5.95616 -5.95047 -5.95047 -1.09162
Beta occ. eigenvalues --   -1.09160 -1.05306 -1.05304 -0.96658 -0.88857

Beta occ. eigenvalues --	-0.88660	-0.85440	-0.83440	-0.78292	-0.78263
Beta occ. eigenvalues --	-0.77309	-0.74221	-0.73053	-0.71077	-0.70860
Beta occ. eigenvalues --	-0.70350	-0.66186	-0.64244	-0.62584	-0.60975
Beta occ. eigenvalues --	-0.59293	-0.57108	-0.56929	-0.54323	-0.54175
Beta occ. eigenvalues --	-0.53927	-0.53008	-0.50012	-0.49687	-0.48686
Beta occ. eigenvalues --	-0.47595	-0.46647	-0.45332	-0.45200	-0.44200
Beta occ. eigenvalues --	-0.44166	-0.43957	-0.43359	-0.42854	-0.41601
Beta occ. eigenvalues --	-0.41449	-0.40200	-0.39833	-0.39287	-0.38963
Beta occ. eigenvalues --	-0.38431	-0.37188	-0.35162	-0.34880	-0.34345
Beta occ. eigenvalues --	-0.33948	-0.33852	-0.33549	-0.32699	-0.32457
Beta occ. eigenvalues --	-0.30986	-0.30440	-0.29796	-0.29016	-0.27436
Beta occ. eigenvalues --	-0.24578	-0.24111	-0.23792	-0.22156	-0.21724
Beta occ. eigenvalues --	-0.21622	-0.16305			
Beta virt. eigenvalues --	-0.08379	-0.02837	-0.01427	0.00291	0.02593
Beta virt. eigenvalues --	0.03281	0.03875	0.04342	0.04719	0.05658
Beta virt. eigenvalues --	0.06508	0.07566	0.07878	0.09154	0.09635
Beta virt. eigenvalues --	0.09680	0.10101	0.10300	0.11002	0.11434
Beta virt. eigenvalues --	0.12057	0.12213	0.12852	0.13722	0.13953
Beta virt. eigenvalues --	0.14490	0.15628	0.16126	0.19461	0.19975
Beta virt. eigenvalues --	0.20127	0.20326	0.21343	0.22264	0.22685
Beta virt. eigenvalues --	0.25955	0.26939	0.27039	0.28963	0.29453
Beta virt. eigenvalues --	0.30501	0.30907	0.31336	0.32432	0.32521
Beta virt. eigenvalues --	0.35685	0.36237	0.36916	0.37131	0.38432
Beta virt. eigenvalues --	0.38872	0.40033	0.40474	0.41075	0.44074
Beta virt. eigenvalues --	0.45308	0.45422	0.45750	0.45997	0.46727
Beta virt. eigenvalues --	0.47227	0.47311	0.48218	0.48509	0.48750
Beta virt. eigenvalues --	0.49829	0.49896	0.50789	0.51045	0.51763
Beta virt. eigenvalues --	0.52309	0.52688	0.52920	0.53052	0.54753
Beta virt. eigenvalues --	0.55720	0.55723	0.56696	0.56953	0.57072
Beta virt. eigenvalues --	0.57586	0.58517	0.58833	0.60183	0.60558
Beta virt. eigenvalues --	0.61352	0.62266	0.63728	0.64448	0.65248
Beta virt. eigenvalues --	0.65706	0.66777	0.66917	0.67007	0.67140
Beta virt. eigenvalues --	0.67247	0.67441	0.68160	0.68497	0.68792
Beta virt. eigenvalues --	0.68835	0.69028	0.69567	0.69802	0.70708
Beta virt. eigenvalues --	0.71311	0.72047	0.72509	0.72744	0.73360
Beta virt. eigenvalues --	0.74523	0.74675	0.75330	0.75614	0.76831
Beta virt. eigenvalues --	0.77064	0.77391	0.79662	0.79917	0.80602
Beta virt. eigenvalues --	0.81268	0.81331	0.82193	0.82884	0.84317
Beta virt. eigenvalues --	0.84326	0.86844	0.88227	0.89117	0.89162
Beta virt. eigenvalues --	0.91253	0.91408	0.94080	0.97545	0.98140
Beta virt. eigenvalues --	1.01596	1.02877	1.05080	1.06584	1.07496
Beta virt. eigenvalues --	1.09866	1.09915	1.12635	1.13026	1.13631
Beta virt. eigenvalues --	1.15484	1.16425	1.17627	1.18750	1.20990
Beta virt. eigenvalues --	1.24204	1.25804	1.26268	1.28895	1.29182
Beta virt. eigenvalues --	1.33249	1.35224	1.35471	1.37625	1.38496
Beta virt. eigenvalues --	1.41259	1.45023	1.45511	1.53349	1.59662

Beta virt. eigenvalues -- 1.61322 1.63899 1.72467 1.74071 1.76743
Beta virt. eigenvalues -- 1.79254 1.80213

Series 2

Benchmarking Plots

Table DFT-SI.2: Benchmarking for Series 2 (PBP, P_2BP_2, PBpyP, PyBPy, FBF). For each structure the computational wavelength maxima were compared to those generated from experiment and plotted to identify the best basis-set/functional pairing that would work for all 5 systems. Unfortunately, there were only 2 plots which included the higher wavelength values (the B98/SV plot is provided for comparison). For this series the B3LYP/6-31G* pairing was found to possess the strongest correlation with an R^2 value of 0.9727.

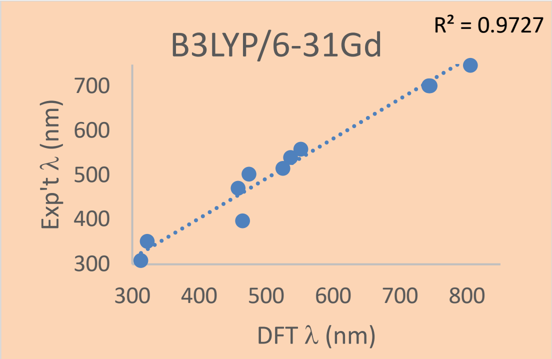	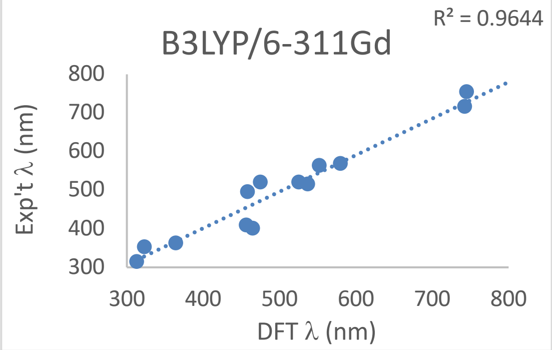
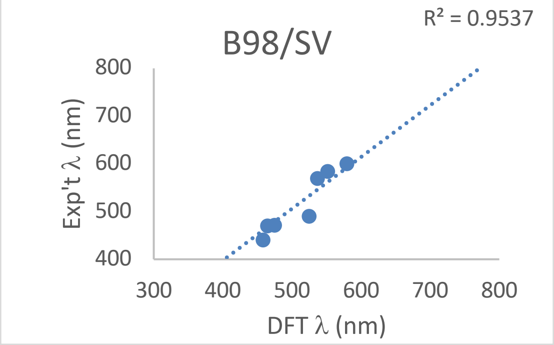	

UV-Vis Experimental vs DFT Overlays + FMO Band Diagrams

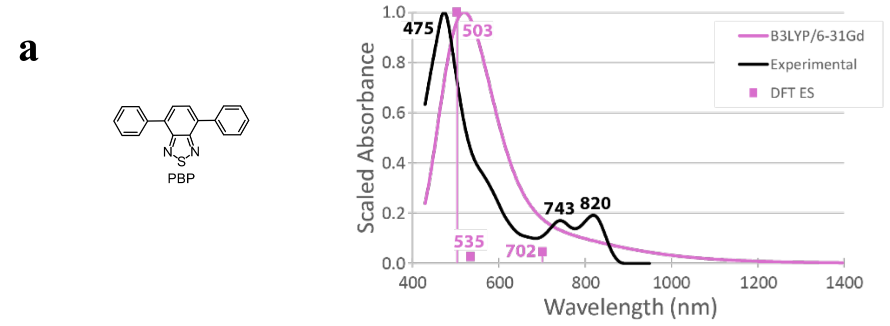

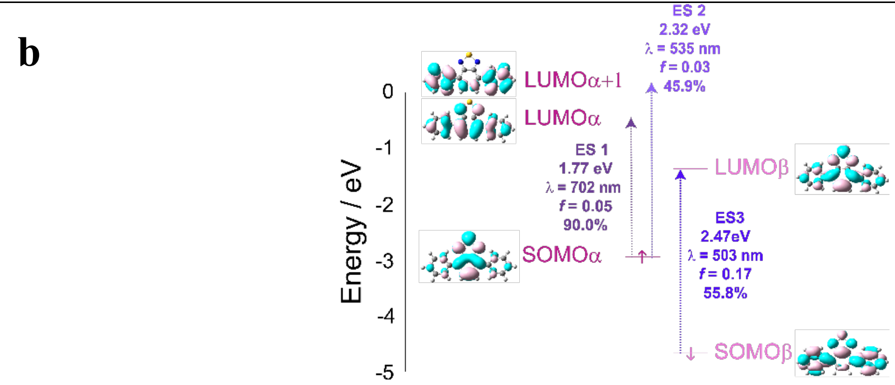

Figure DFT-SI.6: a. An overlay of the experimental (black) and benchmarked computational (pink) UV-visible absorption spectra is shown. The height of the lines below are indicative of oscillator strengths and the numbers correspond to the peak maxima (black/experimental) or excited state value in nm. **b.** A band diagram with corresponding energy levels and frontier molecular orbitals is given. The excited state energy (eV), oscillator strength (*f*), and percentage of contribution of the indicated transition is provided for the lowest 3 lying excited states for PBP.

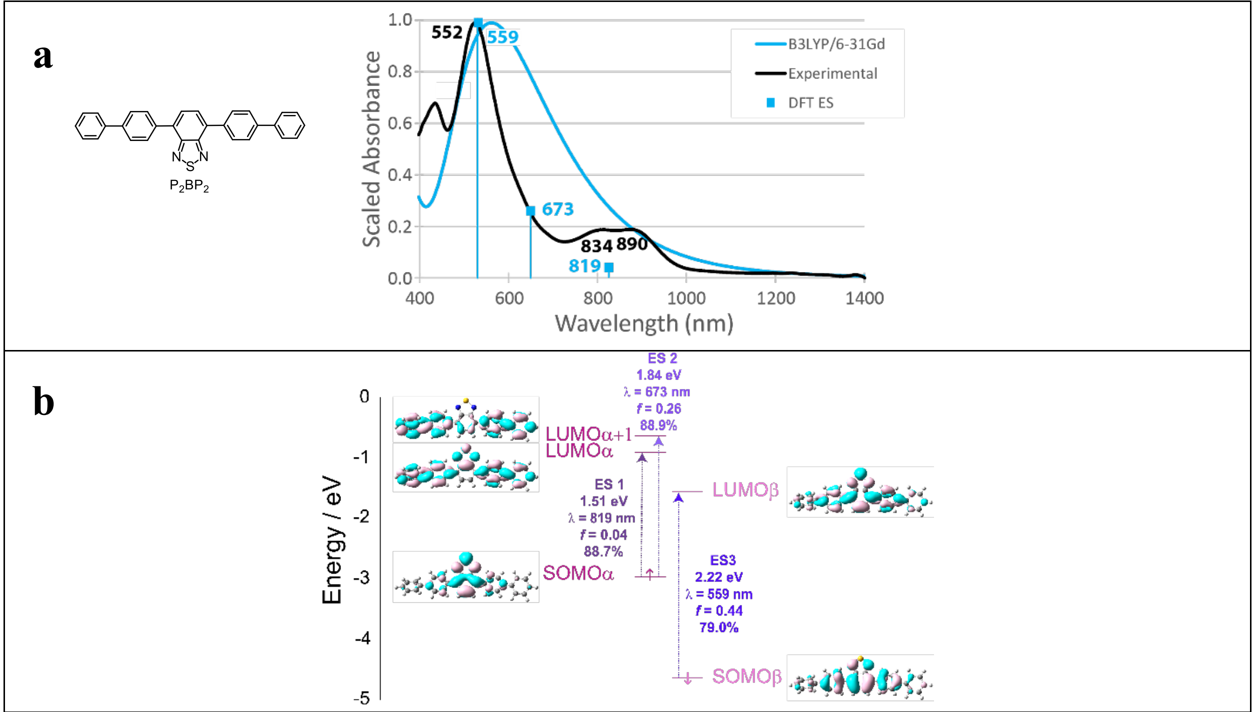

Figure DFT-SI.7: a. An overlay of the experimental (black) and benchmarked computational (blue) UV-visible absorption spectra is shown. The height of the lines below are indicative of oscillator strengths and the numbers correspond to the peak maxima (black/experimental) or excited state value in nm. **b.** A band diagram with corresponding energy levels and frontier molecular orbitals is given. The excited state energy (eV), oscillator strength (*f*), and percentage of contribution of the indicated transition is provided for the lowest 3 lying excited states for P_2BP_2.

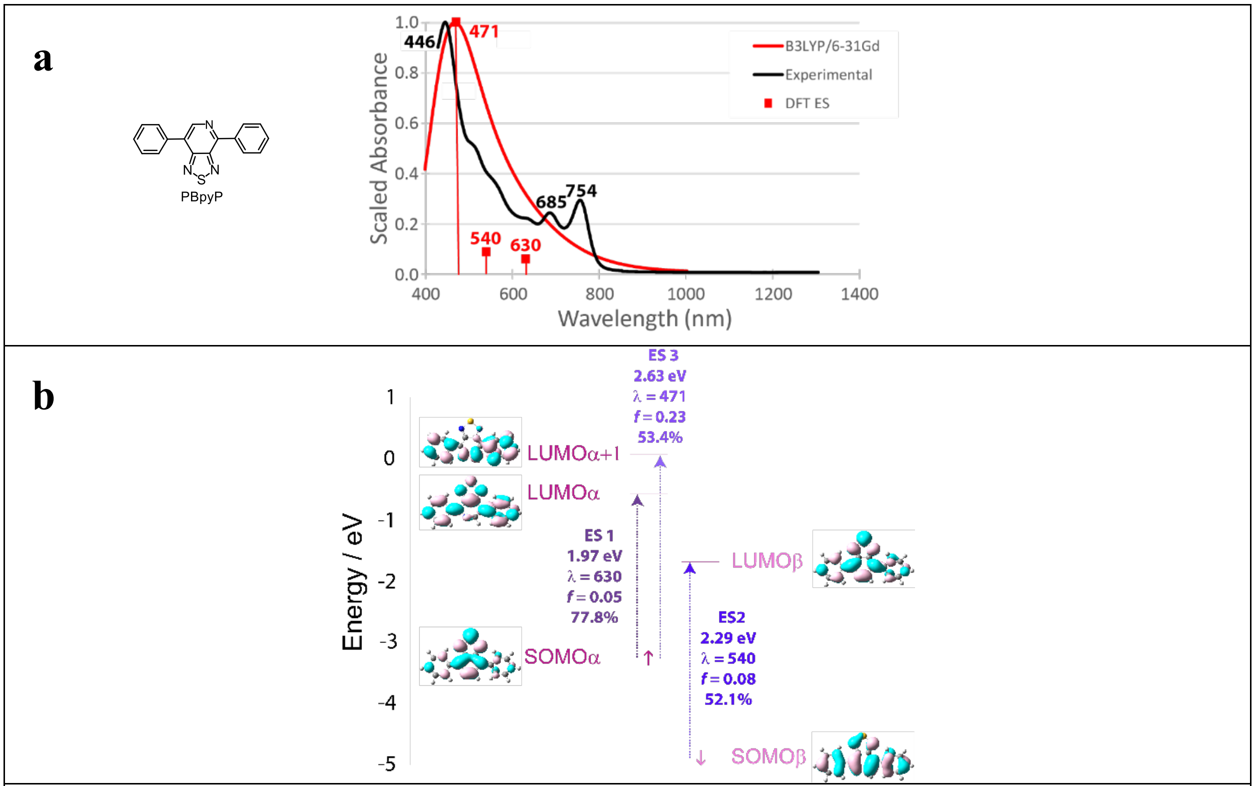

Figure DFT-SI.8: a. An overlay of the experimental (black) and benchmarked computational (red) UV-visible absorption spectra is shown. The height of the lines below are indicative of oscillator strengths and the numbers correspond to the peak maxima (black/experimental) or excited state value in nm. **b.** A band diagram with corresponding energy levels and frontier molecular orbitals is given. The excited state energy (eV), oscillator strength (*f*), and percentage of contribution of the indicated transition is provided for the lowest 3 lying excited states for PBpyP.

a

b

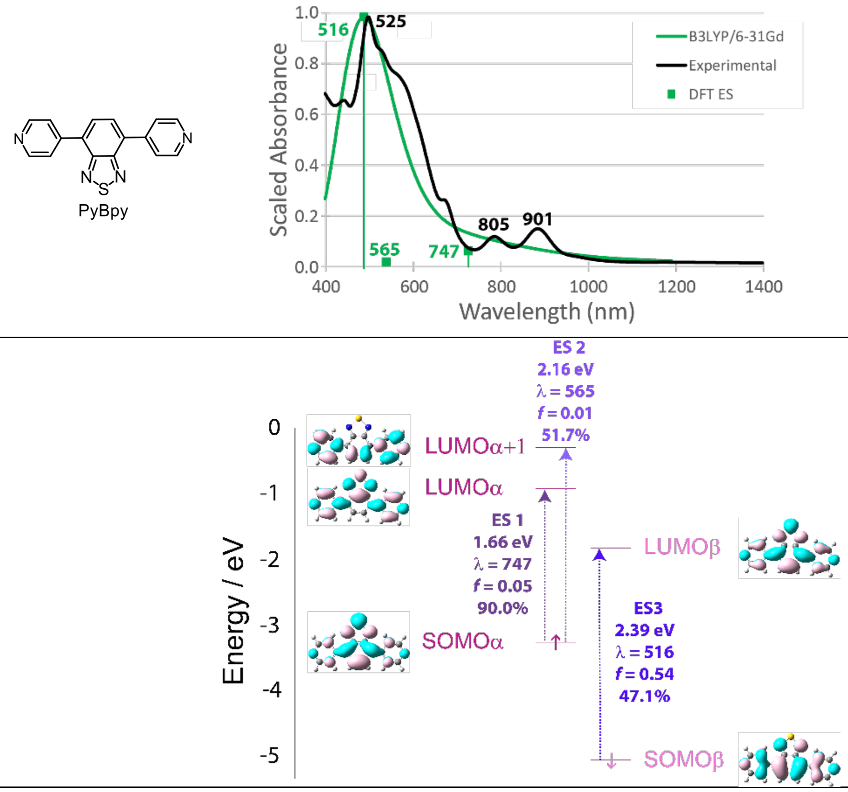

Figure DFT-SI.9: a. An overlay of the experimental (black) and benchmarked computational (green) UV-visible absorption spectra is shown. The height of the lines below are indicative of oscillator strengths and the numbers correspond to the peak maxima (black/experimental) or excited state value in nm. **b.** A band diagram with corresponding energy levels and frontier molecular orbitals is given. The excited state energy (eV), oscillator strength (*f*), and percentage of contribution of the indicated transition is provided for the lowest 3 lying excited states for PyBPy.

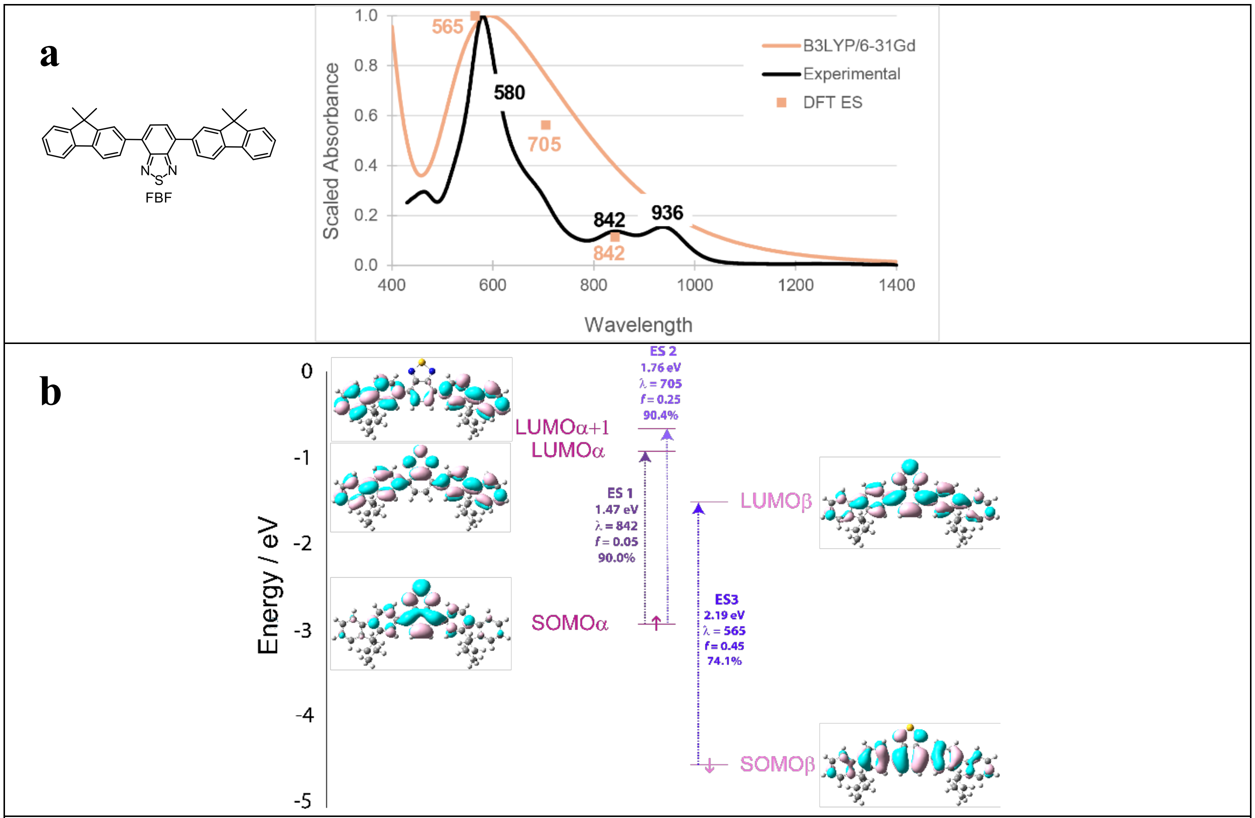

Figure DFT-SI.10: a. An overlay of the experimental (black) and benchmarked computational (orange) UV-visible absorption spectra is shown. The height of the lines below are indicative of oscillator strengths and the numbers correspond to the peak maxima (black/experimental) or excited state value in nm. **b.** A band diagram with corresponding energy levels and frontier molecular orbitals is given. The excited state energy (eV), oscillator strength (*f*), and percentage of contribution of the indicated transition is provided for the lowest 3 lying excited states for FBF.

Cartesian Coordinates

PBP

C	0.73511	0.74831	0.01476
C	-0.73511	0.74831	-0.01476
C	-1.46566	-0.48324	-0.00621
C	-0.69578	-1.66571	0.00121
C	0.69578	-1.66571	-0.00121
C	1.46566	-0.48324	0.00621
H	-1.20737	-2.62371	-0.02014
H	1.20737	-2.62371	0.02014
S	0.	3.07913	0.
N	-1.28345	1.9809	-0.02654
N	1.28345	1.9809	0.02654
C	2.94219	-0.55449	-0.01025
C	3.75951	0.43159	0.58599
C	3.60152	-1.64975	-0.61439
C	5.14974	0.31811	0.58895
H	3.28926	1.29178	1.04604
C	4.99073	-1.76146	-0.61095
H	3.0152	-2.41314	-1.11752
C	5.77937	-0.77834	-0.00633
H	5.74597	1.09358	1.06492
H	5.45941	-2.61609	-1.09349
H	6.86299	-0.86184	-0.00611
C	-2.94219	-0.55449	0.01025
C	-3.75951	0.43159	-0.58599
C	-3.60152	-1.64975	0.61439
C	-5.14974	0.31811	-0.58894
H	-3.28926	1.29178	-1.04603
C	-4.99073	-1.76146	0.61095
H	-3.0152	-2.41314	1.11752
C	-5.77937	-0.77834	0.00633
H	-5.74597	1.09358	-1.06492
H	-5.45941	-2.6161	1.09348
H	-6.86299	-0.86184	0.0061

P_2BP_2

C	-0.73574	1.10216	-0.05683
C	0.73623	1.10019	-0.08569
C	1.47039	-0.12825	0.00449
C	0.69505	-1.3061	0.08824
C	-0.69493	-1.30614	0.08545
C	-1.46989	-0.12727	0.01526
H	1.20209	-2.2655	0.12842

H	-1.20211	-2.26252	0.17065
S	0.00022	3.42502	-0.22734
N	1.27964	2.32901	-0.17939
N	-1.27929	2.33254	-0.12812
C	-2.94207	-0.20033	0.00297
C	-3.60734	-1.35776	-0.46592
C	-3.76983	0.84675	0.46873
C	-4.99278	-1.46686	-0.45712
H	-3.0278	-2.18476	-0.86469
C	-5.15646	0.73289	0.4797
H	-3.30938	1.76284	0.81587
C	-5.80981	-0.4249	0.01992
H	-5.4485	-2.38653	-0.81492
H	-5.74626	1.57437	0.83478
C	2.94205	-0.19972	0.02637
C	3.60687	-1.2908	0.63532
C	3.77099	0.78118	-0.56501
C	4.99193	-1.40222	0.63847
H	3.02763	-2.05124	1.15015
C	5.15739	0.6664	-0.56046
H	3.31077	1.63835	-1.03972
C	5.81013	-0.42703	0.03745
H	5.44884	-2.24486	1.15113
H	5.74568	1.43149	-1.06109
C	7.28802	-0.54423	0.04102
C	7.91816	-1.80194	-0.02284
C	8.10962	0.59753	0.10695
C	9.30843	-1.91357	-0.01886
H	7.31194	-2.70011	-0.10053
C	9.49998	0.4869	0.10594
H	7.65367	1.5806	0.18345
C	10.10828	-0.76956	0.04433
H	9.76769	-2.89717	-0.07542
H	10.10947	1.3851	0.16379
H	11.19139	-0.85602	0.04551
C	-7.28841	-0.53971	0.02998
C	-8.058	0.06645	1.04142
C	-7.97046	-1.25871	-0.97024
C	-9.4487	-0.03962	1.05127
H	-7.55932	0.60675	1.84118
C	-9.36088	-1.36872	-0.95874
H	-7.40691	-1.71638	-1.77844
C	-10.10893	-0.7589	0.05167
H	-10.01701	0.43326	1.84812

H	-9.86142	-1.92456	-1.74746
H	-11.19219	-0.84301	0.05998

PBpyP

C	-0.7376	0.75275	-0.02809
C	0.7376	0.75275	-0.02809
C	1.47444	-0.477	0.06315
C	0.69335	-1.65142	0.17992
C	-0.69335	-1.65142	0.17992
C	-1.47444	-0.477	0.06315
H	1.19452	-2.60335	0.32221
H	-1.19452	-2.60335	0.32221
S	0.	3.0669	-0.25069
N	1.27244	1.97779	-0.14681
N	-1.27244	1.97779	-0.14681
C	-2.93841	-0.55463	0.04239
C	-3.61173	-1.7645	-0.25863
C	-3.78963	0.53923	0.32774
C	-4.99889	-1.82118	-0.24643
H	-3.06	-2.65765	-0.53094
C	-5.1702	0.37224	0.31503
H	-3.36743	1.50837	0.55574
H	-5.49796	-2.75993	-0.48637
H	-5.8112	1.22335	0.54554
C	2.93841	-0.55463	0.04239
C	3.78963	0.53923	0.32774
C	3.61173	-1.7645	-0.25863
C	5.1702	0.37224	0.31502
H	3.36743	1.50837	0.55574
C	4.99889	-1.82118	-0.24642
H	3.06	-2.65765	-0.53094
H	5.8112	1.22335	0.54554
H	5.49796	-2.75993	-0.48637
N	5.80363	-0.7805	0.03665
N	-5.80363	-0.7805	0.03665

PyBPy

C	-0.7376	0.75275	-0.02809
C	0.7376	0.75275	-0.02809
C	1.47444	-0.477	0.06315
C	0.69335	-1.65142	0.17992
C	-0.69335	-1.65142	0.17992
C	-1.47444	-0.477	0.06315
H	1.19452	-2.60335	0.32221

H	-1.19452	-2.60335	0.32221
S	0.	3.0669	-0.25069
N	1.27244	1.97779	-0.14681
N	-1.27244	1.97779	-0.14681
C	-2.93841	-0.55463	0.04239
C	-3.61173	-1.7645	-0.25863
C	-3.78963	0.53923	0.32774
C	-4.99889	-1.82118	-0.24643
H	-3.06	-2.65765	-0.53094
C	-5.1702	0.37224	0.31503
H	-3.36743	1.50837	0.55574
H	-5.49796	-2.75993	-0.48637
H	-5.8112	1.22335	0.54554
C	2.93841	-0.55463	0.04239
C	3.78963	0.53923	0.32774
C	3.61173	-1.7645	-0.25863
C	5.1702	0.37224	0.31502
H	3.36743	1.50837	0.55574
C	4.99889	-1.82118	-0.24642
H	3.06	-2.65765	-0.53094
H	5.8112	1.22335	0.54554
H	5.49796	-2.75993	-0.48637
N	5.80363	-0.7805	0.03665
N	-5.80363	-0.7805	0.03665

FBF

C	-0.72984	-2.20567	-0.09333
C	0.72984	-2.20567	0.09332
C	1.45968	-0.97395	0.16465
C	0.69148	0.2073	0.07371
C	-0.69148	0.2073	-0.07372
C	-1.45968	-0.97395	-0.16466
H	1.19552	1.16627	0.15078
H	-1.19552	1.16627	-0.15079
S	0.	-4.535	0.
N	1.27116	-3.43802	0.16145
N	-1.27116	-3.43802	-0.16145
C	-2.9264	-0.90144	-0.30738
C	-3.67365	-1.89978	-0.97916
C	-3.64112	0.20866	0.21182
C	-5.0534	-1.79952	-1.14511
H	-3.15053	-2.76283	-1.37103
C	-5.01468	0.31172	0.04903
H	-3.10352	0.9725	0.76636

C	-5.73157	-0.68939	-0.633
H	-5.58979	-2.58335	-1.67572
C	-5.95256	1.42583	0.54164
C	-7.14733	-0.32192	-0.6481
C	-7.3052	0.91378	0.02464
C	-8.24707	-0.9907	-1.19368
C	-8.58033	1.46596	0.14411
C	-9.5179	-0.42387	-1.06745
H	-8.11868	-1.93945	-1.709
C	-9.68385	0.79486	-0.40295
H	-8.73624	2.41251	0.6503
H	-10.3811	-0.93371	-1.48753
H	-10.67544	1.22945	-0.30842
C	2.92639	-0.90144	0.30738
C	3.67365	-1.89978	0.97917
C	3.64112	0.20866	-0.21183
C	5.05339	-1.79952	1.14511
H	3.15053	-2.76283	1.37104
C	5.01468	0.31172	-0.04903
H	3.10352	0.9725	-0.76637
C	5.73157	-0.68939	0.63301
H	5.58978	-2.58334	1.67573
C	5.95256	1.42582	-0.54164
C	7.14733	-0.32192	0.64811
C	7.3052	0.91377	-0.02463
C	8.24707	-0.9907	1.1937
C	8.58033	1.46596	-0.1441
C	9.5179	-0.42387	1.06747
H	8.11868	-1.93944	1.70902
C	9.68385	0.79486	0.40297
H	8.73624	2.4125	-0.6503
H	10.3811	-0.9337	1.48755
H	10.67544	1.22945	0.30844
C	-5.87338	1.43591	2.1032
C	-6.72535	2.4351	2.89484
H	-4.82011	1.59494	2.36962
H	-6.12019	0.42179	2.44373
H	-6.59637	2.24904	3.96778
H	-6.43053	3.47216	2.70957
H	-7.79281	2.33691	2.67437
C	5.87339	1.4359	-2.10321
C	6.72536	2.43509	-2.89484
H	4.82011	1.59493	-2.36963
H	6.1202	0.42178	-2.44373

H	6.59638	2.24903	-3.96778
H	6.43054	3.47215	-2.70958
H	7.79282	2.33691	-2.67437
C	-5.49107	2.77051	-0.11343
C	-6.46977	3.95104	-0.18028
H	-5.18237	2.54283	-1.14135
H	-4.57773	3.08679	0.40874
H	-5.96518	4.81227	-0.63435
H	-7.33834	3.71563	-0.80286
H	-6.83535	4.26595	0.80068
C	5.49106	2.77051	0.11342
C	6.46976	3.95104	0.18027
H	5.18236	2.54284	1.14134
H	4.57773	3.08679	-0.40876
H	5.96518	4.81227	0.63434
H	7.33834	3.71563	0.80286
H	6.83536	4.26595	-0.80069

Energy Levels

PBP

```
Alpha  occ. eigenvalues --  -88.90813 -14.31093 -14.31093 -10.20287 -10.20255
Alpha  occ. eigenvalues --  -10.18661 -10.18661 -10.17940 -10.17940 -10.17832
Alpha  occ. eigenvalues --  -10.17832 -10.17716 -10.17716 -10.17576 -10.17576
Alpha  occ. eigenvalues --  -10.17181 -10.17180 -10.17124 -10.17124 -10.16339
Alpha  occ. eigenvalues --  -10.16282  -7.96452  -5.93023  -5.92468  -5.92181
Alpha  occ. eigenvalues --   -0.95083  -0.84768  -0.84553  -0.83115  -0.81337
Alpha  occ. eigenvalues --   -0.76115  -0.73322  -0.73170  -0.73045  -0.71441
Alpha  occ. eigenvalues --   -0.68650  -0.62302  -0.59769  -0.59219  -0.58928
Alpha  occ. eigenvalues --   -0.57941  -0.55583  -0.54099  -0.50292  -0.48996
Alpha  occ. eigenvalues --   -0.46718  -0.46467  -0.44701  -0.43973  -0.43175
Alpha  occ. eigenvalues --   -0.42869  -0.41647  -0.40880  -0.40573  -0.40347
Alpha  occ. eigenvalues --   -0.40052  -0.36978  -0.35978  -0.35567  -0.34854
Alpha  occ. eigenvalues --   -0.34286  -0.33782  -0.33502  -0.32507  -0.32294
Alpha  occ. eigenvalues --   -0.31634  -0.30053  -0.27502  -0.25640  -0.25411
Alpha  occ. eigenvalues --   -0.24035  -0.24017  -0.23656  -0.23359  -0.18618
Alpha  occ. eigenvalues --   -0.10658
Alpha virt. eigenvalues --  -0.01592   0.00747   0.01044   0.01255   0.03542
Alpha virt. eigenvalues --   0.04421   0.06620   0.09776   0.10970   0.10978
Alpha virt. eigenvalues --   0.13094   0.15231   0.15366   0.16636   0.17001
Alpha virt. eigenvalues --   0.17637   0.17678   0.18924   0.19892   0.21047
Alpha virt. eigenvalues --   0.21264   0.22090   0.23837   0.25644   0.26474
Alpha virt. eigenvalues --   0.28090   0.28615   0.29209   0.31341   0.31474
Alpha virt. eigenvalues --   0.31631   0.31949   0.32770   0.33779   0.35089
Alpha virt. eigenvalues --   0.36515   0.37314   0.37493   0.38537   0.41078
Alpha virt. eigenvalues --   0.42902   0.43950   0.45321   0.49093   0.50056
```

Alpha virt. eigenvalues -- 0.52056 0.52594 0.54130 0.54567 0.55085
Alpha virt. eigenvalues -- 0.55357 0.55847 0.55896 0.56094 0.56846
Alpha virt. eigenvalues -- 0.57829 0.58003 0.58696 0.59367 0.60336
Alpha virt. eigenvalues -- 0.60467 0.60735 0.61118 0.61193 0.61507
Alpha virt. eigenvalues -- 0.61718 0.62443 0.62447 0.62955 0.63137
Alpha virt. eigenvalues -- 0.64497 0.64873 0.65003 0.65299 0.66337
Alpha virt. eigenvalues -- 0.66397 0.67839 0.68090 0.70180 0.71396
Alpha virt. eigenvalues -- 0.72255 0.73254 0.76124 0.77029 0.77469
Alpha virt. eigenvalues -- 0.80067 0.81347 0.83003 0.83296 0.84093
Alpha virt. eigenvalues -- 0.84250 0.85368 0.85434 0.85569 0.86105
Alpha virt. eigenvalues -- 0.86826 0.87508 0.90043 0.90222 0.91556
Alpha virt. eigenvalues -- 0.92061 0.92514 0.93163 0.94199 0.95134
Alpha virt. eigenvalues -- 0.96460 0.97634 0.99135 0.99558 1.01035
Alpha virt. eigenvalues -- 1.01151 1.03871 1.03889 1.04971 1.05795
Alpha virt. eigenvalues -- 1.07632 1.07777 1.08231 1.09429 1.11613
Alpha virt. eigenvalues -- 1.13046 1.14672 1.15298 1.16425 1.17702
Alpha virt. eigenvalues -- 1.18034 1.18896 1.19292 1.20001 1.22770
Alpha virt. eigenvalues -- 1.23284 1.25932 1.27755 1.30539 1.30651
Alpha virt. eigenvalues -- 1.33540 1.34478 1.37976 1.39050 1.41241
Alpha virt. eigenvalues -- 1.41718 1.44201 1.45355 1.45697 1.45955
Alpha virt. eigenvalues -- 1.46730 1.48921 1.49071 1.50436 1.50556
Alpha virt. eigenvalues -- 1.51146 1.51446 1.52460 1.53314 1.53472
Alpha virt. eigenvalues -- 1.59063 1.61133 1.66348 1.70389 1.74693
Alpha virt. eigenvalues -- 1.77110 1.77194 1.78024 1.78393 1.80736
Alpha virt. eigenvalues -- 1.81530 1.82191 1.82565 1.83490 1.84803
Alpha virt. eigenvalues -- 1.88097 1.89463 1.91308 1.91881 1.92243
Alpha virt. eigenvalues -- 1.93040 1.93218 1.96029 1.96642 1.99446
Alpha virt. eigenvalues -- 1.99830 2.01112 2.02780 2.04638 2.06262
Alpha virt. eigenvalues -- 2.07133 2.08427 2.13218 2.13655 2.15088
Alpha virt. eigenvalues -- 2.15310 2.15541 2.16423 2.17939 2.21782
Alpha virt. eigenvalues -- 2.21898 2.23467 2.25862 2.26850 2.28644
Alpha virt. eigenvalues -- 2.28811 2.29265 2.31720 2.32039 2.32500
Alpha virt. eigenvalues -- 2.32511 2.33084 2.36532 2.38532 2.39835
Alpha virt. eigenvalues -- 2.44171 2.45730 2.47525 2.50648 2.57182
Alpha virt. eigenvalues -- 2.57971 2.58598 2.59099 2.59889 2.60507
Alpha virt. eigenvalues -- 2.63723 2.66109 2.66149 2.67608 2.68666
Alpha virt. eigenvalues -- 2.72352 2.74215 2.74485 2.74582 2.75769
Alpha virt. eigenvalues -- 2.77045 2.77996 2.78405 2.80034 2.82101
Alpha virt. eigenvalues -- 2.90269 2.91371 2.93483 3.00726 3.04141
Alpha virt. eigenvalues -- 3.07635 3.17500 3.32302 3.34677 3.42269
Alpha virt. eigenvalues -- 3.49502 3.89934 4.05076 4.05401 4.09328
Alpha virt. eigenvalues -- 4.09948 4.11308 4.12989 4.13038 4.14735
Alpha virt. eigenvalues -- 4.14815 4.21437 4.24901 4.31725 4.34526
Alpha virt. eigenvalues -- 4.34842 4.37157 4.42774 4.48737 4.64971
Alpha virt. eigenvalues -- 4.72974 4.85273
Beta occ. eigenvalues -- -88.90695 -14.30591 -14.30590 -10.20371 -10.20339

Beta occ. eigenvalues --	-10.18689	-10.18688	-10.17959	-10.17959	-10.17849
Beta occ. eigenvalues --	-10.17849	-10.17672	-10.17672	-10.17535	-10.17535
Beta occ. eigenvalues --	-10.17083	-10.17083	-10.16979	-10.16978	-10.16259
Beta occ. eigenvalues --	-10.16204	-7.96338	-5.92965	-5.92415	-5.91781
Beta occ. eigenvalues --	-0.94470	-0.84477	-0.84421	-0.82455	-0.81044
Beta occ. eigenvalues --	-0.75836	-0.73227	-0.73112	-0.73000	-0.71133
Beta occ. eigenvalues --	-0.68379	-0.61881	-0.59697	-0.58926	-0.58735
Beta occ. eigenvalues --	-0.57882	-0.55425	-0.53953	-0.50258	-0.48869
Beta occ. eigenvalues --	-0.46518	-0.46343	-0.44533	-0.43847	-0.43101
Beta occ. eigenvalues --	-0.42762	-0.41548	-0.40606	-0.40513	-0.40324
Beta occ. eigenvalues --	-0.39230	-0.36856	-0.35644	-0.35404	-0.34727
Beta occ. eigenvalues --	-0.34061	-0.33428	-0.33297	-0.32389	-0.31893
Beta occ. eigenvalues --	-0.30817	-0.28660	-0.27148	-0.25129	-0.24089
Beta occ. eigenvalues --	-0.23860	-0.23834	-0.23302	-0.22150	-0.17088
Beta virt. eigenvalues --	-0.04885	-0.00089	0.00914	0.01141	0.01459
Beta virt. eigenvalues --	0.03991	0.05138	0.07699	0.10306	0.11006
Beta virt. eigenvalues --	0.11031	0.13190	0.15388	0.15478	0.16681
Beta virt. eigenvalues --	0.17043	0.17719	0.17858	0.18970	0.20000
Beta virt. eigenvalues --	0.21154	0.21492	0.22465	0.23890	0.25827
Beta virt. eigenvalues --	0.26566	0.28151	0.28930	0.29354	0.31438
Beta virt. eigenvalues --	0.31555	0.31676	0.32000	0.32876	0.33847
Beta virt. eigenvalues --	0.35243	0.36919	0.37671	0.37695	0.38795
Beta virt. eigenvalues --	0.41183	0.43139	0.44174	0.45430	0.49247
Beta virt. eigenvalues --	0.50285	0.52240	0.52703	0.54211	0.54654
Beta virt. eigenvalues --	0.55166	0.55467	0.55948	0.55998	0.56242
Beta virt. eigenvalues --	0.56980	0.57974	0.58141	0.58804	0.59503
Beta virt. eigenvalues --	0.60498	0.60512	0.60798	0.61221	0.61314
Beta virt. eigenvalues --	0.61601	0.61823	0.62471	0.62491	0.63169
Beta virt. eigenvalues --	0.63200	0.64660	0.65030	0.65200	0.65453
Beta virt. eigenvalues --	0.66475	0.66625	0.68109	0.69677	0.70377
Beta virt. eigenvalues --	0.72397	0.72431	0.73368	0.76264	0.77246
Beta virt. eigenvalues --	0.77646	0.80257	0.81732	0.83221	0.83771
Beta virt. eigenvalues --	0.84319	0.84345	0.85450	0.85478	0.85683
Beta virt. eigenvalues --	0.86179	0.87502	0.87619	0.90175	0.90366
Beta virt. eigenvalues --	0.91728	0.92176	0.92704	0.93281	0.94284
Beta virt. eigenvalues --	0.95277	0.96565	0.97711	0.99427	0.99710
Beta virt. eigenvalues --	1.01398	1.01501	1.04089	1.04177	1.05323
Beta virt. eigenvalues --	1.05914	1.07765	1.08009	1.08613	1.09636
Beta virt. eigenvalues --	1.11735	1.13284	1.14898	1.15403	1.16513
Beta virt. eigenvalues --	1.17851	1.18111	1.19055	1.19446	1.20245
Beta virt. eigenvalues --	1.22912	1.23467	1.26032	1.27871	1.30732
Beta virt. eigenvalues --	1.30775	1.33728	1.34603	1.38090	1.39390
Beta virt. eigenvalues --	1.41451	1.41915	1.44346	1.45421	1.45790
Beta virt. eigenvalues --	1.46045	1.47165	1.49115	1.49124	1.50522
Beta virt. eigenvalues --	1.50740	1.51246	1.51621	1.52658	1.53404
Beta virt. eigenvalues --	1.53555	1.59349	1.61415	1.66642	1.70731

Beta virt. eigenvalues -- 1.75056 1.77258 1.77500 1.78200 1.78631
Beta virt. eigenvalues -- 1.81781 1.81850 1.82371 1.82635 1.83627
Beta virt. eigenvalues -- 1.84929 1.88621 1.89790 1.91443 1.92450
Beta virt. eigenvalues -- 1.92889 1.93296 1.93306 1.96328 1.96764
Beta virt. eigenvalues -- 1.99511 1.99876 2.01263 2.03283 2.04874
Beta virt. eigenvalues -- 2.06308 2.07245 2.08583 2.13407 2.13806
Beta virt. eigenvalues -- 2.15178 2.15385 2.15615 2.16434 2.17991
Beta virt. eigenvalues -- 2.22165 2.22206 2.23709 2.26132 2.27241
Beta virt. eigenvalues -- 2.28775 2.29351 2.30003 2.31952 2.32180
Beta virt. eigenvalues -- 2.32588 2.32764 2.33243 2.37023 2.38681
Beta virt. eigenvalues -- 2.40180 2.44484 2.46166 2.48045 2.50859
Beta virt. eigenvalues -- 2.57276 2.58316 2.58981 2.59269 2.59943
Beta virt. eigenvalues -- 2.60647 2.63862 2.66182 2.66536 2.67803
Beta virt. eigenvalues -- 2.68926 2.72612 2.74378 2.74592 2.74834
Beta virt. eigenvalues -- 2.75780 2.77074 2.78023 2.78437 2.80219
Beta virt. eigenvalues -- 2.82186 2.90322 2.91431 2.93580 3.00831
Beta virt. eigenvalues -- 3.04161 3.08032 3.17561 3.32370 3.34727
Beta virt. eigenvalues -- 3.42295 3.49559 3.90537 4.05610 4.06091
Beta virt. eigenvalues -- 4.09422 4.09985 4.11473 4.13018 4.13068
Beta virt. eigenvalues -- 4.14877 4.14933 4.21545 4.25307 4.31818
Beta virt. eigenvalues -- 4.34560 4.34884 4.37236 4.42850 4.48940
Beta virt. eigenvalues -- 4.65007 4.73055 4.85414

P_2BP_2

Alpha occ. eigenvalues -- -88.91263 -14.31563 -14.31562 -10.20668 -10.20636
Alpha occ. eigenvalues -- -10.19694 -10.19672 -10.18873 -10.18851 -10.18850
Alpha occ. eigenvalues -- -10.18840 -10.18791 -10.18782 -10.18781 -10.18771
Alpha occ. eigenvalues -- -10.18644 -10.18635 -10.18612 -10.18606 -10.18598
Alpha occ. eigenvalues -- -10.18593 -10.18098 -10.18082 -10.17972 -10.17960
Alpha occ. eigenvalues -- -10.17776 -10.17761 -10.17449 -10.17434 -10.17258
Alpha occ. eigenvalues -- -10.17252 -10.16550 -10.16492 -7.96899 -5.93471
Alpha occ. eigenvalues -- -5.92912 -5.92626 -0.95626 -0.86063 -0.86036
Alpha occ. eigenvalues -- -0.84711 -0.84284 -0.83345 -0.81722 -0.77883
Alpha occ. eigenvalues -- -0.76590 -0.74282 -0.74268 -0.73734 -0.73335
Alpha occ. eigenvalues -- -0.73169 -0.71806 -0.71464 -0.68402 -0.63360
Alpha occ. eigenvalues -- -0.61287 -0.61037 -0.60819 -0.60488 -0.58912
Alpha occ. eigenvalues -- -0.58187 -0.57559 -0.56692 -0.55719 -0.54431
Alpha occ. eigenvalues -- -0.52135 -0.51777 -0.48667 -0.47976 -0.47224
Alpha occ. eigenvalues -- -0.47082 -0.45814 -0.45494 -0.44884 -0.44253
Alpha occ. eigenvalues -- -0.43244 -0.43152 -0.43140 -0.42368 -0.41718
Alpha occ. eigenvalues -- -0.41682 -0.41571 -0.41105 -0.40962 -0.40315
Alpha occ. eigenvalues -- -0.40251 -0.38298 -0.37303 -0.37105 -0.36232
Alpha occ. eigenvalues -- -0.35697 -0.35516 -0.35229 -0.35020 -0.34450
Alpha occ. eigenvalues -- -0.33996 -0.33776 -0.33517 -0.33117 -0.32522
Alpha occ. eigenvalues -- -0.32141 -0.31583 -0.30576 -0.27826 -0.27160
Alpha occ. eigenvalues -- -0.26898 -0.25710 -0.24943 -0.24934 -0.24291

Alpha occ. eigenvalues --   -0.24208 -0.23639 -0.23538 -0.21890 -0.18292
Alpha occ. eigenvalues --   -0.10899
Alpha virt. eigenvalues --   -0.03325 -0.02284 -0.00138 -0.00119   0.00772
Alpha virt. eigenvalues --   0.00866   0.01364   0.03289   0.03345   0.04518
Alpha virt. eigenvalues --   0.07206   0.09644   0.10287   0.10306   0.11763
Alpha virt. eigenvalues --   0.12080   0.13027   0.13503   0.13753   0.14356
Alpha virt. eigenvalues --   0.15290   0.15862   0.16542   0.16836   0.17050
Alpha virt. eigenvalues --   0.17687   0.17900   0.18852   0.19027   0.19363
Alpha virt. eigenvalues --   0.20170   0.20566   0.20706   0.21653   0.22638
Alpha virt. eigenvalues --   0.23987   0.24645   0.25164   0.25617   0.26225
Alpha virt. eigenvalues --   0.26662   0.27356   0.27757   0.28599   0.30012
Alpha virt. eigenvalues --   0.30462   0.30662   0.30918   0.30958   0.30975
Alpha virt. eigenvalues --   0.32071   0.32633   0.32730   0.34328   0.35609
Alpha virt. eigenvalues --   0.35993   0.36418   0.36474   0.38248   0.38660
Alpha virt. eigenvalues --   0.39141   0.39745   0.40302   0.40334   0.42956
Alpha virt. eigenvalues --   0.44352   0.45577   0.47499   0.48420   0.50420
Alpha virt. eigenvalues --   0.50653   0.51165   0.52168   0.52495   0.53301
Alpha virt. eigenvalues --   0.54142   0.54173   0.54302   0.54644   0.55024
Alpha virt. eigenvalues --   0.55157   0.55352   0.55652   0.55828   0.56236
Alpha virt. eigenvalues --   0.56421   0.56838   0.57276   0.57531   0.58125
Alpha virt. eigenvalues --   0.58461   0.58774   0.58848   0.59182   0.59757
Alpha virt. eigenvalues --   0.59925   0.60252   0.60352   0.60454   0.60648
Alpha virt. eigenvalues --   0.60711   0.60767   0.61241   0.61525   0.61681
Alpha virt. eigenvalues --   0.61786   0.61983   0.62043   0.62365   0.62773
Alpha virt. eigenvalues --   0.63061   0.63977   0.64343   0.64649   0.65141
Alpha virt. eigenvalues --   0.65375   0.65586   0.66282   0.67071   0.67565
Alpha virt. eigenvalues --   0.67656   0.68036   0.68433   0.68808   0.70667
Alpha virt. eigenvalues --   0.71188   0.72186   0.72844   0.73967   0.75294
Alpha virt. eigenvalues --   0.76326   0.77294   0.77901   0.78002   0.78555
Alpha virt. eigenvalues --   0.80308   0.80617   0.81943   0.82469   0.83304
Alpha virt. eigenvalues --   0.83411   0.83818   0.83994   0.84343   0.84467
Alpha virt. eigenvalues --   0.84531   0.84736   0.85754   0.85874   0.86454
Alpha virt. eigenvalues --   0.87383   0.87794   0.88285   0.88459   0.88930
Alpha virt. eigenvalues --   0.90263   0.90826   0.91763   0.91850   0.92118
Alpha virt. eigenvalues --   0.93541   0.93891   0.94265   0.94337   0.94951
Alpha virt. eigenvalues --   0.96100   0.96414   0.96869   0.97859   0.98608
Alpha virt. eigenvalues --   0.98753   1.00158   1.00265   1.01037   1.01235
Alpha virt. eigenvalues --   1.02082   1.02497   1.02879   1.04651   1.05237
Alpha virt. eigenvalues --   1.05626   1.06375   1.07156   1.07820   1.09041
Alpha virt. eigenvalues --   1.09600   1.10164   1.11395   1.11814   1.12168
Alpha virt. eigenvalues --   1.12579   1.14730   1.15273   1.15816   1.16477
Alpha virt. eigenvalues --   1.16752   1.17443   1.17716   1.17982   1.19079
Alpha virt. eigenvalues --   1.19567   1.20374   1.21765   1.22221   1.23028
Alpha virt. eigenvalues --   1.24480   1.24965   1.25185   1.26628   1.28229
Alpha virt. eigenvalues --   1.28875   1.30127   1.31388   1.33074   1.33763
Alpha virt. eigenvalues --   1.36228   1.37885   1.38922   1.39619   1.41026

Alpha virt. eigenvalues -- 1.42699 1.42975 1.43036 1.43840 1.44098
Alpha virt. eigenvalues -- 1.44901 1.45535 1.45789 1.47362 1.47708
Alpha virt. eigenvalues -- 1.48386 1.48713 1.49394 1.49760 1.49857
Alpha virt. eigenvalues -- 1.49953 1.50611 1.50890 1.51541 1.51841
Alpha virt. eigenvalues -- 1.52084 1.52681 1.53094 1.53769 1.54445
Alpha virt. eigenvalues -- 1.57757 1.61257 1.62161 1.67509 1.68317
Alpha virt. eigenvalues -- 1.72688 1.73920 1.75596 1.76457 1.76697
Alpha virt. eigenvalues -- 1.77244 1.77342 1.78810 1.78993 1.79913
Alpha virt. eigenvalues -- 1.80143 1.80534 1.80831 1.81884 1.82450
Alpha virt. eigenvalues -- 1.82971 1.83588 1.84519 1.84603 1.85858
Alpha virt. eigenvalues -- 1.88678 1.89533 1.89631 1.90253 1.92071
Alpha virt. eigenvalues -- 1.92461 1.92552 1.92791 1.92919 1.95093
Alpha virt. eigenvalues -- 1.96239 1.97977 1.98010 1.99903 2.00820
Alpha virt. eigenvalues -- 2.01042 2.02248 2.03089 2.04569 2.05278
Alpha virt. eigenvalues -- 2.05587 2.05652 2.05720 2.07675 2.09882
Alpha virt. eigenvalues -- 2.10539 2.11573 2.11771 2.12481 2.12565
Alpha virt. eigenvalues -- 2.13148 2.15088 2.15281 2.15590 2.15856
Alpha virt. eigenvalues -- 2.16075 2.16762 2.18059 2.21213 2.22275
Alpha virt. eigenvalues -- 2.23073 2.23872 2.24828 2.26454 2.26637
Alpha virt. eigenvalues -- 2.27471 2.28442 2.29108 2.29422 2.30459
Alpha virt. eigenvalues -- 2.31267 2.31361 2.31467 2.31694 2.32348
Alpha virt. eigenvalues -- 2.32946 2.33319 2.35299 2.35596 2.35954
Alpha virt. eigenvalues -- 2.38563 2.38778 2.42935 2.43769 2.44262
Alpha virt. eigenvalues -- 2.45904 2.48521 2.49140 2.52241 2.55480
Alpha virt. eigenvalues -- 2.57878 2.58220 2.58500 2.58659 2.59064
Alpha virt. eigenvalues -- 2.59654 2.61484 2.61642 2.61804 2.63239
Alpha virt. eigenvalues -- 2.63567 2.65711 2.67061 2.67273 2.68049
Alpha virt. eigenvalues -- 2.69113 2.69615 2.70523 2.71364 2.73888
Alpha virt. eigenvalues -- 2.74078 2.75154 2.75529 2.75963 2.76085
Alpha virt. eigenvalues -- 2.76759 2.78523 2.78847 2.80421 2.81071
Alpha virt. eigenvalues -- 2.83496 2.87882 2.87999 2.90560 2.94413
Alpha virt. eigenvalues -- 2.94511 2.98140 2.99416 3.01702 3.02041
Alpha virt. eigenvalues -- 3.08129 3.11993 3.19073 3.23987 3.32190
Alpha virt. eigenvalues -- 3.33949 3.36914 3.41938 3.46948 3.50939
Alpha virt. eigenvalues -- 3.89746 4.05108 4.05305 4.08655 4.08886
Alpha virt. eigenvalues -- 4.09528 4.10516 4.11475 4.11968 4.11969
Alpha virt. eigenvalues -- 4.12932 4.12945 4.13158 4.13365 4.16726
Alpha virt. eigenvalues -- 4.17160 4.21389 4.24982 4.29731 4.32440
Alpha virt. eigenvalues -- 4.33323 4.33370 4.34898 4.35123 4.38226
Alpha virt. eigenvalues -- 4.41343 4.44388 4.51153 4.62581 4.67491
Alpha virt. eigenvalues -- 4.74693 4.78726 4.86157
Beta occ. eigenvalues -- -88.91151 -14.31106 -14.31102 -10.20753 -10.20721
Beta occ. eigenvalues -- -10.19704 -10.19681 -10.18879 -10.18867 -10.18815
Beta occ. eigenvalues -- -10.18795 -10.18792 -10.18786 -10.18786 -10.18776
Beta occ. eigenvalues -- -10.18633 -10.18623 -10.18601 -10.18595 -10.18586
Beta occ. eigenvalues -- -10.18581 -10.18121 -10.18106 -10.17995 -10.17983

```
Beta  occ. eigenvalues --  -10.17730 -10.17714 -10.17242 -10.17230 -10.17213
Beta  occ. eigenvalues --  -10.17207 -10.16467 -10.16410  -7.96790  -5.93415
Beta  occ. eigenvalues --   -5.92861  -5.92244  -0.95062  -0.86003  -0.85987
Beta  occ. eigenvalues --   -0.84328  -0.84169  -0.82893  -0.81449  -0.77705
Beta  occ. eigenvalues --   -0.76523  -0.74264  -0.74251  -0.73566  -0.73269
Beta  occ. eigenvalues --   -0.73110  -0.71508  -0.71414  -0.68196  -0.63099
Beta  occ. eigenvalues --   -0.61255  -0.61012  -0.60784  -0.60022  -0.58853
Beta  occ. eigenvalues --   -0.58163  -0.57424  -0.56650  -0.55584  -0.54301
Beta  occ. eigenvalues --   -0.52121  -0.51738  -0.48596  -0.47867  -0.47115
Beta  occ. eigenvalues --   -0.47014  -0.45703  -0.45428  -0.44772  -0.44167
Beta  occ. eigenvalues --   -0.43217  -0.43099  -0.43077  -0.42284  -0.41707
Beta  occ. eigenvalues --   -0.41676  -0.41490  -0.41000  -0.40592  -0.40291
Beta  occ. eigenvalues --   -0.39670  -0.38236  -0.37167  -0.37017  -0.36159
Beta  occ. eigenvalues --   -0.35531  -0.35244  -0.35126  -0.34880  -0.34308
Beta  occ. eigenvalues --   -0.33758  -0.33618  -0.33377  -0.33056  -0.32186
Beta  occ. eigenvalues --   -0.31765  -0.31145  -0.29486  -0.27481  -0.26694
Beta  occ. eigenvalues --   -0.26228  -0.25241  -0.24910  -0.24898  -0.24157
Beta  occ. eigenvalues --   -0.23987  -0.22704  -0.22449  -0.21711  -0.17019
Beta virt. eigenvalues --   -0.05682  -0.02129  -0.02041  -0.00099  -0.00084
Beta virt. eigenvalues --    0.00881   0.00976   0.02089   0.03672   0.03726
Beta virt. eigenvalues --    0.05058   0.08070   0.10168   0.10297   0.10314
Beta virt. eigenvalues --    0.11793   0.12114   0.13113   0.13535   0.13809
Beta virt. eigenvalues --    0.14462   0.15404   0.15900   0.16580   0.16869
Beta virt. eigenvalues --    0.17067   0.17758   0.18018   0.18916   0.19142
Beta virt. eigenvalues --    0.19407   0.20236   0.20659   0.20745   0.22067
Beta virt. eigenvalues --    0.22716   0.24074   0.24681   0.25299   0.25703
Beta virt. eigenvalues --    0.26309   0.26699   0.27401   0.27853   0.28864
Beta virt. eigenvalues --    0.30052   0.30484   0.30702   0.30945   0.30997
Beta virt. eigenvalues --    0.31149   0.32196   0.32716   0.32783   0.34413
Beta virt. eigenvalues --    0.35654   0.36130   0.36500   0.37118   0.38313
Beta virt. eigenvalues --    0.38724   0.39327   0.39808   0.40411   0.40424
Beta virt. eigenvalues --    0.43160   0.44542   0.45684   0.47604   0.48507
Beta virt. eigenvalues --    0.50510   0.50699   0.51309   0.52247   0.52668
Beta virt. eigenvalues --    0.53410   0.54152   0.54179   0.54351   0.54732
Beta virt. eigenvalues --    0.55105   0.55206   0.55398   0.55743   0.55926
Beta virt. eigenvalues --    0.56345   0.56508   0.56923   0.57372   0.57588
Beta virt. eigenvalues --    0.58215   0.58550   0.58860   0.58928   0.59257
Beta virt. eigenvalues --    0.59800   0.59946   0.60351   0.60441   0.60547
Beta virt. eigenvalues --    0.60684   0.60729   0.60813   0.61335   0.61589
Beta virt. eigenvalues --    0.61724   0.61815   0.62038   0.62058   0.62445
Beta virt. eigenvalues --    0.62984   0.63238   0.64124   0.64391   0.64695
Beta virt. eigenvalues --    0.65219   0.65523   0.65704   0.66383   0.67206
Beta virt. eigenvalues --    0.67683   0.68105   0.68273   0.68954   0.69346
Beta virt. eigenvalues --    0.70822   0.72026   0.72272   0.73024   0.74069
Beta virt. eigenvalues --    0.75420   0.76552   0.77447   0.78009   0.78124
Beta virt. eigenvalues --    0.78685   0.80478   0.80723   0.82030   0.82802
```

Beta virt. eigenvalues --	0.83356	0.83448	0.83993	0.84057	0.84491
Beta virt. eigenvalues --	0.84535	0.84688	0.84864	0.85773	0.85903
Beta virt. eigenvalues --	0.86848	0.87481	0.87926	0.88518	0.88625
Beta virt. eigenvalues --	0.89048	0.90367	0.90985	0.91802	0.91872
Beta virt. eigenvalues --	0.92235	0.93589	0.93989	0.94301	0.94394
Beta virt. eigenvalues --	0.95053	0.96167	0.96572	0.97026	0.97902
Beta virt. eigenvalues --	0.98673	0.98816	1.00233	1.00522	1.01201
Beta virt. eigenvalues --	1.01347	1.02362	1.02714	1.03054	1.04759
Beta virt. eigenvalues --	1.05407	1.05721	1.06618	1.07252	1.08023
Beta virt. eigenvalues --	1.09167	1.09739	1.10322	1.11549	1.11917
Beta virt. eigenvalues --	1.12233	1.12646	1.14876	1.15370	1.15953
Beta virt. eigenvalues --	1.16558	1.16798	1.17489	1.17822	1.18037
Beta virt. eigenvalues --	1.19173	1.19638	1.20454	1.22028	1.22323
Beta virt. eigenvalues --	1.23143	1.24593	1.25063	1.25296	1.26704
Beta virt. eigenvalues --	1.28287	1.28975	1.30251	1.31490	1.33235
Beta virt. eigenvalues --	1.33868	1.36363	1.37995	1.39173	1.39844
Beta virt. eigenvalues --	1.41150	1.42734	1.42997	1.43067	1.43887
Beta virt. eigenvalues --	1.44117	1.45062	1.45698	1.46050	1.47441
Beta virt. eigenvalues --	1.47882	1.48478	1.48816	1.49441	1.49786
Beta virt. eigenvalues --	1.49925	1.49992	1.50687	1.51032	1.51630
Beta virt. eigenvalues --	1.51948	1.52111	1.52810	1.53181	1.53855
Beta virt. eigenvalues --	1.54524	1.57932	1.61379	1.62420	1.67603
Beta virt. eigenvalues --	1.68617	1.72877	1.74225	1.75721	1.76733
Beta virt. eigenvalues --	1.76822	1.77337	1.77466	1.78933	1.79060
Beta virt. eigenvalues --	1.80083	1.80246	1.80670	1.81673	1.82127
Beta virt. eigenvalues --	1.82586	1.83080	1.83700	1.84623	1.84680
Beta virt. eigenvalues --	1.85950	1.89105	1.89562	1.89653	1.90863
Beta virt. eigenvalues --	1.92295	1.92478	1.92705	1.93214	1.93288
Beta virt. eigenvalues --	1.95358	1.96352	1.97990	1.98018	2.00036
Beta virt. eigenvalues --	2.00865	2.01087	2.02587	2.03310	2.04608
Beta virt. eigenvalues --	2.05349	2.05643	2.05667	2.05777	2.07771
Beta virt. eigenvalues --	2.09994	2.10699	2.11674	2.11842	2.12530
Beta virt. eigenvalues --	2.12610	2.13276	2.15150	2.15347	2.15632
Beta virt. eigenvalues --	2.15907	2.16132	2.16796	2.18280	2.21371
Beta virt. eigenvalues --	2.22362	2.23512	2.23991	2.25064	2.26572
Beta virt. eigenvalues --	2.26748	2.27564	2.28692	2.29654	2.29724
Beta virt. eigenvalues --	2.30728	2.31344	2.31399	2.31570	2.31855
Beta virt. eigenvalues --	2.32567	2.33032	2.33456	2.35418	2.35628
Beta virt. eigenvalues --	2.36327	2.38880	2.38928	2.43076	2.43983
Beta virt. eigenvalues --	2.44459	2.46339	2.48936	2.49283	2.52284
Beta virt. eigenvalues --	2.55638	2.58133	2.58437	2.58547	2.58733
Beta virt. eigenvalues --	2.59279	2.59771	2.61533	2.61705	2.61889
Beta virt. eigenvalues --	2.63343	2.63698	2.65822	2.67257	2.67385
Beta virt. eigenvalues --	2.68165	2.69214	2.69775	2.70713	2.71646
Beta virt. eigenvalues --	2.73905	2.74102	2.75210	2.75567	2.75987
Beta virt. eigenvalues --	2.76096	2.76893	2.78550	2.78977	2.80478

Beta virt. eigenvalues -- 2.81145 2.83621 2.87905 2.88023 2.90615
Beta virt. eigenvalues -- 2.94455 2.94557 2.98195 2.99500 3.01717
Beta virt. eigenvalues -- 3.02144 3.08513 3.12041 3.19093 3.24020
Beta virt. eigenvalues -- 3.32234 3.34010 3.36930 3.41968 3.46965
Beta virt. eigenvalues -- 3.50986 3.90292 4.05615 4.05933 4.08712
Beta virt. eigenvalues -- 4.08902 4.09617 4.10557 4.11581 4.11976
Beta virt. eigenvalues -- 4.11977 4.12982 4.12998 4.13192 4.13466
Beta virt. eigenvalues -- 4.16799 4.17256 4.21476 4.25351 4.29780
Beta virt. eigenvalues -- 4.32483 4.33335 4.33381 4.34930 4.35164
Beta virt. eigenvalues -- 4.38334 4.41473 4.44401 4.51309 4.62604
Beta virt. eigenvalues -- 4.67546 4.74739 4.78782 4.86291

PBpyP

Alpha occ. eigenvalues -- -88.92281 -14.32634 -14.32633 -14.31567 -14.31567
Alpha occ. eigenvalues -- -10.21629 -10.21598 -10.20847 -10.20847 -10.20751
Alpha occ. eigenvalues -- -10.20751 -10.20198 -10.20198 -10.18171 -10.18170
Alpha occ. eigenvalues -- -10.18102 -10.18102 -10.17516 -10.17516 -10.17275
Alpha occ. eigenvalues -- -10.17216 -7.97908 -5.94481 -5.93914 -5.93635
Alpha occ. eigenvalues -- -0.96869 -0.92379 -0.92374 -0.85529 -0.83803
Alpha occ. eigenvalues -- -0.80366 -0.77913 -0.74531 -0.74406 -0.72733
Alpha occ. eigenvalues -- -0.71629 -0.64699 -0.61851 -0.61254 -0.60733
Alpha occ. eigenvalues -- -0.59998 -0.57041 -0.55517 -0.50606 -0.49347
Alpha occ. eigenvalues -- -0.47619 -0.47606 -0.45985 -0.45629 -0.44575
Alpha occ. eigenvalues -- -0.43740 -0.43290 -0.41911 -0.41031 -0.40604
Alpha occ. eigenvalues -- -0.39451 -0.38654 -0.38472 -0.36767 -0.36119
Alpha occ. eigenvalues -- -0.35233 -0.34376 -0.33482 -0.33096 -0.32143
Alpha occ. eigenvalues -- -0.29001 -0.27928 -0.26767 -0.26726 -0.25112
Alpha occ. eigenvalues -- -0.25012 -0.24803 -0.24630 -0.24301 -0.20166
Alpha occ. eigenvalues -- -0.11912
Alpha virt. eigenvalues -- -0.03369 -0.01070 0.00382 0.00484 0.02670
Alpha virt. eigenvalues -- 0.03778 0.05784 0.09204 0.11599 0.12296
Alpha virt. eigenvalues -- 0.13313 0.14028 0.15259 0.16258 0.17024
Alpha virt. eigenvalues -- 0.17070 0.19375 0.20197 0.21385 0.21816
Alpha virt. eigenvalues -- 0.22656 0.22819 0.25099 0.26011 0.26496
Alpha virt. eigenvalues -- 0.27263 0.30349 0.30675 0.30938 0.32096
Alpha virt. eigenvalues -- 0.32710 0.33707 0.35219 0.35981 0.37075
Alpha virt. eigenvalues -- 0.38278 0.38394 0.39761 0.42942 0.43297
Alpha virt. eigenvalues -- 0.45048 0.47774 0.48359 0.50568 0.50608
Alpha virt. eigenvalues -- 0.50817 0.51850 0.52462 0.52993 0.54346
Alpha virt. eigenvalues -- 0.55648 0.55765 0.56706 0.57564 0.58194
Alpha virt. eigenvalues -- 0.58457 0.59000 0.59462 0.59769 0.60438
Alpha virt. eigenvalues -- 0.61291 0.61375 0.61581 0.61870 0.62232
Alpha virt. eigenvalues -- 0.63034 0.63232 0.64762 0.65175 0.65732
Alpha virt. eigenvalues -- 0.66433 0.67073 0.67348 0.68964 0.69146
Alpha virt. eigenvalues -- 0.69989 0.70681 0.73139 0.73247 0.76375
Alpha virt. eigenvalues -- 0.77616 0.77903 0.81073 0.81519 0.83441

Alpha virt. eigenvalues -- 0.83715 0.84111 0.84372 0.84626 0.85419
Alpha virt. eigenvalues -- 0.86081 0.86118 0.86892 0.87430 0.88474
Alpha virt. eigenvalues -- 0.89636 0.90389 0.90575 0.90793 0.92360
Alpha virt. eigenvalues -- 0.93379 0.94554 0.97179 0.97838 0.98448
Alpha virt. eigenvalues -- 0.99667 1.01221 1.03193 1.03325 1.04267
Alpha virt. eigenvalues -- 1.05601 1.08220 1.09725 1.09729 1.11923
Alpha virt. eigenvalues -- 1.12995 1.13164 1.15473 1.16437 1.17493
Alpha virt. eigenvalues -- 1.18168 1.18658 1.19725 1.21323 1.22185
Alpha virt. eigenvalues -- 1.23259 1.24850 1.27000 1.27265 1.28291
Alpha virt. eigenvalues -- 1.30596 1.31819 1.35223 1.38690 1.38702
Alpha virt. eigenvalues -- 1.39633 1.42604 1.42974 1.43939 1.44680
Alpha virt. eigenvalues -- 1.44814 1.48692 1.49516 1.50178 1.50854
Alpha virt. eigenvalues -- 1.50956 1.51843 1.52011 1.52758 1.54280
Alpha virt. eigenvalues -- 1.54929 1.57554 1.61019 1.65182 1.70307
Alpha virt. eigenvalues -- 1.71042 1.71775 1.72519 1.75426 1.76751
Alpha virt. eigenvalues -- 1.77482 1.77911 1.79729 1.80516 1.81469
Alpha virt. eigenvalues -- 1.81686 1.82184 1.86829 1.87673 1.89093
Alpha virt. eigenvalues -- 1.91371 1.91567 1.91914 1.95227 1.95340
Alpha virt. eigenvalues -- 1.99165 2.01075 2.02230 2.02715 2.02722
Alpha virt. eigenvalues -- 2.03522 2.06747 2.10150 2.13194 2.13319
Alpha virt. eigenvalues -- 2.14903 2.18520 2.20142 2.20278 2.20867
Alpha virt. eigenvalues -- 2.21752 2.22877 2.24062 2.27039 2.28169
Alpha virt. eigenvalues -- 2.29113 2.29776 2.29965 2.30124 2.31424
Alpha virt. eigenvalues -- 2.32035 2.35058 2.35757 2.38580 2.39340
Alpha virt. eigenvalues -- 2.42286 2.42876 2.46065 2.48493 2.51325
Alpha virt. eigenvalues -- 2.55424 2.55943 2.59063 2.60102 2.61137
Alpha virt. eigenvalues -- 2.61156 2.62609 2.63147 2.66844 2.67580
Alpha virt. eigenvalues -- 2.68056 2.69569 2.71110 2.73195 2.76436
Alpha virt. eigenvalues -- 2.76973 2.77896 2.78337 2.79316 2.80181
Alpha virt. eigenvalues -- 2.81535 2.90381 2.95566 2.96178 3.00462
Alpha virt. eigenvalues -- 3.06103 3.06879 3.18940 3.30311 3.33968
Alpha virt. eigenvalues -- 3.38979 3.48468 3.89117 4.04199 4.04654
Alpha virt. eigenvalues -- 4.04859 4.06135 4.10075 4.11596 4.11791
Alpha virt. eigenvalues -- 4.15479 4.16059 4.20382 4.22121 4.24422
Alpha virt. eigenvalues -- 4.29689 4.33890 4.34063 4.41417 4.42652
Alpha virt. eigenvalues -- 4.57375 4.68551 4.84385
Beta occ. eigenvalues -- -88.92174 -14.32259 -14.32258 -14.31471 -14.31471
Beta occ. eigenvalues -- -10.21718 -10.21686 -10.20872 -10.20872 -10.20774
Beta occ. eigenvalues -- -10.20774 -10.20212 -10.20212 -10.18045 -10.18045
Beta occ. eigenvalues -- -10.17944 -10.17944 -10.17461 -10.17461 -10.17180
Beta occ. eigenvalues -- -10.17123 -7.97805 -5.94427 -5.93864 -5.93276
Beta occ. eigenvalues -- -0.96396 -0.92254 -0.92248 -0.84872 -0.83433
Beta occ. eigenvalues -- -0.80083 -0.77755 -0.74445 -0.74337 -0.72429
Beta occ. eigenvalues -- -0.71237 -0.64292 -0.61782 -0.61194 -0.60292
Beta occ. eigenvalues -- -0.59921 -0.56893 -0.55377 -0.50526 -0.49232
Beta occ. eigenvalues -- -0.47467 -0.47406 -0.45930 -0.45564 -0.44473

Beta occ. eigenvalues --	-0.43569	-0.43145	-0.41223	-0.41003	-0.40401
Beta occ. eigenvalues --	-0.39315	-0.38372	-0.38114	-0.36550	-0.36037
Beta occ. eigenvalues --	-0.34832	-0.33965	-0.32934	-0.32510	-0.31168
Beta occ. eigenvalues --	-0.28724	-0.26682	-0.26259	-0.26233	-0.24844
Beta occ. eigenvalues --	-0.24811	-0.24651	-0.24428	-0.23171	-0.18520
Beta virt. eigenvalues --	-0.06767	-0.01784	-0.00553	0.00543	0.00614
Beta virt. eigenvalues --	0.03044	0.04342	0.06963	0.09696	0.11660
Beta virt. eigenvalues --	0.12342	0.13383	0.14478	0.15274	0.16274
Beta virt. eigenvalues --	0.17111	0.17395	0.19467	0.20520	0.21680
Beta virt. eigenvalues --	0.21907	0.22848	0.22904	0.25182	0.26129
Beta virt. eigenvalues --	0.26582	0.27580	0.30581	0.30775	0.31053
Beta virt. eigenvalues --	0.32121	0.32768	0.33958	0.35392	0.36583
Beta virt. eigenvalues --	0.37200	0.38424	0.38507	0.39871	0.43155
Beta virt. eigenvalues --	0.43501	0.45147	0.47880	0.48465	0.50603
Beta virt. eigenvalues --	0.50863	0.50938	0.52008	0.52653	0.53089
Beta virt. eigenvalues --	0.54483	0.55774	0.55916	0.56858	0.57770
Beta virt. eigenvalues --	0.58390	0.58684	0.59097	0.59585	0.59873
Beta virt. eigenvalues --	0.60517	0.61441	0.61585	0.61643	0.61964
Beta virt. eigenvalues --	0.62381	0.63150	0.63421	0.64870	0.65286
Beta virt. eigenvalues --	0.65877	0.66616	0.67573	0.68242	0.69241
Beta virt. eigenvalues --	0.69252	0.70795	0.70917	0.73439	0.73463
Beta virt. eigenvalues --	0.76493	0.77910	0.78052	0.81591	0.81614
Beta virt. eigenvalues --	0.83736	0.84040	0.84137	0.84495	0.84823
Beta virt. eigenvalues --	0.85764	0.86192	0.86355	0.87074	0.87702
Beta virt. eigenvalues --	0.88682	0.90098	0.90547	0.90775	0.90883
Beta virt. eigenvalues --	0.92484	0.93556	0.94662	0.97290	0.97926
Beta virt. eigenvalues --	0.98711	0.99875	1.01692	1.03353	1.03460
Beta virt. eigenvalues --	1.04501	1.05861	1.08408	1.09899	1.10003
Beta virt. eigenvalues --	1.12043	1.13053	1.13333	1.15610	1.16611
Beta virt. eigenvalues --	1.17558	1.18309	1.18866	1.19973	1.21565
Beta virt. eigenvalues --	1.22332	1.23450	1.25094	1.27222	1.27453
Beta virt. eigenvalues --	1.28420	1.30768	1.31937	1.35451	1.38861
Beta virt. eigenvalues --	1.38967	1.39765	1.42811	1.43367	1.44104
Beta virt. eigenvalues --	1.44890	1.44904	1.48782	1.49586	1.50573
Beta virt. eigenvalues --	1.51015	1.51110	1.51988	1.52230	1.52903
Beta virt. eigenvalues --	1.54331	1.54996	1.57778	1.61284	1.65446
Beta virt. eigenvalues --	1.70534	1.71217	1.71996	1.72788	1.75750
Beta virt. eigenvalues --	1.76830	1.77804	1.78146	1.80598	1.80604
Beta virt. eigenvalues --	1.81639	1.81970	1.82456	1.86850	1.87800
Beta virt. eigenvalues --	1.89994	1.91673	1.92039	1.92235	1.95374
Beta virt. eigenvalues --	1.95570	1.99259	2.01159	2.02367	2.02764
Beta virt. eigenvalues --	2.02959	2.03822	2.06984	2.10386	2.13311
Beta virt. eigenvalues --	2.13461	2.15236	2.18786	2.20278	2.20365
Beta virt. eigenvalues --	2.20898	2.22150	2.23113	2.24318	2.27335
Beta virt. eigenvalues --	2.28442	2.29518	2.29973	2.30270	2.30566
Beta virt. eigenvalues --	2.31704	2.32300	2.35438	2.35990	2.38734

Beta virt. eigenvalues -- 2.39593 2.42519 2.43185 2.46474 2.48798
Beta virt. eigenvalues -- 2.51627 2.55674 2.56198 2.59291 2.60490
Beta virt. eigenvalues -- 2.61259 2.61284 2.62962 2.63346 2.67061
Beta virt. eigenvalues -- 2.67754 2.68179 2.69708 2.71329 2.73490
Beta virt. eigenvalues -- 2.76528 2.77186 2.77967 2.78487 2.79357
Beta virt. eigenvalues -- 2.80207 2.81687 2.90440 2.95610 2.96291
Beta virt. eigenvalues -- 3.00543 3.06250 3.07207 3.19004 3.30371
Beta virt. eigenvalues -- 3.34042 3.39011 3.48539 3.89562 4.04545
Beta virt. eigenvalues -- 4.04812 4.05314 4.06490 4.10365 4.11650
Beta virt. eigenvalues -- 4.11839 4.15630 4.16187 4.20486 4.22388
Beta virt. eigenvalues -- 4.24401 4.29852 4.33941 4.34119 4.41536
Beta virt. eigenvalues -- 4.42900 4.57428 4.68705 4.84559

PyBPy

Alpha occ. eigenvalues -- -88.92281 -14.32634 -14.32633 -14.31567 -14.31567
Alpha occ. eigenvalues -- -10.21629 -10.21598 -10.20847 -10.20847 -10.20751
Alpha occ. eigenvalues -- -10.20751 -10.20198 -10.20198 -10.18171 -10.18170
Alpha occ. eigenvalues -- -10.18102 -10.18102 -10.17516 -10.17516 -10.17275
Alpha occ. eigenvalues -- -10.17216 -7.97908 -5.94481 -5.93914 -5.93635
Alpha occ. eigenvalues -- -0.96869 -0.92379 -0.92374 -0.85529 -0.83803
Alpha occ. eigenvalues -- -0.80366 -0.77913 -0.74531 -0.74406 -0.72733
Alpha occ. eigenvalues -- -0.71629 -0.64699 -0.61851 -0.61254 -0.60733
Alpha occ. eigenvalues -- -0.59998 -0.57041 -0.55517 -0.50606 -0.49347
Alpha occ. eigenvalues -- -0.47619 -0.47606 -0.45985 -0.45629 -0.44575
Alpha occ. eigenvalues -- -0.43740 -0.43290 -0.41911 -0.41031 -0.40604
Alpha occ. eigenvalues -- -0.39451 -0.38654 -0.38472 -0.36767 -0.36119
Alpha occ. eigenvalues -- -0.35233 -0.34376 -0.33482 -0.33096 -0.32143
Alpha occ. eigenvalues -- -0.29001 -0.27928 -0.26767 -0.26726 -0.25112
Alpha occ. eigenvalues -- -0.25012 -0.24803 -0.24630 -0.24301 -0.20166
Alpha occ. eigenvalues -- -0.11912
Alpha virt. eigenvalues -- -0.03369 -0.01070 0.00382 0.00484 0.02670
Alpha virt. eigenvalues -- 0.03778 0.05784 0.09204 0.11599 0.12296
Alpha virt. eigenvalues -- 0.13313 0.14028 0.15259 0.16258 0.17024
Alpha virt. eigenvalues -- 0.17070 0.19375 0.20197 0.21385 0.21816
Alpha virt. eigenvalues -- 0.22656 0.22819 0.25099 0.26011 0.26496
Alpha virt. eigenvalues -- 0.27263 0.30349 0.30675 0.30938 0.32096
Alpha virt. eigenvalues -- 0.32710 0.33707 0.35219 0.35981 0.37075
Alpha virt. eigenvalues -- 0.38278 0.38394 0.39761 0.42942 0.43297
Alpha virt. eigenvalues -- 0.45048 0.47774 0.48359 0.50568 0.50608
Alpha virt. eigenvalues -- 0.50817 0.51850 0.52462 0.52993 0.54346
Alpha virt. eigenvalues -- 0.55648 0.55765 0.56706 0.57564 0.58194
Alpha virt. eigenvalues -- 0.58457 0.59000 0.59462 0.59769 0.60438
Alpha virt. eigenvalues -- 0.61291 0.61375 0.61581 0.61870 0.62232

Alpha virt. eigenvalues -- 0.63034 0.63232 0.64762 0.65175 0.65732
Alpha virt. eigenvalues -- 0.66433 0.67073 0.67348 0.68964 0.69146
Alpha virt. eigenvalues -- 0.69989 0.70681 0.73139 0.73247 0.76375
Alpha virt. eigenvalues -- 0.77616 0.77903 0.81073 0.81519 0.83441
Alpha virt. eigenvalues -- 0.83715 0.84111 0.84372 0.84626 0.85419
Alpha virt. eigenvalues -- 0.86081 0.86118 0.86892 0.87430 0.88474
Alpha virt. eigenvalues -- 0.89636 0.90389 0.90575 0.90793 0.92360
Alpha virt. eigenvalues -- 0.93379 0.94554 0.97179 0.97838 0.98448
Alpha virt. eigenvalues -- 0.99667 1.01221 1.03193 1.03325 1.04267
Alpha virt. eigenvalues -- 1.05601 1.08220 1.09725 1.09729 1.11923
Alpha virt. eigenvalues -- 1.12995 1.13164 1.15473 1.16437 1.17493
Alpha virt. eigenvalues -- 1.18168 1.18658 1.19725 1.21323 1.22185
Alpha virt. eigenvalues -- 1.23259 1.24850 1.27000 1.27265 1.28291
Alpha virt. eigenvalues -- 1.30596 1.31819 1.35223 1.38690 1.38702
Alpha virt. eigenvalues -- 1.39633 1.42604 1.42974 1.43939 1.44680
Alpha virt. eigenvalues -- 1.44814 1.48692 1.49516 1.50178 1.50854
Alpha virt. eigenvalues -- 1.50956 1.51843 1.52011 1.52758 1.54280
Alpha virt. eigenvalues -- 1.54929 1.57554 1.61019 1.65182 1.70307
Alpha virt. eigenvalues -- 1.71042 1.71775 1.72519 1.75426 1.76751
Alpha virt. eigenvalues -- 1.77482 1.77911 1.79729 1.80516 1.81469
Alpha virt. eigenvalues -- 1.81686 1.82184 1.86829 1.87673 1.89093
Alpha virt. eigenvalues -- 1.91371 1.91567 1.91914 1.95227 1.95340
Alpha virt. eigenvalues -- 1.99165 2.01075 2.02230 2.02715 2.02722
Alpha virt. eigenvalues -- 2.03522 2.06747 2.10150 2.13194 2.13319
Alpha virt. eigenvalues -- 2.14903 2.18520 2.20142 2.20278 2.20867
Alpha virt. eigenvalues -- 2.21752 2.22877 2.24062 2.27039 2.28169
Alpha virt. eigenvalues -- 2.29113 2.29776 2.29965 2.30124 2.31424
Alpha virt. eigenvalues -- 2.32035 2.35058 2.35757 2.38580 2.39340
Alpha virt. eigenvalues -- 2.42286 2.42876 2.46065 2.48493 2.51325
Alpha virt. eigenvalues -- 2.55424 2.55943 2.59063 2.60102 2.61137
Alpha virt. eigenvalues -- 2.61156 2.62609 2.63147 2.66844 2.67580
Alpha virt. eigenvalues -- 2.68056 2.69569 2.71110 2.73195 2.76436
Alpha virt. eigenvalues -- 2.76973 2.77896 2.78337 2.79316 2.80181
Alpha virt. eigenvalues -- 2.81535 2.90381 2.95566 2.96178 3.00462
Alpha virt. eigenvalues -- 3.06103 3.06879 3.18940 3.30311 3.33968
Alpha virt. eigenvalues -- 3.38979 3.48468 3.89117 4.04199 4.04654
Alpha virt. eigenvalues -- 4.04859 4.06135 4.10075 4.11596 4.11791
Alpha virt. eigenvalues -- 4.15479 4.16059 4.20382 4.22121 4.24422
Alpha virt. eigenvalues -- 4.29689 4.33890 4.34063 4.41417 4.42652
Alpha virt. eigenvalues -- 4.57375 4.68551 4.84385
Beta occ. eigenvalues -- -88.92174 -14.32259 -14.32258 -14.31471 -14.31471
Beta occ. eigenvalues -- -10.21718 -10.21686 -10.20872 -10.20872 -10.20774
Beta occ. eigenvalues -- -10.20774 -10.20212 -10.20212 -10.18045 -10.18045
Beta occ. eigenvalues -- -10.17944 -10.17944 -10.17461 -10.17461 -10.17180
Beta occ. eigenvalues -- -10.17123 -7.97805 -5.94427 -5.93864 -5.93276
Beta occ. eigenvalues -- -0.96396 -0.92254 -0.92248 -0.84872 -0.83433

Beta occ. eigenvalues -- -0.80083 -0.77755 -0.74445 -0.74337 -0.72429
Beta occ. eigenvalues -- -0.71237 -0.64292 -0.61782 -0.61194 -0.60292
Beta occ. eigenvalues -- -0.59921 -0.56893 -0.55377 -0.50526 -0.49232
Beta occ. eigenvalues -- -0.47467 -0.47406 -0.45930 -0.45564 -0.44473
Beta occ. eigenvalues -- -0.43569 -0.43145 -0.41223 -0.41003 -0.40401
Beta occ. eigenvalues -- -0.39315 -0.38372 -0.38114 -0.36550 -0.36037
Beta occ. eigenvalues -- -0.34832 -0.33965 -0.32934 -0.32510 -0.31168
Beta occ. eigenvalues -- -0.28724 -0.26682 -0.26259 -0.26233 -0.24844
Beta occ. eigenvalues -- -0.24811 -0.24651 -0.24428 -0.23171 -0.18520
Beta virt. eigenvalues -- -0.06767 -0.01784 -0.00553   0.00543   0.00614
Beta virt. eigenvalues --   0.03044   0.04342   0.06963   0.09696   0.11660
Beta virt. eigenvalues --   0.12342   0.13383   0.14478   0.15274   0.16274
Beta virt. eigenvalues --   0.17111   0.17395   0.19467   0.20520   0.21680
Beta virt. eigenvalues --   0.21907   0.22848   0.22904   0.25182   0.26129
Beta virt. eigenvalues --   0.26582   0.27580   0.30581   0.30775   0.31053
Beta virt. eigenvalues --   0.32121   0.32768   0.33958   0.35392   0.36583
Beta virt. eigenvalues --   0.37200   0.38424   0.38507   0.39871   0.43155
Beta virt. eigenvalues --   0.43501   0.45147   0.47880   0.48465   0.50603
Beta virt. eigenvalues --   0.50863   0.50938   0.52008   0.52653   0.53089
Beta virt. eigenvalues --   0.54483   0.55774   0.55916   0.56858   0.57770
Beta virt. eigenvalues --   0.58390   0.58684   0.59097   0.59585   0.59873
Beta virt. eigenvalues --   0.60517   0.61441   0.61585   0.61643   0.61964
Beta virt. eigenvalues --   0.62381   0.63150   0.63421   0.64870   0.65286
Beta virt. eigenvalues --   0.65877   0.66616   0.67573   0.68242   0.69241
Beta virt. eigenvalues --   0.69252   0.70795   0.70917   0.73439   0.73463
Beta virt. eigenvalues --   0.76493   0.77910   0.78052   0.81591   0.81614
Beta virt. eigenvalues --   0.83736   0.84040   0.84137   0.84495   0.84823
Beta virt. eigenvalues --   0.85764   0.86192   0.86355   0.87074   0.87702
Beta virt. eigenvalues --   0.88682   0.90098   0.90547   0.90775   0.90883
Beta virt. eigenvalues --   0.92484   0.93556   0.94662   0.97290   0.97926
Beta virt. eigenvalues --   0.98711   0.99875   1.01692   1.03353   1.03460
Beta virt. eigenvalues --   1.04501   1.05861   1.08408   1.09899   1.10003
Beta virt. eigenvalues --   1.12043   1.13053   1.13333   1.15610   1.16611
Beta virt. eigenvalues --   1.17558   1.18309   1.18866   1.19973   1.21565
Beta virt. eigenvalues --   1.22332   1.23450   1.25094   1.27222   1.27453
Beta virt. eigenvalues --   1.28420   1.30768   1.31937   1.35451   1.38861
Beta virt. eigenvalues --   1.38967   1.39765   1.42811   1.43367   1.44104
Beta virt. eigenvalues --   1.44890   1.44904   1.48782   1.49586   1.50573
Beta virt. eigenvalues --   1.51015   1.51110   1.51988   1.52230   1.52903
Beta virt. eigenvalues --   1.54331   1.54996   1.57778   1.61284   1.65446
Beta virt. eigenvalues --   1.70534   1.71217   1.71996   1.72788   1.75750
Beta virt. eigenvalues --   1.76830   1.77804   1.78146   1.80598   1.80604
Beta virt. eigenvalues --   1.81639   1.81970   1.82456   1.86850   1.87800
Beta virt. eigenvalues --   1.89994   1.91673   1.92039   1.92235   1.95374
Beta virt. eigenvalues --   1.95570   1.99259   2.01159   2.02367   2.02764
Beta virt. eigenvalues --   2.02959   2.03822   2.06984   2.10386   2.13311

Beta virt. eigenvalues --	2.13461	2.15236	2.18786	2.20278	2.20365
Beta virt. eigenvalues --	2.20898	2.22150	2.23113	2.24318	2.27335
Beta virt. eigenvalues --	2.28442	2.29518	2.29973	2.30270	2.30566
Beta virt. eigenvalues --	2.31704	2.32300	2.35438	2.35990	2.38734
Beta virt. eigenvalues --	2.39593	2.42519	2.43185	2.46474	2.48798
Beta virt. eigenvalues --	2.51627	2.55674	2.56198	2.59291	2.60490
Beta virt. eigenvalues --	2.61259	2.61284	2.62962	2.63346	2.67061
Beta virt. eigenvalues --	2.67754	2.68179	2.69708	2.71329	2.73490
Beta virt. eigenvalues --	2.76528	2.77186	2.77967	2.78487	2.79357
Beta virt. eigenvalues --	2.80207	2.81687	2.90440	2.95610	2.96291
Beta virt. eigenvalues --	3.00543	3.06250	3.07207	3.19004	3.30371
Beta virt. eigenvalues --	3.34042	3.39011	3.48539	3.89562	4.04545
Beta virt. eigenvalues --	4.04812	4.05314	4.06490	4.10365	4.11650
Beta virt. eigenvalues --	4.11839	4.15630	4.16187	4.20486	4.22388
Beta virt. eigenvalues --	4.24401	4.29852	4.33941	4.34119	4.41536
Beta virt. eigenvalues --	4.42900	4.57428	4.68705	4.84559	

FBF

Alpha occ. eigenvalues --	-88.91092	-14.31384	-14.31384	-10.20506	-10.20474
Alpha occ. eigenvalues --	-10.20054	-10.20054	-10.18787	-10.18787	-10.18767
Alpha occ. eigenvalues --	-10.18767	-10.18628	-10.18628	-10.18562	-10.18562
Alpha occ. eigenvalues --	-10.18487	-10.18487	-10.18450	-10.18450	-10.18404
Alpha occ. eigenvalues --	-10.18404	-10.18117	-10.18116	-10.18110	-10.18110
Alpha occ. eigenvalues --	-10.18005	-10.18005	-10.17963	-10.17963	-10.17924
Alpha occ. eigenvalues --	-10.17924	-10.17558	-10.17558	-10.17311	-10.17311
Alpha occ. eigenvalues --	-10.17182	-10.17182	-10.16967	-10.16967	-10.16862
Alpha occ. eigenvalues --	-10.16862	-10.16431	-10.16374	-7.96729	-5.93301
Alpha occ. eigenvalues --	-5.92744	-5.92457	-0.95415	-0.88019	-0.88016
Alpha occ. eigenvalues --	-0.84564	-0.84067	-0.83193	-0.82046	-0.80801
Alpha occ. eigenvalues --	-0.80287	-0.77762	-0.76755	-0.74236	-0.74232
Alpha occ. eigenvalues --	-0.74154	-0.73846	-0.73645	-0.73384	-0.72794
Alpha occ. eigenvalues --	-0.71750	-0.70803	-0.67956	-0.67607	-0.67562
Alpha occ. eigenvalues --	-0.63537	-0.62293	-0.61214	-0.60939	-0.60935
Alpha occ. eigenvalues --	-0.59986	-0.59805	-0.58314	-0.57745	-0.57115
Alpha occ. eigenvalues --	-0.56258	-0.55622	-0.54503	-0.53891	-0.53065
Alpha occ. eigenvalues --	-0.49945	-0.49895	-0.48197	-0.47844	-0.47133
Alpha occ. eigenvalues --	-0.47052	-0.46101	-0.45496	-0.44948	-0.44332
Alpha occ. eigenvalues --	-0.44227	-0.44201	-0.44028	-0.43828	-0.43449
Alpha occ. eigenvalues --	-0.43076	-0.42810	-0.42072	-0.41874	-0.41226
Alpha occ. eigenvalues --	-0.40746	-0.40454	-0.40293	-0.39986	-0.39969
Alpha occ. eigenvalues --	-0.39416	-0.39135	-0.38795	-0.38726	-0.37589
Alpha occ. eigenvalues --	-0.37395	-0.37094	-0.36362	-0.36171	-0.36140
Alpha occ. eigenvalues --	-0.36102	-0.35971	-0.35954	-0.35233	-0.34623
Alpha occ. eigenvalues --	-0.34436	-0.34149	-0.34132	-0.34069	-0.33449
Alpha occ. eigenvalues --	-0.33400	-0.33285	-0.32731	-0.32194	-0.30962
Alpha occ. eigenvalues --	-0.30891	-0.30683	-0.30373	-0.30318	-0.29888

Alpha occ. eigenvalues --	-0.29759	-0.27709	-0.27256	-0.27180	-0.25582
Alpha occ. eigenvalues --	-0.24384	-0.24346	-0.23702	-0.23699	-0.23438
Alpha occ. eigenvalues --	-0.22819	-0.20926	-0.17874	-0.10730	
Alpha virt. eigenvalues --	-0.03360	-0.02401	-0.00306	-0.00302	0.01470
Alpha virt. eigenvalues --	0.01494	0.01984	0.03404	0.03815	0.04978
Alpha virt. eigenvalues --	0.07575	0.08970	0.08995	0.09712	0.10286
Alpha virt. eigenvalues --	0.10412	0.11391	0.11402	0.12734	0.13119
Alpha virt. eigenvalues --	0.13352	0.13383	0.13460	0.13899	0.14540
Alpha virt. eigenvalues --	0.14833	0.14968	0.15252	0.15274	0.15715
Alpha virt. eigenvalues --	0.15744	0.17298	0.17464	0.17498	0.17553
Alpha virt. eigenvalues --	0.17917	0.18254	0.18391	0.18648	0.18696
Alpha virt. eigenvalues --	0.19313	0.19648	0.19693	0.20186	0.20538
Alpha virt. eigenvalues --	0.21080	0.21183	0.21305	0.21413	0.22025
Alpha virt. eigenvalues --	0.22256	0.22843	0.23332	0.23725	0.23739
Alpha virt. eigenvalues --	0.25367	0.25379	0.25698	0.25990	0.26602
Alpha virt. eigenvalues --	0.26738	0.26994	0.27079	0.27245	0.28108
Alpha virt. eigenvalues --	0.28381	0.28605	0.28715	0.28993	0.29525
Alpha virt. eigenvalues --	0.30120	0.30612	0.30911	0.30994	0.31291
Alpha virt. eigenvalues --	0.31693	0.32603	0.33234	0.33553	0.35026
Alpha virt. eigenvalues --	0.35946	0.36096	0.36663	0.37933	0.38322
Alpha virt. eigenvalues --	0.38566	0.40257	0.40548	0.40731	0.41620
Alpha virt. eigenvalues --	0.42889	0.44062	0.45761	0.46542	0.46968
Alpha virt. eigenvalues --	0.47824	0.48706	0.49234	0.49434	0.50342
Alpha virt. eigenvalues --	0.50384	0.51359	0.51366	0.51934	0.51948
Alpha virt. eigenvalues --	0.52326	0.52733	0.53288	0.54304	0.54461
Alpha virt. eigenvalues --	0.55111	0.55211	0.55375	0.55484	0.55730
Alpha virt. eigenvalues --	0.55757	0.55965	0.56158	0.56443	0.56653
Alpha virt. eigenvalues --	0.56820	0.57690	0.57845	0.58061	0.58222
Alpha virt. eigenvalues --	0.58821	0.59037	0.59316	0.59417	0.59654
Alpha virt. eigenvalues --	0.59740	0.60635	0.60826	0.60923	0.61069
Alpha virt. eigenvalues --	0.61307	0.61462	0.61597	0.61677	0.62025
Alpha virt. eigenvalues --	0.62052	0.62267	0.62332	0.62465	0.62638
Alpha virt. eigenvalues --	0.62728	0.62821	0.63354	0.63519	0.63665
Alpha virt. eigenvalues --	0.65128	0.65453	0.65949	0.66077	0.66156
Alpha virt. eigenvalues --	0.66957	0.66996	0.67479	0.67512	0.68049
Alpha virt. eigenvalues --	0.68468	0.68576	0.70302	0.70321	0.70651
Alpha virt. eigenvalues --	0.71211	0.71838	0.72559	0.73194	0.73246
Alpha virt. eigenvalues --	0.74320	0.74783	0.75725	0.77628	0.77771
Alpha virt. eigenvalues --	0.78232	0.79547	0.79934	0.80182	0.80252
Alpha virt. eigenvalues --	0.80352	0.80790	0.81129	0.81516	0.81647
Alpha virt. eigenvalues --	0.82090	0.82288	0.82436	0.82474	0.82852
Alpha virt. eigenvalues --	0.84050	0.84584	0.84672	0.85530	0.85684
Alpha virt. eigenvalues --	0.86055	0.86247	0.86688	0.86944	0.87228
Alpha virt. eigenvalues --	0.87724	0.88117	0.88261	0.89183	0.89194
Alpha virt. eigenvalues --	0.89611	0.89727	0.90095	0.90580	0.90590
Alpha virt. eigenvalues --	0.91077	0.91492	0.91572	0.91819	0.92120

Alpha virt. eigenvalues --	0.92971	0.93644	0.94178	0.94309	0.94624
Alpha virt. eigenvalues --	0.94802	0.95437	0.95607	0.96386	0.96634
Alpha virt. eigenvalues --	0.97199	0.97217	0.98251	0.98565	0.98808
Alpha virt. eigenvalues --	0.99011	0.99359	0.99383	1.00075	1.00087
Alpha virt. eigenvalues --	1.00638	1.01362	1.01882	1.02429	1.02442
Alpha virt. eigenvalues --	1.03212	1.03331	1.03778	1.04613	1.04804
Alpha virt. eigenvalues --	1.05403	1.05484	1.05987	1.06517	1.06538
Alpha virt. eigenvalues --	1.06904	1.07032	1.07537	1.08871	1.09212
Alpha virt. eigenvalues --	1.10216	1.10645	1.10780	1.11046	1.11489
Alpha virt. eigenvalues --	1.12423	1.13546	1.14123	1.14415	1.15363
Alpha virt. eigenvalues --	1.16326	1.16617	1.17512	1.17892	1.18760
Alpha virt. eigenvalues --	1.18812	1.19240	1.20520	1.20750	1.22927
Alpha virt. eigenvalues --	1.23796	1.24127	1.24156	1.24871	1.27447
Alpha virt. eigenvalues --	1.27541	1.28136	1.28238	1.29597	1.30486
Alpha virt. eigenvalues --	1.31353	1.33374	1.33636	1.34218	1.35610
Alpha virt. eigenvalues --	1.36366	1.37403	1.37706	1.38628	1.39007
Alpha virt. eigenvalues --	1.40201	1.40375	1.42127	1.42703	1.42992
Alpha virt. eigenvalues --	1.43853	1.45301	1.45573	1.45593	1.45982
Alpha virt. eigenvalues --	1.46502	1.47675	1.47820	1.48051	1.48561
Alpha virt. eigenvalues --	1.49461	1.49521	1.49806	1.49996	1.50946
Alpha virt. eigenvalues --	1.50964	1.52174	1.52762	1.53162	1.55165
Alpha virt. eigenvalues --	1.55984	1.56760	1.58421	1.59138	1.60549
Alpha virt. eigenvalues --	1.62029	1.62783	1.62795	1.65353	1.66471
Alpha virt. eigenvalues --	1.66525	1.67681	1.70485	1.70541	1.72466
Alpha virt. eigenvalues --	1.72686	1.73094	1.73654	1.73778	1.75112
Alpha virt. eigenvalues --	1.75484	1.75878	1.76730	1.76966	1.77344
Alpha virt. eigenvalues --	1.78602	1.78928	1.79522	1.80252	1.80704
Alpha virt. eigenvalues --	1.81011	1.81340	1.81617	1.82125	1.82211
Alpha virt. eigenvalues --	1.82329	1.82469	1.83956	1.84861	1.85185
Alpha virt. eigenvalues --	1.85337	1.87302	1.89088	1.89177	1.89404
Alpha virt. eigenvalues --	1.90311	1.91055	1.91638	1.92267	1.92322
Alpha virt. eigenvalues --	1.92579	1.93599	1.93670	1.94935	1.95109
Alpha virt. eigenvalues --	1.95298	1.95332	1.95630	1.95976	1.96964
Alpha virt. eigenvalues --	1.97130	1.99130	1.99205	1.99404	1.99419
Alpha virt. eigenvalues --	2.00547	2.01807	2.02418	2.02923	2.03016
Alpha virt. eigenvalues --	2.03347	2.04594	2.04629	2.06046	2.06324
Alpha virt. eigenvalues --	2.07005	2.07699	2.08902	2.09141	2.09721
Alpha virt. eigenvalues --	2.10138	2.11270	2.12231	2.13495	2.13643
Alpha virt. eigenvalues --	2.13727	2.13919	2.14968	2.15025	2.16864
Alpha virt. eigenvalues --	2.18165	2.19199	2.19879	2.19883	2.21528
Alpha virt. eigenvalues --	2.21765	2.23622	2.24667	2.24697	2.24905
Alpha virt. eigenvalues --	2.25181	2.25929	2.26383	2.26435	2.26724
Alpha virt. eigenvalues --	2.26960	2.27829	2.28304	2.28934	2.29839
Alpha virt. eigenvalues --	2.30229	2.30758	2.30781	2.31402	2.31885
Alpha virt. eigenvalues --	2.31888	2.32054	2.32241	2.32652	2.33790
Alpha virt. eigenvalues --	2.33864	2.34667	2.35146	2.35732	2.37396

Alpha virt. eigenvalues -- 2.37583 2.38594 2.38988 2.39628 2.40811
Alpha virt. eigenvalues -- 2.41529 2.42248 2.42986 2.43138 2.44384
Alpha virt. eigenvalues -- 2.45803 2.46788 2.47237 2.48590 2.49693
Alpha virt. eigenvalues -- 2.50454 2.51996 2.52245 2.54287 2.54758
Alpha virt. eigenvalues -- 2.55106 2.57425 2.58491 2.59476 2.59630
Alpha virt. eigenvalues -- 2.61058 2.61944 2.63240 2.64476 2.65195
Alpha virt. eigenvalues -- 2.65620 2.66847 2.67695 2.67948 2.68608
Alpha virt. eigenvalues -- 2.68870 2.70652 2.71346 2.71747 2.71756
Alpha virt. eigenvalues -- 2.73796 2.73827 2.75054 2.75170 2.75573
Alpha virt. eigenvalues -- 2.76028 2.76751 2.77403 2.77955 2.78434
Alpha virt. eigenvalues -- 2.79391 2.80667 2.82481 2.83011 2.85001
Alpha virt. eigenvalues -- 2.85803 2.88334 2.88655 2.91844 2.94668
Alpha virt. eigenvalues -- 2.94944 2.98536 2.99067 2.99482 2.99912
Alpha virt. eigenvalues -- 3.03048 3.03356 3.07572 3.08300 3.11475
Alpha virt. eigenvalues -- 3.17335 3.27544 3.31407 3.32037 3.35160
Alpha virt. eigenvalues -- 3.35793 3.43274 3.51744 3.53892 3.89852
Alpha virt. eigenvalues -- 4.05024 4.05418 4.08851 4.08977 4.10101
Alpha virt. eigenvalues -- 4.10410 4.10621 4.12847 4.13704 4.15185
Alpha virt. eigenvalues -- 4.15352 4.16312 4.16514 4.18678 4.19238
Alpha virt. eigenvalues -- 4.20432 4.20557 4.21329 4.21670 4.21934
Alpha virt. eigenvalues -- 4.25316 4.31113 4.33250 4.35857 4.36083
Alpha virt. eigenvalues -- 4.38934 4.39179 4.39201 4.41992 4.46352
Alpha virt. eigenvalues -- 4.46846 4.47994 4.50660 4.50686 4.55606
Alpha virt. eigenvalues -- 4.64763 4.67663 4.78767 4.82192 4.82420
Alpha virt. eigenvalues -- 4.85533 4.88876
Beta occ. eigenvalues -- -88.90978 -14.30912 -14.30911 -10.20590 -10.20558
Beta occ. eigenvalues -- -10.20053 -10.20053 -10.18815 -10.18815 -10.18777
Beta occ. eigenvalues -- -10.18777 -10.18617 -10.18617 -10.18568 -10.18568
Beta occ. eigenvalues -- -10.18490 -10.18490 -10.18435 -10.18435 -10.18390
Beta occ. eigenvalues -- -10.18390 -10.18117 -10.18117 -10.18110 -10.18110
Beta occ. eigenvalues -- -10.18011 -10.18011 -10.17976 -10.17976 -10.17898
Beta occ. eigenvalues -- -10.17898 -10.17513 -10.17513 -10.17142 -10.17142
Beta occ. eigenvalues -- -10.17110 -10.17109 -10.16967 -10.16967 -10.16862
Beta occ. eigenvalues -- -10.16862 -10.16350 -10.16294 -7.96620 -5.93245
Beta occ. eigenvalues -- -5.92692 -5.92072 -0.94836 -0.87991 -0.87989
Beta occ. eigenvalues -- -0.84111 -0.83939 -0.82812 -0.81857 -0.80724
Beta occ. eigenvalues -- -0.80202 -0.77629 -0.76698 -0.74234 -0.74232
Beta occ. eigenvalues -- -0.74074 -0.73831 -0.73599 -0.73339 -0.72659
Beta occ. eigenvalues -- -0.71430 -0.70762 -0.67775 -0.67577 -0.67533
Beta occ. eigenvalues -- -0.63366 -0.62275 -0.61004 -0.60940 -0.60908
Beta occ. eigenvalues -- -0.59790 -0.59662 -0.58217 -0.57650 -0.57044
Beta occ. eigenvalues -- -0.56216 -0.55520 -0.54464 -0.53868 -0.52939
Beta occ. eigenvalues -- -0.49927 -0.49876 -0.48119 -0.47773 -0.47068
Beta occ. eigenvalues -- -0.47033 -0.45953 -0.45395 -0.44898 -0.44279
Beta occ. eigenvalues -- -0.44213 -0.44193 -0.43947 -0.43784 -0.43419
Beta occ. eigenvalues -- -0.43034 -0.42741 -0.42046 -0.41854 -0.40932

Beta occ. eigenvalues -- -0.40682 -0.40409 -0.39981 -0.39964 -0.39570
Beta occ. eigenvalues -- -0.39402 -0.39111 -0.38755 -0.38620 -0.37556
Beta occ. eigenvalues -- -0.37379 -0.37057 -0.36334 -0.36137 -0.36110
Beta occ. eigenvalues -- -0.36044 -0.35948 -0.35883 -0.34963 -0.34441
Beta occ. eigenvalues -- -0.34271 -0.34127 -0.34120 -0.33711 -0.33391
Beta occ. eigenvalues -- -0.33364 -0.33055 -0.32674 -0.31249 -0.30933
Beta occ. eigenvalues -- -0.30865 -0.30602 -0.30333 -0.29906 -0.29741
Beta occ. eigenvalues -- -0.29354 -0.27329 -0.26952 -0.26281 -0.25070
Beta occ. eigenvalues -- -0.24314 -0.24295 -0.23562 -0.23433 -0.22323
Beta occ. eigenvalues -- -0.21894 -0.20759 -0.16725
Beta virt. eigenvalues -- -0.05520 -0.02249 -0.02049 -0.00252 -0.00248
Beta virt. eigenvalues -- 0.01579 0.01605 0.02755 0.03834 0.04331
Beta virt. eigenvalues -- 0.05342 0.08318 0.08975 0.09000 0.10214
Beta virt. eigenvalues -- 0.10328 0.10428 0.11402 0.11417 0.12818
Beta virt. eigenvalues -- 0.13164 0.13358 0.13390 0.13485 0.13938
Beta virt. eigenvalues -- 0.14579 0.14883 0.15024 0.15304 0.15306
Beta virt. eigenvalues -- 0.15723 0.15751 0.17375 0.17486 0.17537
Beta virt. eigenvalues -- 0.17587 0.17972 0.18292 0.18431 0.18654
Beta virt. eigenvalues -- 0.18706 0.19488 0.19658 0.19793 0.20225
Beta virt. eigenvalues -- 0.20584 0.21110 0.21210 0.21337 0.21455
Beta virt. eigenvalues -- 0.22051 0.22409 0.23041 0.23379 0.23740
Beta virt. eigenvalues -- 0.23776 0.25387 0.25390 0.25755 0.26017
Beta virt. eigenvalues -- 0.26632 0.26852 0.27061 0.27109 0.27287
Beta virt. eigenvalues -- 0.28142 0.28522 0.28616 0.28729 0.29013
Beta virt. eigenvalues -- 0.29670 0.30207 0.30639 0.30925 0.31021
Beta virt. eigenvalues -- 0.31442 0.31715 0.32656 0.33323 0.33672
Beta virt. eigenvalues -- 0.35065 0.36173 0.36211 0.37262 0.37989
Beta virt. eigenvalues -- 0.38365 0.38634 0.40376 0.40652 0.40842
Beta virt. eigenvalues -- 0.41691 0.42996 0.44188 0.45864 0.46668
Beta virt. eigenvalues -- 0.47140 0.47861 0.48825 0.49272 0.49446
Beta virt. eigenvalues -- 0.50369 0.50397 0.51414 0.51427 0.51968
Beta virt. eigenvalues -- 0.51979 0.52396 0.52761 0.53423 0.54431
Beta virt. eigenvalues -- 0.54554 0.55189 0.55256 0.55414 0.55547
Beta virt. eigenvalues -- 0.55805 0.55842 0.56018 0.56211 0.56491
Beta virt. eigenvalues -- 0.56674 0.56875 0.57752 0.57879 0.58178
Beta virt. eigenvalues -- 0.58272 0.58854 0.59119 0.59342 0.59470
Beta virt. eigenvalues -- 0.59713 0.59860 0.60747 0.60844 0.61002
Beta virt. eigenvalues -- 0.61118 0.61337 0.61552 0.61633 0.61740
Beta virt. eigenvalues -- 0.62087 0.62089 0.62291 0.62356 0.62542
Beta virt. eigenvalues -- 0.62701 0.62760 0.62896 0.63427 0.63753
Beta virt. eigenvalues -- 0.63770 0.65260 0.65525 0.66006 0.66189
Beta virt. eigenvalues -- 0.66295 0.67005 0.67078 0.67522 0.67932
Beta virt. eigenvalues -- 0.68309 0.68578 0.69525 0.70340 0.70358
Beta virt. eigenvalues -- 0.71098 0.71671 0.71947 0.72660 0.73216
Beta virt. eigenvalues -- 0.73265 0.74465 0.74894 0.76056 0.77725
Beta virt. eigenvalues -- 0.77824 0.78495 0.79636 0.79957 0.80201

Beta virt. eigenvalues --	0.80278	0.80431	0.80815	0.81189	0.81539
Beta virt. eigenvalues --	0.81697	0.82146	0.82325	0.82469	0.82613
Beta virt. eigenvalues --	0.83148	0.84181	0.84619	0.84683	0.85623
Beta virt. eigenvalues --	0.85724	0.86236	0.86495	0.87001	0.87018
Beta virt. eigenvalues --	0.87548	0.87782	0.88170	0.88288	0.89208
Beta virt. eigenvalues --	0.89285	0.89654	0.89750	0.90204	0.90591
Beta virt. eigenvalues --	0.90658	0.91185	0.91534	0.91621	0.91830
Beta virt. eigenvalues --	0.92142	0.93078	0.93730	0.94191	0.94351
Beta virt. eigenvalues --	0.94637	0.94814	0.95500	0.95684	0.96402
Beta virt. eigenvalues --	0.96675	0.97232	0.97246	0.98400	0.98618
Beta virt. eigenvalues --	0.98884	0.99087	0.99417	0.99486	1.00113
Beta virt. eigenvalues --	1.00118	1.00793	1.01564	1.02044	1.02455
Beta virt. eigenvalues --	1.02660	1.03288	1.03491	1.03860	1.04665
Beta virt. eigenvalues --	1.04989	1.05515	1.05648	1.06166	1.06613
Beta virt. eigenvalues --	1.06639	1.06923	1.07070	1.07694	1.08900
Beta virt. eigenvalues --	1.09255	1.10281	1.10749	1.10863	1.11105
Beta virt. eigenvalues --	1.11624	1.12555	1.13639	1.14171	1.14556
Beta virt. eigenvalues --	1.15442	1.16428	1.16674	1.17551	1.17993
Beta virt. eigenvalues --	1.18898	1.18913	1.19403	1.20741	1.20837
Beta virt. eigenvalues --	1.23104	1.23845	1.24139	1.24180	1.24940
Beta virt. eigenvalues --	1.27485	1.27594	1.28155	1.28263	1.29722
Beta virt. eigenvalues --	1.30599	1.31407	1.33484	1.33765	1.34341
Beta virt. eigenvalues --	1.35671	1.36487	1.37439	1.37731	1.38750
Beta virt. eigenvalues --	1.39301	1.40309	1.40503	1.42233	1.42714
Beta virt. eigenvalues --	1.43015	1.43926	1.45472	1.45621	1.45791
Beta virt. eigenvalues --	1.46005	1.46607	1.47740	1.47927	1.48207
Beta virt. eigenvalues --	1.48644	1.49512	1.49555	1.49923	1.50118
Beta virt. eigenvalues --	1.50966	1.50992	1.52399	1.52871	1.53337
Beta virt. eigenvalues --	1.55227	1.56011	1.56891	1.58442	1.59181
Beta virt. eigenvalues --	1.60567	1.62113	1.62792	1.62803	1.65526
Beta virt. eigenvalues --	1.66486	1.66549	1.67977	1.70505	1.70554
Beta virt. eigenvalues --	1.72619	1.72924	1.73184	1.73664	1.73808
Beta virt. eigenvalues --	1.75195	1.75539	1.75961	1.76856	1.77236
Beta virt. eigenvalues --	1.77439	1.78720	1.79019	1.79598	1.80451
Beta virt. eigenvalues --	1.80752	1.81417	1.81613	1.81917	1.82203
Beta virt. eigenvalues --	1.82285	1.82371	1.82605	1.84215	1.85004
Beta virt. eigenvalues --	1.85252	1.85445	1.87367	1.89130	1.89190
Beta virt. eigenvalues --	1.90044	1.90504	1.91091	1.91867	1.92341
Beta virt. eigenvalues --	1.92452	1.93110	1.93688	1.93813	1.95073
Beta virt. eigenvalues --	1.95149	1.95321	1.95383	1.95682	1.96045
Beta virt. eigenvalues --	1.97101	1.97190	1.99154	1.99261	1.99422
Beta virt. eigenvalues --	1.99442	2.00651	2.02058	2.02479	2.02939
Beta virt. eigenvalues --	2.03024	2.03415	2.04707	2.04832	2.06077
Beta virt. eigenvalues --	2.06349	2.07054	2.07726	2.08980	2.09213
Beta virt. eigenvalues --	2.09786	2.10225	2.11385	2.12339	2.13535
Beta virt. eigenvalues --	2.13674	2.13728	2.13941	2.15011	2.15051

Beta virt. eigenvalues --	2.17093	2.18276	2.19248	2.19909	2.19930
Beta virt. eigenvalues --	2.21712	2.22003	2.23902	2.24699	2.24737
Beta virt. eigenvalues --	2.24947	2.25226	2.25969	2.26476	2.26526
Beta virt. eigenvalues --	2.26761	2.27038	2.28042	2.28591	2.29406
Beta virt. eigenvalues --	2.30111	2.30343	2.30817	2.30848	2.31524
Beta virt. eigenvalues --	2.31940	2.31957	2.32102	2.32610	2.32783
Beta virt. eigenvalues --	2.33803	2.33885	2.34871	2.35172	2.36019
Beta virt. eigenvalues --	2.37448	2.37595	2.38671	2.39263	2.39674
Beta virt. eigenvalues --	2.40866	2.41574	2.42289	2.43035	2.43170
Beta virt. eigenvalues --	2.44553	2.46213	2.47196	2.47317	2.48650
Beta virt. eigenvalues --	2.49766	2.50524	2.52053	2.52318	2.54336
Beta virt. eigenvalues --	2.54818	2.55257	2.57477	2.58702	2.59798
Beta virt. eigenvalues --	2.59874	2.61164	2.61983	2.63490	2.64499
Beta virt. eigenvalues --	2.65309	2.65673	2.66888	2.67770	2.68216
Beta virt. eigenvalues --	2.68695	2.68965	2.70710	2.71502	2.71814
Beta virt. eigenvalues --	2.71842	2.73836	2.73839	2.75184	2.75197
Beta virt. eigenvalues --	2.75621	2.76069	2.76815	2.77411	2.78032
Beta virt. eigenvalues --	2.78566	2.79413	2.80795	2.82518	2.83037
Beta virt. eigenvalues --	2.85047	2.85864	2.88369	2.88683	2.91918
Beta virt. eigenvalues --	2.94718	2.94968	2.98560	2.99079	2.99498
Beta virt. eigenvalues --	2.99944	3.03150	3.03443	3.07597	3.08565
Beta virt. eigenvalues --	3.11549	3.17377	3.27578	3.31427	3.32069
Beta virt. eigenvalues --	3.35211	3.35808	3.43322	3.51759	3.53922
Beta virt. eigenvalues --	3.90413	4.05533	4.06061	4.08892	4.09000
Beta virt. eigenvalues --	4.10191	4.10432	4.10707	4.12912	4.13863
Beta virt. eigenvalues --	4.15192	4.15363	4.16348	4.16547	4.18716
Beta virt. eigenvalues --	4.19299	4.20482	4.20603	4.21383	4.21687
Beta virt. eigenvalues --	4.21963	4.25673	4.31161	4.33295	4.35874
Beta virt. eigenvalues --	4.36098	4.39021	4.39209	4.39225	4.42122
Beta virt. eigenvalues --	4.46361	4.46858	4.47993	4.50660	4.50686
Beta virt. eigenvalues --	4.55736	4.64777	4.67709	4.78844	4.82200
Beta virt. eigenvalues --	4.82435	4.85569	4.88963		

References

1. Heyd J., Scuseria G., Ernzerhof, E., Efficient hybrid density functional calculations in solids: Assessment of the Heyd–Scuseria–Ernzerhof screened Coulomb hybrid functional, *J. Chem. Phys.* **2004**, *121*, 1187-1192, 10.1063/1.1760074.

2. Krukau A. V., Vydrov O. A., Izmaylov A. F., Scuseria G. E.. Influence of the exchange screening parameter on the performance of screened hybrid functionals., *J. Chem. Phys.* **2006**, *125*, 224106, 10.1063/1.2404663.

3. Xu, X., Goddard III, W. A., The X3LYP extended density functional for accurate descriptions of nonbond interactions, spin states, and thermochemical properties, *Proc. Natl. Acad. Sci. USA*, **2004**, *101,* 2673-2677, 10.1073/pnas.0308730100.

4. Schmider H. L., Becke, A. D., Optimized density functionals from the extended G2 test set, *J. Chem. Phys.*, **1998**, *108,* 9624-9631, 10.1063/1.476438.

5. Hamprecht, F. A., Cohen, A., Tozer, D. J., Handy, N. C. Development and assessment of new exchange-correlation functionals, *J. Chem. Phys.*, **1998**, *109,* 6264-6271, 10.1063/1.477267.

6. Wilson, P. J., Bradley, T. J., Tozer, D. J., Hybrid exchange-correlation functional determined from thermochemical data and ab initio potentials, *J. Chem. Phys.*, **2001**, *115,* 9233-9242, 10.1063/1.412605.

7. Adamo, C., Barone V., Toward reliable adiabatic connection models free from adjustable parameters, *Chem. Phys. Lett.*, **1997**, *274,* 242-250, 1016/S0009-2614(97)00651-9.

8. Becke, A. D., Density-Functional Thermochemistry. Iii. The Role of Exact Exchange. *J. Chem. Phys.* **1993,** *98*, 5648-5652, 10.1063/1.464913.

9. Perdew, J. P.; Wang, Y., Accurate and Simple Analytic Representation of the Electron-Gas Correlation Energy. *Phys. Rev. B* **1992,** *45*, 13244-13249, 10.1103/PhysRevB.45.13244.

10. Heyd, J.; Scuseria, G. E., Efficient Hybrid Density Functional Calculations in Solids: Assessment of the Heyd–Scuseria–Ernzerhof Screened Coulomb Hybrid Functional. *J. Chem. Phys.* **2004,** *121*, 1187-1192, 10.1063/1.1760074.

11. Heyd, J.; Scuseria, G. E., Assessment and Validation of a Screened Coulomb Hybrid Density Functional. *J. Chem. Phys.* **2004,** *120*, 7274-7280, 10.1063/1.1668634.

12. Perdew, J. P.; Burke, K.; Ernzerhof, M., Generalized Gradient Approximation Made Simple. *Phys. Rev. Lett.* **1996,** *77*, 3865-3868, 10.1103/PhysRevLett.77.3865.

13. Lee, C.; Yang, W.; Parr, R. G., Development of the Colle-Salvetti Correlation-Energy Formula into a Functional of the Electron Density. *Phys. Rev. B* **1988,** *37*, 785-789, 10.1103/PhysRevB.37.785.

14. Ditchfield, R.; Hehre, W. J.; Pople, J. A., Self-Consistent Molecular-Orbital Methods. Ix. An Extended Gaussian-Type Basis for Molecular-Orbital Studies of Organic Molecules. *J. Chem. Phys.* **1971,** *54*, 724-728, 10.1063/1.1674902.

15. Schäfer, A.; Horn, H.; Ahlrichs, R., Fully Optimized Contracted Gaussian Basis Sets for Atoms Li to Kr. *J. Chem. Phys.* **1992,** *97*, 2571-2577, 10.1063/1.463096.
